# Gut microbiome signatures of extreme environment adaption in Tibetan pig

**DOI:** 10.1038/s41522-023-00395-3

**Published:** 2023-05-24

**Authors:** Fangfang Zhao, Lili Yang, Tao Zhang, Daohua Zhuang, Qunfu Wu, Jiangkun Yu, Chen Tian, Zhigang Zhang

**Affiliations:** 1 State Key Laboratory for Conservation and Utilization of Bio-Resources in Yunnan, School of Life Sciences, Yunnan University, Kunming, Yunnan 650091 China; 2 Gansu Key Laboratory of Herbivorous Animal Biotechnology, Faculty of Animal Science and Technology, Gansu Agricultural University, Lanzhou, Gansu 730070 China; 3 State Key Laboratory of Genetic Resources and Evolution, Laboratory of Evolutionary & Functional Genomics, Kunming Institute of Zoology, Chinese Academy of Sciences, Kunming, Yunnan 650201 China

**Keywords:** Metagenomics, Microbiome

## Abstract

Tibetan pigs (TPs) can adapt to the extreme environments in the Tibetan plateau implicated by their self-genome signals, but little is known about roles of the gut microbiota in the host adaption. Here, we reconstructed 8210 metagenome-assembled genomes from TPs (*n* = 65) living in high-altitude and low-altitude captive pigs (87 from China—CPs and 200 from Europe—EPs) that were clustered into 1050 species-level genome bins (SGBs) at the threshold of 95% average nucleotide identity. 73.47% of SGBs represented new species. The gut microbial community structure analysis based on 1,048 SGBs showed that TPs was significantly different from low-altitude captive pigs. TP-associated SGBs enabled to digest multiple complex polysaccharides, including cellulose, hemicellulose, chitin and pectin. Especially, we found TPs showed the most common enrichment of phyla Fibrobacterota and Elusimicrobia, which were involved in the productions of short- and medium-chain fatty acids (acetic acid, butanoate and propanoate; octanomic, decanoic and dodecanoic acids), as well as in the biosynthesis of lactate, 20 essential amino acids, multiple B vitamins (B1, B2, B3, B5, B7 and B9) and cofactors. Unexpectedly, Fibrobacterota solely showed powerful metabolic capacity, including the synthesis of acetic acid, alanine, histidine, arginine, tryptophan, serine, threonine, valine, B2, B5, B9, heme and tetrahydrofolate. These metabolites might contribute to host adaptation to high-altitude, such as energy harvesting and resistance against hypoxia and ultraviolet radiation. This study provides insights into understanding the role of gut microbiome played in mammalian high-altitude adaptation and discovers some potential microbes as probiotics for improving animal health.

## Introduction

Tibetan pig (*Sus scrofa domesticus*) is an indigenous breed native to the Qinghai-Tibet Plateau that can survive under the long-term high-altitude harsh environments, such as hypoxia, severe coldness, intense ultraviolet radiation (UVR), and food scarcity^1–3^. Hence, understanding the high-altitude adaptation mechanism of Tibetan pig is very important for the discovery of novel genetic components involved in stress resistance. Genomic analysis has discovered 268 genes under positive selection in Tibetan wild boars that are related to adaptations to high-altitude, such as maintaining genomic stability against UVR and molecular adaptation under hypoxia^4^. More specifically, this study identified three vitamin B6 binding genes (*ALB*, *SPTLC2* and *GLDC*) that aid in the synthesis of hemoglobin and enhances oxygen binding and four hypoxia-related genes (*ALB*, *ECE1*, *GNG2* and *PIK3C2G*). Further study found genetic mutations in *PLA2G12A* and *EPAS1* genes related to phenotypic variation of pulmonary vascular tone and hemoglobin concentration^5^. Recently, additional studies also paid attention to the gut microbiome signatures of high-altitude adaptation in Tibetan pig, owing to the essential role gut microbiota played in nutritional metabolism^6,7^, energy regulation^8,9^ and immune system development for maintaining host health^10,11^. For instance, a 16 S ribosomal meta-analysis revealed the three most abundant gut bacteria *Acinetobacter*, *Pseudomonas*, and *Sphingobacterium* in Tibetan pig in comparison to low-altitude pig^12^. The metabolomics analysis of 12 fecal samples from high-altitude Tibetan pig demonstrated that the productions of propanoic acid and octadecanoic acid were significantly improved and genes related to these two metabolites were also up-regulated^12^. In short, these findings indicated high-altitude environments shaped unique gut microbiome and functional diversity of Tibetan pig. However, it remains unclear about the diversity and functional landscape in the gut microbiota in Tibetan pig and its signature for host high-altitude adaptation, due to the limitations of small sample size and low metagenomic sequencing depth in the previous studies.

Pigs are the major domestic animals worldwide that serve as an important component of global livestock production. However, the rapid livestock production growth over the past decades also has led to the emergence and spread of infectious diseases, such as the porcine epidemic diarrhea virus, and directly brings huge economic losses to the pig-farming industry^13,14^. It drives the most common applications of antibiotic administration in pig production for treatment and prevention of infection in suckling and post-weaning periods. Antibiotic usage contributes to the increasing microbial resistance and the decrease in microbial diversity^15,16^, likewise, it can pose a risk to pig health. To lower the byproduct effect of antibiotic use, Blaser^17^ recommended probiotics to replace vital missing and/or extinct species and strains that modulated crucial developmental pathways, perhaps with accompanying prebiotics. The wild relatives or native breeds of domesticated animals might represent a huge resource for the exploration of probiotics. For instance, Rosshart et al.^18^ reported the eventual engraftment of wild mouse gut microbiome into laboratory mice could promote host fitness and improve the disease resistance of pulmonary infection and tumorigenesis. The transferring fecal microbes from a native pig breed which had stronger disease resistance into commercial piglets could prevent recurring diarrhea induced by early weaning stress^19^. Recently, over 13,000 carbohydrate-degrading genes were identified in the gut microbiome of Tibetan pig (*n*=11), which were mostly distributed in five phyla: Firmicutes, Bacteroidetes, Spirochaetota, Verrucomicrobiota, and Fibrobacterota^20^. These studies indicated Tibetan pig might harbor unexpected probiotic microbes for the improvement of pig health. However, up to now, a systemic genome-level investigation on the gut microbiome of Tibetan pig is still lacking.

Here, we performed deep metagenomic sequencing of 31 fecal and 34 cecum content samples from 47 freely grazing Tibetan pigs (TPs) located in the Deqen prefecture of Yunnan province in China, with an average elevation of over 3500 m above sea level. We recovered 1048 species-level genome bins (SGBs) from pig gut microbiome by integrating published gut metagenomes from 287 low-altitude captive pigs (87 from China -CPs and 200 from Europe -EPs). We discovered distinct diversity and functional profiles of gut microbiota in TPs compared to both CPs and EPs. The results will not only provide insights into understanding the high-altitude adaptation of Tibetan pigs but also explore the potential of TP-associated gut microbiota as probiotics.

## Results

### 1050 SGBs clustered from 8210 high-medium quality metagenome-assembled genomes (MAGs)

**Fig. 1:**
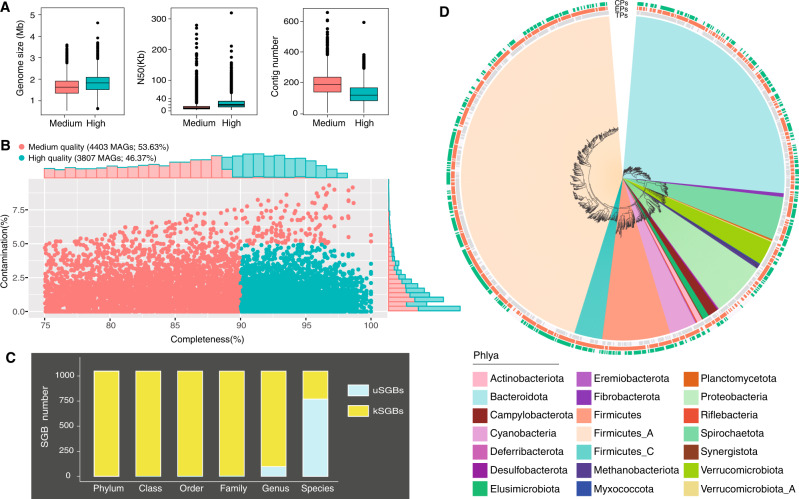
Assessment of MAGs quality and taxonomic annotation of SGBs. **A** Profiles of medium and high-quality of MAGs. Genome size, N50 and contigs number of medium and high-quality genomes with different colors indicating different genome quality. The related source data is provided as a source data file. **B** Estimated completeness and contamination of 8210 genomes recovered from pig gut metagenomes. Genome quality was scored as completeness − 5 contamination, and only genomes with a quality score of above 75% were retained. Medium-quality genomes are shown in green, and high-quality genomes in red. Histograms along the x and y axes showing the percentage of genomes at varying levels of completeness and contamination, respectively. The related source data is provided as a source data file. **C** Numbers of SGBs in each taxonomic rank. The SGBs without existing reference genomes at species-level by GTDB-Tk were defined as unknown SGBs (uSGBs), instead, the SGBs having at least one reference genome were considered as known SGBs (kSGBs). **D** Phylogenetic tree of pig gut representative SGBs. Inner circle is a phylogenetic tree of 1048 representative SGBs colored according to GTDB phylum-level taxonomic classifications (see color legend). Concentric rings moving outward from first to third ring representing group enriched SGBs according to the presence/absence of SGBs in each group. TPs represents freely grazing Tibetan pigs, EPs represents low-altitude captive European pigs, and CPs represents low-altitude captive Chinese pigs. The related source data is provided as a source data file.

Over 779 Gb metagenomic sequence data was generated by Illumina HiSeq × Ten platform from 65 TPs samples (Supplementary Table 1). We integrated the above sequencing raw reads with a published metagenomic sequence dataset of 287 captive pigs from Denmark, France and China^21^ to generate the gut genome catalogue of pigs. Using a single-sample assembly strategy, we reconstructed a total of 8210 MAGs with a quality threshold of completeness >75% and contamination <10%^22,23^ (see “Methods”; Fig. 1A). In total, 3807 of these MAGs were high-quality genomes with >90% completeness with <5% contamination (Fig. 1B). After de-replication at an average nucleotide identity (ANI) threshold of 95%^24^, 1050 SGBs were identified for further analysis (see “Methods”). We used at least 40% genome coverage to determine the presence of SGBs in each sample, and 1048 representative SGBs were finally obtained, of which 623 SGBs (59.45%) were high-quality genomes (>90% completeness and <5% contamination) (Supplementary Table 2). Each SGB was supported by an average of 7.8 MAGs and 57.25% of SGBs contained at least two MAGs (Supplementary Table 2). We used the genome taxonomy database toolkit (GTDB-Tk)^25^ to perform taxonomic assignment of the SGBs (see “Methods)”. The results showed they were classified into 20 bacterial phyla and one archaea phylum, 90.74% of SGBs were assigned to known genera, and 73.47% of SGBs were unclassified species (named uSGBs) (Fig. 1C). Besides, 45.04% of 1048 SGBs were assigned into Firmicutes A, 25.86% to Bacteroidetes, 6.97% to Firmicutes, and 5.63% to Proteobacteria (Supplementary Table 3). The prevalence and classification of SGBs in TPs, EPs and CPs were shown in Fig. 1D, indicating the differences of microbial community at phylum-level between three groups. Additionally, functional gene profiles of 1048 SGBs were predicted using MetaGeneMark (v.3.38)^26^ (see “Methods”). All gene annotations were performed by using the Carbohydrate-active enzymes (CAZymes)^27^ and Kyoto Encyclopedia of Genes and Genomes (KEGG) Orthology (KO)^28^ databases (see “Methods”).

Chen et al.^29^ identified 6339 MAGs (>50% completeness and <5% contamination) and 2673 SGBs (ANI ≥ 95%) from gut microbiome dataset from 500 Chinese pigs. To explore the uniqueness of our identified SGBs, we integrated our 8210 MAGs with Chen’s dataset and kept the MAGs with >75% completeness and <5% contamination for the identification of SGBs at the threshold of ANI ≥ 95%. A total of 2266 SGBs were finally obtained. 1248 (55.08%) of them were unique to Chen’s study, 519 (22.90%) to this study and 499 (22.02%) were overlapped (Supplementary Fig. 1). This finding indicates that ongoing efforts are needed for understanding the pig gut microbial diversity.

### The differences in microbial community between TPs, EPs and CPs

**Fig. 2:**
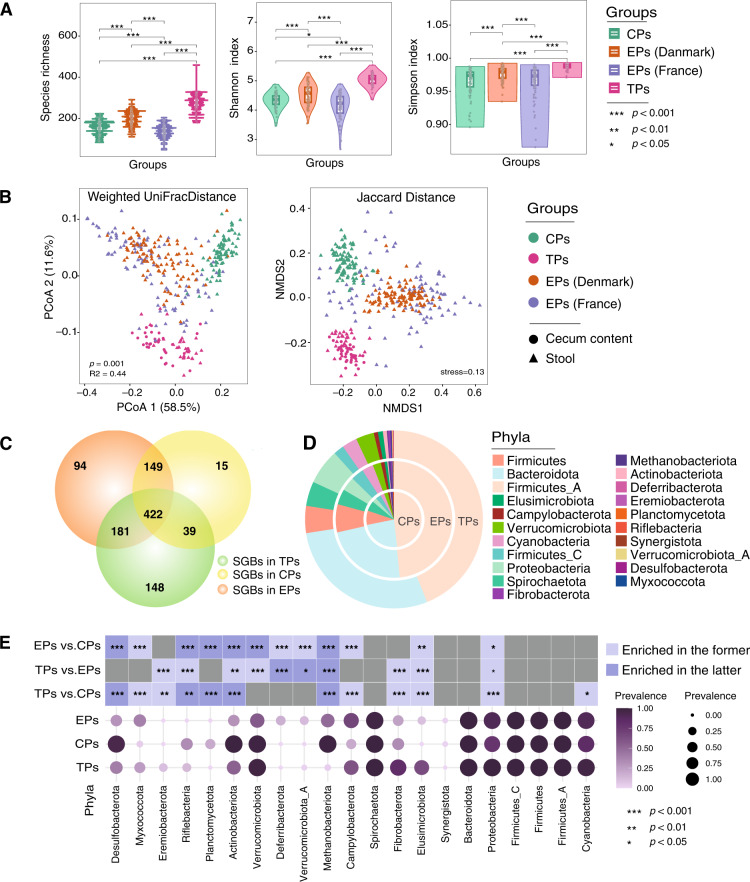
Microbial composition and diversity of TPs, EPs and CPs. **A** Comparison of alpha diversity indices of gut microbiome among TPs, EPs and CPs. The violin plot and box plot representing the Species richness, Shannon and Simpson diversity index, respectively. Species Richness bar plot based on the presence or absence of SGBs in each sample, Shannon and Simpson diversity index based on relative abundance of SGBs in each sample. The colors indicate host-groups. The related source data is provided as a source data file. **B** Comparison of beta diversity among TPs, EPs and CPs. PCoA was based on weighted UniFrac distance matrix among samples. The colors and shapes indicate host-groups and sample types, respectively. PCoA plotting showing the microbial community of TPs separated from those of EPs and CPs (Per mutational multivariate analysis of variance, *p* = 0.001, adjusted R^2^ = 0.44). **B** NMDS plotting analysis was based on Jaccard distance matrix among samples. The colors and shapes indicate host-groups and sample types, respectively. NMDS plotting demonstrates the microbial community of TPs separate from EPs and CPs (stress=0.13). The related source data is provided as a source data file. **C** Distribution of SGBs among TPs, EPs and CPs. Venn diagram showing the prevalence of SGBs in TPs, EPs, and CPs. The colors indicate sample group. **D** Distribution of microbiota at phylum-level among TPs, EPs and CPs. Pie chart showing the prevalence of phyla in TPs, EPs, and CPs. **E**, Comparison of enrichment analysis at phylum-level among TPs, EPs and CPs. Bubble plot color and size corresponding to the prevalence of SGBs in a certain phylum. Heat map shows the enrichment significance level (no * representing *p* > 0.05), and the colors indicate enriched groups.

The alpha and beta diversity analyses of gut microbiota from four groups (TPs, CPs, EPs-Denmark, EPs-France) were performed based on the prevalence or abundance of SGBs (see “Methods”). The results demonstrated that Species Richness, Shannon and Simpson diversity indexes were significantly increased in TPs in comparison to other groups (Fig. 2A, Wilcoxon rank-sum test, *p* < 0.001). The principal co-ordinates analysis (PCoA) (based on the Weighted UniFrac distance matrix) and non-metric multidimensional scaling (NMDS) plotting analysis (based on Jaccard distance matrix) consistently indicated a clear separation of TPs from the other groups (Fig. 2B, Supplementary Fig. 2A, B). Moreover, the difference in the microbial diversity between sample types (fecal and cecum) was significantly lower than that between host-groups (Supplementary Fig. 2C–F), suggesting that the TPs harbor distinct gut microbiota. For instance, 14.12%, 8.97% and 1.43% of all SGBs were present uniquely in TPs, EPs and CPs, respectively (Fig. 2C). According to the presence or absence of each SGB in each sample, we found that 464 SGBs were significantly associated with TPs, 209 SGBs with EPs, and 146 SGBs with CPs (Supplementary Table 4, Wilcoxon rank-sum test, *p* < 0.05, FDR corrected). At phylum-level, the prevalence of Fibrobacterota and Elusimicrobia was significantly higher in TPs than that in EPs and CPs (Fig. 2D; Fig. 2E, Fisher’s test, *p* < 0.001). Instead, Methanobacteriota known for its role in methanogenesis^30^ was extremely significantly less in TPs than in other groups (Fisher’s test, *p* < 0.001). CPs had the highest prevalence of Methanobacteriota (Fisher’s test, *p* < 0.001). Both TPs and CPs had higher prevalence of Actinobacteriota and Verrucomicrobiota than EPs (Fisher’s test, *p* < 0.01). The Desulfobacterota and Planctomycetotah existed mainly in CPs (Fisher’s test, *p* < 0.001), and the Deferribacterota and Verrucomicrobiota_A were prominently present in EPs (Fisher’s test, *p* < 0.05).

### Profiling of CAZymes in host-groups-associated SGBs

**Fig. 3:**
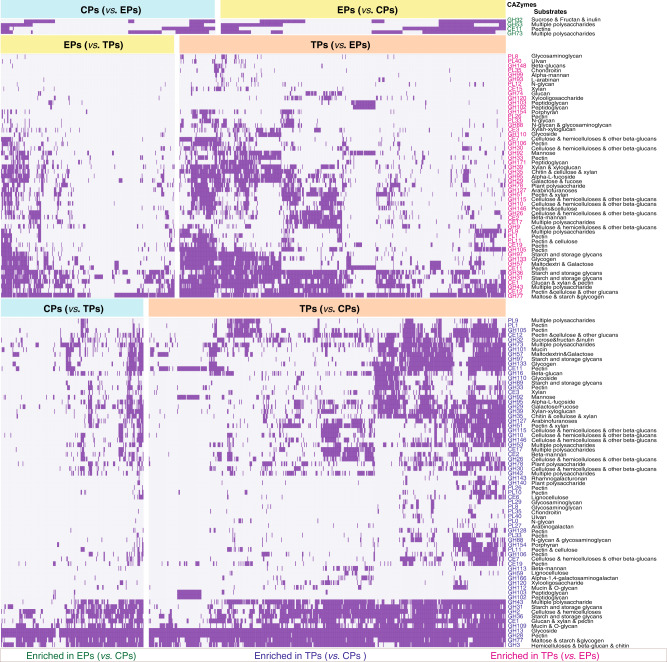
Significant difference in CAZymes and potential substrates between TPs, EPs, and CPs. Heat map showing the significant difference in CAZymes (except glycosyltransferases) between TPs, EPs and CPs, and their related potential substrates. It only demonstrates the significantly enriched CAZymes in the former host-group.

To investigate the potential of carbohydrate utilization of host-groups-associated SGBs, we performed CAZyme annotation using dbCAN2^31^ and performed enrichment analysis of CAZymes via Fisher’s test (see “Methods”). The results demonstrated that TPs-associated SGBs encoded much more carbohydrate utilization genes than captive pigs-associated SGBs, and the genes mainly belonged to glycoside hydrolases (GHs) families, carbohydrate esterases (CEs) families and polysaccharide lyases (PLs) families (Supplementary Table 5 and Fig. 3, Fisher’s test, *p* < 0.05). For example, TPs-associated SGBs included more genes in GH43, CE12, GH105, GH88, GH35 and GH51 than CPs and EPs (Fisher’s test, *p* < 0.001). They also included more genes in CE1, PL1, and GH53 than CPs, and genes in GH127 and GH146 than EPs, respectively (Fisher’s test, *p* < 0.05). Based on the CAZy and dbCAN-PUL database, we found that all the CAZymes of GH43, CE12, GH105, GH35 and GH51 were associated with the degradation of plant polysaccharides, such as cellulose, pectin, chitin, xylan and other glucans. GH88 is related to the utilization of N-glycan and glycosaminoglycan. CE1, PL1, GH53, GH127 and GH146 are involved in the degradation of multiple polysaccharides, such as cellulose, hemicelluloses and other beta-glucans. These findings suggest that the TPs gut microbiome have significantly stronger polysaccharide utilization capability than captive pigs.

### Functional potential profile of TPs-associated bacteria

**Fig. 4:**
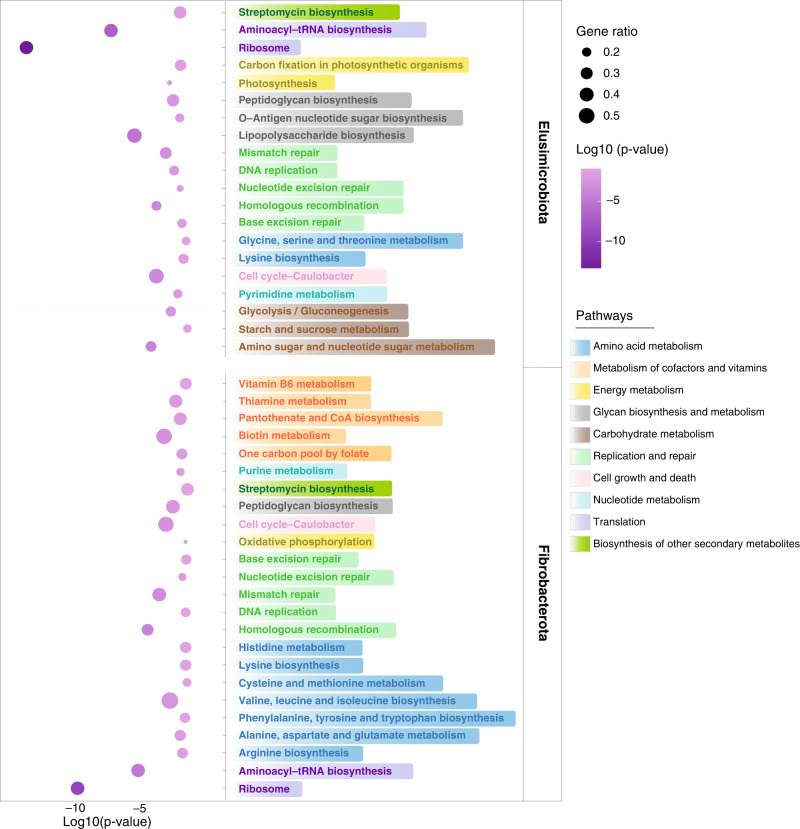
Functional profiles of Elusimicrobia and Fibrobacterota based on KEGG pathways. Bubble plot showing the significantly enriched KEGG pathways in Elusimicrobia and Fibrobacterota. Enrichment significance (*p* value) was measured with Fisher’s test (see “Methods”). Bubble color responds to the enrichment significance and bubble size is related to the ratio of the number of genes mapped to a certain pathway. The same color of metabolism pathways in right indicates same pathway module.

The Fibrobacterota and Elusimicrobia existed mainly in TPs rather than other groups (Fisher’s test, *p* < 0.001). Notably, the SGBs in the two bacteria phyla were all of high quality with > 90% completeness and < 5% contamination (Supplementary Table 2). Hence, we performed KO annotation and enrichment analysis of KEGG pathways to decipher their functional potential. The results showed that there were 38 KEGG pathways significantly enriched in the SGBs of Fibrobacterota and 39 pathways in the SGBs of Elusimicrobia, respectively (Supplementary Table 6, Fisher’s test, *p* < 0.05, FDR corrected). Overall, the two phyla bacteria exhibited different metabolic characteristics, even though the most enriched ten pathways in them both included translation, replication and repair, signal transduction and energy metabolism (Fig. 4, Fisher’s test, *p* < 0.001). Fibrobacterota was particularly involved in the metabolism of cofactors and vitamins, as well as amino acid metabolism, and Elusimicrobia mostly participated in glycan biosynthesis and metabolism, as well as carbohydrate metabolism. Additionally, enriched pathways involved in amino acid metabolism were distinct between Fibrobacterota and Elusimicrobia (Fig. 4 and Supplementary Table 6, Fisher’s test, *p* < 0.05, FDR corrected). For example, Fibrobacterota was significantly enriched in the pathways of valine, leucine and isoleucine biosynthesis (Fisher’s test, *p* = 0.002), alanine, aspartate and glutamate metabolism (Fisher’s test, *p* = 0.010), arginine biosynthesis (Fisher’s test, *p* = 0.014), phenylalanine, tyrosine and tryptophan biosynthesis (Fisher’s test, *p* = 0.023), lysine biosynthesis (Fisher’s test, *p* = 0.025), histidine metabolism (Fisher’s test, *p* = 0.025), cysteine and methionine metabolism (Fisher’s test, *p* = 0.032). Elusimicrobia preferred to lysine biosynthesis (Fisher’s test, *p* = 0.0174), and glycine, serine and threonine metabolism (Fisher’s test, *p* = 0.0277).

### Synthesis of energy sources and key precursor metabolites of SGBs in phyla Fibrobacteres and Elusimicrobiota

**Fig. 5:**
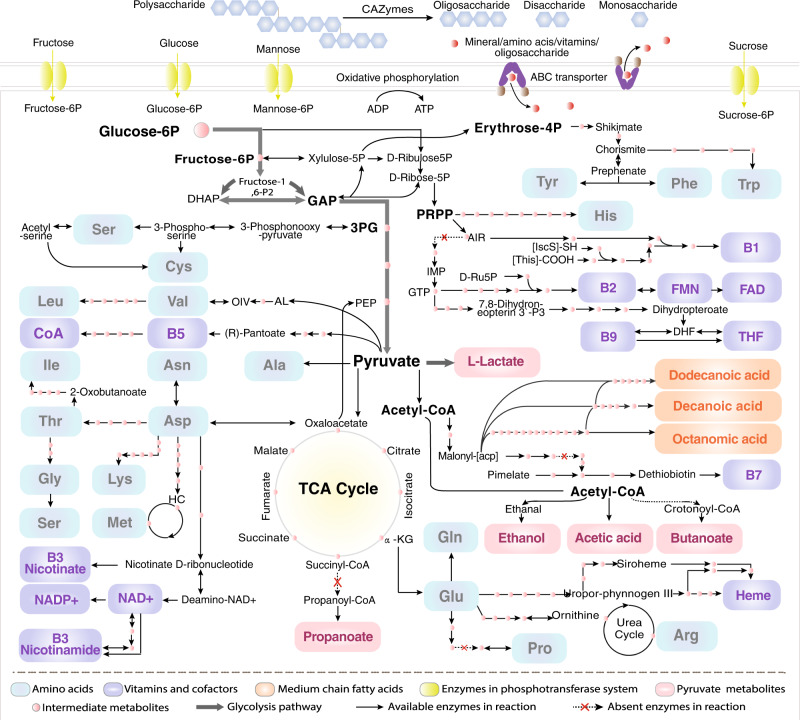
Metabolic pathway overview of TPs-associated bacteria. Elusimicrobia and Fibrobacterota were involved in polysaccharide degradation, membrane transport, glycolysis, and TCA cycle, as well as anabolism of fatty acids, amino acids, and B vitamins and cofactors. DHAP dihydroxyacetone phosphate, GAP glyceraldehyde 3-phosphate, PRPP 5-phosphoribosyl diphosphate, 3PG 3-phosphoglycerate, PEP phosphoenolpyruvate, AIR amino imidazole ribonucleotide, IMP inosine monophosphate, GTP guanosine 5′-triphosphate, D-Ru5P d-ribulose 5-phosphate, FAD flavin adenine dinucleotide, FMN riboflavin-5-phosphate, DHF 7,8-Dihydrofolate, THF tetrahydrofolate, AL (S)-2-acetolactate, OIV 2-oxoisovalerate, NAD+ nicotinamide adenine dinucleotide, NADP+ nicotinamide adenine dinucleotide phosphate.

Metabolic pathway analysis demonstrated that the SGBs in Fibrobacteres and Elusimicrobiota contained a series of key enzyme genes in the glycolysis pathway (10 genes) and pyruvate metabolism pathway (2 genes) for catalyzing the conversion of glucose 6- phosphate to pyruvate and further into short-chain fatty acids (SCFAs), medium-chain fatty acids (MCFAs), lactate, ethanol and so on (Fig. 5, Supplementary Fig. 3 and Supplementary Table 7). Moreover, they encoded numerous enzyme genes for fatty acids synthesis, the conversion of acetyl-CoA to malonyl-CoA, as well as the synthesis of octanoic acid, decanoic acid and dodecanoic acid. Nevertheless, the SGBs in Fibrobacteres encoded more enzyme genes in these pathways than in Elusimicrobiota, except the synthesis pathways of lactate, butanoate, ethanol and propanoate. It was remarkable that the SGBs in two phyla encoded some unique enzyme genes to participate in a certain catalytic process. For example, the SGBs in Fibrobacteres were solely involved in acetic acid synthesis through two pathways. One was the conversion of pyruvate directly into acetic acid. Another was pyruvate into acetyl adenylate then into acetic acid. The SGBs in Elusimicrobiota were merely involved in the direct conversion of pyruvate into lactate. Certainly, some pathways might require inter-cooperation of the two phyla, such as the butanoate synthesis. The key enzymes trans-2-enoyl-CoA reductase [EC:1.3.1.44] and butyrate kinase [EC:2.7.2.7] were annotated from the SGBs in Fibrobacteres and in Elusimicrobiota, respectively.

Additionally, abundant enzyme genes catalyzing the process of non-oxidative branches of the pentose phosphate pathway were encoded by the SGBs in Fibrobacteres and Elusimicrobiota (Supplementary Fig. 3). For example, transketolase [EC:2.2.1.1], ribulose-phosphate 3-epimerase [EC:5.1.3.1], ribose 5-phosphate isomerase A [EC:5.3.1.6], and ribose-phosphate pyrophosphokinase [EC:2.7.6.1] catalyze the conversion of fructose 6-phosphate to erythrose 4-phosphate (erythrose-4P) and to 5-Phospho-alpha-D-ribose 1-diphosphate (PRPP). It is known that erythrose-4P is the essential substrate for chorismate synthesis through the shikimate pathway. Chorismate can serve as a precursor for the biosynthesis of tyrosine, phenylalanine, and tryptophan. And PRPP is the key precursor for histidine synthesis and formation of amino imidazole ribonucleotide (AIR) and guanosine triphosphate (GTP), which are used for the biosynthesis of vitamin B1, B2, B9, flavin adenine dinucleotide (FAD), flavin mononucleotide (FMN) and tetrahydrofolate (THF).

### Synthesis of amino acids and vitamins (or cofactors) of SGBs in phyla Fibrobacteres and Elusimicrobiota

The enrichment analyses on metabolic pathways of Fibrobacteres and Elusimicrobiota illustrated that the two phyla both were involved in the synthesis of 20 essential amino acids (Fig. 5, Supplementary Fig. 4 and Supplementary Table 7). The SGBs in Fibrobacteres encoded more related enzyme genes than the ones in Elusimicrobiota. For instance, the SGBs in Fibrobacteres contained all of the key enzyme genes that catalyzed PRPP to form histidine (9 genes), oxaloacetate to glutamate and further to arginine (11 genes), oxaloacetate to aspartate (1 gene), aspartate to lysine (7 genes) and to threonine(5 genes) as well as to glycine (7 genes), 3-phosphoglycerate to serine (3 genes), pyruvate to alanine (2 genes) and to valine (5 genes), erythrose-4P to chorismate and further to tryptophan (12 genes). The results indicated Fibrobacteres had stronger capacity to synthesize amino acids than Elusimicrobiota. The SGBs in Elusimicrobiota encoded all seven key enzyme genes in lysine synthesis pathway (7 genes) and all the key enzyme genes catalyzed the conversion of threonine to glycine and further to serine, such as threonine 3-dehydrogenase [EC:1.1.1.103].

In addition, the SGBs in Fibrobacteres and Elusimicrobiota contained multiple enzyme genes in the synthesis pathways of B vitamins and their active forms with the exception of pyridoxine and cobalamin (Supplementary Fig. 4). For example, the SGBs in two bacteria phyla included 10 genes in the synthesis pathway of thiamine, 13 genes in niacin, nicotinamide, and nicotinamide adenine dinucleotide (NAD+), 11 genes in pantothenate and coenzyme A (CoA), 13 genes in biotin, as well as 19 genes in heme. It was remarkable that Fibrobacteres had all essential enzyme genes for catalyzing GTP to form riboflavin (7 genes), folate and THF (12 genes), indicating their important roles in the B vitamins synthesis. The SGBs in Elusimicrobiota contained more enzyme genes in niacin and nicotinamide synthesis.

## Discussion

In this study, we recovered 8210 MAGs from various pig breeds and clustered that into 1050 SGBs. At least 73.47% SGBs belonged to novel unidentified bacterial species. Compared to the largest dataset of pig microbiome^29^, there were still 519 SGBs unique in this study. These findings suggest that this study can enlarge the known species repertoire and expand the known phylogenetic diversity of microbiota in pig gut. Additionally, TPs had more microbial diversity than CPs and EPs, which was consistent with the finding that *Chinese rhesus macaques* in Tibet possessed higher gut microbial diversity than that in low-altitude relatives^32^. However, Zeng et al.^14^ found less microbial diversity in Tibetan pigs and Tibetans than that in their low-altitude relatives. Worthmann et al.^33^ reported that cold exposure resulted in the lower richness and a decreased Shannon diversity index in mice. Whether high-altitude drives the formation of gut microbial diversity of Tibetan animals needs to be further clarified. Besides, we found that beta diversity difference between sample types (feces vs. cecum contents) was significantly lower than that among host-groups. This was consistent with our previous finding that the difference in microbial diversity among sites along the human intestine was lower than inter-individual variation^34^.

Our previous study demonstrated the convergent evolution of rumen microbiomes in yak and Tibetan sheep for their hosts’ energy harvesting persistence^35^, indicating that energy homeostasis mediated by gut microbiomes might be vital for the high-attitude adaptation of mammals. In this study, we identified larger amounts of enzyme genes with the potential to degrade polysaccharides, cellulose and other beta-glucans, hemicellulose, chitin, and pectin in TP-associated microbial genomes, which was consistent with the previous study that the gut microbiomes of TPs encoded plentiful carbohydrate-degrading enzyme genes than those from their low-altitude relatives^20^. Moreover, we discovered a significantly high prevalence of Fibrobacterota and Elusimicrobia in the gut of TPs. They were involved in the energy producing, such as butyrate, acetic acid, propionate, ethanol and lactate. Butyrate can be absorbed by the colonic epithelium cells to maintain the integrity of the intestinal wall barrier and prevent inflammation of enterocytes^36^. Acetic acid and propionate were absorbed into the bloodstream and traveled to the liver for fueling the tissue energy metabolism^37^. Zheng et al.^38^ demonstrated Elusimicrobia could utilize glucose to ferment to lactate and acetic acid, which was consistent with our finding. Thus, we inferred that unique gut microbiota harbored in TPs could improve high-fiber diet utilization to promote energy metabolism for host adaptation to extreme environments at high-altitude.

High-altitude hypoxia and UVR are typical characteristics in the Qinghai-Tibet Plateau^2^. Severe oxygen deprivation could increase the incidence rates of lung damage^39^, cardiovascular diseases^40^ and brain injury diseases^41^. As exposure to high UVR resulted in premature aging of the skin and many other environmentally influenced skin disorders^42^. In this study, we found functional potentials of Fibrobacterota and Elusimicrobia in the production of lactate, amino acids (i.e., histidine and arginine), and vitamins/cofactors (i.e., B2, B3, B9, THF, and heme) (Fig. 5, Supplementary Fig. 3 and Supplementary Fig. 4) were associated with resistance to hypoxia and UVR damage. For example, lactate was regarded as a promoter of hypoxic response independent of hypoxia-inducible factor (HIF) by binding to NDRG3 protein to promote angiogenesis and cell growth^43^. Arginine is a precursor of nitric oxide (NO) which is regarded as the natural vasodilator to boost blood flow and oxygen delivery^44^. Histidine can be converted into histamine to play a particularly important roles in increasing blood flow and capillary permeability^45^. It also can be converted into trans-urethane and further into cis-urethane by UVR to protect skin^46^. Riboflavin and its cofactors were benefit to maintain blood cells healthy, protecting skin health, and improving the hypoxia endurance in young adults^47,48^. Folate and THF played vital roles in repressing hypoxia-induced inflammation^49^, forming red blood cells, and producing melanin for the protection of skin^50^. Nicotinamide and niacin were involved in preventing lung tissue damage^51^, as well as NAD+ in prolonging both health span and life span^52^. Heme is the important precursor to hemoglobin which is essential to oxygen sensing, oxygen transfer and electron transfer^53,54^. These findings showed an unexpected association between the gut microbiome and host adaption to high-altitude hypoxia and UVR.

Our study provides insights into understanding the role of the gut microbiome in the high-altitude adaptation of mammals and displays some candidate microbes for the exploration of probiotics in the future, such as the members from Fibrobacterota and Elusimicrobia that are significantly associated with high-altitude mammals and potentially contribute to the host adaptation to extremely harsh environments in the Qinghai-Tibet Plateau. However, we still can’t completely disentangle the effect of different lifestyles on the divergence of gut microbiome among Tibetan pigs and their low-land relatives. In the future, it is necessary to confirm our discovered associations between gut microbiome and host traits by using germ-free animal models colonized by cultured gut bacteria under controlled experiments.

## Methods

### Ethical statement

All animal experiment was approved by the Yunnan University Ethical Committee for animal experiments.

### Sample collection of Tibetan pig

Fresh cecum content samples (*n* = 34) and fecal samples (*n* = 31) were collected from 47 freely grazing Tibetan pigs (females and males) located 3500 m above sea level at the Deqen prefecture in Yunnan province in China into sterile collection tubes (Supplementary Table 1). All samples were immediately frozen in dry ice and then stored at −80 °C until use.

### DNA extraction and metagenome sequencing

DNA extractions were performed using the QIAamp DNA Stool Mini Kit (Qiagen, Hilden, Germany) according to the manufacturer’s instructions, with additional steps of mechanical disruption and ammonium acetate precipitate protein. The concentration and purity of DNA were measured using the NanoDrop (Thermo Fisher Scientific Inc., Wilmington, DE, USA) and agarose gel electrophoresis. Metagenome library preparation was conducted in one DNA paired-end library with an insert size of 300 base pairs (bp) for each sample following the manufacturer’s instructions (Illumina, San Diego, CA, USA). The sample was sequenced on an Illumina HiSeq × 10 platform adopting a 150-bp PE sequencing strategy.

### Public available pig gut metagenomes

We collected the publically available metagenomic dataset from Xiao et al. study^21^, totaling 1758 Gb high-quality reads deposited in the European Nucleotide Archive (ENA) under accession code PRJEB11755. The sequencing raw reads were generated from 287 low-altitude captive pigs (100 pigs from France, 100 pigs from Denmark and 87 pigs from China) with an average of 6.13 Gb per sample. These datasets were integrated with our raw reads for further metagenomic assembly.

### Metagenomic assembly and genome binning

Our raw reads and publically available reads were filtered to remove low-quality sequences including the ones: (1) more than 40% continuous bases whose quality score was lower than 32-bp; (2) more than 10% N bases. Then the reads were further quality controlled by using Trimmomatic for filtering poor quality reads adapters and lower quality reads^55^. We obtained a total of 2.3Tb high-quality clean reads for 352 specimens with an average of 6.6 Gb per specimen. MEGAHIT (v1.0)^56^ was used to assemble all clean reads in each sample individually. Contigs with length > 500 bp were aligned to reads using BWA(v.0.7.12)^57^. The coverage and depth of contigs were then computed by using Samtools (v.1.9)^58^ and Bedtools (v.2.27.1)^59^. We performed MetaBAT2^60^ to bin the assembled contigs into putative genomes (MAGs) within each sample based on tetranucleotide frequency and abundance(or average depth) of contigs. Subsequently, CheckM (v.1.0.7)^61^ was used to estimate the completeness and contamination of MAGs by lineage-specific markers genes and default parameters. Generally, recovered MAGs with completeness >50% and contamination < 10% were considered medium or high-quality^62–64^, but we set up a more strict threshold for medium quality as completeness > 75% and contamination < 10%^22,23^. The MAGs with completeness > 75% and contamination < 10% were retained for further refinement and validation. We used RefineM (v.0.0.14)^22^ to filter the bins with divergent genomic properties, with incongruent taxonomic classification and with incongruent 16 S rRNA genes using default parameters. CheckM (v.1.0.7) was re-run to assess the genome quality of the retained MAGs. 8210 high-medium quality MAGs (completeness 75% and contamination 10%) were obtained. Then, those MAGs were clustered into species-level genomes using dRep (v.2.6.2)^65^ with default parameters of Mash^66^ and ANIs^67^ (at the threshold of 95%). The genomes with maximum genome quality score (completeness-5X contamination + 0.5logN50) in each cluster were selected as representative SGBs. SGBs with coverage greater than 40% in a sample were determined to be present in this sample. So a total of 1048 representative SGBs in pig gut were finally reconstructed in this study (Supplementary Table 2).

### Taxonomic annotation and phylogenetic analysis of 1048 representative SGBs

GTDB-Tk (v.1.4.0)^25^ was used to perform taxonomic assignments of 1048 representative SGBs based on GTDB(release 95) (Supplementary Table 3). The phylogenetic trees of the SGBs were built by FastTree (v.2.1.10)^68^ and visualized using iTOL (v.6.6)^69^.

### Functional annotations of SGBs

Open Reading Frames (ORFs) were predicted using MetaGeneMark (v.3.38)^26^ in each SGB. All ORFs were mapped to the KEGG genes database (v.96) to annotate KOs and aligned to the CAZy database using dbCAN2^31^ to annotate CAZymes. The carbohydrate substrates of the CAZyme-containing genes were obtained by using the dbCAN-PUL database.

### Statistical analysis

The Species Richness was calculated based on the presentence or absence of each SGB in each sample (see Source data file) by Vegan package. The Shannon and Simpson diversity index was calculated based on the abundance of each SGB in each sample (see Source data file) by Vegan package. The abundance or depth of each SGB was calculated based on the formula: total base length mapped into the genome divided by the genome size. The statistical difference in alpha diversity between the four groups was measured by employing the Wilcoxon rank-sum test. The PCoA plotting analysis was performed based on the Weighted UniFrac distance matrix among samples by using the Phyloseq package. The NMDS plotting analysis was performed based on the Jaccard distance matrix among samples by using the Vegan package. The enrichment analysis of each SGBs in every group was measured based on their presence or absence in each sample. The statistical difference in each SGB enrichment between three groups was analyzed by the Wilcoxon rank-sum test. The enriched SGBs in each group were annotated CAZymes and then according to the count of annotated CAZymes in each group, we performed the enrichment analysis by using Fisher’s exact test. All SGBs of phyla Fibrobacteres and Elusimicrobiota were assigned to their KOs annotated. We used Fisher’s exact test to analyze the KEGG pathway enrichment of each phylum based on the ratio of annotated KO counts to total KO counts in the certain pathway.

### Reporting summary

Further information on research design is available in the Nature Research Reporting Summary linked to this article.

## Supplementary information

Supplementary Information

Reporting Summary

## Data Availability

Raw sequence data and MAGs profiles have been deposited into the CNGB Sequence Archive (CNSA) of China National GeneBank DataBase (CNGBdb) with accession number CNP0003325. They are also available at https://db.cngb.org/qtp/. The source data underlying Figs. 1–2 and Supplementary Figures 1–2 are provided as a Source Data file.

## Source data

Source Data file

## Code availability

The detailed information of code used to perform our data analyses is available in the supplementary materials.
