# Supplementary material for: Gut microbiome signatures of extreme environment adaption in Tibetan pig
Source: NPJ Biofilms Microbiomes. 2023 May 24;9:27. doi: 10.1038/s41522-023-00395-3 (PMC10209067; doi:10.1038/s41522-023-00395-3)
Supplement: Supplementary file 1 — Supplementary Information [file 41522_2023_395_MOESM1_ESM.pdf]

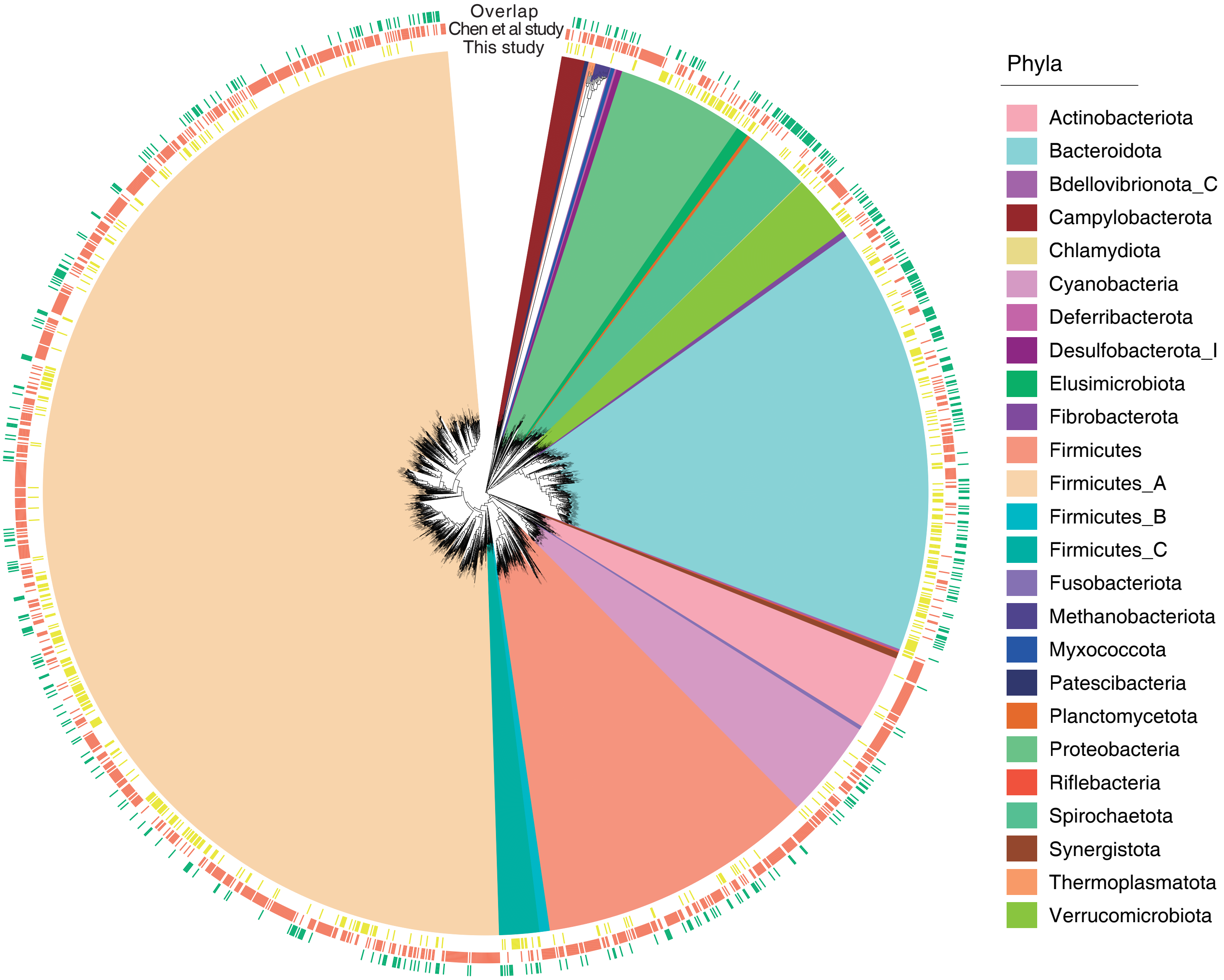

Supplementary Fig. 1. Phylogenetic tree of pig gut representative SGBs in this study and public dataset.
Inner circle is phylogenetic tree of 2,266 representative SGBs colored according to GTDB phylum-level taxonomic classifications (see color legend). Concentric rings moving outward from the first ring represent the prevalence/absence of SGBs detected by this study (inner ring) and the prevalence/absence of SGBs identified by Chen et al (2021) (medium ring), as well as the prevalence/absence of SGBs discovered from the two studies (outer ring), respectively.The related source data is provided as a source data file.

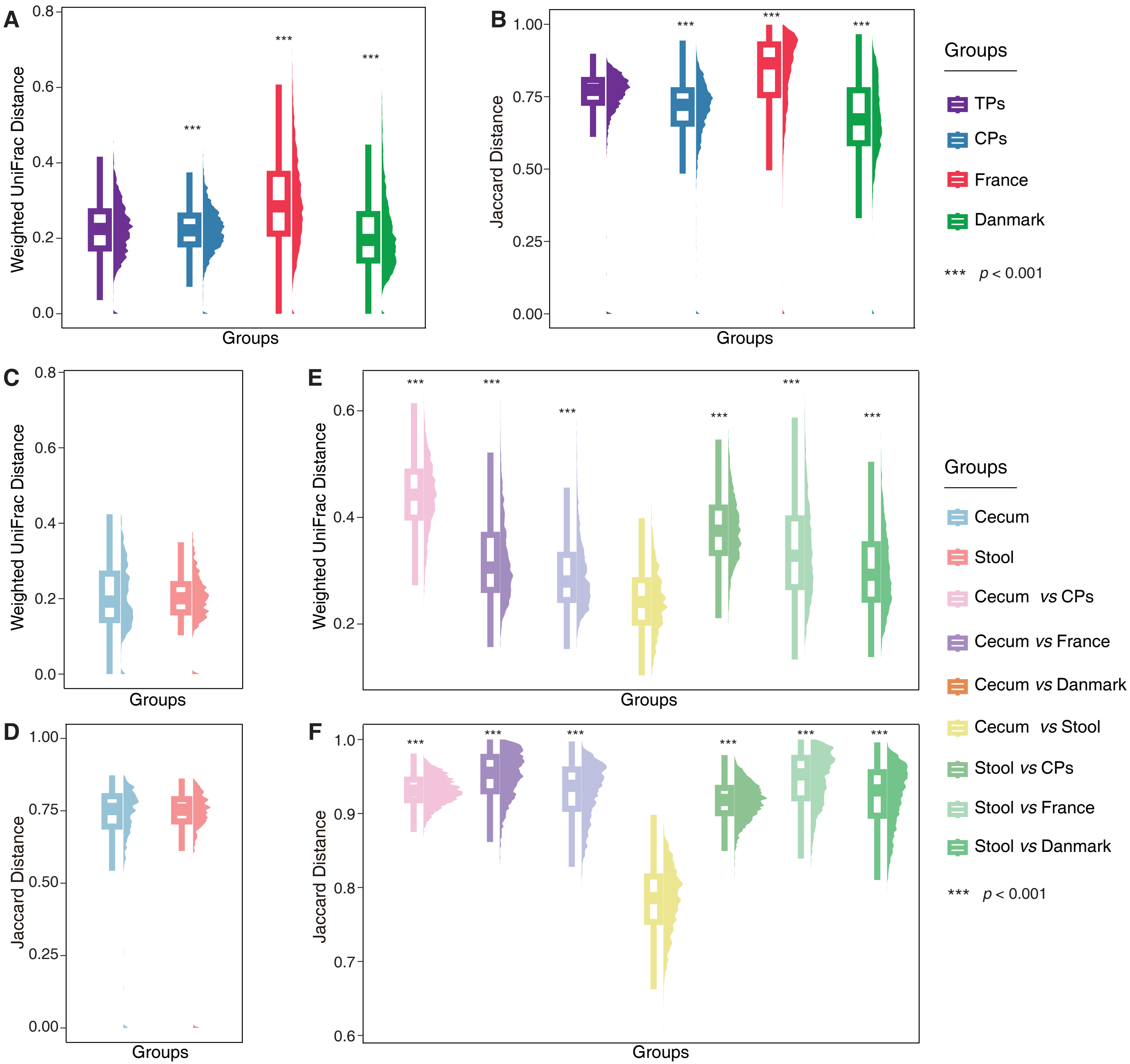

Supplementary Fig.2. Distance of beta diversity between host-groups or sample types
The violin plots and box plots represent Weighted UniFrac distance values and Jaccard distance values between host-groups or sample types. A and B represent the significant difference in Weighted UniFrac distance values and Jaccard distance values between TPs and other host-groups. C and D represent no significant differences (Permutational multivariate analysis of variance, $p<0.05$) in Weighted UniFrac distance values and Jaccard distance values between fecal and cecum samples. E and F represent the significant difference in Weighted UniFrac distance values and Jaccard distance values between sample types and host-groups. The related source data is provided as a source data file.

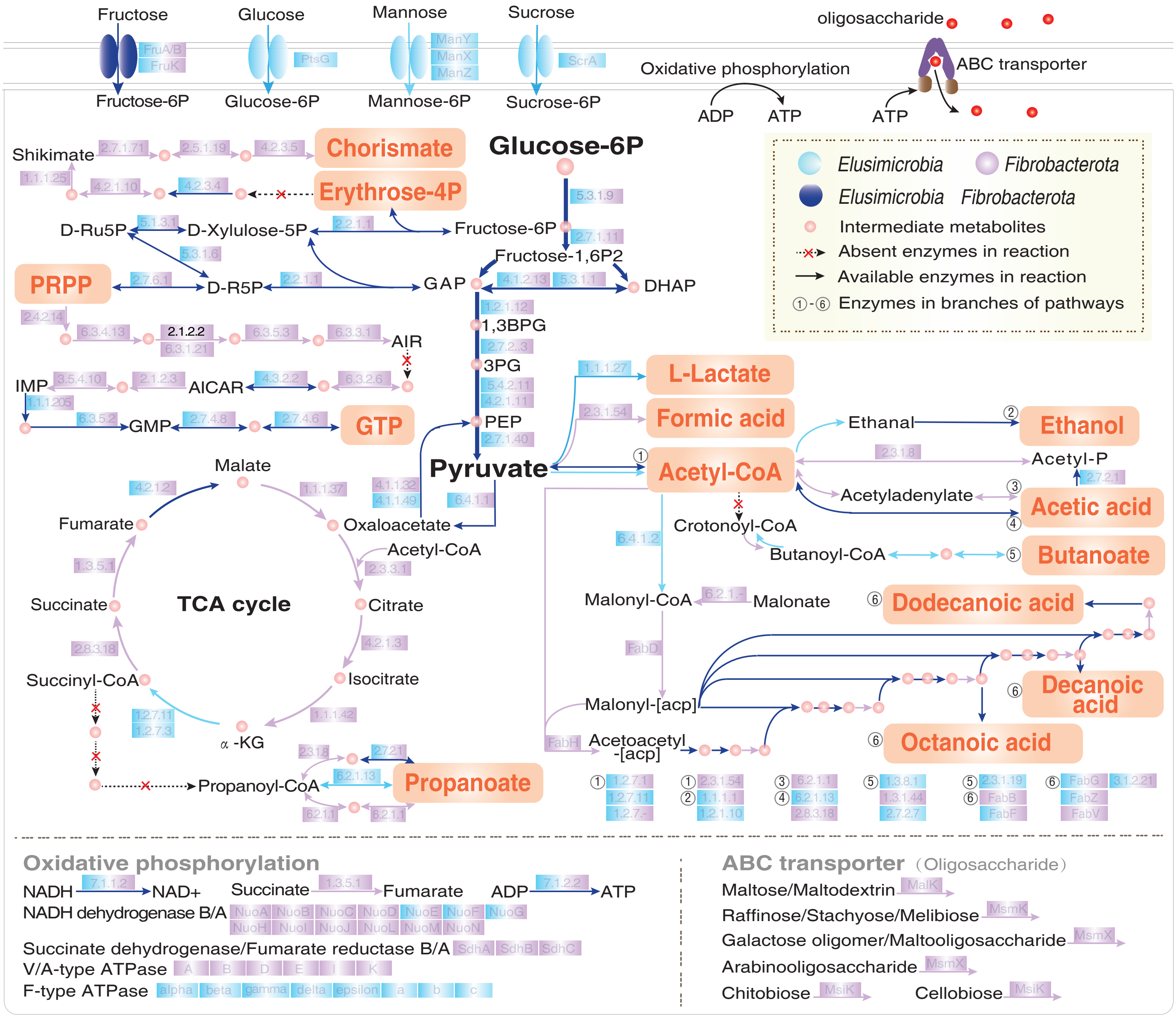

Supplementary Fig. 3. Synthesis of energy sources and key precursor metabolites of SGBs in TPs-associated bacteria.
TP-associated bacteria were involved in the energy producing, such as butyrate, acetic acid, propionate, ethanol and lactate, and some key precursor metabolites, including erythrose-4phosphate (erythrose-4P) and 5-phosphoribosyl diphosphate (PRPP). All enzymes and enzyme nomenclature (EC) numbers were obtained from KEGG database (Supplementary Table 5). GAP, glyceraldehyde 3-phosphate; D-R5P, d-ribose 5-phosphate; D-Ru5P, d-ribulose 5-phosphate; AIR, amino imidazole ribonucleotide; AICAR, 1-(5'-Phosphoribosyl)-5-amino-4-imidazolecarboxamide; IMP, inosine monophosphate; GMP, guanosine 5'-phosphate; GTP, guanosine 5'-triphosphate; 1,3BPG, glycerate 1,3-bisphosphate; 3PG, 3-phosphoglycerate; PEP, phosphoenolpyruvate; α-KG, alpha-ketoglutaric acid.

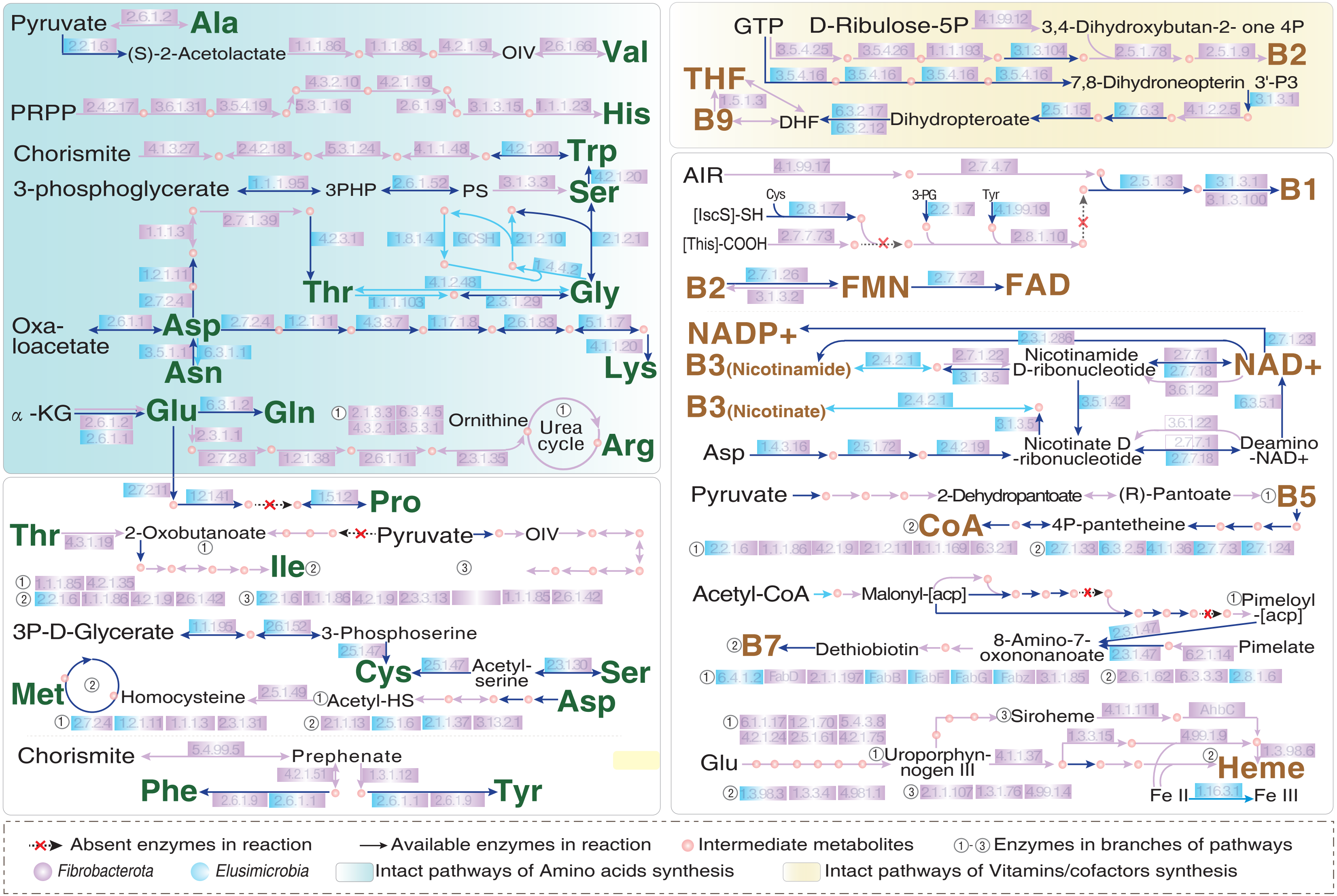

Supplementary Fig. 4. Synthesis of amino acids and B vitamins of SGBs in TPs-associated bacteria.

TP-associated bacteria were involved in the synthesis of 20 essential amino acids, and B vitamins and their active forms. All enzymes and EC numbers were referred to KEGG database (Supplementary Table 5). OIV, 2-oxoisovalerate; PRPP, 5-phosphoribosyl diphosphate; PS, 3-phosphoserine; 3PHP, 3-phosphohydroxypyruvate; α -KG, alpha-ketoglutaric acid; GTP, guanosine 5'-triphosphate; DHF, 7,8-Dihydrofolate; THF, tetrahydrofolate; AIR, amino imidazole ribonucleotide; 3PG, 3-phosphoglycerate; FAD, flavin adenine dinucleotide; FMN, riboflavin-5-phosphate; NAD+, nicotinamide adenine dinucleotide; NADP+, nicotinamide adenine dinucleotide phosphate.

Supplementary Table 1. Background information on Tibetan pig samples

Sample ID	Body site	Region
TP001	Stool	Deqen prefecture, Yunnan, China
TP001MC	Cecum content	Deqen prefecture, Yunnan, China
TP002	Stool	Deqen prefecture, Yunnan, China
TP003	Stool	Deqen prefecture, Yunnan, China
TP003MC	Cecum content	Deqen prefecture, Yunnan, China
TP004MC	Cecum content	Deqen prefecture, Yunnan, China
TP005	Stool	Deqen prefecture, Yunnan, China
TP005MC	Cecum content	Deqen prefecture, Yunnan, China
TP006MC	Cecum content	Deqen prefecture, Yunnan, China
TP007MC	Cecum content	Deqen prefecture, Yunnan, China
TP008	Stool	Deqen prefecture, Yunnan, China
TP008MC	Cecum content	Deqen prefecture, Yunnan, China
TP009	Stool	Deqen prefecture, Yunnan, China
TP010	Stool	Deqen prefecture, Yunnan, China
TP011	Stool	Deqen prefecture, Yunnan, China
TP012	Stool	Deqen prefecture, Yunnan, China
TP012MC	Cecum content	Deqen prefecture, Yunnan, China
TP014	Stool	Deqen prefecture, Yunnan, China
TP014MC	Cecum content	Deqen prefecture, Yunnan, China
TP015	Stool	Deqen prefecture, Yunnan, China
TP015MC	Cecum content	Deqen prefecture, Yunnan, China
TP017MC	Cecum content	Deqen prefecture, Yunnan, China
TP018	Stool	Deqen prefecture, Yunnan, China
TP018MC	Cecum content	Deqen prefecture, Yunnan, China
TP019MC	Cecum content	Deqen prefecture, Yunnan, China
TP020	Stool	Deqen prefecture, Yunnan, China
TP020MC	Cecum content	Deqen prefecture, Yunnan, China
TP021	Stool	Deqen prefecture, Yunnan, China
TP021MC	Cecum content	Deqen prefecture, Yunnan, China
TP022	Stool	Deqen prefecture, Yunnan, China
TP022MC	Cecum content	Deqen prefecture, Yunnan, China
TP023	Stool	Deqen prefecture, Yunnan, China
TP024	Stool	Deqen prefecture, Yunnan, China
TP024MC	Cecum content	Deqen prefecture, Yunnan, China
TP025	Stool	Deqen prefecture, Yunnan, China
TP025MC	Cecum content	Deqen prefecture, Yunnan, China
TP026	Stool	Deqen prefecture, Yunnan, China
TP027	Stool	Deqen prefecture, Yunnan, China
TP027MC	Cecum content	Deqen prefecture, Yunnan, China
TP028	Stool	Deqen prefecture, Yunnan, China
TP029	Stool	Deqen prefecture, Yunnan, China
TP029MC	Cecum content	Deqen prefecture, Yunnan, China
TP030MC	Cecum content	Deqen prefecture, Yunnan, China
TP031MC	Cecum content	Deqen prefecture, Yunnan, China
TP033	Stool	Deqen prefecture, Yunnan, China
TP034MC	Cecum content	Deqen prefecture, Yunnan, China
TP036	Stool	Deqen prefecture, Yunnan, China
TP036MC	Cecum content	Deqen prefecture, Yunnan, China
TP037MC	Cecum content	Deqen prefecture, Yunnan, China
TP040MC	Cecum content	Deqen prefecture, Yunnan, China
TP041	Stool	Deqen prefecture, Yunnan, China
TP041MC	Cecum content	Deqen prefecture, Yunnan, China
TP042	Stool	Deqen prefecture, Yunnan, China
TP043	Stool	Deqen prefecture, Yunnan, China
TP043MC	Cecum content	Deqen prefecture, Yunnan, China
TP044MC	Cecum content	Deqen prefecture, Yunnan, China
TP045	Stool	Deqen prefecture, Yunnan, China
TP047MC	Cecum content	Deqen prefecture, Yunnan, China
TP049MC	Cecum content	Deqen prefecture, Yunnan, China
TP051	Stool	Deqen prefecture, Yunnan, China
TP052MC	Cecum content	Deqen prefecture, Yunnan, China
TP053MC	Cecum content	Deqen prefecture, Yunnan, China
TP056	Stool	Deqen prefecture, Yunnan, China
TP058	Stool	Deqen prefecture, Yunnan, China
TP059MC	Cecum content	Deqen prefecture, Yunnan, China

Supplementary Table 2. Basic information of 1048 SGBs

SGBs ID	MAGs in SGBs (cluster number)	Completeness	Contamination	Length	N50	Gene number	Contig number
ERR1135178_bin.26	1	88.5	1.88	2257366	55331	2166	84
ERR1135178_bin.33	1	84.49	1.77	2678723	9172	2611	367
ERR1135178_bin.34	24	95.05	0.29	1584585	12932	1633	164
ERR1135178_bin.36	3	99.27	0.34	2502352	30766	2412	130
ERR1135178_bin.56	1	94.76	2.63	2753559	44474	2611	108
ERR1135178_bin.57	1	76.96	1.23	2149882	7664	2195	341
ERR1135178_bin.73	2	84.59	1.97	1705052	7749	1784	260
ERR1135178_bin.9	1	77.34	2.6	2106566	6870	2327	355
ERR1135179_bin.22	1	88.03	1.71	2107912	50805	2115	79
ERR1135179_bin.29	1	88.79	1.72	2813357	27345	2803	190
ERR1135180_bin.57	3	89.01	0	871682	200038	841	11
ERR1135181_bin.2	1	88	1.33	1680851	53338	1748	63
ERR1135181_bin.32	16	95.96	1.19	2731682	42634	2282	100
ERR1135181_bin.58	1	93.33	0	1658542	11186	2012	213
ERR1135181_bin.62	15	90.11	0	937271	70674	931	18
ERR1135181_bin.67	21	88.17	0	1739183	23647	1557	97
ERR1135181_bin.68	3	88.89	1.14	2070247	21839	2086	157
ERR1135182_bin.35	2	78.77	0.26	1748043	8007	1615	258
ERR1135182_bin.53	1	93.22	0	1786782	12425	1816	188
ERR1135183_bin.20	2	88.03	0.85	1629902	32634	1620	91
ERR1135183_bin.4	1	95.41	2.9	2932975	46669	2686	103
ERR1135183_bin.75	2	83.54	1.61	2049359	8593	2061	291
ERR1135184_bin.93	1	77.68	2.67	2753618	6841	2968	466
ERR1135185_bin.73	6	91.23	1.12	2604828	21432	2080	167
ERR1135187_bin.18	1	86.96	0.67	1666088	11998	1636	183
ERR1135187_bin.39	1	94.38	0	1428354	119860	1375	24
ERR1135187_bin.47	3	99.43	0.96	2786180	80335	2681	52
ERR1135187_bin.54	4	97.18	0.43	2746841	19165	2385	224
ERR1135187_bin.70	1	83.33	1.57	1536368	20187	1616	114
ERR1135187_bin.84	8	98.84	0	2909810	30364	2691	127
ERR1135187_bin.93	1	97.4	2.5	1558349	35923	1439	67
ERR1135188_bin.12	3	94.28	0.57	2489980	77790	1940	44
ERR1135188_bin.29	1	86.8	0.67	1586610	10817	1733	190
ERR1135188_bin.33	1	95.51	1.12	1597494	47358	1465	49
ERR1135188_bin.61	28	96.47	0.4	2570356	18365	2235	210
ERR1135188_bin.68	13	94.35	0	1947137	127292	1687	24
ERR1135188_bin.70	3	96.35	0	2576848	23230	2213	175
ERR1135189_bin.31	13	97.5	0.26	3116019	29896	2681	148
ERR1135189_bin.44	3	95.56	0.16	1407608	92198	1249	20
ERR1135189_bin.5	1	96.33	1.01	1816790	14880	1831	158
ERR1135189_bin.63	6	93.96	0.22	2449927	31919	2070	116
ERR1135189_bin.8	1	78.8	0.4	1033591	25584	950	67
ERR1135190_bin.104	2	97.32	0.34	2562126	80855	2539	45
ERR1135190_bin.27	11	97.41	0.19	2294365	61801	1854	52
ERR1135190_bin.32	1	95.35	1.12	1540898	75968	1332	34
ERR1135190_bin.34	1	89.04	0.69	1988426	8006	2071	298
ERR1135190_bin.4	18	98.66	0	2923597	77734	2901	89
ERR1135190_bin.41	3	96.26	1.97	2929311	139051	2634	44
ERR1135190_bin.63	9	89.04	0.67	1619412	34953	1723	81
ERR1135190_bin.64	7	92.17	0.67	1593438	33512	1560	73
ERR1135190_bin.88	1	93.12	1.68	2648601	20410	2503	208
ERR1135190_bin.91	66	96.28	0	1822853	63539	1601	40
ERR1135191_bin.13	93	93.63	0	2801324	29741	2455	141
ERR1135191_bin.2	2	96.66	0.67	3296480	17990	3379	288
ERR1135191_bin.22	2	79.25	1.89	2195170	17936	2183	175
ERR1135191_bin.23	21	96.85	0.37	2931501	31222	2451	140
ERR1135191_bin.26	1	90.38	0.23	2188630	12240	2371	241
ERR1135191_bin.33	2	97.38	0	1375872	32698	1220	63
ERR1135191_bin.44	4	95.34	2.45	3032793	26375	2812	204
ERR1135191_bin.69	6	92.88	0	1279731	173831	1176	19
ERR1135191_bin.71	8	92.62	0.16	2508788	62273	2056	67
ERR1135192_bin.10	18	87.6	0	2204081	24618	2205	124
ERR1135192_bin.11	1	98.49	0	1782147	17404	1693	144
ERR1135192_bin.18	9	97.58	3.6	2820212	85724	2730	53
ERR1135192_bin.2	2	83.26	2.24	2093406	13104	2117	212
ERR1135192_bin.41	1	88.85	0.99	2173827	10847	1963	255
ERR1135192_bin.68	1	79.59	4.41	2559855	14139	2531	260
ERR1135192_bin.7	3	93.32	1.24	2270190	22663	1904	128
ERR1135192_bin.82	1	95.74	0.71	2480647	28854	2516	124
ERR1135192_bin.89	1	77.66	2.04	968453	14425	939	88
ERR1135192_bin.90	1	93.15	0	1352538	113869	1219	15
ERR1135192_bin.94	6	85.12	0	1595088	29012	1369	83
ERR1135193_bin.102	1	95.69	0.29	1993887	17152	1845	147
ERR1135193_bin.27	1	87.35	0.67	1776482	15237	1757	155
ERR1135193_bin.58	1	90.67	1.33	1073489	47829	1119	47
ERR1135193_bin.97	52	98.39	0	1707438	38744	1739	60
ERR1135194_bin.3	3	90.07	1.01	1931919	37839	1900	83
ERR1135194_bin.34	2	89.24	0	1846600	11484	1935	215
ERR1135194_bin.39	1	99.11	0	1825562	54025	1735	53
ERR1135194_bin.48	59	96.99	0.37	3137673	78890	2251	67
ERR1135194_bin.5	2	90.45	0	832162	24491	784	53
ERR1135194_bin.51	2	82.54	2.27	2300350	10610	2361	260
ERR1135194_bin.53	1	93.71	0.94	2227305	15109	2318	205
ERR1135194_bin.56	2	96.81	0	2084550	26019	2082	116
ERR1135194_bin.68	187	96.42	0.13	2676095	27241	2242	157
ERR1135195_bin.12	3	94.01	0	1414616	52860	1364	39

SGBs ID	MAGs in SGBs (cluster number)	Completeness	Contamination	Length	N50	Gene number	Contig number
ERR1135195_bin.18	1	92.72	5.78	3593999	21325	2990	263
ERR1135195_bin.19	1	77.69	0.81	1063010	35311	970	43
ERR1135195_bin.36	4	90.73	0	1648050	49498	1289	61
ERR1135195_bin.54	2	85.95	0.02	1634400	15860	1493	135
ERR1135195_bin.55	1	94.79	0.37	2795664	34337	2248	114
ERR1135195_bin.61	13	83.21	0	2122936	10195	2246	283
ERR1135195_bin.69	126	95	0.48	2126006	33520	1759	93
ERR1135196_bin.11	97	99.66	1.31	2193751	41590	2076	77
ERR1135196_bin.23	1	77.48	1.68	1433246	9373	1533	174
ERR1135196_bin.24	2	97.91	1.37	2590016	78668	2386	45
ERR1135196_bin.29	1	88.95	2.01	1773297	12085	1815	185
ERR1135196_bin.4	1	96.64	0.67	1925396	41289	1831	70
ERR1135196_bin.50	2	91.71	0.36	1926388	9093	2067	246
ERR1135196_bin.51	8	89.74	0.85	1968378	126542	1914	25
ERR1135196_bin.55	1	77.18	0.55	1841362	12788	1891	203
ERR1135196_bin.68	2	77.55	0.38	1966121	65468	1671	47
ERR1135197_bin.2	1	90.75	1.12	1148188	18915	1210	89
ERR1135197_bin.22	54	95.4	0	2311544	37753	1883	86
ERR1135197_bin.34	1	98.55	0.82	2473076	117321	2390	41
ERR1135197_bin.36	5	95.97	0	1785086	78291	1654	38
ERR1135197_bin.41	1	88.93	2.97	1957816	12172	1673	195
ERR1135197_bin.53	73	96.56	0.74	2196133	21729	2115	135
ERR1135197_bin.71	2	88.26	0.17	1760044	7741	1781	280
ERR1135197_bin.78	5	93.1	0	2094915	22013	1866	151
ERR1135198_bin.11	7	98.37	1.33	2812521	43261	2359	114
ERR1135198_bin.37	76	97.2	0.7	2145028	39463	1945	77
ERR1135198_bin.40	1	83.02	1.89	2511960	14145	2519	241
ERR1135198_bin.54	2	99.44	1.12	1843300	64110	1829	56
ERR1135198_bin.73	9	97.36	0	2394085	44167	1974	78
ERR1135198_bin.75	2	92.13	1.12	1177806	99372	932	27
ERR1135198_bin.77	2	78.36	0.19	1753064	14606	1485	146
ERR1135199_bin.23	3	84.79	0.22	1428675	15626	1319	122
ERR1135199_bin.3	1	90.73	0	1056407	21950	1121	74
ERR1135199_bin.45	2	94	0	1706721	21707	1780	104
ERR1135199_bin.55	1	93.82	3.37	1172387	37640	1294	60
ERR1135199_bin.61	22	97.06	1.61	1910779	35859	1790	73
ERR1135199_bin.64	5	97.09	0	2118147	51440	1925	58
ERR1135199_bin.71	4	95.6	0.48	2283095	86484	1867	44
ERR1135199_bin.72	1	92.17	4.55	3069483	17765	2986	262
ERR1135200_bin.105	1	86.16	1.03	1899368	6649	2044	315
ERR1135200_bin.11	1	92.13	0.36	1609476	140590	1506	27
ERR1135200_bin.12	4	88.7	0.77	2283865	14079	1944	216
ERR1135200_bin.22	1	94.46	1.34	2118205	20968	2194	148
ERR1135200_bin.44	3	92.6	2.35	2931169	29390	2458	160
ERR1135200_bin.65	162	94.61	0.48	2104098	87676	1934	35
ERR1135200_bin.73	1	91.83	0.34	2291034	15523	2266	193
ERR1135200_bin.85	9	97.99	1.23	3114644	23756	2843	209
ERR1135200_bin.9	1	87.18	1.8	1744038	19110	1782	126
ERR1135201_bin.12	3	86.25	1.13	2076313	21023	1716	131
ERR1135201_bin.17	2	75.53	0.67	1997774	10318	1821	240
ERR1135201_bin.34	1	89.98	0.67	2111095	15411	2029	193
ERR1135201_bin.47	2	85.43	1.14	1904776	8348	1955	286
ERR1135201_bin.67	1	82.05	0	1892035	27009	1863	96
ERR1135201_bin.70	1	91.13	0	1515097	21703	1445	103
ERR1135201_bin.79	1	79.65	2.24	1654429	6987	1700	280
ERR1135201_bin.83	57	95.48	0	1791389	38429	1582	70
ERR1135201_bin.84	1	86.8	0.67	2066172	22339	2173	146
ERR1135201_bin.86	1	84.27	0	870814	7604	1006	127
ERR1135201_bin.99	1	84.44	0.04	1377845	11131	1349	170
ERR1135202_bin.1	6	97.72	0.34	2306788	33752	2183	98
ERR1135202_bin.10	8	92.62	0	2187302	49995	1797	64
ERR1135202_bin.14	1	92.62	0	1531121	48795	1494	45
ERR1135202_bin.31	1	90.51	1.01	1883633	11096	1975	210
ERR1135202_bin.56	1	78.46	0.6	1655077	7202	1676	268
ERR1135202_bin.68	1	80.31	0	1317904	63786	1144	28
ERR1135202_bin.70	51	94.03	0.53	2346688	15670	2161	202
ERR1135203_bin.24	35	94.76	0	2185873	38332	1981	85
ERR1135203_bin.44	2	95.24	3.49	2274154	38838	1947	91
ERR1135204_bin.18	3	79.19	1.2	1519103	9079	1579	205
ERR1135204_bin.75	1	79.17	3	2353732	45985	2197	116
ERR1135204_bin.76	3	85	0	1951064	38565	1672	77
ERR1135205_bin.17	1	94.08	0.67	1905622	15981	1977	146
ERR1135205_bin.43	9	88.08	0	2585658	8343	2435	356
ERR1135205_bin.45	2	91.98	1.64	2780270	22592	2367	201
ERR1135205_bin.52	1	75.45	3.18	2002461	6045	1930	367
ERR1135205_bin.60	5	93.57	0	2314679	72774	1897	56
ERR1135205_bin.8	1	94.31	1.6	1963962	16074	1939	165
ERR1135206_bin.63	3	93.16	0.89	1397340	22593	1337	85
ERR1135206_bin.69	4	82.87	0	1389446	18181	1164	112
ERR1135207_bin.59	1	77.99	0.54	1123216	6171	1248	205
ERR1135208_bin.18	2	91.96	1.4	2985488	11809	2852	330
ERR1135208_bin.20	1	95.6	0.67	2607188	20043	2554	177
ERR1135208_bin.40	1	76.19	0.81	1276400	5894	1387	226
ERR1135208_bin.43	3	86.03	0	1597808	11729	1637	181
ERR1135208_bin.72	12	92.99	1.11	1756788	38144	1544	88
ERR1135209_bin.24	1	88.26	0.67	2199383	9341	2224	284
ERR1135209_bin.28	57	90.94	0	1604098	73603	1585	37
ERR1135209_bin.29	5	87.05	0.93	2043341	22113	1789	116

SGBs ID	MAGs in SGBs (cluster number)	Completeness	Contamination	Length	N50	Gene number	Contig number
ERR1135209_bin.42	37	96.77	0.93	2759433	27829	2164	148
ERR1135209_bin.8	2	88.81	0.02	1653666	78073	1440	32
ERR1135210_bin.33	13	92.51	0	1488691	32193	1349	78
ERR1135210_bin.54	6	82.49	1.45	1965807	25525	1902	124
ERR1135211_bin.21	14	87.65	0	1792285	9899	1886	224
ERR1135211_bin.33	4	97.47	1.44	2945472	121069	2795	65
ERR1135212_bin.14	3	87.81	0.69	1878410	12486	1822	200
ERR1135212_bin.26	2	78.11	0.84	2562843	6159	2576	458
ERR1135212_bin.6	3	91.13	1.25	1923744	28161	1645	87
ERR1135212_bin.70	1	87.58	2.01	2088260	8449	2257	289
ERR1135212_bin.78	6	93.03	0.72	1474122	27072	1331	85
ERR1135213_bin.14	1	77.36	0.43	1488033	5288	1770	309
ERR1135213_bin.44	8	92.33	0.56	2450940	21644	1983	163
ERR1135213_bin.46	1	91.72	1.34	2333592	26403	2254	132
ERR1135213_bin.84	11	97.99	0.38	2361627	46423	1981	66
ERR1135213_bin.9	2	94.44	2.84	2612940	42643	2517	119
ERR1135213_bin.96	3	91.42	0.13	2471434	20063	2153	161
ERR1135214_bin.3	1	75.08	0	1669438	6819	1757	273
ERR1135214_bin.8	1	76.78	0.57	1570700	18561	1590	120
ERR1135215_bin.21	3	95	2.42	2090517	21701	2134	147
ERR1135215_bin.23	1	80.31	0.89	1647329	11162	1799	201
ERR1135215_bin.52	5	98.18	0.26	2611811	50559	2062	86
ERR1135216_bin.19	24	96.88	1.06	2307515	29182	1928	116
ERR1135216_bin.4	1	85.23	2.01	1748441	17623	1794	135
ERR1135216_bin.5	1	89.89	1.12	1510476	19222	1808	111
ERR1135216_bin.67	29	97.27	0	1680285	63588	1450	51
ERR1135217_bin.14	5	94.13	0.48	2367876	48871	1906	74
ERR1135217_bin.5	8	86.52	0	1753163	45070	1678	66
ERR1135217_bin.57	100	97.4	0.37	2531892	46791	2020	82
ERR1135217_bin.65	2	85.47	0.85	2041537	21105	2183	146
ERR1135218_bin.14	2	91.45	1.68	2544796	15643	2524	232
ERR1135218_bin.58	4	88.89	2.42	2058320	54290	2070	71
ERR1135219_bin.28	2	84.21	0.71	1433954	10256	1374	173
ERR1135219_bin.32	1	87.47	0.67	1775884	34680	1718	91
ERR1135219_bin.41	5	91.67	0.29	1751561	9349	1871	227
ERR1135219_bin.51	1	84.28	0	1125799	6520	1167	194
ERR1135219_bin.61	1	86.23	0.38	1913354	9772	1707	245
ERR1135219_bin.8	1	99.88	0.37	1465574	30090	1516	74
ERR1135220_bin.1	6	98.78	0	1077283	355043	904	4
ERR1135220_bin.65	2	96.55	1.34	2712530	60924	2694	71
ERR1135220_bin.68	7	98.59	0.12	1585468	81072	1632	35
ERR1135220_bin.70	26	92.48	0	1197619	11419	1282	123
ERR1135221_bin.12	1	75.17	3.45	2820228	40291	2335	117
ERR1135221_bin.24	1	82.31	2.53	1750315	8496	1850	256
ERR1135221_bin.29	2	78.41	0.79	2501031	28461	2105	134
ERR1135222_bin.9	21	96.2	0	2664495	50756	2078	101
ERR1135223_bin.20	1	91.29	1.75	1782659	11612	1856	197
ERR1135223_bin.22	2	83.84	3.08	2539697	9538	2219	334
ERR1135223_bin.52	9	94.29	0	1826745	27873	1714	93
ERR1135224_bin.55	1	75.29	1.4	1162293	25730	1198	74
ERR1135224_bin.9	1	87.33	4.03	1729706	9311	1848	227
ERR1135225_bin.13	2	76.08	0.79	1698951	9709	1804	221
ERR1135225_bin.24	8	95.16	0	2204664	50763	2021	83
ERR1135226_bin.23	1	87.87	0.13	1540443	14342	1640	148
ERR1135227_bin.18	3	89.15	0.78	2583145	12040	2675	269
ERR1135227_bin.55	1	92.11	1.68	1978910	11085	1929	230
ERR1135227_bin.69	7	91.72	0.37	2364227	14759	1883	225
ERR1135229_bin.55	1	94.38	1.12	1181184	23435	1026	74
ERR1135230_bin.14	1	95.97	1.51	2979173	14781	3090	296
ERR1135231_bin.4	6	90.06	0.67	1738209	22065	2022	117
ERR1135232_bin.13	12	94.54	0.56	2677513	18445	2247	215
ERR1135232_bin.36	12	94	0.25	2588664	17625	2189	202
ERR1135233_bin.32	2	94.27	3.14	2899920	28155	2717	159
ERR1135233_bin.45	1	90.95	1.72	2179170	9429	2193	291
ERR1135235_bin.13	1	95.04	1.27	3174966	44388	3013	108
ERR1135235_bin.60	2	84.9	0	2895284	66649	2745	96
ERR1135239_bin.54	1	98.32	0.34	2873231	29338	2703	161
ERR1135241_bin.22	1	95.3	1.01	1862777	25822	1809	103
ERR1135241_bin.24	1	95.24	1.27	1972550	14118	1991	190
ERR1135241_bin.6	1	94.99	0.69	2955525	17681	2784	237
ERR1135241_bin.71	4	94.57	0.48	1813453	81857	1626	31
ERR1135242_bin.36	27	86.91	0.76	2195478	10686	1990	252
ERR1135245_bin.26	1	91.94	0	1575106	21396	1403	124
ERR1135245_bin.30	1	92.5	1.4	2032279	11364	2030	233
ERR1135245_bin.31	20	100	0	2221324	32768	2163	115
ERR1135246_bin.1	1	85.7	1.35	2281555	28678	1957	116
ERR1135246_bin.54	1	95.38	2.2	2335107	21631	2319	163
ERR1135247_bin.31	12	98.79	0.29	2356059	64069	2089	66
ERR1135247_bin.55	1	84.35	0	1360498	56392	1363	38
ERR1135248_bin.59	1	85.46	1.48	1612981	11934	1758	182
ERR1135250_bin.5	29	94.64	1.6	2850677	19805	2524	227
ERR1135251_bin.5	12	98.39	1.08	2105251	54672	1806	72
ERR1135252_bin.17	2	83.66	0.92	2404585	9583	2256	300
ERR1135252_bin.27	10	92.2	0	1954620	47351	1636	67
ERR1135252_bin.29	3	98.66	0	2166204	53032	2029	67
ERR1135252_bin.35	4	95.18	0	1639161	26491	1666	98
ERR1135252_bin.62	23	96.5	0	1601960	59061	1527	55
ERR1135253_bin.12	1	93.01	0	1799607	59075	1576	49

SGBs ID	MAGs in SGBs (cluster number)	Completeness	Contamination	Length	N50	Gene number	Contig number
ERR1135253_bin.21	14	97.9	1.05	2111643	85175	1819	59
ERR1135254_bin.35	13	96.53	0.06	1648322	22046	1629	98
ERR1135254_bin.37	32	87.17	0	1878216	14817	1932	182
ERR1135254_bin.65	6	94.38	0.11	1635110	19207	1734	132
ERR1135256_bin.12	41	98.83	0.74	2889976	56727	2378	111
ERR1135256_bin.53	2	85.57	0.32	1654770	7160	1798	269
ERR1135256_bin.60	5	95.51	1.12	1298434	12846	1320	139
ERR1135257_bin.51	1	97.32	0.67	2405203	19878	2392	165
ERR1135257_bin.52	7	99.61	0.59	2067957	25544	1906	125
ERR1135258_bin.21	2	91.72	0	2288657	39518	2067	105
ERR1135259_bin.12	195	94.43	0.37	2518739	21776	1998	167
ERR1135259_bin.56	1	83.92	0	1882416	28554	1889	101
ERR1135260_bin.22	62	94.63	0.2	2596734	26643	2044	157
ERR1135260_bin.40	10	88.32	0.16	1755429	15372	1614	172
ERR1135260_bin.45	1	88.49	0.9	1854525	20559	1641	127
ERR1135260_bin.46	13	97.36	1.64	2993572	36859	2458	117
ERR1135261_bin.34	32	92.04	0.56	2655020	45064	2102	82
ERR1135262_bin.1	1	76.35	0.02	1563275	6479	1610	268
ERR1135262_bin.42	16	90.94	0.19	2531032	31306	1991	130
ERR1135262_bin.43	1	98.31	0.85	2441305	50728	2318	121
ERR1135262_bin.62	1	89.74	0	1882563	49285	1786	79
ERR1135263_bin.3	15	97.26	0.12	2384241	30067	1940	113
ERR1135263_bin.42	2	92.62	0.67	1907623	24268	1711	121
ERR1135264_bin.17	2	89.03	0.54	1990235	19618	1622	135
ERR1135264_bin.18	1	92.92	2.58	2328198	17818	2311	181
ERR1135264_bin.32	3	88.64	1.25	1920406	8247	2049	267
ERR1135265_bin.18	4	95.12	1.94	2039056	20390	1920	144
ERR1135265_bin.33	4	92.04	0.95	2064369	21814	1989	149
ERR1135265_bin.44	2	93.45	3.49	2763572	43278	2574	127
ERR1135266_bin.18	2	94.44	0	1754050	29636	1786	77
ERR1135266_bin.21	2	89.34	0.46	2161431	23061	1718	141
ERR1135266_bin.58	79	96.27	0.49	2714450	23624	2237	177
ERR1135266_bin.67	1	93.33	1.67	1632710	30722	1664	70
ERR1135267_bin.18	1	89.93	2.58	3311767	19052	3236	295
ERR1135267_bin.21	10	91.13	0.54	1943676	29358	1516	88
ERR1135267_bin.30	25	97.08	0.65	2339567	36616	1959	93
ERR1135267_bin.37	104	92.49	0.37	2574634	18567	2040	199
ERR1135268_bin.39	2	91.73	0	2631030	33085	2293	128
ERR1135269_bin.18	71	95.76	0.34	1905902	14826	1920	171
ERR1135269_bin.35	2	75.44	0.56	1593050	22605	1903	111
ERR1135270_bin.3	8	96.05	2.65	2949133	61259	2913	108
ERR1135270_bin.77	3	95.57	0.63	2581046	54089	2349	84
ERR1135272_bin.36	1	93.99	1.01	2552698	17072	2538	212
ERR1135272_bin.44	1	81.4	0.22	1856512	7617	1912	289
ERR1135272_bin.51	6	91.99	1.05	2905199	26430	2438	174
ERR1135273_bin.2	1	92.95	0.67	2709283	25488	2539	149
ERR1135273_bin.20	14	96.49	0.47	2483936	42714	2470	96
ERR1135273_bin.35	1	83.2	1.9	1831168	8334	1660	261
ERR1135273_bin.37	1	86.37	0.59	2045812	22889	1896	141
ERR1135273_bin.39	3	89.74	0	1951892	55416	1872	62
ERR1135273_bin.51	2	98.1	0.32	2414494	51466	2400	65
ERR1135273_bin.8	2	76.82	0	2028224	15487	1826	173
ERR1135273_bin.81	1	94.51	1.27	2055732	9731	2109	270
ERR1135274_bin.6	1	89.67	0.95	2115021	77958	2078	49
ERR1135274_bin.61	15	96.69	1.9	2443042	16515	2406	218
ERR1135274_bin.69	2	89.51	0	1688775	32908	1532	79
ERR1135275_bin.2	2	98.66	0	2648995	107315	2319	33
ERR1135275_bin.23	1	77.68	0.48	1356995	9467	1328	166
ERR1135275_bin.38	2	95.97	1.57	2250284	146783	2155	34
ERR1135275_bin.52	2	95.2	3.9	2421464	20630	2418	169
ERR1135275_bin.62	6	87.78	0.37	2297351	33555	1914	101
ERR1135276_bin.43	24	89.52	0.62	2450477	14203	2065	215
ERR1135276_bin.56	1	86.74	0	1975794	14818	2037	179
ERR1135276_bin.61	9	95.82	1.75	2238508	17931	2243	168
ERR1135276_bin.72	1	90.24	3.17	2004457	17515	2018	153
ERR1135276_bin.96	1	86.94	1.23	2537321	11719	2357	274
ERR1135277_bin.10	1	75.81	0	1937349	15652	1677	170
ERR1135277_bin.11	1	90.45	1.12	1385245	23395	1507	88
ERR1135277_bin.29	1	91.41	2.5	2306885	25531	2172	124
ERR1135277_bin.54	1	94.97	0.97	2562259	24245	2486	162
ERR1135278_bin.11	2	99.28	0	1773773	76837	1642	29
ERR1135278_bin.32	9	93.45	0	1389582	28403	1242	65
ERR1135278_bin.69	15	90.14	0	1684748	14354	1532	169
ERR1135278_bin.75	4	92.56	0.97	2138816	11173	2081	247
ERR1135279_bin.11	8	94.38	0.87	1795985	9322	2100	231
ERR1135280_bin.46	2	85.68	0	1482754	13300	1587	151
ERR1135281_bin.21	1	91.85	3.37	1271702	33145	1311	68
ERR1135283_bin.47	22	95.45	0	1511129	18978	1507	117
ERR1135284_bin.14	1	86.32	0.85	1708274	25379	1662	102
ERR1135284_bin.23	3	87.36	0.13	2039096	29839	1775	99
ERR1135284_bin.38	1	88.6	4.54	2618178	11650	2783	295
ERR1135285_bin.14	8	99.36	0	2566071	47815	2467	112
ERR1135285_bin.74	50	99.3	0	2206068	70587	1968	47
ERR1135286_bin.7	67	97.85	0	2335217	84017	1996	44
ERR1135286_bin.84	3	95.02	1.44	3071256	45052	2818	114
ERR1135288_bin.11	3	94.92	1.63	2131199	16417	2110	190
ERR1135288_bin.89	1	80.53	4	2806314	5211	3182	574
ERR1135288_bin.96	5	92.39	0.05	2047642	23155	1913	132

SGBs ID	MAGs in SGBs (cluster number)	Completeness	Contamination	Length	N50	Gene number	Contig number
ERR1135289_bin.22	7	99.3	0	2549276	47029	2333	93
ERR1135289_bin.33	17	96.34	0.81	1738156	22073	1673	119
ERR1135289_bin.44	25	91.91	0	2597546	21519	2036	169
ERR1135290_bin.10	1	88.03	1.28	1930148	26680	1906	113
ERR1135290_bin.47	1	90.45	2.97	2116011	23832	2555	173
ERR1135291_bin.45	4	82.93	0.19	2118159	33880	1844	110
ERR1135291_bin.83	51	94.13	1.51	2981649	25005	2496	165
ERR1135291_bin.88	39	93.1	0	2078521	16814	1694	173
ERR1135292_bin.53	8	95.73	1.11	2774588	24892	2606	186
ERR1135292_bin.87	1	80.09	1.22	1186065	5990	1368	214
ERR1135293_bin.33	1	87.72	0.58	2087175	8105	2166	310
ERR1135293_bin.44	2	75.4	0.36	1263569	9032	1197	175
ERR1135293_bin.50	5	87.51	0.83	2508793	16178	2091	224
ERR1135294_bin.23	1	83.15	0.11	2040792	14339	1702	197
ERR1135294_bin.5	1	95.89	0.67	1811775	15280	1824	151
ERR1135294_bin.61	4	94.93	0.37	2211843	23412	1975	122
ERR1135295_bin.34	4	97.33	0.11	1856185	51654	1909	89
ERR1135295_bin.8	1	85.76	2.3	2223960	10509	2280	279
ERR1135297_bin.27	3	92.07	0	2313928	14614	1750	210
ERR1135297_bin.46	2	97.85	0	1447846	93668	1432	21
ERR1135299_bin.83	15	95.51	0	1180195	20037	1127	95
ERR1135300_bin.25	12	96.79	0	1998622	25215	1979	122
ERR1135301_bin.23	3	98.5	0.63	2079209	19468	2024	142
ERR1135302_bin.36	1	93.98	3.45	2299294	15932	2342	216
ERR1135304_bin.16	1	89.86	0	1418324	11302	1397	166
ERR1135304_bin.69	3	87.47	1.34	1402910	11103	1447	158
ERR1135306_bin.47	1	81.25	0	1767755	48430	1513	61
ERR1135307_bin.30	2	96.04	3.61	2851806	16496	2941	266
ERR1135308_bin.44	3	94.54	0.65	2063368	22711	1943	146
ERR1135308_bin.99	3	88.05	2.24	2658504	14136	2739	304
ERR1135309_bin.22	11	99.33	0	2004665	34322	1884	83
ERR1135311_bin.31	1	97.7	2.01	2826734	39785	2757	120
ERR1135311_bin.79	12	95.86	0	1525123	18583	1557	107
ERR1135311_bin.91	2	95.77	1.54	775724	26525	1361	46
ERR1135311_bin.94	2	95.86	1.13	2878466	26561	2850	160
ERR1135312_bin.41	13	94.39	1.94	3185817	23625	2506	227
ERR1135315_bin.55	3	96.7	1.77	1759075	17438	1864	136
ERR1135316_bin.81	70	94.55	0.19	2408185	29436	1981	134
ERR1135317_bin.24	28	98.6	0	2548497	48236	2410	110
ERR1135318_bin.25	98	98.39	0	2047854	48271	1827	57
ERR1135318_bin.39	1	85.33	1.33	1103232	37519	1178	54
ERR1135318_bin.98	1	83.08	0.42	2545741	11826	2578	291
ERR1135319_bin.2	3	89.32	0.13	1642620	22586	1610	97
ERR1135319_bin.20	35	85.57	0.83	1618392	62851	1607	43
ERR1135319_bin.63	1	80.67	0	865228	7018	996	146
ERR1135319_bin.78	1	88	1.33	1504837	36850	1578	84
ERR1135320_bin.26	1	91.76	1.23	2140701	24401	1949	128
ERR1135322_bin.22	2	87.58	1.63	1842944	7480	1950	291
ERR1135322_bin.29	1	97.68	0.16	2529557	62360	2357	73
ERR1135322_bin.55	1	78.14	4.15	1942850	7183	2105	322
ERR1135324_bin.51	9	96.64	1.13	2633838	19446	2142	206
ERR1135325_bin.13	2	97.32	2.91	4718082	30739	4316	299
ERR1135325_bin.58	1	97.97	3.47	3092596	68911	2919	112
ERR1135327_bin.40	1	95.19	0.67	2134307	88453	2014	45
ERR1135327_bin.92	1	91.15	1.08	1958617	18513	1661	153
ERR1135328_bin.18	2	94.34	1.01	2004047	16683	1980	163
ERR1135328_bin.24	9	89.93	1.34	1449333	13135	1527	145
ERR1135328_bin.36	92	96.61	0.34	2743514	34654	2120	131
ERR1135328_bin.48	3	75.3	0.78	1781612	14415	1830	162
ERR1135328_bin.73	23	98.66	1.34	2491913	44370	2366	97
ERR1135330_bin.69	31	97.95	0.48	2712652	67402	2599	81
ERR1135333_bin.56	2	85.47	2.56	1858828	25215	1909	118
ERR1135334_bin.33	25	94.93	0	1866060	37454	1665	71
ERR1135335_bin.32	34	99.45	0	1597880	31745	1576	85
ERR1135336_bin.80	1	83.94	4.45	2475880	7501	2644	369
ERR1135337_bin.78	3	94.57	1.58	3182246	11626	3297	379
ERR1135341_bin.51	1	80.86	2.99	1618542	8762	1731	233
ERR1135341_bin.54	59	92.88	0.06	1757998	8539	1595	246
ERR1135341_bin.9	69	94.76	0	1960683	28211	1640	108
ERR1135342_bin.51	30	95.21	0.6	1808721	13604	1767	183
ERR1135342_bin.65	8	97.9	0	2994301	75237	2853	97
ERR1135346_bin.81	3	88.84	0	1884253	9846	2020	248
ERR1135347_bin.12	23	97.55	0	2404787	55647	2192	102
ERR1135347_bin.19	2	94.41	0	2671313	105656	2530	47
ERR1135348_bin.2	18	96.15	0	2129523	10230	1966	256
ERR1135348_bin.33	1	91.4	0	1317427	32231	1338	56
ERR1135348_bin.59	38	98.66	0	2407369	71268	2221	47
ERR1135348_bin.60	37	94.13	0	2311384	51315	1858	70
ERR1135350_bin.18	1	79.78	3.37	1576245	13816	1836	165
ERR1135350_bin.23	58	96.01	0.83	1302815	12067	1318	141
ERR1135350_bin.27	14	98.6	0.7	2419792	76951	2194	61
ERR1135351_bin.34	1	78.36	0.54	1126930	6075	1271	207
ERR1135351_bin.45	8	94.41	0.47	2779270	41533	2234	123
ERR1135351_bin.65	2	95.33	2.04	2948699	33835	2880	139
ERR1135352_bin.30	37	90.06	0.67	1324734	18182	1356	104
ERR1135353_bin.10	2	86.13	1.06	1845767	11369	1801	208
ERR1135356_bin.12	1	89.26	2.57	1985660	10935	2067	228
ERR1135356_bin.72	13	96.1	0	2220815	77066	1820	41

SGBs ID	MAGs in SGBs (cluster number)	Completeness	Contamination	Length	N50	Gene number	Contig number
ERR1135357_bin.28	1	89.42	0.56	2177683	22392	1762	135
ERR1135359_bin.27	1	79.94	1.2	1162556	6179	1303	212
ERR1135360_bin.30	5	93.96	0.67	1912812	20809	1941	134
ERR1135360_bin.47	2	89.4	1.9	2547856	10248	2632	316
ERR1135362_bin.43	1	75.53	0.97	1280819	6414	1452	223
ERR1135362_bin.58	27	96.19	0	1596532	17622	1705	138
ERR1135363_bin.22	18	99.3	0	2383648	56783	2141	68
ERR1135364_bin.79	6	94.43	1.5	2385962	15676	1899	217
ERR1135366_bin.11	4	87.91	0	956743	103916	952	15
ERR1135368_bin.10	5	99.88	0.75	1522387	75639	1580	34
ERR1135370_bin.25	1	89.24	0.77	2635042	21578	2537	205
ERR1135370_bin.31	1	95.65	0.24	2007506	72286	1886	41
ERR1135371_bin.19	1	82.17	2.87	1940639	8956	1776	268
ERR1135371_bin.2	5	86.25	2.6	1752436	8369	1662	247
ERR1135371_bin.25	1	96.88	3.26	3705323	42521	3579	211
ERR1135371_bin.30	1	75.67	2.77	2206556	6230	2322	396
ERR1135371_bin.47	1	81.46	2.73	3457068	20330	3049	256
ERR1135373_bin.22	94	98.88	0.45	1827094	26638	1801	105
ERR1135373_bin.49	17	93.06	0.37	2337995	13371	1796	204
ERR1135374_bin.10	15	97.43	1.11	2697337	29294	2509	156
ERR1135376_bin.19	66	97.75	0	1635651	26439	1708	107
ERR1135377_bin.41	53	97.9	0	2347146	64342	2091	56
ERR1135378_bin.20	5	81.56	0	1546709	42551	1658	64
ERR1135378_bin.53	30	95.8	0	2357862	91435	2122	53
ERR1135378_bin.9	6	80.87	0	1333741	10305	1422	167
ERR1135379_bin.27	11	81.78	1.55	2411743	10061	2225	290
ERR1135380_bin.23	12	96.79	0	2292311	56702	1877	70
ERR1135380_bin.51	4	95.97	0	1580171	96272	1535	23
ERR1135381_bin.24	5	96.68	0.83	3771363	57263	3045	131
ERR1135383_bin.11	178	98.15	0.19	2654125	57327	2176	70
ERR1135383_bin.33	4	95.75	0.17	1919896	29744	1788	97
ERR1135383_bin.49	2	86.19	0.67	1858350	8044	1893	271
ERR1135385_bin.10	1	96.7	2.4	1647656	60302	1823	59
ERR1135385_bin.11	8	97.76	1.9	1656216	24554	1631	91
ERR1135385_bin.68	1	80.9	3.37	1301336	21504	1448	102
ERR1135385_bin.9	1	75.28	1.5	767131	9721	853	100
ERR1135386_bin.37	68	95.9	0.7	1542167	23693	1524	97
ERR1135387_bin.51	3	87.88	1.17	1807264	8685	1941	249
ERR1135388_bin.55	3	92.38	1.45	2753732	16320	2357	228
ERR1135389_bin.14	1	87.76	0	2294472	20863	2020	154
ERR1135389_bin.43	1	78.95	1.84	1892269	7110	1809	302
ERR1135389_bin.51	2	97.32	0	1806764	60535	1730	51
ERR1135389_bin.61	25	98.6	0	2489837	49218	2311	86
ERR1135391_bin.3	1	90.46	2.35	1693623	23982	1714	111
ERR1135391_bin.5	1	92.53	0.73	1986881	16738	2092	177
ERR1135396_bin.46	1	75.77	4.15	1785300	6806	1950	306
ERR1135397_bin.48	1	98.85	1.54	2769680	77506	2656	55
ERR1135398_bin.29	1	93.29	0.67	1889616	25690	1829	117
ERR1135399_bin.16	1	81.92	6.08	2521798	5962	2535	446
ERR1135400_bin.30	1	89.3	0.37	2397106	13108	1792	224
ERR1135401_bin.33	50	100	0	1594337	52348	1717	56
ERR1135401_bin.43	1	90.17	1.85	1347218	31616	1449	73
ERR1135402_bin.1	2	75.1	0.22	1250175	56819	1313	39
ERR1135403_bin.7	8	98.43	0	2280537	52770	2101	71
ERR1135406_bin.22	73	97.58	0	1665015	46915	1603	62
ERR1135407_bin.19	1	90.96	5.03	2931023	9905	2661	376
ERR1135407_bin.2	7	89.44	0	1643842	19648	1704	132
ERR1135407_bin.25	3	94.72	1.01	1690100	34935	1644	71
ERR1135407_bin.53	2	89.33	1.12	1335417	21897	1392	109
ERR1135408_bin.15	1	95.3	0.39	2496401	18310	2458	195
ERR1135408_bin.67	10	92.62	0.24	1885290	24183	1648	118
ERR1135409_bin.26	6	84.62	0	1534070	15574	1615	131
ERR1135410_bin.13	68	96.57	0	2498429	28587	2070	136
ERR1135410_bin.80	17	98.51	0.19	4239072	34477	4073	203
ERR1135415_bin.35	1	92.6	0	2276324	29955	2111	112
ERR1135415_bin.65	1	86.18	2.02	1536379	8468	1654	221
ERR1135416_bin.41	3	95.08	0.48	1710991	24346	1496	100
ERR1135417_bin.29	1	79.58	1.68	1751670	6682	1844	288
ERR1135417_bin.79	1	77.84	0.71	1210531	6676	1355	203
ERR1135418_bin.17	10	85.74	2.59	1771227	10230	1913	221
ERR1135419_bin.22	3	90.1	0.34	1663757	16492	1688	142
ERR1135419_bin.50	4	87.13	0.73	1772222	26016	1553	94
ERR1135419_bin.55	14	96.71	0	2327518	42403	2246	96
ERR1135419_bin.72	1	97.46	0.11	1676067	32302	1684	94
ERR1135419_bin.8	1	85.69	1.17	1950674	11017	1975	224
ERR1135420_bin.28	50	98.6	0.23	3048668	45331	2850	109
ERR1135421_bin.27	9	97.4	0.74	3234422	63133	2543	71
ERR1135422_bin.26	7	97.99	0.67	1815948	57167	1715	50
ERR1135422_bin.56	1	80.34	0	1618552	15034	1780	161
ERR1135422_bin.61	7	81.82	0.35	1583315	12150	1697	177
ERR1135424_bin.1	14	93.76	1.63	2429420	25712	2260	146
ERR1135425_bin.34	1	93.48	1.51	1884368	13839	1923	187
ERR1135426_bin.24	4	97.65	2.01	1992057	18666	1974	164
ERR1135426_bin.43	1	88.7	1.4	1679981	8383	1731	239
ERR1135426_bin.50	5	91.95	0	1420136	8723	1403	205
ERR1135426_bin.54	1	75.86	0	1165695	8400	1246	166
ERR1135429_bin.48	1	86.85	3.76	1769808	13071	1594	174
ERR1135433_bin.33	3	93.85	2.03	2113647	16722	2117	179

SGBs ID	MAGs in SGBs (cluster number)	Completeness	Contamination	Length	N50	Gene number	Contig number
ERR1135435_bin.8	42	96.85	0	1796333	16999	1816	140
ERR1135439_bin.5	2	75.38	0.22	1472962	28681	1618	105
ERR1135439_bin.54	3	84.77	1.51	1578126	14230	1574	146
ERR1135441_bin.14	1	88.81	1.1	1408882	12780	1516	139
ERR1135441_bin.78	28	97.9	0.23	2872809	59197	2696	113
ERR1135445_bin.40	14	94.59	0	1960566	22001	1915	137
ERR1135446_bin.30	10	87.47	1.01	1654215	10717	1681	201
ERR1135446_bin.34	14	93.12	0.27	2717726	17135	2386	213
ERR1135447_bin.18	5	94.66	1.28	1174370	20118	1255	88
ERR1135447_bin.34	15	89.75	0	1581307	31744	1562	66
ERR1135449_bin.10	3	83.02	0.94	1241018	14243	1260	109
ERR1135449_bin.13	1	93.64	1.21	1710290	37060	1601	72
ERR1135450_bin.9	55	88.06	0	1530673	16310	1539	119
ERR1135452_bin.26	35	97.99	0	1809744	55100	1694	43
ERR1135453_bin.4	22	93.08	2.39	2647508	31691	2593	145
ERR1135453_bin.41	1	79.87	0.67	1353980	17061	1420	114
ERR1135453_bin.49	9	98.39	1.08	2222094	59077	1808	81
ERR1135455_bin.2	31	97.1	1.89	2420253	32158	2263	114
ERR1135456_bin.15	4	95.04	0	2132478	126418	1923	25
ERR1135456_bin.36	10	90.94	0	1595740	41304	1612	55
ERR1135458_bin.26	3	92.38	1.9	1552192	22615	1598	116
ERR1135458_bin.47	4	95.17	0.74	2438934	18078	1840	167
ERR1135458_bin.60	1	96.08	1.01	1807017	51566	1678	58
ERR1135458_bin.70	12	90.31	0.85	1846138	55444	1760	57
ERR1135459_bin.47	1	81.46	1.12	875669	20131	942	65
ERR1135459_bin.65	2	92.17	1.7	1763742	10496	1770	204
ERR1135460_bin.61	69	92.77	0	1354242	25021	1401	92
ERR1135462_bin.12	5	94.52	0	1679881	32291	1595	69
ERR1135462_bin.24	1	81.19	6.21	2346703	11073	2284	283
ERR1135462_bin.8	3	95.75	0.34	2454236	31777	2273	115
ERR1135464_bin.16	2	78.57	0	747425	14533	790	60
ERR1135464_bin.47	2	87.75	1.71	2099803	14519	2190	230
ERR1135464_bin.7	34	95.32	0.16	2103549	111103	1736	43
ERR1135464_bin.77	9	95.97	0	2522456	72606	2275	56
ERR1135464_bin.8	5	95.3	0	1637882	44440	1627	58
ERR1135465_bin.7	13	98.39	0	2125354	15263	2156	198
ERR1135465_bin.9	74	96.04	1.26	2438182	35588	2011	102
ERR1135466_bin.30	10	93.41	0	1711702	34740	1667	79
ERR1135466_bin.58	1	95.1	0.28	2211761	77676	1997	67
ERR1135467_bin.21	6	93.57	3.58	1907130	15017	2002	175
ERR1135468_bin.29	6	98.4	0.8	1730231	22657	1734	108
ERR1135468_bin.57	1	80.54	1.47	1503806	9159	1543	194
ERR1135470_bin.34	10	95.04	0.57	1935113	38662	1874	85
ERR1135471_bin.11	3	94.41	0	1745799	46980	1751	57
ERR1135471_bin.59	2	79.18	0	3328010	47770	2466	121
ERR1135472_bin.52	12	97.5	1.15	4163546	130368	2997	61
ERR1135472_bin.8	2	86.58	0.67	1909810	15601	1909	164
ERR1135472_bin.83	23	97.43	1.34	1615125	16376	1644	137
TP001_bin.109	4	96.86	0.29	2069956	26537	1917	118
TP001_bin.117	8	86.54	1.07	1768638	9579	1524	215
TP001_bin.18	2	95.22	1.5	2075824	32462	2205	116
TP001_bin.27	1	84.62	1.28	1750344	29752	1740	79
TP001_bin.35	3	86.51	3.27	2347993	8312	2168	335
TP001_bin.41	1	76.49	0.61	1194378	5845	1282	222
TP001_bin.56	1	87.27	6.54	4074626	8352	4286	575
TP001_bin.90	1	77.19	2.63	1988438	11542	1987	227
TP002_bin.105	3	93.82	0.94	1825798	31644	1640	97
TP002_bin.106	1	87.48	3.3	1451447	12053	1453	166
TP002_bin.136	1	92.65	0.34	2197272	14663	2133	207
TP002_bin.148	1	76.36	3.02	1309008	5624	1392	248
TP002_bin.164	1	96.86	1.63	2587734	41828	2413	96
TP002_bin.4	2	89.82	1.79	3381247	26156	3506	275
TP002_bin.67	2	88.87	0	1675112	19718	1541	119
TP002_bin.70	1	87.98	3.53	2329367	16586	2188	204
TP002_bin.75	1	82.13	3.02	2213173	6856	2456	367
TP002_bin.8	1	81.26	3.1	1772837	8876	1727	244
TP002_bin.84	1	97.75	2.25	1715941	42285	1590	57
TP002_bin.87	4	100	0	2702752	100094	2554	65
TP003_bin.127	2	95.8	3.48	3798561	13065	3937	426
TP003_bin.65	10	97.74	0.75	734391	36233	1331	37
TP003MC_bin.114	3	93.66	1.34	1976223	42269	1622	53
TP003MC_bin.120	4	89.36	0.02	3081545	20787	2572	221
TP003MC_bin.13	1	95.5	6.75	4310119	13727	4413	470
TP004MC_bin.13	1	80.22	3.76	2321495	7197	2154	361
TP004MC_bin.38	1	84	1.33	1267827	8580	1275	186
TP004MC_bin.41	4	93.55	1.27	1646045	48412	1586	65
TP004MC_bin.63	2	96.86	2.85	2367736	74960	2266	54
TP004MC_bin.66	1	98.67	8	2412592	20188	2281	191
TP004MC_bin.67	8	96.63	0	1528173	68132	1473	41
TP004MC_bin.86	1	81.12	0.67	2025479	28019	1763	113
TP005_bin.147	1	88.4	3.76	2434323	13612	2310	253
TP005_bin.3	1	80.2	2.85	1410438	10611	1393	170
TP005_bin.59	2	86.97	0.51	1704928	7907	1910	264
TP005_bin.79	1	76.54	2.87	1907087	10853	1964	225
TP005_bin.95	1	91.47	1.36	2030384	32778	1975	95
TP005MC_bin.38	1	78.68	2.91	1097959	6360	1125	191
TP005MC_bin.82	1	96.64	0	1784025	25555	1752	94
TP006MC_bin.121	1	87.39	0	1399964	13887	1343	129

SGBs ID	MAGs in SGBs (cluster number)	Completeness	Contamination	Length	N50	Gene number	Contig number
TP006MC_bin.130	1	82.44	4.18	2729207	35678	2661	118
TP006MC_bin.14	20	85.11	1.38	1977118	38397	1702	69
TP006MC_bin.52	1	79.03	4.99	1296381	10289	1340	154
TP006MC_bin.74	3	92.52	0	2014955	55848	1759	62
TP007MC_bin.110	1	90.02	2.02	2216507	14758	2223	205
TP007MC_bin.123	1	85.07	3.48	2952216	9089	3017	398
TP007MC_bin.132	1	82.93	2.96	2100054	13730	1770	197
TP007MC_bin.20	1	89.85	2.35	2374906	14910	2342	229
TP007MC_bin.49	1	85.19	0.85	1687567	56601	1793	57
TP007MC_bin.50	22	96.43	0.48	2155776	175038	1767	22
TP007MC_bin.55	1	88.03	3.24	2595886	30292	2001	141
TP008_bin.11	19	93.57	0.63	2132560	105130	1779	35
TP008_bin.114	1	97.01	1.13	2431979	51677	2000	75
TP008_bin.61	1	85.46	3.58	2465548	8918	2210	323
TP008_bin.78	1	97.32	1.57	3315448	34494	3071	167
TP008MC_bin.100	1	88.15	4.48	2489536	25993	2226	174
TP008MC_bin.139	1	77.37	2.46	1910935	9727	1693	239
TP008MC_bin.31	5	91.58	0.67	1897184	36330	1576	74
TP008MC_bin.33	1	92.82	0.96	2290254	11666	1860	239
TP008MC_bin.72	2	88.02	4.6	3315393	10468	3284	413
TP008MC_bin.8	1	86.13	2.13	1553064	30865	1390	77
TP008MC_bin.81	1	91.6	2.42	2719374	25116	2272	163
TP008MC_bin.88	10	89.33	1.02	2344854	12618	1794	224
TP009_bin.103	1	86.92	3.95	2215188	16798	2131	178
TP009_bin.25	4	96.64	0.34	1807341	167095	1702	25
TP009_bin.28	2	80.05	1.35	1569428	9521	1711	214
TP009_bin.4	1	82.88	5.65	1734591	6038	1692	320
TP009_bin.41	1	93.18	1.45	2457910	22214	2362	148
TP009_bin.49	1	80.21	4.59	2247912	13019	2175	251
TP009_bin.66	1	87.94	6.01	2484718	17260	2493	230
TP010_bin.100	4	86.38	3.16	2076942	11174	2105	250
TP010_bin.119	113	98.88	0.37	1451508	23114	1489	94
TP010_bin.76	1	89.56	2.33	2794489	14736	2319	267
TP011_bin.101	1	86.7	3.03	2861416	10601	2403	343
TP011_bin.135	1	90.53	3.09	3158437	20364	3024	237
TP011_bin.6	30	100	0.27	2235105	167984	2026	22
TP011_bin.67	1	87.94	0.67	1995900	10734	2115	240
TP011_bin.84	4	94.01	0	2190750	25199	1829	118
TP011_bin.85	1	84.68	0.78	1396498	12603	1412	151
TP011_bin.90	2	91.13	0.54	1776085	119174	1444	26
TP011_bin.97	4	86.72	0.19	1902685	38315	1601	66
TP012_bin.137	1	80.6	2.11	1951649	12055	1469	182
TP012_bin.29	3	89.89	1.69	1279349	11947	1212	134
TP012_bin.3	1	91.91	1.36	2436490	23349	2053	138
TP012_bin.39	25	98.9	0	2525646	59684	2316	71
TP012_bin.49	1	84.4	4.37	2433280	20383	2355	173
TP012_bin.56	4	91.72	2.68	2662090	26793	2405	162
TP012_bin.62	2	84.47	2.01	1395046	6880	1449	222
TP012_bin.66	1	76	0.56	1943659	48682	1974	87
TP012_bin.84	1	84.69	5.7	1734722	8400	1917	258
TP012_bin.9	3	96.99	0	1684341	70922	1664	46
TP012MC_bin.25	2	94.3	1.17	1683813	27591	1651	79
TP012MC_bin.71	1	86.55	0.22	2331255	32312	2228	122
TP012MC_bin.85	2	99.33	0	2769582	117669	2456	45
TP014_bin.24	3	94.51	2.53	2910182	16344	2468	248
TP014_bin.35	1	93.01	0	1437524	21751	1468	91
TP014_bin.39	2	93.31	1.2	2795959	49356	2179	75
TP014_bin.67	1	97.85	1.08	1706995	30643	1827	79
TP014_bin.7	1	75.77	1.06	1394508	14349	1493	132
TP014_bin.70	1	81.6	0.86	1192003	8275	1387	183
TP014_bin.83	8	94.07	0.57	3028854	30259	2503	161
TP014MC_bin.156	1	95.77	6.09	2201875	22681	2064	138
TP014MC_bin.61	3	92.1	3.57	2442221	22526	2199	169
TP014MC_bin.66	1	90.85	3.23	2334922	19294	2217	187
TP014MC_bin.90	8	92.47	1.01	2569676	12857	2101	255
TP015_bin.102	1	96.09	6.38	2962810	21244	2972	222
TP015_bin.103	5	93.91	2.42	2821133	36541	2569	119
TP015_bin.111	5	98.74	1.92	3822256	42665	3627	179
TP015_bin.30	1	93.77	0	1634833	54494	1592	58
TP015_bin.34	2	92.53	2.9	2511722	28859	2082	144
TP015_bin.39	9	94.27	2.4	2765841	35268	2350	127
TP015_bin.88	1	87.07	2.14	2114297	10980	2094	243
TP015MC_bin.116	1	89.7	5.45	2774172	13810	2705	271
TP015MC_bin.135	1	85.71	1.1	985282	10043	1060	122
TP015MC_bin.18	2	88.88	2.71	2717073	54004	2460	81
TP015MC_bin.36	1	87.01	2.15	1342370	11044	1407	167
TP015MC_bin.37	1	89.74	1.07	1712903	46135	1680	57
TP015MC_bin.54	1	85.32	3.33	2168196	35167	1677	84
TP015MC_bin.62	1	91.22	1.86	2722379	31855	2604	162
TP015MC_bin.75	1	79.76	4.29	2157200	11299	2016	262
TP015MC_bin.76	1	97.7	0.57	2878267	137724	2750	39
TP015MC_bin.77	4	91.4	0	2310070	75459	1865	48
TP015MC_bin.87	1	80.51	0.58	1312187	25798	1146	69
TP017MC_bin.126	7	90.24	0	1741497	69030	1519	54
TP017MC_bin.2	3	94.63	1.79	1621389	16973	1647	135
TP017MC_bin.98	3	95.14	0.37	2450799	46751	1940	82
TP018_bin.101	1	90.51	7.76	3281820	16036	3138	312
TP018_bin.105	6	90.48	0	1923576	50017	1601	52

SGBs ID	MAGs in SGBs (cluster number)	Completeness	Contamination	Length	N50	Gene number	Contig number
TP018_bin.180	13	91.89	2.56	2645088	17309	2513	232
TP018_bin.26	2	89.88	3.57	2029730	10997	1985	229
TP018_bin.55	2	97.32	0	2455942	60222	2147	74
TP018_bin.78	1	85.67	3.42	2231700	27987	2416	133
TP018_bin.85	3	95.3	0	2559269	73274	2289	52
TP018MC_bin.102	1	89.09	2.35	2251794	10663	1936	272
TP018MC_bin.11	1	94.29	1.79	2058632	10162	2004	263
TP018MC_bin.115	4	94.16	1	1976593	20911	1663	170
TP018MC_bin.143	1	81.88	1.26	1810620	7490	1666	283
TP018MC_bin.156	1	84.62	1.1	826927	20059	862	54
TP018MC_bin.157	2	83.11	0.03	1634198	12188	1554	168
TP018MC_bin.177	1	93.63	0	1436857	14345	1467	147
TP018MC_bin.185	1	93.13	1.37	1410182	18624	1462	103
TP018MC_bin.51	2	93.65	0.81	2284933	36256	1750	98
TP018MC_bin.69	4	84.36	1.95	2202548	27439	2071	120
TP018MC_bin.74	10	93.48	1.93	2401948	40134	2162	104
TP018MC_bin.85	1	94.78	3.63	2450301	20169	2359	167
TP019MC_bin.103	1	86.19	0	1402532	75684	1282	28
TP019MC_bin.30	17	89.19	0.03	3582817	18516	2942	278
TP019MC_bin.8	1	92.93	5.11	2093713	15439	2213	181
TP019MC_bin.9	1	95.84	8.72	2661352	17223	2697	254
TP019MC_bin.95	1	98.11	1.6	2456089	33688	2037	107
TP020_bin.103	3	91.25	3.97	2238587	16276	1827	184
TP020_bin.111	2	85.92	1.02	2193922	15070	1984	213
TP020_bin.122	4	82.26	0.66	1772168	9707	1604	227
TP020_bin.23	2	78.04	3.67	2008384	8054	1812	304
TP020_bin.26	5	97.65	0	2476735	13592	2229	243
TP020_bin.5	5	99.04	0.48	1928189	47206	1646	66
TP020MC_bin.105	14	93.95	0.43	1594615	14911	1652	147
TP020MC_bin.144	1	91.57	7.87	1929881	21358	2087	151
TP020MC_bin.157	1	96.43	3.11	2066697	53883	1738	67
TP020MC_bin.23	1	79.57	4.25	1356469	24484	1237	87
TP020MC_bin.75	2	78.28	3.78	1971303	7351	1958	310
TP021_bin.103	3	93.01	0	1417224	17117	1445	106
TP021_bin.106	3	83.27	0.56	2104965	21227	1811	142
TP021_bin.115	6	94.51	0	945367	82542	961	21
TP021_bin.119	1	93.14	0	1260137	10063	1343	157
TP021_bin.140	2	91.73	1.67	2271766	39817	1871	75
TP021_bin.147	5	78	0	1823373	46909	1684	57
TP021_bin.156	1	85.84	0.48	1449146	16772	1354	105
TP021_bin.28	1	97.2	3.37	3016258	65743	2737	99
TP021_bin.45	2	96.64	0	1888759	49395	1789	57
TP021_bin.51	4	92.46	0	1333700	15302	1379	111
TP021_bin.55	2	96.43	0	2646271	84993	2176	65
TP021_bin.56	2	83.33	0	1808752	22094	1719	124
TP021_bin.7	1	88.98	7.46	2914289	15694	2896	274
TP021_bin.93	1	85.96	3.93	1590422	38683	1548	67
TP021MC_bin.139	1	92.47	3.23	1399848	23845	1479	93
TP021MC_bin.45	2	76.08	0.74	2243704	18813	1694	145
TP021MC_bin.53	11	98.12	0.54	2599235	33256	2208	120
TP021MC_bin.71	1	83.27	1.67	1905728	15829	1743	181
TP021MC_bin.72	1	91.78	1.68	1752022	20141	1712	128
TP022_bin.117	1	85.39	3.4	2330965	33931	2115	105
TP022_bin.99	1	86.93	0.84	1791304	8415	1867	242
TP022MC_bin.127	4	97.85	0	1394988	77355	1389	23
TP022MC_bin.16	4	93.55	1.61	2238034	48778	1885	56
TP022MC_bin.63	2	92.28	0.83	3247673	38914	2872	114
TP022MC_bin.70	1	94.03	3.75	3092512	25671	2995	227
TP023_bin.58	2	79.95	1.02	1775601	6612	1865	310
TP023_bin.70	1	97.13	1.34	2649393	235109	2438	19
TP023_bin.85	1	84.68	4.14	1849292	17738	1547	156
TP024_bin.106	3	96.66	0.37	2710016	50880	2145	84
TP024_bin.115	1	91.7	2.35	4175512	25113	4185	356
TP024_bin.125	12	94.76	0	2484952	31547	2240	120
TP024_bin.130	1	79.83	4.36	1599401	6165	1688	299
TP024_bin.19	3	97.34	1.35	1664776	15261	1797	147
TP024_bin.56	1	91.13	0.81	1729547	11869	1689	183
TP024_bin.91	5	98.85	1.05	2685480	52868	2414	72
TP024MC_bin.118	2	94.75	1.33	2302946	34143	2153	107
TP024MC_bin.130	1	91.99	4.57	3702369	15810	3729	365
TP024MC_bin.131	13	94.49	0.56	2463695	31917	1873	100
TP024MC_bin.53	3	81.39	1.14	1816422	8151	1939	261
TP024MC_bin.61	2	80.96	2.15	2382904	9408	2216	327
TP025_bin.106	7	90.98	0.81	1880998	14185	1857	171
TP025_bin.115	5	98.85	1.15	2727014	120139	2568	36
TP025_bin.117	1	91.72	6.96	3282672	15791	3295	322
TP025_bin.120	1	91.01	2.41	1542662	48599	1800	70
TP025_bin.136	5	94.13	0.67	2251707	69673	2172	53
TP025_bin.138	21	89.57	0.59	1717066	108219	1484	23
TP025_bin.139	1	82.22	0.67	2040202	50582	1911	60
TP025_bin.142	13	95.43	0.54	1788739	59716	1554	43
TP025_bin.18	4	82.92	0.72	2053180	10441	2003	234
TP025_bin.31	3	93.74	0.95	2206143	10449	2214	272
TP025_bin.34	2	84.64	4.14	2711428	11584	2676	295
TP025_bin.45	3	82.07	0	2015722	14896	1735	165
TP025_bin.59	1	83.13	2.35	1845005	7469	2048	290
TP025_bin.6	1	95.44	0	1403907	32132	1369	61

SGBs ID	MAGs in SGBs (cluster number)	Completeness	Contamination	Length	N50	Gene number	Contig number
TP025_bin.93	1	95.97	0.36	1825509	16545	1851	158
TP025_bin.98	6	83.9	1.35	1423210	18323	1354	115
TP025MC_bin.148	13	89.5	0.71	2375347	24958	2002	126
TP025MC_bin.63	1	89.11	0.89	2937915	36652	2673	122
TP026_bin.108	4	90.32	0	1378182	13644	1427	130
TP026_bin.115	5	79.56	0.68	1023576	43612	1031	40
TP026_bin.154	5	89.01	0	899346	261729	902	6
TP026_bin.65	3	77.92	1.01	2014910	7837	1744	295
TP026_bin.87	1	91.53	1.84	1785617	12048	1790	194
TP027_bin.100	3	97.2	1.12	2719499	35882	2205	127
TP027_bin.147	4	81.8	0.94	2102041	18649	1754	161
TP027_bin.153	1	96.14	2.63	2946506	38636	2351	105
TP027_bin.157	1	86.11	3.19	2245575	9209	2336	299
TP027_bin.5	2	94.63	0.34	2239324	41227	2056	85
TP027_bin.78	1	82.8	0.63	1797509	11448	1770	207
TP027_bin.9	2	78.25	0	1577116	10375	1598	208
TP027_bin.91	3	93.48	1.25	1905299	36745	1731	82
TP027MC_bin.112	6	98.55	0.48	2576937	98948	2459	46
TP027MC_bin.116	18	97.55	0	2216079	154861	1961	25
TP027MC_bin.12	10	95.8	0.31	2743142	34242	2282	128
TP027MC_bin.147	2	91.61	1.16	2641645	31919	2464	125
TP027MC_bin.153	1	80.15	0	937319	5564	1052	179
TP027MC_bin.2	2	91.2	3.18	2911748	17666	2854	247
TP027MC_bin.27	1	96.77	0	1422517	27083	1432	62
TP027MC_bin.38	1	86.04	0	2026357	62807	1727	45
TP027MC_bin.89	4	89.96	2.35	2703692	17127	2329	236
TP027MC_bin.96	1	85.75	1.01	1449154	13538	1455	147
TP028_bin.145	4	90.76	0.37	2330031	15469	1865	207
TP028_bin.30	3	87.17	0.39	2376766	40712	1979	86
TP028_bin.51	1	90.34	4.87	2306611	15311	2097	218
TP028_bin.79	3	88.45	2.75	2877696	12631	2398	289
TP029_bin.122	1	91.2	1.09	2428345	22629	2165	156
TP029_bin.128	1	90.45	2.88	1475490	13164	1531	155
TP029_bin.15	1	100	1.47	3042375	85137	2795	56
TP029_bin.154	3	83.18	1.3	2375836	15141	1974	218
TP029_bin.158	4	97.2	0	2588493	58077	2420	70
TP029_bin.169	1	80.08	2.3	1764859	13546	1605	192
TP029_bin.43	1	83.6	2.28	1797519	47109	1565	81
TP029_bin.61	1	90.32	2.09	1519804	12906	1394	151
TP029_bin.9	1	86.81	0	904820	20711	941	65
TP029MC_bin.10	1	92.04	1.67	2349411	33167	2251	140
TP029MC_bin.101	1	93.96	1.34	1806553	20252	1780	132
TP029MC_bin.102	1	77.41	5.17	2868519	22906	2390	182
TP029MC_bin.20	2	92.73	0	2297287	13198	2267	244
TP029MC_bin.42	3	97.63	2.39	2925358	41466	2285	107
TP029MC_bin.50	6	93.57	0	1759516	56441	1578	48
TP029MC_bin.53	1	90.13	2.82	2255741	14331	2389	212
TP029MC_bin.85	1	83.69	1.13	1743067	9217	1651	233
TP029MC_bin.89	1	89.98	0.72	1900202	18933	1611	150
TP029MC_bin.9	2	96.36	0.48	2730371	147034	2488	35
TP029MC_bin.90	1	77.71	0.13	1980661	11800	1685	202
TP030MC_bin.109	3	93.82	0.65	2476788	38106	2073	97
TP030MC_bin.27	2	79.44	1.7	1569404	8154	1504	230
TP030MC_bin.3	1	95.51	8.03	1762326	47540	1741	57
TP030MC_bin.48	5	98.22	2.24	3127377	33529	2842	173
TP030MC_bin.59	4	100	0.67	2613855	94592	2466	51
TP030MC_bin.63	7	91.54	0.37	2377484	20427	1890	156
TP031MC_bin.105	5	95.72	0	3169326	76692	2388	43
TP031MC_bin.2	1	92.25	2.23	2858189	16231	2748	247
TP031MC_bin.26	13	98.66	0	2438545	88099	2222	45
TP031MC_bin.46	5	92.89	1.69	2186211	78073	2029	49
TP031MC_bin.51	1	76.17	1.13	2460715	49874	2158	70
TP031MC_bin.68	1	95.71	5.61	4088876	55722	3857	217
TP033_bin.130	1	84.5	1.57	2235449	6954	2326	361
TP033_bin.32	4	97.99	0.79	2888283	70513	2810	84
TP033_bin.74	8	96.24	2.15	2766441	22635	2193	164
TP033_bin.8	4	90.24	1.52	1713463	39610	1533	75
TP033_bin.92	9	93.61	0	2417282	53313	2180	72
TP034MC_bin.112	1	95.58	5.14	4245455	15858	4477	414
TP034MC_bin.136	6	95.58	0.74	2561959	47181	2016	73
TP034MC_bin.147	5	83.15	1.39	2186811	36677	1781	83
TP034MC_bin.156	9	90.09	0.37	1931104	49879	1601	52
TP034MC_bin.157	9	90.7	0.36	1515427	39666	1494	52
TP034MC_bin.30	2	94.54	0.49	2568933	56050	2026	68
TP034MC_bin.40	1	75.86	0	2326171	14638	1724	195
TP034MC_bin.53	5	77.66	0.08	2289690	36012	1865	94
TP034MC_bin.55	1	87.75	0	1770947	18245	1783	127
TP034MC_bin.57	1	93.27	1.74	2838191	19527	2997	218
TP034MC_bin.67	18	91.68	0.12	2194648	26652	2122	122
TP034MC_bin.76	1	90.82	1.81	2576626	24802	2445	155
TP034MC_bin.88	2	85.97	1.65	2563157	58716	2319	83
TP034MC_bin.90	3	86.35	2.39	1950729	20489	1814	159
TP034MC_bin.98	1	93.62	0.67	2535713	18296	2391	195
TP036_bin.108	1	78.35	0.13	1750836	14866	1686	164
TP036_bin.120	1	85.51	2.52	2164238	7621	2250	336
TP036_bin.122	2	97.42	1.71	2524532	158307	2307	28
TP036_bin.44	2	81.05	4.39	3179423	9912	3112	397
TP036_bin.66	1	92.42	2.87	2988725	18499	2785	233

SGBs ID	MAGs in SGBs (cluster number)	Completeness	Contamination	Length	N50	Gene number	Contig number
TP036_bin.7	1	79.31	4.31	2235755	15732	1697	180
TP036_bin.79	2	94.68	3.66	2492808	82780	2279	41
TP036_bin.80	7	92.2	0	1934160	40817	1709	72
TP036MC_bin.106	11	95.97	1.61	1987488	84258	1682	30
TP036MC_bin.117	1	87.72	6.06	1940023	18738	1851	141
TP036MC_bin.15	3	91.74	2.35	1977372	38901	1908	81
TP036MC_bin.37	4	89.74	0.93	1901565	39861	1869	63
TP036MC_bin.50	2	90.5	1.56	2450981	35053	2069	130
TP036MC_bin.76	4	98.66	0.67	2763939	76253	2643	65
TP037MC_bin.162	1	94.51	1.1	1290125	68705	1281	30
TP037MC_bin.21	1	87.98	3.01	1226849	10938	1177	149
TP037MC_bin.37	1	78.51	1.1	755484	7715	884	114
TP037MC_bin.44	3	87.01	0.56	2478101	7247	2443	400
TP037MC_bin.57	1	80.11	5.54	1850410	8144	1903	286
TP040MC_bin.111	1	94.76	0.83	1720137	62115	1566	35
TP040MC_bin.126	1	94.22	3.57	3237289	11654	3264	396
TP040MC_bin.137	2	93.41	2.16	2228933	18751	1875	158
TP040MC_bin.156	1	76.09	0.4	1021552	6800	1053	163
TP040MC_bin.19	11	94.11	0.68	2748730	36737	2149	114
TP040MC_bin.35	1	89.32	2.27	2800163	8570	3086	410
TP040MC_bin.40	2	88.8	1.34	1896961	12218	1939	196
TP040MC_bin.6	1	94.82	2.11	3519228	11572	3794	397
TP040MC_bin.79	3	97.28	0.48	2373818	102398	1944	36
TP040MC_bin.9	6	86.22	1.01	1983061	8642	1878	267
TP040MC_bin.98	1	83.48	0.5	1328802	20871	1252	88
TP041_bin.136	1	92.66	3.91	2469950	23289	2439	168
TP041_bin.152	1	82.57	1.22	1926976	10464	1849	225
TP041_bin.163	2	86.49	0.84	2061366	19910	1641	137
TP041_bin.31	1	98.73	0	3148200	62078	2929	90
TP041_bin.33	2	89.55	2.22	2413887	23519	1938	159
TP041_bin.34	3	86.46	2.73	2608887	32268	2199	130
TP041MC_bin.110	1	79.64	0.17	1568257	70895	1480	44
TP041MC_bin.115	22	96.19	0	2058022	62891	1689	52
TP041MC_bin.117	2	93.26	0.92	2070356	30132	1594	100
TP041MC_bin.118	1	84.38	0	1454340	7350	1383	224
TP041MC_bin.125	4	87.91	0	835612	180265	839	10
TP041MC_bin.31	3	96.42	1.07	2392487	106941	1982	42
TP042_bin.125	1	77.62	0	1346861	8421	1387	191
TP042_bin.126	1	92.31	1.92	2136493	56127	1869	56
TP042_bin.127	62	98.9	0	3351689	72039	2951	76
TP042_bin.29	5	98.39	0.27	2132093	93993	1895	33
TP042_bin.53	2	90.7	3	2682178	26697	2545	154
TP043_bin.104	1	87.17	1.44	2322639	56959	1865	60
TP043_bin.118	2	96.63	1.12	1201563	26240	1126	70
TP043_bin.119	3	89.84	0	1764844	42090	1584	68
TP043_bin.127	3	94.38	0.56	1371666	11950	1384	142
TP043_bin.129	69	96.75	0	2273670	63840	1846	55
TP043_bin.130	1	85.71	0	975585	10581	979	111
TP043_bin.149	1	77.05	1.29	3099719	30430	2242	140
TP043_bin.15	11	84.76	0.84	3421308	11576	2969	370
TP043_bin.162	1	90.11	7.44	2626286	10894	2643	322
TP043_bin.169	23	96.42	1.13	2869158	32580	2476	141
TP043_bin.174	31	90.94	0	1611083	69977	1585	33
TP043_bin.177	1	93.01	0	1861477	109246	1920	40
TP043_bin.179	4	87.13	1.3	2250621	41494	1728	81
TP043_bin.18	5	95.87	0.03	2074357	43383	1699	80
TP043_bin.40	2	85.3	1.19	1986919	30406	1615	99
TP043_bin.49	1	100	2.92	1731799	30878	1575	92
TP043_bin.51	4	93.33	0.48	2131353	84297	1735	54
TP043_bin.52	3	82.76	0	2186484	27465	1865	115
TP043_bin.81	1	93.1	0	2035763	17706	1992	173
TP043_bin.93	1	79.42	5.34	2462696	8263	2553	376
TP043_bin.97	1	82.92	1.08	1515088	24596	1343	83
TP043_bin.98	1	78.08	1.51	1152121	5043	1186	240
TP043MC_bin.109	5	97.48	0	2411557	130543	1943	26
TP043MC_bin.127	1	79.44	0.56	1068929	8006	1102	165
TP043MC_bin.14	1	77.59	0.86	1824764	10675	1691	210
TP043MC_bin.142	2	97.33	0	2227132	31304	2103	103
TP043MC_bin.147	1	89.02	0.68	1799982	9714	1751	230
TP043MC_bin.22	4	90.32	0	1488348	53200	1541	49
TP043MC_bin.32	2	97.75	0.29	2046020	36608	1874	86
TP043MC_bin.59	2	92.49	1.54	2164186	12317	1813	222
TP044MC_bin.16	1	81.5	0	1817202	9583	1661	232
TP044MC_bin.5	2	82.04	1.72	2367130	15528	1768	201
TP044MC_bin.58	24	93.94	0.37	3189164	51645	2418	92
TP044MC_bin.62	1	89.1	2.3	3078159	17956	2676	255
TP044MC_bin.94	19	99.26	0	2665899	62719	2138	66
TP044MC_bin.96	9	87.92	0.38	2367716	25370	1907	125
TP045_bin.1	2	88.04	0	1308117	44546	1158	41
TP045_bin.106	5	87.91	0	860767	135344	853	12
TP045_bin.113	21	97.45	0	2598882	83096	2369	50
TP045_bin.120	1	82.23	0.03	1794088	26959	1513	98
TP045_bin.123	2	81.21	0.02	1550396	52686	1424	46
TP045_bin.129	1	78.15	4.81	1342249	7328	1320	211
TP045_bin.14	1	91.16	1.96	1611285	9911	1707	203
TP045_bin.16	2	77.44	1.23	1157256	6205	1198	209
TP045_bin.23	1	82.8	1.68	1733467	10905	1636	208
TP045_bin.32	1	76.3	1.34	1560762	8257	1406	228

SGBs ID	MAGs in SGBs (cluster number)	Completeness	Contamination	Length	N50	Gene number	Contig number
TP045_bin.44	1	84.48	3.09	1948087	9741	2074	266
TP045_bin.48	1	92.27	2.15	1997853	17663	1820	171
TP045_bin.86	7	87.13	2.06	2066118	25819	1754	125
TP045_bin.91	1	89.15	2.43	2269740	9980	1786	271
TP045_bin.93	1	79.48	1.29	1809301	18111	1882	158
TP047MC_bin.127	6	91.96	0.38	2443333	14956	2139	207
TP047MC_bin.136	1	89.24	0	1427590	24998	1415	78
TP047MC_bin.22	6	92.17	0.36	2004860	12866	2112	215
TP047MC_bin.79	6	83.84	0.95	1992913	47467	1647	70
TP047MC_bin.9	1	96.46	4.76	2332876	25306	2201	151
TP049MC_bin.106	2	84.12	1.69	2014183	16415	1833	175
TP049MC_bin.114	1	94.51	0	1349734	20823	1376	86
TP049MC_bin.125	1	87.18	4.27	1783899	39464	1818	72
TP049MC_bin.131	7	94.37	0.68	2751515	81781	2412	53
TP049MC_bin.155	2	81.98	4.39	1749658	11791	1494	188
TP049MC_bin.34	1	91.99	2.65	2821050	20066	2428	201
TP049MC_bin.39	1	94.94	1.9	2362437	43271	2356	102
TP049MC_bin.45	1	75.11	4.3	922869	4751	1089	200
TP049MC_bin.7	1	93.75	5.7	2071759	28706	2096	111
TP049MC_bin.72	1	93.94	0.59	2220874	13181	2036	235
TP049MC_bin.85	1	81.11	1.34	1951888	10744	2015	225
TP049MC_bin.88	1	81.53	2.39	3274438	21172	2843	232
TP051_bin.108	1	79.96	0.57	2302365	35134	2057	102
TP051_bin.119	1	86.18	1.56	2299657	7550	2272	348
TP051_bin.130	1	93.26	5.59	1556666	22092	1620	117
TP051_bin.134	1	87.71	2.03	2111249	14198	1988	197
TP051_bin.142	5	92.66	0	2369064	99922	2209	28
TP051_bin.24	1	79.78	0.34	2222213	30845	2118	116
TP051_bin.6	1	97.99	1.34	2540011	46031	2434	123
TP051_bin.64	3	94.52	0.24	2856791	74200	2367	83
TP051_bin.70	1	81.46	4.02	1601720	7165	1698	258
TP051_bin.95	1	83.21	5.22	2759794	7654	3017	452
TP052MC_bin.118	1	81.76	5.04	2366277	17049	2334	198
TP052MC_bin.128	1	91.65	0.86	2815181	60757	2507	79
TP052MC_bin.26	14	91.61	0	2579815	54601	2360	72
TP052MC_bin.32	2	87.53	2.39	2900447	25569	2402	164
TP052MC_bin.34	4	93.09	0	2095760	18033	2055	145
TP052MC_bin.39	1	93.22	2.54	2205031	51222	2058	79
TP052MC_bin.45	1	90.69	0.81	1943834	19433	1853	142
TP052MC_bin.56	1	84.14	2.09	2702380	12108	2413	288
TP052MC_bin.57	6	87.61	0	2232668	86675	1780	41
TP052MC_bin.61	2	93.85	2.91	2453297	30168	2344	112
TP052MC_bin.64	2	94.97	0	2042188	53501	1950	54
TP052MC_bin.82	1	82.41	2.52	2147825	7166	2262	332
TP052MC_bin.93	1	96.64	1.7	2336014	38574	2159	99
TP053MC_bin.10	1	86.55	2.38	1322127	46447	1287	44
TP053MC_bin.119	1	94.63	0	1305197	34984	1301	56
TP053MC_bin.136	2	90.43	2.42	2249796	71329	1706	48
TP053MC_bin.17	1	96.62	0.56	2488991	17944	2313	188
TP053MC_bin.2	3	95.33	2.79	2135761	14615	2045	188
TP053MC_bin.38	1	81.8	5.85	2303916	8433	2249	327
TP053MC_bin.7	2	89.43	1.4	1823085	10838	1807	216
TP053MC_bin.93	1	87.18	0.93	1618210	12586	1677	156
TP053MC_bin.97	1	88.39	1.16	2227481	13348	1798	220
TP056_bin.100	2	93.44	5.21	2059687	17454	1945	160
TP056_bin.102	4	92.62	0.51	1920416	94638	1626	34
TP056_bin.109	1	93.77	1.43	2552635	31652	2114	118
TP056_bin.37	5	89.62	0	2450307	8151	2436	352
TP056_bin.5	2	92.68	0	2309517	194897	1728	17
TP056_bin.56	1	93.49	1.49	2747631	23783	2313	174
TP056_bin.70	1	87.68	2.1	1521866	11321	1469	181
TP058_bin.103	1	89.53	2.73	2183657	59137	1587	57
TP058_bin.105	1	91.4	3.03	1406401	14729	1459	130
TP058_bin.17	1	75.38	5.03	1442021	4932	1653	298
TP058_bin.18	6	82.87	0.71	2349090	17611	1819	182
TP058_bin.27	3	93.75	1.29	2567723	23036	2109	161
TP058_bin.47	1	91.38	2.52	1889725	7422	1892	303
TP058_bin.50	11	94.55	0.19	2806156	68210	2202	59
TP058_bin.7	1	97.58	0.97	2777811	131607	2548	36
TP058_bin.70	1	75.82	4.81	1944896	6514	2036	339
TP058_bin.84	1	97.32	2.35	2531576	22866	2447	155
TP058_bin.85	2	93.82	1.12	1013602	159916	1062	14
TP058_bin.96	1	80.7	2.65	1906755	9571	1965	249
TP059MC_bin.112	3	87.8	1.34	1941224	14973	1927	163
TP059MC_bin.114	1	75.11	2.59	2269778	19536	1848	168
TP059MC_bin.116	1	94.29	2.38	1626651	13021	1765	169
TP059MC_bin.121	3	90.13	0	1608547	13468	1532	160
TP059MC_bin.127	1	81.3	4.58	1858731	8859	1853	257
TP059MC_bin.151	1	75.32	4.03	1366679	14535	1320	132
TP059MC_bin.181	1	83.51	1.72	2247824	17742	1941	177
TP059MC_bin.25	6	98.92	0.54	1968769	133975	1784	24
TP059MC_bin.84	10	93.72	0.84	2755195	56678	2248	76
TP059MC_bin.87	1	90.38	3.78	3364743	40836	2983	130

Supplementary Table 3. The classification of 1048 SGBs

SGBs ID	Classification
ERR1135178_bin.26	d__Bacteria;p__Cyanobacteria;c__Vampirovibrionia;o__Gastranaerophilales;f__Gastranaerophilaceae;g__CAG-196;s__
ERR1135178_bin.33	d__Bacteria;p__Firmicutes_A;c__Clostridia;o__Lachnospirales;f__Lachnospiraceae;g__COE1;s__COE1 sp001916965
ERR1135178_bin.34	d__Bacteria;p__Firmicutes;c__Bacilli;o__Lactobacillales;f__Lactobacillaceae;g__Lactobacillus;s__Lactobacillus johnsonii
ERR1135178_bin.36	d__Bacteria;p__Firmicutes_A;c__Clostridia;o__Oscillospirales;f__Oscillospiraceae;g__CAG-110;s__
ERR1135178_bin.56	d__Bacteria;p__Firmicutes_A;c__Clostridia;o__Lachnospirales;f__Lachnospiraceae;g__Agathobacter;s__Agathobacter faecis
ERR1135178_bin.57	d__Bacteria;p__Firmicutes_A;c__Clostridia;o__Lachnospirales;f__Lachnospiraceae;g__CAG-45;s__
ERR1135178_bin.73	d__Bacteria;p__Firmicutes_C;c__Negativicutes;o__Acidaminococcales;f__Acidaminococcaceae;g__Phascolarctobacterium_A;s__
ERR1135178_bin.9	d__Bacteria;p__Firmicutes_A;c__Clostridia;o__Lachnospirales;f__Lachnospiraceae;g__992a;s__992a sp001940245
ERR1135179_bin.22	d__Bacteria;p__Cyanobacteria;c__Vampirovibrionia;o__Gastranaerophilales;f__Gastranaerophilaceae;g__;s__
ERR1135179_bin.29	d__Bacteria;p__Firmicutes_A;c__Clostridia;o__Lachnospirales;f__Lachnospiraceae;g__CAG-95;s__
ERR1135180_bin.57	d__Bacteria;p__Proteobacteria;c__Alphaproteobacteria;o__Rs-D84;f__Rs-D84;g__Rs-D84;s__Rs-D84 sp900549365
ERR1135181_bin.2	d__Bacteria;p__Firmicutes;c__Bacilli;o__ML615J-28;f__CAG-313;g__UMGS1109;s__UMGS1109 sp900548675
ERR1135181_bin.32	d__Bacteria;p__Bacteroidota;c__Bacteroidia;o__Bacteroidales;f__Muribaculaceae;g__SFTJ01;s__SFTJ01 sp004557385
ERR1135181_bin.58	d__Bacteria;p__Firmicutes;c__Bacilli;o__RF39;f__UBA5348;g__CAG-1193;s__
ERR1135181_bin.62	d__Bacteria;p__Proteobacteria;c__Alphaproteobacteria;o__Rs-D84;f__Rs-D84;g__Rs-D84;s__
ERR1135181_bin.67	d__Bacteria;p__Bacteroidota;c__Bacteroidia;o__Bacteroidales;f__Paludibacteraceae;g__RF16;s__
ERR1135181_bin.68	d__Bacteria;p__Cyanobacteria;c__Vampirovibrionia;o__Gastranaerophilales;f__Gastranaerophilaceae;g__;s__
ERR1135182_bin.35	d__Bacteria;p__Bacteroidota;c__Bacteroidia;o__Bacteroidales;f__Muribaculaceae;g__UBA7173;s__UBA7173 sp004557825
ERR1135182_bin.53	d__Bacteria;p__Synergistota;c__Synergistia;o__Synergistales;f__Dethiosulfovibrionaceae;g__Pyramidobacter;s__
ERR1135183_bin.20	d__Bacteria;p__Cyanobacteria;c__Vampirovibrionia;o__Gastranaerophilales;f__Gastranaerophilaceae;g__CAG-196;s__
ERR1135183_bin.4	d__Bacteria;p__Firmicutes_A;c__Clostridia;o__Lachnospirales;f__Lachnospiraceae;g__;s__
ERR1135183_bin.75	d__Bacteria;p__Firmicutes_A;c__Clostridia;o__Lachnospirales;f__Lachnospiraceae;g__UBA7050;s__
ERR1135184_bin.93	d__Bacteria;p__Firmicutes_A;c__Clostridia;o__Lachnospirales;f__Lachnospiraceae;g__TWA4;s__
ERR1135185_bin.73	d__Bacteria;p__Bacteroidota;c__Bacteroidia;o__Bacteroidales;f__Bacteroidaceae;g__UBA4372;s__UBA4372 sp900543815
ERR1135187_bin.18	d__Bacteria;p__Firmicutes_A;c__Clostridia;o__Oscillospirales;f__Acutalibacteraceae;g__Eubacterium_R;s__
ERR1135187_bin.39	d__Bacteria;p__Elusimicrobiota;c__Elusimicrobia;o__Elusimicrobiales;f__Elusimicrobiaceae;g__;s__
ERR1135187_bin.47	d__Bacteria;p__Firmicutes_A;c__Clostridia;o__Lachnospirales;f__Lachnospiraceae;g__;s__
ERR1135187_bin.54	d__Bacteria;p__Bacteroidota;c__Bacteroidia;o__Bacteroidales;f__Tannerellaceae;g__Parabacteroides;s__Parabacteroides sp004562445
ERR1135187_bin.70	d__Bacteria;p__Firmicutes_A;c__Clostridia;o__Oscillospirales;f__Oscillospiraceae;g__CAG-83;s__
ERR1135187_bin.84	d__Bacteria;p__Fibrobacterota;c__Fibrobacteria;o__Fibrobacterales;f__Fibrobacteraceae;g__Fibrobacter_A;s__Fibrobacter_A intestinalis
ERR1135187_bin.93	d__Bacteria;p__Proteobacteria;c__Gammaproteobacteria;o__Burkholderiales;f__Burkholderiaceae;g__Duodenibacillus;s__Duodenibacillus sp900542805
ERR1135188_bin.12	d__Bacteria;p__Bacteroidota;c__Bacteroidia;o__Bacteroidales;f__Muribaculaceae;g__UBA7173;s__
ERR1135188_bin.29	d__Bacteria;p__Firmicutes_A;c__Clostridia;o__Oscillospirales;f__Oscillospiraceae;g__CAG-170;s__
ERR1135188_bin.33	d__Bacteria;p__Elusimicrobiota;c__Elusimicrobia;o__Elusimicrobiales;f__Elusimicrobiaceae;g__UBA1174;s__
ERR1135188_bin.61	d__Bacteria;p__Bacteroidota;c__Bacteroidia;o__Bacteroidales;f__Bacteroidaceae;g__Prevotella;s__Prevotella sp002251435
ERR1135188_bin.68	d__Bacteria;p__Bacteroidota;c__Bacteroidia;o__Bacteroidales;f__Paludibacteraceae;g__W0P28-013;s__W0P28-013 sp004557405
ERR1135188_bin.70	d__Bacteria;p__Bacteroidota;c__Bacteroidia;o__Bacteroidales;f__Muribaculaceae;g__;s__
ERR1135189_bin.31	d__Bacteria;p__Bacteroidota;c__Bacteroidia;o__Bacteroidales;f__Tannerellaceae;g__Parabacteroides;s__
ERR1135189_bin.44	d__Bacteria;p__Firmicutes_A;c__Clostridia_A;o__Christensenellales;f__CAG-917;g__CAG-1138;s__CAG-1138 sp000434675
ERR1135189_bin.5	d__Bacteria;p__Firmicutes_A;c__Clostridia;o__Oscillospirales;f__Oscillospiraceae;g__CAG-110;s__
ERR1135189_bin.63	d__Bacteria;p__Bacteroidota;c__Bacteroidia;o__Bacteroidales;f__Muribaculaceae;g__CAG-873;s__
ERR1135189_bin.8	d__Bacteria;p__Firmicutes_A;c__Clostridia_A;o__Christensenellales;f__UBA1242;g__UBA11490;s__UBA11490 sp003524095
ERR1135190_bin.104	d__Bacteria;p__Firmicutes_A;c__Clostridia;o__Oscillospirales;f__Ruminococcaceae;g__CAG-115;s__CAG-115 sp004554555
ERR1135190_bin.27	d__Bacteria;p__Bacteroidota;c__Bacteroidia;o__Bacteroidales;f__Bacteroidaceae;g__Prevotella;s__
ERR1135190_bin.32	d__Bacteria;p__Firmicutes;c__Bacilli;o__RFN20;f__CAG-826;g__UBA733;s__
ERR1135190_bin.34	d__Bacteria;p__Firmicutes_A;c__Clostridia;o__Oscillospirales;f__Acutalibacteraceae;g__Ruminococcus_E;s__
ERR1135190_bin.4	d__Bacteria;p__Firmicutes_A;c__Clostridia;o__Lachnospirales;f__Lachnospiraceae;g__CAG-127;s__
ERR1135190_bin.41	d__Bacteria;p__Firmicutes_A;c__Clostridia;o__Lachnospirales;f__Lachnospiraceae;g__UBA2882;s__
ERR1135190_bin.63	d__Bacteria;p__Firmicutes_A;c__Clostridia;o__Oscillospirales;f__Ruminococcaceae;g__UMGS1668;s__UMGS1668 sp004556975
ERR1135190_bin.64	d__Bacteria;p__Firmicutes_A;c__Clostridia;o__Oscillospirales;f__CAG-272;g__CAG-841;s__CAG-841 sp000437375
ERR1135190_bin.88	d__Bacteria;p__Firmicutes_A;c__Clostridia;o__Oscillospirales;f__Oscillospiraceae;g__CAG-110;s__
ERR1135190_bin.91	d__Bacteria;p__Firmicutes_A;c__Clostridia;o__Lachnospirales;f__Lachnospiraceae;g__UBA1712;s__
ERR1135191_bin.13	d__Bacteria;p__Bacteroidota;c__Bacteroidia;o__Bacteroidales;f__Bacteroidaceae;g__UBA4334;s__UBA4334 sp900316975
ERR1135191_bin.2	d__Bacteria;p__Firmicutes_A;c__Clostridia;o__Lachnospirales;f__Lachnospiraceae;g__SFDP01;s__SFDP01 sp004558185
ERR1135191_bin.22	d__Bacteria;p__Spirochaetota;c__Spirochaetia;o__Sphaerochaetales;f__Sphaerochaetaceae;g__Spiro-01;s__
ERR1135191_bin.23	d__Bacteria;p__Bacteroidota;c__Bacteroidia;o__Bacteroidales;f__Bacteroidaceae;g__Prevotella;s__
ERR1135191_bin.26	d__Bacteria;p__Firmicutes_A;c__Clostridia;o__Oscillospirales;f__Ruminococcaceae;g__CAG-115;s__
ERR1135191_bin.33	d__Bacteria;p__Firmicutes_A;c__Clostridia_A;o__Christensenellales;f__UBA3700;g__UBA3700;s__
ERR1135191_bin.44	d__Bacteria;p__Spirochaetota;c__Spirochaetia;o__Treponematales;f__Treponemataceae;g__Treponema_D;s__
ERR1135191_bin.69	d__Bacteria;p__Firmicutes_A;c__Clostridia_A;o__Christensenellales;f__CAG-314;g__SFEB01;s__SFEB01 sp004558105
ERR1135191_bin.71	d__Bacteria;p__Bacteroidota;c__Bacteroidia;o__Bacteroidales;f__UBA932;g__RC9;s__
ERR1135192_bin.10	d__Bacteria;p__Spirochaetota;c__Spirochaetia;o__Sphaerochaetales;f__Sphaerochaetaceae;g__Spiro-01;s__
ERR1135192_bin.11	d__Bacteria;p__Firmicutes_A;c__Clostridia;o__Oscillospirales;f__Acutalibacteraceae;g__Ruminococcus_E;s__
ERR1135192_bin.18	d__Bacteria;p__Firmicutes_A;c__Clostridia;o__Lachnospirales;f__Lachnospiraceae;g__CAG-95;s__
ERR1135192_bin.2	d__Bacteria;p__Firmicutes_A;c__Clostridia;o__Lachnospirales;f__Lachnospiraceae;g__Dorea;s__
ERR1135192_bin.41	d__Bacteria;p__Bacteroidota;c__Bacteroidia;o__Bacteroidales;f__Bacteroidaceae;g__Bacteroides;s__
ERR1135192_bin.68	d__Bacteria;p__Firmicutes_A;c__Clostridia;o__Lachnospirales;f__Lachnospiraceae;g__Acetatifactor;s__
ERR1135192_bin.7	d__Bacteria;p__Bacteroidota;c__Bacteroidia;o__Bacteroidales;f__Bacteroidaceae;g__UBA6382;s__
ERR1135192_bin.82	d__Bacteria;p__Firmicutes_A;c__Clostridia;o__Peptostreptococcales;f__Anaerovoracaceae;g__;s__
ERR1135192_bin.89	d__Bacteria;p__Firmicutes_A;c__Clostridia_A;o__Christensenellales;f__UBA1242;g__UMGS1470;s__
ERR1135192_bin.90	d__Bacteria;p__Firmicutes_A;c__Clostridia_A;o__Christensenellales;f__Borkfalkiaceae;g__;s__
ERR1135192_bin.94	d__Bacteria;p__Firmicutes_A;c__Clostridia_A;o__Christensenellales;f__CAG-917;g__CAG-475;s__CAG-475 sp900547975
ERR1135193_bin.102	d__Bacteria;p__Proteobacteria;c__Gammaproteobacteria;o__Enterobacterales;f__Succinivibrionaceae;g__Succinivibrio;s__Succinivibrio hippei_B
ERR1135193_bin.27	d__Bacteria;p__Firmicutes_A;c__Clostridia;o__Oscillospirales;f__Acutalibacteraceae;g__CAG-180;s__CAG-180 sp004555265
ERR1135193_bin.58	d__Bacteria;p__Firmicutes;c__Bacilli;o__RF39;f__CAG-302;g__CAG-302;s__
ERR1135193_bin.97	d__Bacteria;p__Campylobacterota;c__Campylobacteria;o__Campylobacterales;f__Campylobacteraceae;g__Campylobacter;s__
ERR1135194_bin.3	d__Bacteria;p__Firmicutes_A;c__Clostridia;o__Oscillospirales;f__Oscillospiraceae;g__F23-B02;s__
ERR1135194_bin.34	d__Bacteria;p__Firmicutes_A;c__Clostridia;o__Oscillospirales;f__Oscillospiraceae;g__UBA1777;s__UBA1777 sp004560305
ERR1135194_bin.39	d__Bacteria;p__Firmicutes_A;c__Clostridia;o__Oscillospirales;f__Acutalibacteraceae;g__Ruminococcus_E;s__Ruminococcus_E sp004560275
ERR1135194_bin.48	d__Bacteria;p__Bacteroidota;c__Bacteroidia;o__Bacteroidales;f__Bacteroidaceae;g__Prevotella;s__Prevotella sp004556065
ERR1135194_bin.5	d__Bacteria;p__Firmicutes_A;c__Clostridia_A;o__Christensenellales;f__UBA1242;g__UMGS687;s__UMGS687 sp900544595
ERR1135194_bin.51	d__Bacteria;p__Spirochaetota;c__Spirochaetia;o__Sphaerochaetales;f__Sphaerochaetaceae;g__UBA5920;s__
ERR1135194_bin.53	d__Bacteria;p__Firmicutes;c__Bacilli;o__Erysipelotrichales;f__Erysipelatoclostridiaceae;g__Sharpea;s__
ERR1135194_bin.56	d__Bacteria;p__Firmicutes_A;c__Clostridia;o__Peptostreptococcales;f__Anaerovoracaceae;g__;s__
ERR1135194_bin.68	d__Bacteria;p__Bacteroidota;c__Bacteroidia;o__Bacteroidales;f__Muribaculaceae;g__C941;s__C941 sp004557565
ERR1135195_bin.12	d__Bacteria;p__Elusimicrobiota;c__Elusimicrobia;o__Elusimicrobiales;f__Elusimicrobiaceae;g__UBA1436;s__
ERR1135195_bin.18	d__Bacteria;p__Bacteroidota;c__Bacteroidia;o__Bacteroidales;f__Bacteroidaceae;g__Prevotella;s__

SGBs ID	Classification
ERR1135195_bin.19	d__Bacteria;p__Firmicutes_A;c__Clostridia_A;o__Christensenellales;f__UBA1242;g__;s__
ERR1135195_bin.36	d__Bacteria;p__Firmicutes_A;c__Clostridia_A;o__Christensenellales;f__CAG-1252;g__;s__
ERR1135195_bin.54	d__Bacteria;p__Bacteroidota;c__Bacteroidia;o__Bacteroidales;f__UBA932;g__RC9;s__
ERR1135195_bin.55	d__Bacteria;p__Bacteroidota;c__Bacteroidia;o__Bacteroidales;f__Bacteroidaceae;g__Phocaeicola;s__
ERR1135195_bin.61	d__Bacteria;p__Spirochaetota;c__Spirochaetia;o__Sphaerochaetales;f__Sphaerochaetaceae;g__UBA5920;s__
ERR1135195_bin.69	d__Bacteria;p__Bacteroidota;c__Bacteroidia;o__Bacteroidales;f__UBA932;g__RC9;s__RC9 sp004554455
ERR1135196_bin.11	d__Bacteria;p__Firmicutes_C;c__Negativicutes;o__Selenomonadales;f__Selenomonadaceae;g__Anaerovibrio;s__Anaerovibrio sp900548165
ERR1135196_bin.23	d__Bacteria;p__Firmicutes_A;c__Clostridia;o__TANB77;f__CAG-508;g__CAG-269;s__CAG-269 sp900551615
ERR1135196_bin.24	d__Bacteria;p__Firmicutes_A;c__Clostridia;o__Lachnospirales;f__Lachnospiraceae;g__Acetatifactor;s__Acetatifactor sp900066365
ERR1135196_bin.29	d__Bacteria;p__Firmicutes_A;c__Clostridia;o__Oscillospirales;f__Acutalibacteraceae;g__UBA737;s__
ERR1135196_bin.4	d__Bacteria;p__Firmicutes_A;c__Clostridia;o__Oscillospirales;f__Acutalibacteraceae;g__Eubacterium_R;s__Eubacterium_R sp900542875
ERR1135196_bin.50	d__Bacteria;p__Firmicutes;c__Bacilli;o__Erysipelotrichales;f__Erysipelotrichaceae;g__Bulleidia;s__
ERR1135196_bin.51	d__Bacteria;p__Cyanobacteria;c__Vampirovibrionia;o__Gastranaerophilales;f__Gastranaerophilaceae;g__Zag111;s__Zag111 sp002103105
ERR1135196_bin.55	d__Bacteria;p__Firmicutes_C;c__Negativicutes;o__Selenomonadales;f__Selenomonadaceae;g__Mitsuokella;s__Mitsuokella multacida
ERR1135196_bin.68	d__Bacteria;p__Bacteroidota;c__Bacteroidia;o__Bacteroidales;f__Muribaculaceae;g__CAG-279;s__CAG-279 sp000437795
ERR1135197_bin.2	d__Bacteria;p__Firmicutes;c__Bacilli;o__RF39;f__CAG-302;g__CAG-302;s__CAG-302 sp900548425
ERR1135197_bin.22	d__Bacteria;p__Bacteroidota;c__Bacteroidia;o__Bacteroidales;f__UBA932;g__RC9;s__RC9 sp004552115
ERR1135197_bin.34	d__Bacteria;p__Firmicutes_A;c__Clostridia;o__Lachnospirales;f__Lachnospiraceae;g__Agathobacter;s__
ERR1135197_bin.36	d__Bacteria;p__Firmicutes_A;c__Clostridia;o__Oscillospirales;f__Acutalibacteraceae;g__Eubacterium_R;s__
ERR1135197_bin.41	d__Bacteria;p__Bacteroidota;c__Bacteroidia;o__Bacteroidales;f__Paludibacteraceae;g__RF16;s__
ERR1135197_bin.53	d__Bacteria;p__Firmicutes_A;c__Clostridia;o__Lachnospirales;f__Lachnospiraceae;g__Agathobacter;s__Agathobacter sp900549895
ERR1135197_bin.71	d__Bacteria;p__Firmicutes_A;c__Clostridia;o__Oscillospirales;f__Ruminococcaceae;g__Ruminococcus;s__Ruminococcus sp002438605
ERR1135197_bin.78	d__Bacteria;p__Bacteroidota;c__Bacteroidia;o__Bacteroidales;f__UBA932;g__RC9;s__
ERR1135198_bin.11	d__Bacteria;p__Bacteroidota;c__Bacteroidia;o__Bacteroidales;f__Bacteroidaceae;g__Prevotella;s__
ERR1135198_bin.37	d__Bacteria;p__Spirochaetota;c__Spirochaetia;o__Treponematales;f__Treponemataceae;g__Treponema_D;s__Treponema_D porcinum
ERR1135198_bin.40	d__Bacteria;p__Spirochaetota;c__Spirochaetia;o__Sphaerochaetales;f__Sphaerochaetaceae;g__UBA5920;s__
ERR1135198_bin.54	d__Bacteria;p__Firmicutes;c__Bacilli;o__RFN20;f__CAG-449;g__UBA3210;s__
ERR1135198_bin.73	d__Bacteria;p__Bacteroidota;c__Bacteroidia;o__Bacteroidales;f__Muribaculaceae;g__CAG-279;s__CAG-279 sp004555955
ERR1135198_bin.75	d__Bacteria;p__Firmicutes_A;c__Clostridia_A;o__Christensenellales;f__UBA1242;g__UBA7862;s__
ERR1135198_bin.77	d__Bacteria;p__Bacteroidota;c__Bacteroidia;o__Bacteroidales;f__Muribaculaceae;g__CAG-873;s__
ERR1135199_bin.23	d__Bacteria;p__Firmicutes_A;c__Clostridia_A;o__Christensenellales;f__CAG-917;g__CAG-475;s__CAG-475 sp000434435
ERR1135199_bin.3	d__Bacteria;p__Firmicutes;c__Bacilli;o__RF39;f__CAG-302;g__CAG-302;s__CAG-302 sp900543825
ERR1135199_bin.45	d__Bacteria;p__Firmicutes_A;c__Clostridia;o__Peptostreptococcales;f__Anaerovoracaceae;g__CAG-145;s__CAG-145 sp002320005
ERR1135199_bin.55	d__Bacteria;p__Firmicutes;c__Bacilli;o__RF39;f__CAG-611;g__CAG-710;s__CAG-710 sp000432595
ERR1135199_bin.61	d__Bacteria;p__Firmicutes_A;c__Clostridia_A;o__Christensenellales;f__CAG-138;g__PeH17;s__PeH17 sp000435055
ERR1135199_bin.64	d__Bacteria;p__Firmicutes_A;c__Clostridia;o__Oscillospirales;f__Acutalibacteraceae;g__CAG-488;s__
ERR1135199_bin.71	d__Bacteria;p__Bacteroidota;c__Bacteroidia;o__Bacteroidales;f__UBA932;g__RC9;s__RC9 sp004552615
ERR1135199_bin.72	d__Bacteria;p__Spirochaetota;c__Spirochaetia;o__Treponematales;f__Treponemataceae;g__Treponema_D;s__
ERR1135200_bin.105	d__Bacteria;p__Firmicutes_A;c__Clostridia;o__Oscillospirales;f__Ruminococcaceae;g__;s__
ERR1135200_bin.11	d__Bacteria;p__Firmicutes_A;c__Clostridia;o__Oscillospirales;f__CAG-272;g__UBA1380;s__
ERR1135200_bin.12	d__Bacteria;p__Bacteroidota;c__Bacteroidia;o__Bacteroidales;f__Bacteroidaceae;g__Prevotellamassilia;s__Prevotellamassilia sp004556745
ERR1135200_bin.22	d__Bacteria;p__Firmicutes_A;c__Clostridia;o__Oscillospirales;f__Oscillospiraceae;g__UBA1777;s__
ERR1135200_bin.44	d__Bacteria;p__Bacteroidota;c__Bacteroidia;o__Bacteroidales;f__Bacteroidaceae;g__Prevotella;s__Prevotella sp900546535
ERR1135200_bin.65	d__Bacteria;p__Firmicutes_A;c__Clostridia;o__Lachnospirales;f__Lachnospiraceae;g__UBA2868;s__UBA2868 sp004552595
ERR1135200_bin.73	d__Bacteria;p__Firmicutes_A;c__Clostridia;o__Oscillospirales;f__Ruminococcaceae;g__CAG-115;s__
ERR1135200_bin.85	d__Bacteria;p__Firmicutes_A;c__Clostridia;o__Oscillospirales;f__Ruminococcaceae;g__HUN007;s__
ERR1135200_bin.9	d__Bacteria;p__Cyanobacteria;c__Vampirovibrionia;o__Gastranaerophilales;f__Gastranaerophilaceae;g__;s__
ERR1135201_bin.12	d__Bacteria;p__Bacteroidota;c__Bacteroidia;o__Bacteroidales;f__Muribaculaceae;g__;s__
ERR1135201_bin.17	d__Bacteria;p__Firmicutes_A;c__Clostridia;o__Oscillospirales;f__Oscillospiraceae;g__CAG-110;s__
ERR1135201_bin.34	d__Bacteria;p__Firmicutes_A;c__Clostridia;o__Oscillospirales;f__CAG-382;g__UMGS1052;s__UMGS1052 sp900548115
ERR1135201_bin.47	d__Bacteria;p__Spirochaetota;c__Spirochaetia;o__Sphaerochaetales;f__Sphaerochaetaceae;g__Spiro-01;s__
ERR1135201_bin.67	d__Bacteria;p__Cyanobacteria;c__Vampirovibrionia;o__Gastranaerophilales;f__Gastranaerophilaceae;g__CAG-196;s__CAG-196 sp900549855
ERR1135201_bin.70	d__Bacteria;p__Firmicutes_A;c__Clostridia_A;o__Christensenellales;f__Borkfalkiaceae;g__;s__
ERR1135201_bin.79	d__Bacteria;p__Firmicutes_A;c__Clostridia;o__Lachnospirales;f__Lachnospiraceae;g__;s__
ERR1135201_bin.83	d__Bacteria;p__Bacteroidota;c__Bacteroidia;o__Bacteroidales;f__UBA932;g__UBA1232;s__UBA1232 sp004561775
ERR1135201_bin.84	d__Bacteria;p__Firmicutes_A;c__Clostridia_A;o__Christensenellales;f__UBA3700;g__UBA3700;s__
ERR1135201_bin.86	d__Bacteria;p__Firmicutes;c__Bacilli;o__RF39;f__CAG-433;g__CAG-594;s__CAG-594 sp000434835
ERR1135201_bin.99	d__Bacteria;p__Firmicutes_A;c__Clostridia;o__Oscillospirales;f__CAG-272;g__UBA1380;s__
ERR1135202_bin.1	d__Bacteria;p__Firmicutes_A;c__Clostridia;o__Oscillospirales;f__Acutalibacteraceae;g__CAG-177;s__
ERR1135202_bin.10	d__Bacteria;p__Bacteroidota;c__Bacteroidia;o__Bacteroidales;f__UBA932;g__RC9;s__
ERR1135202_bin.14	d__Bacteria;p__Firmicutes_A;c__Clostridia;o__Oscillospirales;f__Acutalibacteraceae;g__UBA737;s__
ERR1135202_bin.31	d__Bacteria;p__Firmicutes_A;c__Clostridia;o__Oscillospirales;f__Oscillospiraceae;g__UBA1777;s__
ERR1135202_bin.56	d__Bacteria;p__Firmicutes_A;c__Clostridia_A;o__Christensenellales;f__Borkfalkiaceae;g__UBA7597;s__
ERR1135202_bin.68	d__Bacteria;p__Firmicutes_A;c__Clostridia_A;o__Christensenellales;f__Borkfalkiaceae;g__;s__
ERR1135202_bin.70	d__Bacteria;p__Bacteroidota;c__Bacteroidia;o__Bacteroidales;f__Muribaculaceae;g__C941;s__C941 sp004559845
ERR1135203_bin.24	d__Bacteria;p__Spirochaetota;c__Spirochaetia;o__Treponematales;f__Treponemataceae;g__Treponema_D;s__
ERR1135203_bin.44	d__Bacteria;p__Bacteroidota;c__Bacteroidia;o__Bacteroidales;f__UBA932;g__RC9;s__
ERR1135204_bin.18	d__Bacteria;p__Firmicutes_C;c__Negativicutes;o__Acidaminococcales;f__Acidaminococcaceae;g__Phascolarctobacterium_A;s__Phascolarctobacterium_A sp900553055
ERR1135204_bin.75	d__Bacteria;p__Firmicutes_A;c__Clostridia;o__Lachnospirales;f__Lachnospiraceae;g__UBA3766;s__
ERR1135204_bin.76	d__Bacteria;p__Bacteroidota;c__Bacteroidia;o__Bacteroidales;f__UBA932;g__RC9;s__
ERR1135205_bin.17	d__Bacteria;p__Firmicutes_A;c__Clostridia;o__Oscillospirales;f__Acutalibacteraceae;g__Ruminococcus_H;s__Ruminococcus_H sp003531055
ERR1135205_bin.43	d__Bacteria;p__Bacteroidota;c__Bacteroidia;o__Bacteroidales;f__Tannerellaceae;g__Parabacteroides;s__
ERR1135205_bin.45	d__Bacteria;p__Bacteroidota;c__Bacteroidia;o__Bacteroidales;f__Bacteroidaceae;g__Prevotella;s__
ERR1135205_bin.52	d__Bacteria;p__Bacteroidota;c__Bacteroidia;o__Bacteroidales;f__Bacteroidaceae;g__Prevotella;s__
ERR1135205_bin.60	d__Bacteria;p__Bacteroidota;c__Bacteroidia;o__Bacteroidales;f__UBA932;g__RC9;s__RC9 sp000434935
ERR1135205_bin.8	d__Bacteria;p__Firmicutes_C;c__Negativicutes;o__Acidaminococcales;f__Acidaminococcaceae;g__Phascolarctobacterium_A;s__
ERR1135206_bin.63	d__Bacteria;p__Firmicutes_A;c__Clostridia_A;o__Christensenellales;f__CAG-917;g__CAG-349;s__CAG-349 sp001940895
ERR1135206_bin.69	d__Bacteria;p__Firmicutes_A;c__Clostridia_A;o__Christensenellales;f__CAG-917;g__CAG-475;s__CAG-475 sp900551145
ERR1135207_bin.59	d__Bacteria;p__Proteobacteria;c__Alphaproteobacteria;o__RF32;f__CAG-239;g__RUG410;s__RUG410 sp900313185
ERR1135208_bin.18	d__Bacteria;p__Spirochaetota;c__Spirochaetia;o__Treponematales;f__Treponemataceae;g__Treponema_D;s__
ERR1135208_bin.20	d__Bacteria;p__Firmicutes_A;c__Clostridia;o__Oscillospirales;f__Oscillospiraceae;g__CAG-110;s__
ERR1135208_bin.40	d__Bacteria;p__Firmicutes_A;c__Clostridia_A;o__Christensenellales;f__Borkfalkiaceae;g__;s__
ERR1135208_bin.43	d__Bacteria;p__Firmicutes_A;c__Clostridia;o__Oscillospirales;f__Acutalibacteraceae;g__Eubacterium_R;s__
ERR1135208_bin.72	d__Bacteria;p__Bacteroidota;c__Bacteroidia;o__Bacteroidales;f__UBA932;g__RC9;s__
ERR1135209_bin.24	d__Bacteria;p__Firmicutes_A;c__Clostridia;o__Oscillospirales;f__Ruminococcaceae;g__Ruminiclostridium_E;s__
ERR1135209_bin.28	d__Bacteria;p__Firmicutes_A;c__Clostridia;o__Oscillospirales;f__Oscillospiraceae;g__F23-B02;s__F23-B02 sp004556755
ERR1135209_bin.29	d__Bacteria;p__Bacteroidota;c__Bacteroidia;o__Bacteroidales;f__Bacteroidaceae;g__UBA4372;s__
ERR1135209_bin.42	d__Bacteria;p__Bacteroidota;c__Bacteroidia;o__Bacteroidales;f__Bacteroidaceae;g__Prevotella;s__
ERR1135209_bin.8	d__Bacteria;p__Bacteroidota;c__Bacteroidia;o__Bacteroidales;f__UBA932;g__RC9;s__

SGBs ID	Classification
ERR1135210_bin.33	d__Bacteria;p__Bacteroidota;c__Bacteroidia;o__Bacteroidales;f__UBA932;g__CAG-831;s__
ERR1135210_bin.54	d__Bacteria;p__Firmicutes_A;c__Clostridia;o__Lachnospirales;f__Lachnospiraceae;g__CAG-95;s__
ERR1135211_bin.21	d__Bacteria;p__Firmicutes_A;c__Clostridia;o__Oscillospirales;f__Acutalibacteraceae;g__UBA6857;s__
ERR1135211_bin.33	d__Bacteria;p__Firmicutes_A;c__Clostridia;o__Lachnospirales;f__Lachnospiraceae;g__NK4A136;s__
ERR1135212_bin.14	d__Bacteria;p__Firmicutes_A;c__Clostridia;o__Oscillospirales;f__CAG-272;g__UMGS1865;s__
ERR1135212_bin.26	d__Bacteria;p__Firmicutes_A;c__Clostridia;o__Oscillospirales;f__Oscillospiraceae;g__CAG-110;s__
ERR1135212_bin.6	d__Bacteria;p__Bacteroidota;c__Bacteroidia;o__Bacteroidales;f__Paludibacteraceae;g__RF16;s__
ERR1135212_bin.70	d__Bacteria;p__Firmicutes_A;c__Clostridia;o__Oscillospirales;f__Ruminococcaceae;g__CAG-115;s__
ERR1135212_bin.78	d__Bacteria;p__Bacteroidota;c__Bacteroidia;o__Bacteroidales;f__Rikenellaceae;g__Alistipes;s__
ERR1135213_bin.14	d__Archaea;p__Methanobacteriota;c__Methanobacteria;o__Methanobacteriales;f__Methanobacteriaceae;g__Methanobrevibacter_A;s__
ERR1135213_bin.44	d__Bacteria;p__Bacteroidota;c__Bacteroidia;o__Bacteroidales;f__Bacteroidaceae;g__Prevotella;s__Prevotella sp004555245
ERR1135213_bin.46	d__Bacteria;p__Firmicutes_A;c__Clostridia;o__Lachnospirales;f__Lachnospiraceae;g__;s__
ERR1135213_bin.84	d__Bacteria;p__Bacteroidota;c__Bacteroidia;o__Bacteroidales;f__Muribaculaceae;g__CAG-485;s__CAG-485 sp004553095
ERR1135213_bin.9	d__Bacteria;p__Firmicutes_A;c__Clostridia;o__Lachnospirales;f__Lachnospiraceae;g__CAG-510;s__
ERR1135213_bin.96	d__Bacteria;p__Bacteroidota;c__Bacteroidia;o__Bacteroidales;f__Muribaculaceae;g__C941;s__
ERR1135214_bin.3	d__Bacteria;p__Firmicutes_A;c__Clostridia;o__Oscillospirales;f__Acutalibacteraceae;g__CAG-488;s__CAG-488 sp000434055
ERR1135214_bin.8	d__Bacteria;p__Firmicutes_A;c__Clostridia;o__Oscillospirales;f__CAG-272;g__CAG-841;s__CAG-841 sp002479075
ERR1135215_bin.21	d__Bacteria;p__Firmicutes_A;c__Clostridia_A;o__Christensenellales;f__CAG-138;g__RUG472;s__RUG472 sp900545265
ERR1135215_bin.23	d__Bacteria;p__Firmicutes_A;c__Clostridia;o__Oscillospirales;f__Oscillospiraceae;g__UBA1777;s__UBA1777 sp900549645
ERR1135215_bin.52	d__Bacteria;p__Bacteroidota;c__Bacteroidia;o__Bacteroidales;f__Bacteroidaceae;g__Prevotella;s__
ERR1135216_bin.19	d__Bacteria;p__Bacteroidota;c__Bacteroidia;o__Bacteroidales;f__Bacteroidaceae;g__Prevotella;s__Prevotella sp002299635
ERR1135216_bin.4	d__Bacteria;p__Firmicutes_A;c__Clostridia;o__Oscillospirales;f__Oscillospiraceae;g__F23-B02;s__F23-B02 sp002472405
ERR1135216_bin.5	d__Bacteria;p__Firmicutes;c__Bacilli;o__RF39;f__CAG-1000;g__CAG-533;s__CAG-533 sp900553855
ERR1135216_bin.67	d__Bacteria;p__Firmicutes;c__Bacilli;o__RFN20;f__CAG-288;g__UBA7642;s__UBA7642 sp900549915
ERR1135217_bin.14	d__Bacteria;p__Bacteroidota;c__Bacteroidia;o__Bacteroidales;f__UBA932;g__RC9;s__RC9 sp000431015
ERR1135217_bin.5	d__Bacteria;p__Firmicutes_A;c__Clostridia;o__Oscillospirales;f__Oscillospiraceae;g__CAG-110;s__
ERR1135217_bin.57	d__Bacteria;p__Bacteroidota;c__Bacteroidia;o__Bacteroidales;f__Bacteroidaceae;g__Prevotella;s__Prevotella sp000434975
ERR1135217_bin.65	d__Bacteria;p__Cyanobacteria;c__Vampirovibrionia;o__Gastranaerophilales;f__Gastranaerophilaceae;g__Zag111;s__Zag111 sp003258735
ERR1135218_bin.14	d__Bacteria;p__Firmicutes_A;c__Clostridia;o__Oscillospirales;f__Oscillospiraceae;g__CAG-110;s__
ERR1135218_bin.58	d__Bacteria;p__Cyanobacteria;c__Vampirovibrionia;o__Gastranaerophilales;f__Gastranaerophilaceae;g__Zag111;s__Zag111 sp002102825
ERR1135219_bin.28	d__Bacteria;p__Bacteroidota;c__Bacteroidia;o__Bacteroidales;f__UBA932;g__RC9;s__RC9 sp900546445
ERR1135219_bin.32	d__Bacteria;p__Firmicutes_A;c__Clostridia;o__Oscillospirales;f__CAG-272;g__CAG-724;s__CAG-724 sp003524145
ERR1135219_bin.41	d__Bacteria;p__Firmicutes_A;c__Clostridia_A;o__Christensenellales;f__CAG-138;g__Phil1;s__Phil1 sp001940855
ERR1135219_bin.51	d__Bacteria;p__Firmicutes;c__Bacilli;o__RFN20;f__CAG-826;g__UBA733;s__UBA733 sp900550795
ERR1135219_bin.61	d__Bacteria;p__Bacteroidota;c__Bacteroidia;o__Bacteroidales;f__Muribaculaceae;g__CAG-873;s__CAG-873 sp001701165
ERR1135219_bin.8	d__Bacteria;p__Campylobacterota;c__Campylobacteria;o__Campylobacterales;f__Campylobacteraceae;g__Campylobacter;s__Campylobacter lanienae
ERR1135220_bin.1	d__Bacteria;p__Verrucomicrobiota_A;c__Chlamydiia;o__Chlamydiales;f__Chlamydiaceae;g__Chlamydia;s__Chlamydia suis
ERR1135220_bin.65	d__Bacteria;p__Firmicutes_A;c__Clostridia;o__Lachnospirales;f__Lachnospiraceae;g__SFDP01;s__
ERR1135220_bin.68	d__Bacteria;p__Campylobacterota;c__Campylobacteria;o__Campylobacterales;f__Helicobacteraceae;g__Helicobacter_F;s__Helicobacter_F sp002287135
ERR1135220_bin.70	d__Bacteria;p__Firmicutes;c__Bacilli;o__RF39;f__CAG-302;g__CAG-914;s__CAG-914 sp000437895
ERR1135221_bin.12	d__Bacteria;p__Bacteroidota;c__Bacteroidia;o__Bacteroidales;f__Bacteroidaceae;g__Prevotella;s__Prevotella sp000436035
ERR1135221_bin.24	d__Bacteria;p__Firmicutes_C;c__Negativicutes;o__Selenomonadales;f__Selenomonadaceae;g__Megamonas;s__Megamonas funiformis
ERR1135221_bin.29	d__Bacteria;p__Bacteroidota;c__Bacteroidia;o__Bacteroidales;f__Bacteroidaceae;g__Prevotella;s__Prevotella sp900322035
ERR1135222_bin.9	d__Bacteria;p__Bacteroidota;c__Bacteroidia;o__Bacteroidales;f__Bacteroidaceae;g__Prevotella;s__Prevotella sp002480935
ERR1135223_bin.20	d__Bacteria;p__Firmicutes;c__Bacilli;o__Erysipelotrichales;f__Erysipelotrichaceae;g__Bulleidia;s__Bulleidia timonensis
ERR1135223_bin.22	d__Bacteria;p__Bacteroidota;c__Bacteroidia;o__Bacteroidales;f__Bacteroidaceae;g__Prevotella;s__
ERR1135223_bin.52	d__Bacteria;p__Firmicutes_C;c__Negativicutes;o__Acidaminococcales;f__Acidaminococcaceae;g__Acidaminococcus;s__Acidaminococcus fermentans
ERR1135224_bin.55	d__Bacteria;p__Firmicutes_A;c__Clostridia;o__TANB77;f__CAG-508;g__CAG-273;s__CAG-273 sp003534295
ERR1135224_bin.9	d__Bacteria;p__Firmicutes_A;c__Clostridia;o__Oscillospirales;f__Oscillospiraceae;g__CAG-170;s__CAG-170 sp003516765
ERR1135225_bin.13	d__Bacteria;p__Firmicutes_A;c__Clostridia;o__Oscillospirales;f__Ruminococcaceae;g__Faecalibacterium;s__Faecalibacterium prausnitzii_E
ERR1135225_bin.24	d__Bacteria;p__Firmicutes_A;c__Clostridia;o__Lachnospirales;f__Lachnospiraceae;g__Weimerbacter;s__Weimerbacter sp900316165
ERR1135226_bin.23	d__Bacteria;p__Firmicutes;c__Bacilli;o__Erysipelotrichales;f__Erysipelotrichaceae;g__Holdemanella;s__Holdemanella sp002299315
ERR1135227_bin.18	d__Bacteria;p__Firmicutes_C;c__Negativicutes;o__Selenomonadales;f__Selenomonadaceae;g__Selenomonas_C;s__Selenomonas_C sp900318215
ERR1135227_bin.55	d__Bacteria;p__Firmicutes_A;c__Clostridia;o__Oscillospirales;f__Acutalibacteraceae;g__GCA-900066995;s__
ERR1135227_bin.69	d__Bacteria;p__Bacteroidota;c__Bacteroidia;o__Bacteroidales;f__Bacteroidaceae;g__Prevotellamassilia;s__Prevotellamassilia sp900540885
ERR1135229_bin.55	d__Bacteria;p__Firmicutes;c__Bacilli;o__RFN20;f__CAG-288;g__;s__
ERR1135230_bin.14	d__Bacteria;p__Firmicutes_A;c__Clostridia;o__Lachnospirales;f__Lachnospiraceae;g__Eubacterium_F;s__
ERR1135231_bin.4	d__Bacteria;p__Firmicutes;c__Bacilli;o__RF39;f__CAG-611;g__UBA3789;s__UBA3789 sp900543055
ERR1135232_bin.13	d__Bacteria;p__Bacteroidota;c__Bacteroidia;o__Bacteroidales;f__Bacteroidaceae;g__Prevotella;s__
ERR1135232_bin.36	d__Bacteria;p__Bacteroidota;c__Bacteroidia;o__Bacteroidales;f__Bacteroidaceae;g__Prevotella;s__Prevotella sp004554665
ERR1135233_bin.32	d__Bacteria;p__Firmicutes_A;c__Clostridia;o__Lachnospirales;f__Lachnospiraceae;g__Acetatifactor;s__
ERR1135233_bin.45	d__Bacteria;p__Deferribacterota;c__Deferribacteres;o__Deferribacterales;f__Mucispirillaceae;g__Mucispirillum;s__
ERR1135235_bin.13	d__Bacteria;p__Firmicutes_A;c__Clostridia;o__Lachnospirales;f__Lachnospiraceae;g__TWA4;s__
ERR1135235_bin.60	d__Bacteria;p__Firmicutes_A;c__Clostridia;o__Oscillospirales;f__Ruminococcaceae;g__Ruminococcus;s__Ruminococcus flavefaciens_S
ERR1135239_bin.54	d__Bacteria;p__Firmicutes_A;c__Clostridia;o__Monoglobales;f__Firm-18;g__UBA1775;s__
ERR1135241_bin.22	d__Bacteria;p__Firmicutes_A;c__Clostridia;o__Oscillospirales;f__Acutalibacteraceae;g__CAG-180;s__
ERR1135241_bin.24	d__Bacteria;p__Firmicutes;c__Bacilli;o__Erysipelotrichales;f__Erysipelotrichaceae;g__Bulleidia;s__
ERR1135241_bin.6	d__Bacteria;p__Firmicutes_A;c__Clostridia;o__Lachnospirales;f__Lachnospiraceae;g__CAG-791;s__CAG-791 sp900318875
ERR1135241_bin.71	d__Bacteria;p__Firmicutes_A;c__Clostridia;o__Lachnospirales;f__Lachnospiraceae;g__Weimerbacter;s__Weimerbacter sp900315505
ERR1135242_bin.36	d__Bacteria;p__Bacteroidota;c__Bacteroidia;o__Bacteroidales;f__Bacteroidaceae;g__Prevotella;s__Prevotella sp900313215
ERR1135245_bin.26	d__Bacteria;p__Bacteroidota;c__Bacteroidia;o__Bacteroidales;f__UBA932;g__RC9;s__RC9 sp000432655
ERR1135245_bin.30	d__Bacteria;p__Spirochaetota;c__Spirochaetia;o__Treponematales;f__Treponemataceae;g__Treponema_F;s__
ERR1135245_bin.31	d__Bacteria;p__Spirochaetota;c__Spirochaetia;o__Treponematales;f__Treponemataceae;g__Treponema_D;s__Treponema_D succinifaciens
ERR1135246_bin.1	d__Bacteria;p__Bacteroidota;c__Bacteroidia;o__Bacteroidales;f__Bacteroidaceae;g__Prevotella;s__Prevotella sp900543585
ERR1135246_bin.54	d__Bacteria;p__Firmicutes_A;c__Clostridia;o__Lachnospirales;f__Lachnospiraceae;g__CAG-510;s__CAG-510 sp000434615
ERR1135247_bin.31	d__Bacteria;p__Proteobacteria;c__Gammaproteobacteria;o__Enterobacterales;f__Succinivibrionaceae;g__Succinivibrio;s__Succinivibrio sp000431835
ERR1135247_bin.55	d__Bacteria;p__Firmicutes_C;c__Negativicutes;o__Selenomonadales;f__Selenomonadaceae;g__Selenomonas_A;s__
ERR1135248_bin.59	d__Bacteria;p__Firmicutes_A;c__Clostridia;o__Oscillospirales;f__Oscillospiraceae;g__F23-B02;s__F23-B02 sp004553495
ERR1135250_bin.5	d__Bacteria;p__Bacteroidota;c__Bacteroidia;o__Bacteroidales;f__Bacteroidaceae;g__Prevotella;s__Prevotella sp000434515
ERR1135251_bin.5	d__Bacteria;p__Bacteroidota;c__Bacteroidia;o__Bacteroidales;f__Paludibacteraceae;g__SFVR01;s__
ERR1135252_bin.17	d__Bacteria;p__Firmicutes_A;c__Clostridia;o__Oscillospirales;f__Oscillospiraceae;g__CAG-110;s__
ERR1135252_bin.27	d__Bacteria;p__Bacteroidota;c__Bacteroidia;o__Bacteroidales;f__Paludibacteraceae;g__RF16;s__
ERR1135252_bin.29	d__Bacteria;p__Firmicutes_A;c__Clostridia;o__Oscillospirales;f__Ruminococcaceae;g__Ruminococcus_F;s__Ruminococcus_F sp004558835
ERR1135252_bin.35	d__Bacteria;p__Firmicutes_A;c__Clostridia;o__Peptostreptococcales;f__Anaerovoracaceae;g__;s__
ERR1135252_bin.62	d__Bacteria;p__Firmicutes_A;c__Clostridia_A;o__Christensenellales;f__CAG-138;g__SFDB01;s__SFDB01 sp004558825
ERR1135253_bin.12	d__Bacteria;p__Bacteroidota;c__Bacteroidia;o__Bacteroidales;f__Paludibacteraceae;g__RF16;s__
ERR1135253_bin.21	d__Bacteria;p__Spirochaetota;c__Spirochaetia;o__Treponematales;f__Treponemataceae;g__Treponema_D;s__
ERR1135254_bin.35	d__Bacteria;p__Firmicutes_A;c__Clostridia;o__Oscillospirales;f__Acutalibacteraceae;g__CAG-180;s__CAG-180 sp004556705
ERR1135254_bin.37	d__Bacteria;p__Spirochaetota;c__Spirochaetia;o__Sphaerochaetales;f__Sphaerochaetaceae;g__UBA5920;s__

SGBs ID	Classification
ERR1135254_bin.65	d__Bacteria;p__Firmicutes;c__Bacilli;o__Lactobacillales;f__Streptococcaceae;g__Streptococcus;s__Streptococcus hyointestinalis
ERR1135256_bin.12	d__Bacteria;p__Bacteroidota;c__Bacteroidia;o__Bacteroidales;f__Bacteroidaceae;g__Prevotella;s__Prevotella sp002299275
ERR1135256_bin.53	d__Bacteria;p__Firmicutes;c__Bacilli;o__Erysipelotrichales;f__Erysipelotrichaceae;g__Bulleidia;s__
ERR1135256_bin.60	d__Bacteria;p__Firmicutes;c__Bacilli;o__RFN20;f__CAG-826;g__UBA4855;s__
ERR1135257_bin.51	d__Bacteria;p__Firmicutes_A;c__Clostridia;o__Lachnospirales;f__Lachnospiraceae;g__Eubacterium_F;s__Eubacterium_F sp003491505
ERR1135257_bin.52	d__Bacteria;p__Proteobacteria;c__Gammaproteobacteria;o__Enterobacterales;f__Succinivibrionaceae;g__Succinivibrio;s__Succinivibrio sp003456415
ERR1135258_bin.21	d__Bacteria;p__Spirochaetota;c__Spirochaetia;o__Treponematales;f__Treponemataceae;g__Treponema_D;s__
ERR1135259_bin.12	d__Bacteria;p__Bacteroidota;c__Bacteroidia;o__Bacteroidales;f__Bacteroidaceae;g__UBA4372;s__
ERR1135259_bin.56	d__Bacteria;p__Firmicutes_A;c__Clostridia;o__Oscillospirales;f__Oscillospiraceae;g__CAG-110;s__
ERR1135260_bin.22	d__Bacteria;p__Bacteroidota;c__Bacteroidia;o__Bacteroidales;f__Bacteroidaceae;g__Prevotella;s__Prevotella sp002251365
ERR1135260_bin.40	d__Bacteria;p__Bacteroidota;c__Bacteroidia;o__Bacteroidales;f__UBA932;g__RC9;s__
ERR1135260_bin.45	d__Bacteria;p__Bacteroidota;c__Bacteroidia;o__Bacteroidales;f__Muribaculaceae;g__CAG-873;s__
ERR1135260_bin.46	d__Bacteria;p__Bacteroidota;c__Bacteroidia;o__Bacteroidales;f__Muribaculaceae;g__SFTJ01;s__SFTJ01 sp004563195
ERR1135261_bin.34	d__Bacteria;p__Bacteroidota;c__Bacteroidia;o__Bacteroidales;f__Bacteroidaceae;g__Prevotella;s__
ERR1135262_bin.1	d__Bacteria;p__Firmicutes_A;c__Clostridia;o__Lachnospirales;f__Lachnospiraceae;g__CAG-127;s__
ERR1135262_bin.42	d__Bacteria;p__Bacteroidota;c__Bacteroidia;o__Bacteroidales;f__Bacteroidaceae;g__UBA4334;s__
ERR1135262_bin.43	d__Bacteria;p__Firmicutes_A;c__Clostridia;o__Lachnospirales;f__Lachnospiraceae;g__Agathobacter;s__
ERR1135262_bin.62	d__Bacteria;p__Cyanobacteria;c__Vampirovibrionia;o__Gastranaerophilales;f__Gastranaerophilaceae;g__UBA2883;s__
ERR1135263_bin.3	d__Bacteria;p__Bacteroidota;c__Bacteroidia;o__Bacteroidales;f__Bacteroidaceae;g__Prevotella;s__
ERR1135263_bin.42	d__Bacteria;p__Firmicutes_A;c__Clostridia;o__Lachnospirales;f__Lachnospiraceae;g__Eubacterium_Q;s__Eubacterium_Q sp900314445
ERR1135264_bin.17	d__Bacteria;p__Bacteroidota;c__Bacteroidia;o__Bacteroidales;f__Paludibacteraceae;g__RF16;s__
ERR1135264_bin.18	d__Bacteria;p__Firmicutes_A;c__Clostridia;o__Lachnospirales;f__Lachnospiraceae;g__Agathobacter;s__
ERR1135264_bin.32	d__Bacteria;p__Firmicutes_A;c__Clostridia;o__Lachnospirales;f__Lachnospiraceae;g__CAG-194;s__CAG-194 sp000432915
ERR1135265_bin.18	d__Bacteria;p__Firmicutes_C;c__Negativicutes;o__Selenomonadales;f__Selenomonadaceae;g__Anaerovibrio;s__Anaerovibrio sp004553105
ERR1135265_bin.33	d__Bacteria;p__Firmicutes_A;c__Clostridia;o__Oscillospirales;f__Oscillospiraceae;g__CAG-110;s__
ERR1135265_bin.44	d__Bacteria;p__Firmicutes_A;c__Clostridia;o__Lachnospirales;f__Lachnospiraceae;g__Agathobacter;s__
ERR1135266_bin.18	d__Bacteria;p__Proteobacteria;c__Alphaproteobacteria;o__RF32;f__CAG-239;g__RUG410;s__
ERR1135266_bin.21	d__Bacteria;p__Bacteroidota;c__Bacteroidia;o__Bacteroidales;f__Bacteroidaceae;g__Prevotella;s__
ERR1135266_bin.58	d__Bacteria;p__Bacteroidota;c__Bacteroidia;o__Bacteroidales;f__Bacteroidaceae;g__Prevotella;s__Prevotella sp000834015
ERR1135266_bin.67	d__Bacteria;p__Firmicutes;c__Bacilli;o__Erysipelotrichales;f__Erysipelotrichaceae;g__UBA636;s__UBA636 sp900546285
ERR1135267_bin.18	d__Bacteria;p__Firmicutes_A;c__Clostridia;o__Lachnospirales;f__Lachnospiraceae;g__Agathobacter;s__
ERR1135267_bin.21	d__Bacteria;p__Bacteroidota;c__Bacteroidia;o__Bacteroidales;f__Paludibacteraceae;g__RF16;s__
ERR1135267_bin.30	d__Bacteria;p__Bacteroidota;c__Bacteroidia;o__Bacteroidales;f__Bacteroidaceae;g__Prevotella;s__Prevotella sp004557285
ERR1135267_bin.37	d__Bacteria;p__Bacteroidota;c__Bacteroidia;o__Bacteroidales;f__Bacteroidaceae;g__Prevotella;s__Prevotella sp900548195
ERR1135268_bin.39	d__Bacteria;p__Firmicutes_A;c__Clostridia;o__Lachnospirales;f__Lachnospiraceae;g__Eubacterium_H;s__Eubacterium_H sp002394235
ERR1135269_bin.18	d__Bacteria;p__Firmicutes_A;c__Clostridia;o__Oscillospirales;f__Acutalibacteraceae;g__CAG-177;s__CAG-177 sp003514385
ERR1135269_bin.35	d__Bacteria;p__Firmicutes_A;c__Clostridia;o__TANB77;f__CAG-508;g__CAG-273;s__CAG-273 sp000437855
ERR1135270_bin.3	d__Bacteria;p__Firmicutes_A;c__Clostridia;o__Lachnospirales;f__Lachnospiraceae;g__Agathobacter;s__
ERR1135270_bin.77	d__Bacteria;p__Firmicutes_A;c__Clostridia;o__Lachnospirales;f__Lachnospiraceae;g__UBA11957;s__
ERR1135272_bin.36	d__Bacteria;p__Firmicutes_A;c__Clostridia;o__Lachnospirales;f__Lachnospiraceae;g__Lachnospira;s__Lachnospira sp003537285
ERR1135272_bin.44	d__Bacteria;p__Firmicutes_A;c__Clostridia;o__Lachnospirales;f__Lachnospiraceae;g__Butyrivibrio_A;s__
ERR1135272_bin.51	d__Bacteria;p__Bacteroidota;c__Bacteroidia;o__Bacteroidales;f__Bacteroidaceae;g__Prevotella;s__
ERR1135273_bin.2	d__Bacteria;p__Firmicutes_A;c__Clostridia;o__Lachnospirales;f__Lachnospiraceae;g__TF01-11;s__
ERR1135273_bin.20	d__Bacteria;p__Firmicutes_A;c__Clostridia;o__Lachnospirales;f__Lachnospiraceae;g__Bariatricus;s__Bariatricus sp004560705
ERR1135273_bin.35	d__Bacteria;p__Firmicutes_C;c__Negativicutes;o__Veillonellales;f__Dialisteraceae;g__Dialister;s__Dialister sp002320515
ERR1135273_bin.37	d__Bacteria;p__Proteobacteria;c__Gammaproteobacteria;o__Enterobacterales;f__Succinivibrionaceae;g__Succinivibrio;s__Succinivibrio hippei_A
ERR1135273_bin.39	d__Bacteria;p__Cyanobacteria;c__Vampirovibrionia;o__Gastranaerophilales;f__Gastranaerophilaceae;g__UBA2883;s__
ERR1135273_bin.51	d__Bacteria;p__Firmicutes;c__Bacilli;o__Erysipelotrichales;f__Erysipelotrichaceae;g__Bulleidia;s__Bulleidia massiliensis
ERR1135273_bin.8	d__Bacteria;p__Proteobacteria;c__Gammaproteobacteria;o__Enterobacterales;f__Succinivibrionaceae;g__UBA2810;s__UBA2810 sp002351705
ERR1135273_bin.81	d__Bacteria;p__Firmicutes_A;c__Clostridia;o__Lachnospirales;f__Lachnospiraceae;g__Blautia_A;s__
ERR1135274_bin.6	d__Bacteria;p__Cyanobacteria;c__Vampirovibrionia;o__Gastranaerophilales;f__Gastranaerophilaceae;g__Zag111;s__
ERR1135274_bin.61	d__Bacteria;p__Firmicutes_C;c__Negativicutes;o__Selenomonadales;f__Selenomonadaceae;g__Anaerovibrio;s__
ERR1135274_bin.69	d__Bacteria;p__Firmicutes;c__Bacilli;o__RFN20;f__CAG-288;g__UBA7642;s__
ERR1135275_bin.2	d__Bacteria;p__Firmicutes_A;c__Clostridia;o__Lachnospirales;f__Lachnospiraceae;g__Coprococcus;s__
ERR1135275_bin.23	d__Bacteria;p__Bacteroidota;c__Bacteroidia;o__Bacteroidales;f__UBA932;g__RC9;s__
ERR1135275_bin.38	d__Bacteria;p__Firmicutes_A;c__Clostridia;o__Lachnospirales;f__Lachnospiraceae;g__UBA2882;s__
ERR1135275_bin.52	d__Bacteria;p__Firmicutes_A;c__Clostridia;o__Lachnospirales;f__Lachnospiraceae;g__Eubacterium_J;s__
ERR1135275_bin.62	d__Bacteria;p__Bacteroidota;c__Bacteroidia;o__Bacteroidales;f__Bacteroidaceae;g__Prevotella;s__Prevotella sp002373375
ERR1135276_bin.43	d__Bacteria;p__Bacteroidota;c__Bacteroidia;o__Bacteroidales;f__Bacteroidaceae;g__Prevotella;s__Prevotella sp900543975
ERR1135276_bin.56	d__Bacteria;p__Firmicutes_A;c__Clostridia;o__Oscillospirales;f__Butyricicoccaceae;g__Butyricicoccus_A;s__Butyricicoccus_A porcorum
ERR1135276_bin.61	d__Bacteria;p__Firmicutes_C;c__Negativicutes;o__Selenomonadales;f__Selenomonadaceae;g__Selenomonas_A;s__Selenomonas_A sp004552655
ERR1135276_bin.72	d__Bacteria;p__Firmicutes;c__Bacilli;o__Erysipelotrichales;f__Erysipelotrichaceae;g__Bulleidia;s__
ERR1135276_bin.96	d__Bacteria;p__Firmicutes_A;c__Clostridia;o__Lachnospirales;f__Lachnospiraceae;g__Coprococcus;s__
ERR1135277_bin.10	d__Bacteria;p__Bacteroidota;c__Bacteroidia;o__Bacteroidales;f__W3P20-009;g__W3P20-009;s__W3P20-009 sp004552385
ERR1135277_bin.11	d__Bacteria;p__Firmicutes;c__Bacilli;o__RF39;f__CAG-1000;g__CAG-1000;s__
ERR1135277_bin.29	d__Bacteria;p__Firmicutes_A;c__Clostridia;o__Lachnospirales;f__Lachnospiraceae;g__Agathobacter;s__Agathobacter sp900543445
ERR1135277_bin.54	d__Bacteria;p__Firmicutes_A;c__Clostridia;o__Lachnospirales;f__Lachnospiraceae;g__Agathobacter;s__Agathobacter sp900548765
ERR1135278_bin.11	d__Bacteria;p__Firmicutes_C;c__Negativicutes;o__Veillonellales;f__Veillonellaceae;g__;s__
ERR1135278_bin.32	d__Bacteria;p__Firmicutes;c__Bacilli;o__RFN20;f__CAG-288;g__UBA7642;s__
ERR1135278_bin.69	d__Bacteria;p__Firmicutes_A;c__Clostridia;o__Lachnospirales;f__Lachnospiraceae;g__Oribacterium;s__Oribacterium sp004554245
ERR1135278_bin.75	d__Bacteria;p__Firmicutes_A;c__Clostridia;o__Lachnospirales;f__Lachnospiraceae;g__CAG-127;s__CAG-127 sp900553925
ERR1135279_bin.11	d__Bacteria;p__Firmicutes;c__Bacilli;o__RF39;f__CAG-822;g__UBA5364;s__
ERR1135280_bin.46	d__Bacteria;p__Firmicutes_A;c__Clostridia;o__Oscillospirales;f__Acutalibacteraceae;g__UBA737;s__
ERR1135281_bin.21	d__Bacteria;p__Firmicutes;c__Bacilli;o__RF39;f__CAG-302;g__CAG-302;s__CAG-302 sp001916775
ERR1135283_bin.47	d__Bacteria;p__Firmicutes_A;c__Clostridia_A;o__Christensenellales;f__CAG-138;g__;s__
ERR1135284_bin.14	d__Bacteria;p__Cyanobacteria;c__Vampirovibrionia;o__Gastranaerophilales;f__Gastranaerophilaceae;g__UBA6984;s__UBA6984 sp003258725
ERR1135284_bin.23	d__Bacteria;p__Bacteroidota;c__Bacteroidia;o__Bacteroidales;f__Muribaculaceae;g__CAG-279;s__CAG-279 sp004561555
ERR1135284_bin.38	d__Bacteria;p__Firmicutes_A;c__Clostridia;o__Lachnospirales;f__Lachnospiraceae;g__CAG-95;s__CAG-95 sp000436115
ERR1135285_bin.14	d__Bacteria;p__Firmicutes_A;c__Clostridia;o__Lachnospirales;f__Lachnospiraceae;g__Blautia;s__
ERR1135285_bin.74	d__Bacteria;p__Spirochaetota;c__Spirochaetia;o__Treponematales;f__Treponemataceae;g__Treponema_D;s__
ERR1135286_bin.7	d__Bacteria;p__Bacteroidota;c__Bacteroidia;o__Bacteroidales;f__F082;g__F082;s__
ERR1135286_bin.84	d__Bacteria;p__Firmicutes_A;c__Clostridia;o__Lachnospirales;f__Lachnospiraceae;g__Acetatifactor;s__
ERR1135288_bin.11	d__Bacteria;p__Firmicutes_C;c__Negativicutes;o__Selenomonadales;f__Selenomonadaceae;g__Anaerovibrio;s__
ERR1135288_bin.89	d__Bacteria;p__Firmicutes_A;c__Clostridia;o__Lachnospirales;f__Lachnospiraceae;g__TF01-11;s__
ERR1135288_bin.96	d__Bacteria;p__Firmicutes_A;c__Clostridia;o__Lachnospirales;f__Lachnospiraceae;g__Agathobacter;s__
ERR1135289_bin.22	d__Bacteria;p__Spirochaetota;c__Spirochaetia;o__Treponematales;f__Treponemataceae;g__Treponema_F;s__
ERR1135289_bin.33	d__Bacteria;p__Firmicutes_A;c__Clostridia_A;o__Christensenellales;f__CAG-138;g__PeH17;s__PeH17 sp900542285
ERR1135289_bin.44	d__Bacteria;p__Bacteroidota;c__Bacteroidia;o__Bacteroidales;f__Bacteroidaceae;g__UBA4372;s__
ERR1135290_bin.10	d__Bacteria;p__Cyanobacteria;c__Vampirovibrionia;o__Gastranaerophilales;f__Gastranaerophilaceae;g__UBA6984;s__

SGBs ID	Classification
ERR1135290_bin.47	d__Bacteria;p__Firmicutes;c__Bacilli;o__RF39;f__CAG-1000;g__CAG-1000;s__CAG-1000 sp004560915
ERR1135291_bin.45	d__Bacteria;p__Bacteroidota;c__Bacteroidia;o__Bacteroidales;f__Muribaculaceae;g__CAG-485;s__
ERR1135291_bin.83	d__Bacteria;p__Bacteroidota;c__Bacteroidia;o__Bacteroidales;f__UBA932;g__RC9;s__RC9 sp000433355
ERR1135291_bin.88	d__Bacteria;p__Bacteroidota;c__Bacteroidia;o__Bacteroidales;f__Bacteroidaceae;g__Prevotellamassilia;s__Prevotellamassilia sp004552865
ERR1135292_bin.53	d__Bacteria;p__Spirochaetota;c__Spirochaetia;o__Treponematales;f__Treponemataceae;g__Treponema_D;s__
ERR1135292_bin.87	d__Bacteria;p__Campylobacterota;c__Campylobacteria;o__Campylobacterales;f__Helicobacteraceae;g__Helicobacter_D;s__Helicobacter_D sp003288905
ERR1135293_bin.33	d__Bacteria;p__Firmicutes_A;c__Clostridia;o__Lachnospirales;f__Lachnospiraceae;g__Dorea;s__Dorea longicatena
ERR1135293_bin.44	d__Bacteria;p__Firmicutes_A;c__Clostridia_A;o__Christensenellales;f__CAG-917;g__CAG-475;s__CAG-475 sp900549225
ERR1135293_bin.50	d__Bacteria;p__Bacteroidota;c__Bacteroidia;o__Bacteroidales;f__Bacteroidaceae;g__Prevotella;s__Prevotella sp900544495
ERR1135294_bin.23	d__Bacteria;p__Bacteroidota;c__Bacteroidia;o__Bacteroidales;f__Bacteroidaceae;g__Prevotellamassilia;s__Prevotellamassilia sp004562975
ERR1135294_bin.5	d__Bacteria;p__Firmicutes_A;c__Clostridia;o__Lachnospirales;f__Lachnospiraceae;g__Butyrivibrio_A;s__Butyrivibrio_A crossotus
ERR1135294_bin.61	d__Bacteria;p__Bacteroidota;c__Bacteroidia;o__Bacteroidales;f__Bacteroidaceae;g__UBA6398;s__UBA6398 sp003150315
ERR1135295_bin.34	d__Bacteria;p__Firmicutes;c__Bacilli;o__ML615J-28;f__CAG-313;g__CAG-313;s__CAG-313 sp003539625
ERR1135295_bin.8	d__Bacteria;p__Firmicutes_A;c__Clostridia;o__Lachnospirales;f__Lachnospiraceae;g__UBA2882;s__UBA2882 sp900317505
ERR1135297_bin.27	d__Bacteria;p__Bacteroidota;c__Bacteroidia;o__Bacteroidales;f__Bacteroidaceae;g__Prevotellamassilia;s__Prevotellamassilia sp900546605
ERR1135297_bin.46	d__Bacteria;p__Proteobacteria;c__Alphaproteobacteria;o__RF32;f__CAG-239;g__51-20;s__51-20 sp900542055
ERR1135299_bin.83	d__Bacteria;p__Firmicutes;c__Bacilli;o__RFN20;f__CAG-288;g__UBA6879;s__
ERR1135300_bin.25	d__Bacteria;p__Firmicutes_A;c__Clostridia;o__Oscillospirales;f__Butyricicoccaceae;g__Butyricicoccus_A;s__Butyricicoccus_A sp002395695
ERR1135301_bin.23	d__Bacteria;p__Firmicutes_C;c__Negativicutes;o__Selenomonadales;f__Selenomonadaceae;g__RUG114;s__RUG114 sp900315615
ERR1135302_bin.36	d__Bacteria;p__Firmicutes_A;c__Clostridia;o__Lachnospirales;f__Lachnospiraceae;g__Agathobacter;s__
ERR1135304_bin.16	d__Bacteria;p__Firmicutes;c__Bacilli;o__RFN20;f__CAG-826;g__UBA733;s__UBA733 sp900539595
ERR1135304_bin.69	d__Bacteria;p__Firmicutes_A;c__Clostridia;o__Oscillospirales;f__CAG-272;g__CAG-841;s__
ERR1135306_bin.47	d__Bacteria;p__Bacteroidota;c__Bacteroidia;o__Bacteroidales;f__Bacteroidaceae;g__Prevotella;s__
ERR1135307_bin.30	d__Bacteria;p__Firmicutes_A;c__Clostridia;o__Lachnospirales;f__Lachnospiraceae;g__CAG-45;s__CAG-45 sp002299665
ERR1135308_bin.44	d__Bacteria;p__Firmicutes_A;c__Clostridia;o__Lachnospirales;f__Lachnospiraceae;g__CAG-791;s__CAG-791 sp000431495
ERR1135308_bin.99	d__Bacteria;p__Firmicutes_A;c__Clostridia;o__Lachnospirales;f__Lachnospiraceae;g__Agathobacter;s__Agathobacter sp000434275
ERR1135309_bin.22	d__Bacteria;p__Firmicutes_A;c__Clostridia;o__Oscillospirales;f__Acutalibacteraceae;g__Ruminococcus_E;s__Ruminococcus_E bromii_A
ERR1135311_bin.31	d__Bacteria;p__Firmicutes_A;c__Clostridia;o__Lachnospirales;f__Lachnospiraceae;g__C-53;s__
ERR1135311_bin.79	d__Bacteria;p__Firmicutes_A;c__Clostridia;o__Peptostreptococcales;f__Anaerovoracaceae;g__;s__
ERR1135311_bin.91	d__Bacteria;p__Firmicutes;c__Bacilli;o__Mycoplasmatales;f__Mycoplasmoidaceae;g__;s__
ERR1135311_bin.94	d__Bacteria;p__Firmicutes_A;c__Clostridia;o__Lachnospirales;f__Lachnospiraceae;g__;s__
ERR1135312_bin.41	d__Bacteria;p__Bacteroidota;c__Bacteroidia;o__Bacteroidales;f__Bacteroidaceae;g__Prevotella;s__Prevotella sp900319905
ERR1135315_bin.55	d__Bacteria;p__Campylobacterota;c__Campylobacteria;o__Campylobacterales;f__Helicobacteraceae;g__Helicobacter_D;s__
ERR1135316_bin.81	d__Bacteria;p__Bacteroidota;c__Bacteroidia;o__Bacteroidales;f__Bacteroidaceae;g__Prevotella;s__Prevotella sp000436915
ERR1135317_bin.24	d__Bacteria;p__Spirochaetota;c__Spirochaetia;o__Treponematales;f__Treponemataceae;g__Treponema_D;s__
ERR1135318_bin.25	d__Bacteria;p__Bacteroidota;c__Bacteroidia;o__Bacteroidales;f__P3;g__UBA3388;s__UBA3388 sp004551865
ERR1135318_bin.39	d__Bacteria;p__Firmicutes;c__Bacilli;o__RF39;f__CAG-302;g__CAG-762;s__
ERR1135318_bin.98	d__Bacteria;p__Firmicutes_A;c__Clostridia_A;o__Christensenellales;f__CAG-74;g__SFFH01;s__SFFH01 sp004557905
ERR1135319_bin.2	d__Bacteria;p__Firmicutes_A;c__Clostridia;o__Oscillospirales;f__CAG-272;g__CAG-724;s__CAG-724 sp900551005
ERR1135319_bin.20	d__Bacteria;p__Firmicutes_A;c__Clostridia;o__Oscillospirales;f__Oscillospiraceae;g__CAG-83;s__CAG-83 sp004555205
ERR1135319_bin.63	d__Bacteria;p__Firmicutes;c__Bacilli;o__RF39;f__CAG-611;g__RUG591;s__
ERR1135319_bin.78	d__Bacteria;p__Firmicutes;c__Bacilli;o__RF39;f__UBA5348;g__CAG-1193;s__
ERR1135320_bin.26	d__Bacteria;p__Firmicutes_A;c__Clostridia;o__Lachnospirales;f__Lachnospiraceae;g__TF01-11;s__
ERR1135322_bin.22	d__Bacteria;p__Firmicutes_A;c__Clostridia;o__Oscillospirales;f__Oscillospiraceae;g__CAG-170;s__CAG-170 sp002404795
ERR1135322_bin.29	d__Bacteria;p__Firmicutes_A;c__Clostridia;o__Lachnospirales;f__Lachnospiraceae;g__Agathobacter;s__Agathobacter sp900550845
ERR1135322_bin.55	d__Bacteria;p__Firmicutes_A;c__Clostridia;o__Oscillospirales;f__Oscillospiraceae;g__CAG-110;s__
ERR1135324_bin.51	d__Bacteria;p__Bacteroidota;c__Bacteroidia;o__Bacteroidales;f__Bacteroidaceae;g__Prevotella;s__Prevotella sp004558865
ERR1135325_bin.13	d__Bacteria;p__Firmicutes_A;c__Clostridia;o__Lachnospirales;f__Lachnospiraceae;g__Clostridium_AI;s__
ERR1135325_bin.58	d__Bacteria;p__Firmicutes_A;c__Clostridia;o__Lachnospirales;f__Lachnospiraceae;g__1XD8-76;s__
ERR1135327_bin.40	d__Bacteria;p__Firmicutes_A;c__Clostridia;o__Lachnospirales;f__Lachnospiraceae;g__Eubacterium_F;s__
ERR1135327_bin.92	d__Bacteria;p__Bacteroidota;c__Bacteroidia;o__Bacteroidales;f__Paludibacteraceae;g__RF16;s__
ERR1135328_bin.18	d__Bacteria;p__Firmicutes_A;c__Clostridia;o__Oscillospirales;f__Oscillospiraceae;g__CAG-110;s__CAG-110 sp900540635
ERR1135328_bin.24	d__Bacteria;p__Firmicutes_A;c__Clostridia;o__Oscillospirales;f__Oscillospiraceae;g__ER4;s__ER4 sp900317525
ERR1135328_bin.36	d__Bacteria;p__Bacteroidota;c__Bacteroidia;o__Bacteroidales;f__Bacteroidaceae;g__Prevotella;s__Prevotella sp002300055
ERR1135328_bin.48	d__Bacteria;p__Firmicutes_A;c__Clostridia;o__TANB77;f__CAG-508;g__CAG-492;s__
ERR1135328_bin.73	d__Bacteria;p__Firmicutes_A;c__Clostridia;o__Lachnospirales;f__Lachnospiraceae;g__Coprococcus_A;s__Coprococcus_A catus
ERR1135330_bin.69	d__Bacteria;p__Firmicutes_A;c__Clostridia;o__Lachnospirales;f__Lachnospiraceae;g__Agathobacter;s__Agathobacter sp900317585
ERR1135333_bin.56	d__Bacteria;p__Cyanobacteria;c__Vampirovibrionia;o__Gastranaerophilales;f__Gastranaerophilaceae;g__UBA791;s__
ERR1135334_bin.33	d__Bacteria;p__Firmicutes_A;c__Clostridia;o__Lachnospirales;f__Lachnospiraceae;g__UBA629;s__UBA629 sp900316625
ERR1135335_bin.32	d__Bacteria;p__Firmicutes;c__Bacilli;o__Lactobacillales;f__Lactobacillaceae;g__Limosilactobacillus;s__Limosilactobacillus reuteri
ERR1135336_bin.80	d__Bacteria;p__Firmicutes_C;c__Negativicutes;o__Selenomonadales;f__Selenomonadaceae;g__Selenomonas_C;s__Selenomonas_C sp900315575
ERR1135337_bin.78	d__Bacteria;p__Firmicutes_A;c__Clostridia;o__Lachnospirales;f__Lachnospiraceae;g__Ruminococcus_A;s__
ERR1135341_bin.51	d__Bacteria;p__Firmicutes_C;c__Negativicutes;o__Selenomonadales;f__Selenomonadaceae;g__Mitsuokella;s__Mitsuokella jalaludinii
ERR1135341_bin.54	d__Bacteria;p__Firmicutes_C;c__Negativicutes;o__Veillonellales;f__Dialisteraceae;g__Dialister;s__Dialister sp000434475
ERR1135341_bin.9	d__Bacteria;p__Bacteroidota;c__Bacteroidia;o__Bacteroidales;f__UBA932;g__RC9;s__RC9 sp900546925
ERR1135342_bin.51	d__Bacteria;p__Firmicutes_C;c__Negativicutes;o__Veillonellales;f__Megasphaeraceae;g__Megasphaera;s__Megasphaera elsdenii
ERR1135342_bin.65	d__Bacteria;p__Spirochaetota;c__Spirochaetia;o__Treponematales;f__Treponemataceae;g__Treponema_D;s__Treponema_D sp002395155
ERR1135346_bin.81	d__Bacteria;p__Firmicutes;c__Bacilli;o__Erysipelotrichales;f__Erysipelatoclostridiaceae;g__Catenibacterium;s__Catenibacterium mitsuokai
ERR1135347_bin.12	d__Bacteria;p__Spirochaetota;c__Spirochaetia;o__Treponematales;f__Treponemataceae;g__Treponema_D;s__
ERR1135347_bin.19	d__Bacteria;p__Spirochaetota;c__Spirochaetia;o__Treponematales;f__Treponemataceae;g__Treponema_D;s__
ERR1135348_bin.2	d__Bacteria;p__Firmicutes_A;c__Clostridia;o__Oscillospirales;f__Ruminococcaceae;g__Gemmiger;s__Gemmiger qucibialis
ERR1135348_bin.33	d__Bacteria;p__Proteobacteria;c__Alphaproteobacteria;o__RF32;f__CAG-239;g__;s__
ERR1135348_bin.59	d__Bacteria;p__Firmicutes_A;c__Clostridia;o__Lachnospirales;f__Lachnospiraceae;g__Lachnospira;s__
ERR1135348_bin.60	d__Bacteria;p__Bacteroidota;c__Bacteroidia;o__Bacteroidales;f__Bacteroidaceae;g__Prevotellamassilia;s__Prevotellamassilia sp004552155
ERR1135350_bin.18	d__Bacteria;p__Firmicutes;c__Bacilli;o__RF39;f__CAG-1000;g__CAG-1000;s__CAG-1000 sp000434555
ERR1135350_bin.23	d__Bacteria;p__Firmicutes_A;c__Clostridia_A;o__Christensenellales;f__CAG-917;g__CAG-349;s__CAG-349 sp003539515
ERR1135350_bin.27	d__Bacteria;p__Spirochaetota;c__Spirochaetia;o__Treponematales;f__Treponemataceae;g__Treponema_D;s__
ERR1135351_bin.34	d__Bacteria;p__Campylobacterota;c__Campylobacteria;o__Campylobacterales;f__Campylobacteraceae;g__Campylobacter;s__
ERR1135351_bin.45	d__Bacteria;p__Bacteroidota;c__Bacteroidia;o__Bacteroidales;f__Bacteroidaceae;g__Prevotella;s__Prevotella sp900315635
ERR1135351_bin.65	d__Bacteria;p__Firmicutes_A;c__Clostridia;o__Lachnospirales;f__Lachnospiraceae;g__Eubacterium_J;s__
ERR1135352_bin.30	d__Bacteria;p__Firmicutes_A;c__Clostridia;o__Oscillospirales;f__Oscillospiraceae;g__CAG-83;s__CAG-83 sp004557655
ERR1135353_bin.10	d__Bacteria;p__Firmicutes_A;c__Clostridia;o__Oscillospirales;f__Acutalibacteraceae;g__DTU089_A;s__
ERR1135356_bin.12	d__Bacteria;p__Firmicutes_A;c__Clostridia;o__Oscillospirales;f__Oscillospiraceae;g__UBA1777;s__
ERR1135356_bin.72	d__Bacteria;p__Bacteroidota;c__Bacteroidia;o__Bacteroidales;f__Bacteroidaceae;g__UBA4372;s__
ERR1135357_bin.28	d__Bacteria;p__Bacteroidota;c__Bacteroidia;o__Bacteroidales;f__Bacteroidaceae;g__Prevotella;s__
ERR1135359_bin.27	d__Archaea;p__Methanobacteriota;c__Methanobacteria;o__Methanobacteriales;f__Methanobacteriaceae;g__Methanobrevibacter_A;s__Methanobrevibacter_A smithii
ERR1135360_bin.30	d__Bacteria;p__Firmicutes_A;c__Clostridia;o__Oscillospirales;f__Butyricicoccaceae;g__;s__
ERR1135360_bin.47	d__Bacteria;p__Firmicutes_A;c__Clostridia;o__Oscillospirales;f__Oscillospiraceae;g__CAG-110;s__
ERR1135362_bin.43	d__Bacteria;p__Firmicutes_A;c__Clostridia;o__Peptostreptococcales;f__Anaerovoracaceae;g__;s__

SGBs ID	Classification
ERR1135362_bin.58	d__Bacteria;p__Firmicutes;c__Bacilli;o__Erysipelotrichales;f__Erysipelotrichaceae;g__UBA636;s__UBA636 sp002299675
ERR1135363_bin.22	d__Bacteria;p__Spirochaetota;c__Spirochaetia;o__Treponematales;f__Treponemataceae;g__Treponema_D;s__
ERR1135364_bin.79	d__Bacteria;p__Bacteroidota;c__Bacteroidia;o__Bacteroidales;f__Bacteroidaceae;g__Prevotella;s__
ERR1135366_bin.11	d__Bacteria;p__Proteobacteria;c__Alphaproteobacteria;o__Rs-D84;f__Rs-D84;g__Rs-D84;s__
ERR1135368_bin.10	d__Bacteria;p__Campylobacterota;c__Campylobacteria;o__Campylobacterales;f__Campylobacteraceae;g__Campylobacter;s__Campylobacter sp002139915
ERR1135370_bin.25	d__Bacteria;p__Firmicutes_A;c__Clostridia;o__Lachnospirales;f__Lachnospiraceae;g__CAG-194;s__
ERR1135370_bin.31	d__Bacteria;p__Firmicutes_A;c__Clostridia;o__Lachnospirales;f__Lachnospiraceae;g__AM51-8;s__
ERR1135371_bin.19	d__Bacteria;p__Bacteroidota;c__Bacteroidia;o__Bacteroidales;f__Bacteroidaceae;g__Prevotellamassilia;s__
ERR1135371_bin.2	d__Bacteria;p__Bacteroidota;c__Bacteroidia;o__Bacteroidales;f__Paludibacteraceae;g__RF16;s__
ERR1135371_bin.25	d__Bacteria;p__Firmicutes_A;c__Clostridia;o__Lachnospirales;f__Lachnospiraceae;g__UBA2882;s__
ERR1135371_bin.30	d__Bacteria;p__Firmicutes_A;c__Clostridia;o__Lachnospirales;f__Lachnospiraceae;g__Roseburia;s__
ERR1135371_bin.47	d__Bacteria;p__Verrucomicrobiota;c__Kiritimatiellae;o__RFP12;f__UBA1067;g__UBA1067;s__
ERR1135373_bin.22	d__Bacteria;p__Firmicutes_C;c__Negativicutes;o__Acidaminococcales;f__Acidaminococcaceae;g__Phascolarctobacterium_A;s__Phascolarctobacterium_A succinatutens
ERR1135373_bin.49	d__Bacteria;p__Bacteroidota;c__Bacteroidia;o__Bacteroidales;f__Bacteroidaceae;g__Prevotellamassilia;s__Prevotellamassilia sp004555425
ERR1135374_bin.10	d__Bacteria;p__Firmicutes_A;c__Clostridia;o__Lachnospirales;f__Lachnospiraceae;g__Eubacterium_J;s__
ERR1135376_bin.19	d__Bacteria;p__Firmicutes;c__Bacilli;o__Lactobacillales;f__Lactobacillaceae;g__Lactobacillus;s__Lactobacillus amylovorus
ERR1135377_bin.41	d__Bacteria;p__Spirochaetota;c__Spirochaetia;o__Treponematales;f__Treponemataceae;g__Treponema_D;s__
ERR1135378_bin.20	d__Bacteria;p__Firmicutes_A;c__Clostridia;o__TANB77;f__CAG-508;g__CAG-273;s__CAG-273 sp003507395
ERR1135378_bin.53	d__Bacteria;p__Spirochaetota;c__Spirochaetia;o__Treponematales;f__Treponemataceae;g__Treponema_D;s__
ERR1135378_bin.9	d__Bacteria;p__Firmicutes_A;c__Clostridia;o__Oscillospirales;f__Ruminococcaceae;g__Angelakisella;s__Angelakisella sp004554485
ERR1135379_bin.27	d__Bacteria;p__Verrucomicrobiota;c__Kiritimatiellae;o__RFP12;f__UBA1067;g__UBA1067;s__
ERR1135380_bin.23	d__Bacteria;p__Bacteroidota;c__Bacteroidia;o__Bacteroidales;f__Muribaculaceae;g__;s__
ERR1135380_bin.51	d__Bacteria;p__Firmicutes_A;c__Clostridia_A;o__Christensenellales;f__CAG-138;g__SFDB01;s__
ERR1135381_bin.24	d__Bacteria;p__Bacteroidota;c__Bacteroidia;o__Bacteroidales;f__Bacteroidaceae;g__Prevotella;s__
ERR1135383_bin.11	d__Bacteria;p__Bacteroidota;c__Bacteroidia;o__Bacteroidales;f__Bacteroidaceae;g__Prevotella;s__Prevotella sp002251295
ERR1135383_bin.33	d__Bacteria;p__Firmicutes_A;c__Clostridia;o__Oscillospirales;f__Acutalibacteraceae;g__UMGS1532;s__
ERR1135383_bin.49	d__Bacteria;p__Firmicutes_A;c__Clostridia;o__Oscillospirales;f__Acutalibacteraceae;g__CAG-488;s__
ERR1135385_bin.10	d__Archaea;p__Methanobacteriota;c__Methanobacteria;o__Methanobacteriales;f__Methanobacteriaceae;g__Methanobrevibacter_A;s__
ERR1135385_bin.11	d__Bacteria;p__Firmicutes_C;c__Negativicutes;o__Selenomonadales;f__Selenomonadaceae;g__;s__
ERR1135385_bin.68	d__Bacteria;p__Firmicutes;c__Bacilli;o__RF39;f__CAG-1000;g__CAG-1000;s__CAG-1000 sp004552445
ERR1135385_bin.9	d__Bacteria;p__Firmicutes;c__Bacilli;o__RF39;f__CAG-1000;g__CAG-628;s__CAG-628 sp003524085
ERR1135386_bin.37	d__Bacteria;p__Firmicutes_A;c__Clostridia_A;o__Christensenellales;f__CAG-138;g__PeH17;s__PeH17 sp004556165
ERR1135387_bin.51	d__Bacteria;p__Firmicutes_A;c__Clostridia_A;o__Christensenellales;f__CAG-138;g__UBA1685;s__UBA1685 sp004560435
ERR1135388_bin.55	d__Bacteria;p__Bacteroidota;c__Bacteroidia;o__Bacteroidales;f__Muribaculaceae;g__SFTJ01;s__
ERR1135389_bin.14	d__Bacteria;p__Verrucomicrobiota;c__Verrucomicrobiae;o__Verrucomicrobiales;f__Akkermansiaceae;g__Akkermansia;s__
ERR1135389_bin.43	d__Bacteria;p__Bacteroidota;c__Bacteroidia;o__Bacteroidales;f__Muribaculaceae;g__UBA7173;s__
ERR1135389_bin.51	d__Bacteria;p__Firmicutes_A;c__Clostridia;o__Oscillospirales;f__Acutalibacteraceae;g__Ruminococcus_E;s__
ERR1135389_bin.61	d__Bacteria;p__Spirochaetota;c__Spirochaetia;o__Treponematales;f__Treponemataceae;g__Treponema_D;s__
ERR1135391_bin.3	d__Bacteria;p__Firmicutes_A;c__Clostridia;o__Oscillospirales;f__Oscillospiraceae;g__F23-B02;s__
ERR1135391_bin.5	d__Bacteria;p__Firmicutes_A;c__Clostridia;o__Oscillospirales;f__Ruminococcaceae;g__CAG-115;s__
ERR1135396_bin.46	d__Bacteria;p__Firmicutes_A;c__Clostridia;o__Oscillospirales;f__Oscillospiraceae;g__Oscillibacter;s__
ERR1135397_bin.48	d__Bacteria;p__Firmicutes_A;c__Clostridia;o__Lachnospirales;f__Lachnospiraceae;g__;s__
ERR1135398_bin.29	d__Bacteria;p__Firmicutes_A;c__Clostridia;o__Oscillospirales;f__Oscillospiraceae;g__CAG-110;s__CAG-110 sp004556205
ERR1135399_bin.16	d__Bacteria;p__Firmicutes_A;c__Clostridia;o__Oscillospirales;f__Oscillospiraceae;g__CAG-110;s__
ERR1135400_bin.30	d__Bacteria;p__Bacteroidota;c__Bacteroidia;o__Bacteroidales;f__Bacteroidaceae;g__Prevotellamassilia;s__
ERR1135401_bin.33	d__Archaea;p__Methanobacteriota;c__Methanobacteria;o__Methanobacteriales;f__Methanobacteriaceae;g__Methanobrevibacter_A;s__
ERR1135401_bin.43	d__Bacteria;p__Firmicutes;c__Bacilli;o__RF39;f__CAG-611;g__CAG-877;s__
ERR1135402_bin.1	d__Bacteria;p__Firmicutes_A;c__Clostridia;o__TANB77;f__CAG-508;g__UMGS1781;s__UMGS1781 sp004554705
ERR1135403_bin.7	d__Bacteria;p__Firmicutes_A;c__Clostridia;o__Oscillospirales;f__Oscillospiraceae;g__CAG-110;s__
ERR1135406_bin.22	d__Bacteria;p__Firmicutes_A;c__Clostridia_A;o__Christensenellales;f__CAG-138;g__Phil1;s__Phil1 sp004558525
ERR1135407_bin.19	d__Bacteria;p__Bacteroidota;c__Bacteroidia;o__Bacteroidales;f__Bacteroidaceae;g__Prevotella;s__
ERR1135407_bin.2	d__Bacteria;p__Firmicutes_A;c__Clostridia;o__Peptostreptococcales;f__Anaerovoracaceae;g__;s__
ERR1135407_bin.25	d__Bacteria;p__Firmicutes_A;c__Clostridia;o__Oscillospirales;f__Oscillospiraceae;g__CAG-103;s__
ERR1135407_bin.53	d__Bacteria;p__Firmicutes;c__Bacilli;o__RF39;f__CAG-1000;g__UBA6985;s__
ERR1135408_bin.15	d__Bacteria;p__Firmicutes_A;c__Clostridia;o__Oscillospirales;f__Ruminococcaceae;g__Ruminiclostridium_E;s__
ERR1135408_bin.67	d__Bacteria;p__Bacteroidota;c__Bacteroidia;o__Bacteroidales;f__UBA932;g__RC9;s__
ERR1135409_bin.26	d__Bacteria;p__Firmicutes_A;c__Clostridia;o__Oscillospirales;f__Oscillospiraceae;g__F23-B02;s__F23-B02 sp004558025
ERR1135410_bin.13	d__Bacteria;p__Bacteroidota;c__Bacteroidia;o__Bacteroidales;f__Bacteroidaceae;g__Prevotella;s__Prevotella sp002251245
ERR1135410_bin.80	d__Bacteria;p__Proteobacteria;c__Gammaproteobacteria;o__Enterobacterales;f__Enterobacteriaceae;g__Escherichia;s__Escherichia flexneri
ERR1135415_bin.35	d__Bacteria;p__Firmicutes_A;c__Clostridia;o__Oscillospirales;f__Ruminococcaceae;g__Ruminococcus;s__Ruminococcus flavefaciens_G
ERR1135415_bin.65	d__Bacteria;p__Firmicutes_A;c__Clostridia_A;o__Christensenellales;f__CAG-138;g__RUG472;s__
ERR1135416_bin.41	d__Bacteria;p__Actinobacteriota;c__Actinomycetia;o__Actinomycetales;f__Bifidobacteriaceae;g__Bifidobacterium;s__Bifidobacterium pseudolongum
ERR1135417_bin.29	d__Bacteria;p__Firmicutes_A;c__Clostridia;o__Oscillospirales;f__Acutalibacteraceae;g__UBA1425;s__
ERR1135417_bin.79	d__Bacteria;p__Firmicutes_A;c__Clostridia;o__Peptostreptococcales;f__Anaerovoracaceae;g__UBA1191;s__
ERR1135418_bin.17	d__Bacteria;p__Firmicutes_A;c__Clostridia;o__Oscillospirales;f__Oscillospiraceae;g__CAG-83;s__
ERR1135419_bin.22	d__Bacteria;p__Firmicutes_A;c__Clostridia;o__Oscillospirales;f__Oscillospiraceae;g__CAG-103;s__CAG-103 sp900317855
ERR1135419_bin.50	d__Bacteria;p__Bacteroidota;c__Bacteroidia;o__Bacteroidales;f__UBA932;g__RC9;s__
ERR1135419_bin.55	d__Bacteria;p__Firmicutes_A;c__Clostridia;o__Oscillospirales;f__Acutalibacteraceae;g__CAG-177;s__
ERR1135419_bin.72	d__Bacteria;p__Campylobacterota;c__Campylobacteria;o__Campylobacterales;f__Helicobacteraceae;g__Helicobacter_D;s__
ERR1135419_bin.8	d__Bacteria;p__Firmicutes_C;c__Negativicutes;o__Selenomonadales;f__Selenomonadaceae;g__Selenomonas_C;s__Selenomonas_C bovis
ERR1135420_bin.28	d__Bacteria;p__Spirochaetota;c__Spirochaetia;o__Treponematales;f__Treponemataceae;g__Treponema_D;s__
ERR1135421_bin.27	d__Bacteria;p__Bacteroidota;c__Bacteroidia;o__Bacteroidales;f__Bacteroidaceae;g__Bacteroides;s__
ERR1135422_bin.26	d__Bacteria;p__Firmicutes_A;c__Clostridia;o__Oscillospirales;f__Acutalibacteraceae;g__Eubacterium_R;s__
ERR1135422_bin.56	d__Bacteria;p__Cyanobacteria;c__Vampirovibrionia;o__Gastranaerophilales;f__Gastranaerophilaceae;g__Zag1;s__
ERR1135422_bin.61	d__Bacteria;p__Firmicutes_A;c__Clostridia;o__Peptostreptococcales;f__Anaerovoracaceae;g__;s__
ERR1135424_bin.1	d__Bacteria;p__Firmicutes_A;c__Clostridia;o__Lachnospirales;f__Lachnospiraceae;g__UBA2882;s__
ERR1135425_bin.34	d__Bacteria;p__Firmicutes_A;c__Clostridia;o__Oscillospirales;f__Acutalibacteraceae;g__CAG-180;s__
ERR1135426_bin.24	d__Bacteria;p__Firmicutes_A;c__Clostridia;o__Oscillospirales;f__Oscillospiraceae;g__CAG-110;s__
ERR1135426_bin.43	d__Bacteria;p__Firmicutes_A;c__Clostridia;o__Oscillospirales;f__Acutalibacteraceae;g__;s__
ERR1135426_bin.50	d__Bacteria;p__Actinobacteriota;c__Coriobacteriia;o__Coriobacteriales;f__QAMH01;g__W2P13-069;s__W2P13-069 sp004553405
ERR1135426_bin.54	d__Bacteria;p__Firmicutes_A;c__Clostridia;o__Peptostreptococcales;f__Anaerovoracaceae;g__UBA1191;s__
ERR1135429_bin.48	d__Bacteria;p__Bacteroidota;c__Bacteroidia;o__Bacteroidales;f__Paludibacteraceae;g__RF16;s__
ERR1135433_bin.33	d__Bacteria;p__Firmicutes_A;c__Clostridia;o__Oscillospirales;f__Oscillospiraceae;g__CAG-110;s__
ERR1135435_bin.8	d__Bacteria;p__Firmicutes_A;c__Clostridia;o__Oscillospirales;f__Oscillospiraceae;g__Oscillibacter;s__
ERR1135439_bin.5	d__Bacteria;p__Firmicutes_A;c__Clostridia;o__TANB77;f__CAG-508;g__SFKP01;s__SFKP01 sp004553715
ERR1135439_bin.54	d__Bacteria;p__Firmicutes_A;c__Clostridia;o__Oscillospirales;f__Oscillospiraceae;g__CAG-110;s__
ERR1135441_bin.14	d__Bacteria;p__Firmicutes;c__Bacilli;o__Staphylococcales;f__Salinicoccaceae;g__Nosocomiicoccus;s__
ERR1135441_bin.78	d__Bacteria;p__Spirochaetota;c__Spirochaetia;o__Treponematales;f__Treponemataceae;g__Treponema_D;s__
ERR1135445_bin.40	d__Bacteria;p__Firmicutes_A;c__Clostridia;o__Oscillospirales;f__Acutalibacteraceae;g__CAG-177;s__

SGBs ID	Classification
ERR1135446_bin.30	d__Bacteria;p__Firmicutes_A;c__Clostridia;o__Oscillospirales;f__Oscillospiraceae;g__CAG-103;s__CAG-103 sp004556965
ERR1135446_bin.34	d__Bacteria;p__Bacteroidota;c__Bacteroidia;o__Bacteroidales;f__Marinilabiliaceae;g__Breznakibacter;s__
ERR1135447_bin.18	d__Bacteria;p__Firmicutes;c__Bacilli;o__RF39;f__CAG-611;g__CAG-877;s__
ERR1135447_bin.34	d__Bacteria;p__Firmicutes_A;c__Clostridia;o__Oscillospirales;f__Oscillospiraceae;g__F23-B02;s__
ERR1135449_bin.10	d__Bacteria;p__Firmicutes;c__Bacilli;o__Erysipelotrichales;f__Erysipelotrichaceae;g__UBA1783;s__
ERR1135449_bin.13	d__Bacteria;p__Firmicutes_A;c__Clostridia;o__Lachnospirales;f__Lachnospiraceae;g__;s__
ERR1135450_bin.9	d__Bacteria;p__Firmicutes_A;c__Clostridia;o__Oscillospirales;f__Oscillospiraceae;g__F23-B02;s__
ERR1135452_bin.26	d__Bacteria;p__Firmicutes_A;c__Clostridia;o__Oscillospirales;f__Oscillospiraceae;g__;s__
ERR1135453_bin.4	d__Bacteria;p__Firmicutes_A;c__Clostridia;o__Lachnospirales;f__Lachnospiraceae;g__CAG-95;s__
ERR1135453_bin.41	d__Bacteria;p__Firmicutes_A;c__Clostridia;o__Oscillospirales;f__Oscillospiraceae;g__CAG-83;s__CAG-83 sp900313295
ERR1135453_bin.49	d__Bacteria;p__Bacteroidota;c__Bacteroidia;o__Bacteroidales;f__Paludibacteraceae;g__SFVR01;s__
ERR1135455_bin.2	d__Bacteria;p__Firmicutes_A;c__Clostridia;o__Lachnospirales;f__Lachnospiraceae;g__Acetatifactor;s__
ERR1135456_bin.15	d__Bacteria;p__Firmicutes_A;c__Clostridia;o__Oscillospirales;f__Acutalibacteraceae;g__CAG-177;s__CAG-177 sp000431775
ERR1135456_bin.36	d__Bacteria;p__Firmicutes_A;c__Clostridia;o__Oscillospirales;f__Oscillospiraceae;g__F23-B02;s__
ERR1135458_bin.26	d__Bacteria;p__Firmicutes;c__Bacilli;o__Erysipelotrichales;f__Erysipelotrichaceae;g__;s__
ERR1135458_bin.47	d__Bacteria;p__Bacteroidota;c__Bacteroidia;o__Bacteroidales;f__Bacteroidaceae;g__Prevotellamassilia;s__
ERR1135458_bin.60	d__Bacteria;p__Firmicutes_A;c__Clostridia;o__Oscillospirales;f__Acutalibacteraceae;g__Eubacterium_R;s__
ERR1135458_bin.70	d__Bacteria;p__Cyanobacteria;c__Vampirovibrionia;o__Gastranaerophilales;f__Gastranaerophilaceae;g__RUG841;s__
ERR1135459_bin.47	d__Bacteria;p__Firmicutes;c__Bacilli;o__RF39;f__CAG-822;g__;s__
ERR1135459_bin.65	d__Bacteria;p__Firmicutes_A;c__Clostridia;o__Oscillospirales;f__Acutalibacteraceae;g__DTU089_A;s__
ERR1135460_bin.61	d__Bacteria;p__Firmicutes_A;c__Clostridia;o__Oscillospirales;f__Oscillospiraceae;g__ER4;s__
ERR1135462_bin.12	d__Bacteria;p__Firmicutes_A;c__Clostridia;o__Oscillospirales;f__CAG-382;g__SFLA01;s__SFLA01 sp004553575
ERR1135462_bin.24	d__Bacteria;p__Firmicutes_A;c__Clostridia;o__Oscillospirales;f__Oscillospiraceae;g__;s__
ERR1135462_bin.8	d__Bacteria;p__Firmicutes_A;c__Clostridia;o__Oscillospirales;f__Acutalibacteraceae;g__CAG-177;s__
ERR1135464_bin.16	d__Bacteria;p__Proteobacteria;c__Alphaproteobacteria;o__Rs-D84;f__Rs-D84;g__Rs-D84;s__
ERR1135464_bin.47	d__Bacteria;p__Cyanobacteria;c__Vampirovibrionia;o__Gastranaerophilales;f__Gastranaerophilaceae;g__UBA2813;s__
ERR1135464_bin.7	d__Bacteria;p__Bacteroidota;c__Bacteroidia;o__Bacteroidales;f__UBA932;g__RC9;s__RC9 sp004556005
ERR1135464_bin.77	d__Bacteria;p__Firmicutes_A;c__Clostridia;o__Oscillospirales;f__Ruminococcaceae;g__Ruminococcus;s__Ruminococcus flavefaciens_B
ERR1135464_bin.8	d__Bacteria;p__Firmicutes_A;c__Clostridia;o__Oscillospirales;f__Acutalibacteraceae;g__UBA737;s__
ERR1135465_bin.7	d__Bacteria;p__Firmicutes_A;c__Clostridia;o__Lachnospirales;f__Lachnospiraceae;g__Frisingicoccus;s__
ERR1135465_bin.9	d__Bacteria;p__Bacteroidota;c__Bacteroidia;o__Bacteroidales;f__UBA3663;g__UBA3663;s__
ERR1135466_bin.30	d__Bacteria;p__Firmicutes_A;c__Clostridia;o__Oscillospirales;f__Oscillospiraceae;g__CAG-110;s__
ERR1135466_bin.58	d__Bacteria;p__Spirochaetota;c__Spirochaetia;o__Treponematales;f__Treponemataceae;g__Treponema_D;s__
ERR1135467_bin.21	d__Bacteria;p__Firmicutes_A;c__Clostridia;o__Oscillospirales;f__Oscillospiraceae;g__CAG-170;s__
ERR1135468_bin.29	d__Archaea;p__Methanobacteriota;c__Methanobacteria;o__Methanobacteriales;f__Methanobacteriaceae;g__Methanobrevibacter;s__Methanobrevibacter ruminantium_A
ERR1135468_bin.57	d__Bacteria;p__Firmicutes_A;c__Clostridia;o__Oscillospirales;f__Oscillospiraceae;g__CAG-110;s__
ERR1135470_bin.34	d__Bacteria;p__Firmicutes_A;c__Clostridia;o__Lachnospirales;f__Lachnospiraceae;g__SFDP01;s__
ERR1135471_bin.11	d__Bacteria;p__Firmicutes_A;c__Clostridia_A;o__Christensenellales;f__CAG-138;g__SFEL01;s__
ERR1135471_bin.59	d__Bacteria;p__Planctomycetota;c__Planctomycetes;o__Pirellulales;f__Thermoguttaceae;g__;s__
ERR1135472_bin.52	d__Bacteria;p__Planctomycetota;c__Planctomycetes;o__Pirellulales;f__Thermoguttaceae;g__;s__
ERR1135472_bin.8	d__Bacteria;p__Firmicutes_A;c__Clostridia;o__Oscillospirales;f__Oscillospiraceae;g__UBA738;s__UBA738 sp004557735
ERR1135472_bin.83	d__Bacteria;p__Firmicutes_A;c__Clostridia_A;o__Christensenellales;f__CAG-138;g__SFEL01;s__SFEL01 sp004557245
TP001_bin.109	d__Bacteria;p__Proteobacteria;c__Gammaproteobacteria;o__Enterobacterales;f__Succinivibrionaceae;g__Succinivibrio;s__
TP001_bin.117	d__Bacteria;p__Bacteroidota;c__Bacteroidia;o__Bacteroidales;f__Muribaculaceae;g__CAG-873;s__CAG-873 sp004553485
TP001_bin.18	d__Bacteria;p__Firmicutes;c__Bacilli;o__RF39;f__CAG-611;g__UBA3789;s__
TP001_bin.27	d__Bacteria;p__Cyanobacteria;c__Vampirovibrionia;o__Gastranaerophilales;f__Gastranaerophilaceae;g__;s__
TP001_bin.35	d__Bacteria;p__Bacteroidota;c__Bacteroidia;o__Bacteroidales;f__Bacteroidaceae;g__Prevotella;s__
TP001_bin.41	d__Bacteria;p__Campylobacterota;c__Campylobacteria;o__Campylobacterales;f__Helicobacteraceae;g__Helicobacter_F;s__Helicobacter_F sp003288805
TP001_bin.56	d__Bacteria;p__Firmicutes_A;c__Clostridia;o__Lachnospirales;f__Lachnospiraceae;g__COE1;s__
TP001_bin.90	d__Bacteria;p__Firmicutes_A;c__Clostridia;o__Lachnospirales;f__Lachnospiraceae;g__CAG-510;s__
TP002_bin.105	d__Bacteria;p__Bacteroidota;c__Bacteroidia;o__Bacteroidales;f__Paludibacteraceae;g__RF16;s__
TP002_bin.106	d__Bacteria;p__Proteobacteria;c__Alphaproteobacteria;o__RF32;f__CAG-239;g__CAG-267;s__
TP002_bin.136	d__Bacteria;p__Firmicutes_A;c__Clostridia;o__Oscillospirales;f__Ruminococcaceae;g__Ruminiclostridium_E;s__
TP002_bin.148	d__Bacteria;p__Firmicutes_A;c__Clostridia;o__Oscillospirales;f__Acutalibacteraceae;g__CAG-180;s__
TP002_bin.164	d__Bacteria;p__Firmicutes_A;c__Clostridia_A;o__Christensenellales;f__CAG-74;g__SFFH01;s__
TP002_bin.4	d__Bacteria;p__Firmicutes_A;c__Clostridia;o__Lachnospirales;f__Lachnospiraceae;g__CAG-127;s__
TP002_bin.67	d__Bacteria;p__Firmicutes_A;c__Clostridia;o__Oscillospirales;f__CAG-272;g__Firm-07;s__
TP002_bin.70	d__Bacteria;p__Firmicutes_A;c__Clostridia;o__Lachnospirales;f__Lachnospiraceae;g__Acetatifactor;s__
TP002_bin.75	d__Bacteria;p__Firmicutes_A;c__Clostridia;o__Oscillospirales;f__Ruminococcaceae;g__CAG-353;s__
TP002_bin.8	d__Bacteria;p__Firmicutes_A;c__Clostridia;o__Lachnospirales;f__Lachnospiraceae;g__CAG-127;s__
TP002_bin.84	d__Bacteria;p__Firmicutes;c__Bacilli;o__RFN20;f__CAG-826;g__UBA733;s__
TP002_bin.87	d__Bacteria;p__Firmicutes_A;c__Clostridia;o__Lachnospirales;f__Lachnospiraceae;g__;s__
TP003_bin.127	d__Bacteria;p__Spirochaetota;c__Spirochaetia;o__Treponematales;f__Treponemataceae;g__Treponema_D;s__
TP003_bin.65	d__Bacteria;p__Firmicutes;c__Bacilli;o__Mycoplasmatales;f__Metamycoplasmataceae;g__UBA710;s__
TP003MC_bin.114	d__Bacteria;p__Bacteroidota;c__Bacteroidia;o__Bacteroidales;f__Paludibacteraceae;g__RF16;s__
TP003MC_bin.120	d__Bacteria;p__Bacteroidota;c__Bacteroidia;o__Bacteroidales;f__Bacteroidaceae;g__UBA4334;s__
TP003MC_bin.13	d__Bacteria;p__Firmicutes_A;c__Clostridia;o__Lachnospirales;f__Lachnospiraceae;g__Agathobacter;s__
TP004MC_bin.13	d__Bacteria;p__Bacteroidota;c__Bacteroidia;o__Bacteroidales;f__Bacteroidaceae;g__Phocaeicola;s__Phocaeicola plebeius_B
TP004MC_bin.38	d__Bacteria;p__Firmicutes;c__Bacilli;o__Acholeplasmatales;f__Anaeroplasmataceae;g__Anaeroplasma;s__
TP004MC_bin.41	d__Bacteria;p__Proteobacteria;c__Alphaproteobacteria;o__RF32;f__CAG-239;g__CAG-267;s__
TP004MC_bin.63	d__Bacteria;p__Firmicutes_A;c__Clostridia;o__Lachnospirales;f__Lachnospiraceae;g__Agathobacter;s__
TP004MC_bin.66	d__Bacteria;p__Firmicutes;c__Bacilli;o__Acholeplasmatales;f__Anaeroplasmataceae;g__UBA3206;s__
TP004MC_bin.67	d__Bacteria;p__Elusimicrobiota;c__Elusimicrobia;o__Elusimicrobiales;f__Elusimicrobiaceae;g__UBA1436;s__
TP004MC_bin.86	d__Bacteria;p__Firmicutes_A;c__Clostridia;o__Lachnospirales;f__Lachnospiraceae;g__CAG-882;s__
TP005_bin.147	d__Bacteria;p__Firmicutes_A;c__Clostridia;o__Lachnospirales;f__Lachnospiraceae;g__Agathobacter;s__
TP005_bin.3	d__Bacteria;p__Firmicutes_A;c__Clostridia;o__Oscillospirales;f__Oscillospiraceae;g__;s__
TP005_bin.59	d__Bacteria;p__Firmicutes_A;c__Clostridia;o__Oscillospirales;f__Butyricicoccaceae;g__Butyricicoccus;s__Butyricicoccus sp004552245
TP005_bin.79	d__Bacteria;p__Firmicutes_A;c__Clostridia;o__Oscillospirales;f__Ruminococcaceae;g__SFJ001;s__SFJ001 sp004555865
TP005_bin.95	d__Bacteria;p__Firmicutes_A;c__Clostridia;o__Oscillospirales;f__Ruminococcaceae;g__Gemmiger;s__Gemmiger sp004555405
TP005MC_bin.38	d__Bacteria;p__Firmicutes_A;c__Clostridia_A;o__Christensenellales;f__Borkfalkiaceae;g__;s__
TP005MC_bin.82	d__Bacteria;p__Firmicutes_A;c__Clostridia;o__Oscillospirales;f__Acutalibacteraceae;g__UBA737;s__
TP006MC_bin.121	d__Bacteria;p__Firmicutes_A;c__Clostridia_A;o__Christensenellales;f__Borkfalkiaceae;g__UBA1259;s__
TP006MC_bin.130	d__Bacteria;p__Firmicutes_A;c__Clostridia;o__Lachnospirales;f__Lachnospiraceae;g__CAG-95;s__
TP006MC_bin.14	d__Bacteria;p__Verrucomicrobiota;c__Kiritimatiellae;o__RFP12;f__UBA1067;g__UBA1067;s__
TP006MC_bin.52	d__Bacteria;p__Proteobacteria;c__Alphaproteobacteria;o__RF32;f__CAG-239;g__CAG-267;s__
TP006MC_bin.74	d__Bacteria;p__Verrucomicrobiota;c__Verrucomicrobiae;o__Verrucomicrobiales;f__Akkermansiaceae;g__Akkermansia;s__
TP007MC_bin.110	d__Bacteria;p__Firmicutes_A;c__Clostridia;o__Oscillospirales;f__Oscillospiraceae;g__CAG-110;s__
TP007MC_bin.123	d__Bacteria;p__Firmicutes_A;c__Clostridia;o__Lachnospirales;f__Lachnospiraceae;g__COE1;s__
TP007MC_bin.132	d__Bacteria;p__Bacteroidota;c__Bacteroidia;o__Bacteroidales;f__Paludibacteraceae;g__RF16;s__

SGBs ID	Classification
TP007MC_bin.20	d__Bacteria;p__Firmicutes_A;c__Clostridia_A;o__Christensenellales;f__CAG-74;g__UBA11524;s__
TP007MC_bin.49	d__Bacteria;p__Cyanobacteria;c__Vampirovibrionia;o__Gastranaerophilales;f__Gastranaerophilaceae;g__Zag1;s__
TP007MC_bin.50	d__Bacteria;p__Bacteroidota;c__Bacteroidia;o__Bacteroidales;f__UBA932;g__RC9;s__
TP007MC_bin.55	d__Bacteria;p__Bacteroidota;c__Bacteroidia;o__Bacteroidales;f__Bacteroidaceae;g__Prevotella;s__
TP008_bin.11	d__Bacteria;p__Bacteroidota;c__Bacteroidia;o__Bacteroidales;f__UBA932;g__RC9;s__
TP008_bin.114	d__Bacteria;p__Bacteroidota;c__Bacteroidia;o__Bacteroidales;f__Bacteroidaceae;g__;s__
TP008_bin.61	d__Bacteria;p__Bacteroidota;c__Bacteroidia;o__Bacteroidales;f__Muribaculaceae;g__;s__
TP008_bin.78	d__Bacteria;p__Firmicutes_A;c__Clostridia;o__Oscillospirales;f__Ruminococcaceae;g__HUN007;s__
TP008MC_bin.100	d__Bacteria;p__Verrucomicrobiota;c__Kiritimatiellae;o__RFP12;f__UBA1067;g__UBA1067;s__
TP008MC_bin.139	d__Bacteria;p__Bacteroidota;c__Bacteroidia;o__Bacteroidales;f__Bacteroidaceae;g__Prevotellamassilia;s__
TP008MC_bin.31	d__Bacteria;p__Bacteroidota;c__Bacteroidia;o__Bacteroidales;f__Paludibacteraceae;g__RF16;s__
TP008MC_bin.33	d__Bacteria;p__Bacteroidota;c__Bacteroidia;o__Bacteroidales;f__Bacteroidaceae;g__Prevotella;s__
TP008MC_bin.72	d__Bacteria;p__Firmicutes_A;c__Clostridia;o__Lachnospirales;f__Lachnospiraceae;g__COE1;s__
TP008MC_bin.8	d__Bacteria;p__Bacteroidota;c__Bacteroidia;o__Bacteroidales;f__UBA932;g__RC9;s__
TP008MC_bin.81	d__Bacteria;p__Bacteroidota;c__Bacteroidia;o__Bacteroidales;f__Bacteroidaceae;g__Bacteroides;s__
TP008MC_bin.88	d__Bacteria;p__Bacteroidota;c__Bacteroidia;o__Bacteroidales;f__Bacteroidaceae;g__UBA4372;s__
TP009_bin.103	d__Bacteria;p__Firmicutes_A;c__Clostridia;o__Lachnospirales;f__Lachnospiraceae;g__CAG-510;s__
TP009_bin.25	d__Bacteria;p__Firmicutes_A;c__Clostridia;o__Oscillospirales;f__Acutalibacteraceae;g__CAG-180;s__
TP009_bin.28	d__Bacteria;p__Cyanobacteria;c__Vampirovibrionia;o__Gastranaerophilales;f__Gastranaerophilaceae;g__CAG-196;s__
TP009_bin.4	d__Bacteria;p__Verrucomicrobiota;c__Verrucomicrobiae;o__Verrucomicrobiales;f__Akkermansiaceae;g__Akkermansia;s__
TP009_bin.41	d__Bacteria;p__Firmicutes_A;c__Clostridia;o__Lachnospirales;f__Lachnospiraceae;g__Agathobacter;s__
TP009_bin.49	d__Bacteria;p__Firmicutes_A;c__Clostridia;o__Lachnospirales;f__Lachnospiraceae;g__COE1;s__
TP009_bin.66	d__Bacteria;p__Firmicutes_A;c__Clostridia;o__Lachnospirales;f__Lachnospiraceae;g__CAG-95;s__
TP010_bin.100	d__Bacteria;p__Firmicutes_A;c__Clostridia;o__Lachnospirales;f__Lachnospiraceae;g__UBA2868;s__
TP010_bin.119	d__Bacteria;p__Firmicutes;c__Bacilli;o__Lactobacillales;f__Streptococcaceae;g__Streptococcus;s__Streptococcus sp002300045
TP010_bin.76	d__Bacteria;p__Bacteroidota;c__Bacteroidia;o__Bacteroidales;f__Bacteroidaceae;g__Prevotella;s__
TP011_bin.101	d__Bacteria;p__Firmicutes_A;c__Clostridia;o__Lachnospirales;f__Lachnospiraceae;g__;s__
TP011_bin.135	d__Bacteria;p__Spirochaetota;c__Spirochaetia;o__Treponematales;f__Treponemataceae;g__Treponema_D;s__
TP011_bin.6	d__Bacteria;p__Bacteroidota;c__Bacteroidia;o__Bacteroidales;f__Paludibacteraceae;g__UBA4363;s__
TP011_bin.67	d__Bacteria;p__Firmicutes_A;c__Clostridia;o__Oscillospirales;f__Ruminococcaceae;g__;s__
TP011_bin.84	d__Bacteria;p__Bacteroidota;c__Bacteroidia;o__Bacteroidales;f__Muribaculaceae;g__UBA7173;s__
TP011_bin.85	d__Bacteria;p__Firmicutes_A;c__Clostridia;o__Oscillospirales;f__Ruminococcaceae;g__Ruminococcus_C;s__
TP011_bin.90	d__Bacteria;p__Bacteroidota;c__Bacteroidia;o__Bacteroidales;f__Paludibacteraceae;g__RF16;s__
TP011_bin.97	d__Bacteria;p__Bacteroidota;c__Bacteroidia;o__Bacteroidales;f__Bacteroidaceae;g__UBA4372;s__
TP012_bin.137	d__Bacteria;p__Bacteroidota;c__Bacteroidia;o__Bacteroidales;f__Bacteroidaceae;g__Prevotellamassilia;s__
TP012_bin.29	d__Bacteria;p__Firmicutes;c__Bacilli;o__RFN20;f__CAG-826;g__;s__
TP012_bin.3	d__Bacteria;p__Verrucomicrobiota;c__Verrucomicrobiae;o__Verrucomicrobiales;f__Akkermansiaceae;g__Akkermansia;s__
TP012_bin.39	d__Bacteria;p__Fibrobacterota;c__Fibrobacteria;o__Fibrobacterales;f__Fibrobacteraceae;g__Fibrobacter_A;s__Fibrobacter_A sp002797675
TP012_bin.49	d__Bacteria;p__Spirochaetota;c__Spirochaetia;o__Treponematales;f__Treponemataceae;g__Treponema_D;s__
TP012_bin.56	d__Bacteria;p__Firmicutes_A;c__Clostridia_A;o__Christensenellales;f__CAG-74;g__UBA11524;s__
TP012_bin.62	d__Bacteria;p__Firmicutes_A;c__Clostridia;o__Oscillospirales;f__Acutalibacteraceae;g__;s__
TP012_bin.66	d__Bacteria;p__Proteobacteria;c__Alphaproteobacteria;o__RF32;f__CAG-977;g__UBA3864;s__UBA3864 sp002393065
TP012_bin.84	d__Bacteria;p__Firmicutes_A;c__Clostridia;o__Oscillospirales;f__Oscillospiraceae;g__Oscillibacter;s__
TP012_bin.9	d__Bacteria;p__Firmicutes_A;c__Clostridia;o__Oscillospirales;f__Oscillospiraceae;g__CAG-110;s__
TP012MC_bin.25	d__Bacteria;p__Proteobacteria;c__Alphaproteobacteria;o__RF32;f__CAG-239;g__CAG-267;s__
TP012MC_bin.71	d__Bacteria;p__Firmicutes_A;c__Clostridia;o__Lachnospirales;f__Lachnospiraceae;g__;s__
TP012MC_bin.85	d__Bacteria;p__Firmicutes_A;c__Clostridia;o__Lachnospirales;f__Lachnospiraceae;g__CAG-882;s__
TP014_bin.24	d__Bacteria;p__Bacteroidota;c__Bacteroidia;o__Bacteroidales;f__Bacteroidaceae;g__Prevotella;s__
TP014_bin.35	d__Bacteria;p__Proteobacteria;c__Alphaproteobacteria;o__RF32;f__CAG-239;g__RUG410;s__
TP014_bin.39	d__Bacteria;p__Bacteroidota;c__Bacteroidia;o__Bacteroidales;f__Bacteroidaceae;g__Prevotella;s__
TP014_bin.67	d__Bacteria;p__Proteobacteria;c__Alphaproteobacteria;o__RF32;f__CAG-239;g__CAG-495;s__CAG-495 sp000436375
TP014_bin.7	d__Bacteria;p__Firmicutes_A;c__Clostridia;o__TANB77;f__CAG-508;g__;s__
TP014_bin.70	d__Bacteria;p__Campylobacterota;c__Campylobacteria;o__Campylobacterales;f__Campylobacteraceae;g__Campylobacter;s__Campylobacter sp002139855
TP014_bin.83	d__Bacteria;p__Bacteroidota;c__Bacteroidia;o__Bacteroidales;f__Bacteroidaceae;g__Prevotella;s__
TP014MC_bin.156	d__Bacteria;p__Firmicutes_C;c__Negativicutes;o__Selenomonadales;f__Selenomonadaceae;g__Anaerovibrio;s__
TP014MC_bin.61	d__Bacteria;p__Bacteroidota;c__Bacteroidia;o__Bacteroidales;f__UBA932;g__RC9;s__
TP014MC_bin.66	d__Bacteria;p__Firmicutes_A;c__Clostridia_A;o__Christensenellales;f__CAG-74;g__;s__
TP014MC_bin.90	d__Bacteria;p__Bacteroidota;c__Bacteroidia;o__Bacteroidales;f__Bacteroidaceae;g__F0040;s__
TP015_bin.102	d__Bacteria;p__Firmicutes_A;c__Clostridia;o__Oscillospirales;f__Oscillospiraceae;g__CAG-110;s__
TP015_bin.103	d__Bacteria;p__Firmicutes_A;c__Clostridia_A;o__Christensenellales;f__CAG-74;g__;s__
TP015_bin.111	d__Bacteria;p__Firmicutes_A;c__Clostridia;o__Lachnospirales;f__Lachnospiraceae;g__COE1;s__
TP015_bin.30	d__Bacteria;p__Proteobacteria;c__Alphaproteobacteria;o__RF32;f__CAG-239;g__CAG-267;s__
TP015_bin.34	d__Bacteria;p__Bacteroidota;c__Bacteroidia;o__Bacteroidales;f__Bacteroidaceae;g__Bacteroides;s__
TP015_bin.39	d__Bacteria;p__Bacteroidota;c__Bacteroidia;o__Bacteroidales;f__UBA3663;g__UBA3663;s__
TP015_bin.88	d__Bacteria;p__Firmicutes_A;c__Clostridia;o__Lachnospirales;f__Lachnospiraceae;g__Schaedlerella;s__
TP015MC_bin.116	d__Bacteria;p__Firmicutes_A;c__Clostridia;o__Lachnospirales;f__Lachnospiraceae;g__Eubacterium_J;s__
TP015MC_bin.135	d__Bacteria;p__Proteobacteria;c__Alphaproteobacteria;o__RF32;f__UBA3637;g__;s__
TP015MC_bin.18	d__Bacteria;p__Firmicutes_A;c__Clostridia;o__Lachnospirales;f__Lachnospiraceae;g__Acetatifactor;s__
TP015MC_bin.36	d__Bacteria;p__Proteobacteria;c__Alphaproteobacteria;o__RF32;f__CAG-239;g__RUG410;s__
TP015MC_bin.37	d__Bacteria;p__Cyanobacteria;c__Vampirovibrionia;o__Gastranaerophilales;f__Gastranaerophilaceae;g__CAG-196;s__
TP015MC_bin.54	d__Bacteria;p__Bacteroidota;c__Bacteroidia;o__Bacteroidales;f__Paludibacteraceae;g__RF16;s__
TP015MC_bin.62	d__Bacteria;p__Firmicutes_A;c__Clostridia;o__Lachnospirales;f__Lachnospiraceae;g__Eubacterium_J;s__
TP015MC_bin.75	d__Bacteria;p__Bacteroidota;c__Bacteroidia;o__Bacteroidales;f__UBA932;g__RC9;s__
TP015MC_bin.76	d__Bacteria;p__Firmicutes_A;c__Clostridia;o__Lachnospirales;f__Lachnospiraceae;g__CAG-95;s__
TP015MC_bin.77	d__Bacteria;p__Bacteroidota;c__Bacteroidia;o__Bacteroidales;f__Paludibacteraceae;g__RF16;s__
TP015MC_bin.87	d__Bacteria;p__Proteobacteria;c__Gammaproteobacteria;o__Enterobacterales;f__Succinivibrionaceae;g__Ruminobacter;s__
TP017MC_bin.126	d__Bacteria;p__Bacteroidota;c__Bacteroidia;o__Bacteroidales;f__UBA932;g__RC9;s__
TP017MC_bin.2	d__Bacteria;p__Firmicutes_A;c__Clostridia;o__UMGS1883;f__;g__;s__
TP017MC_bin.98	d__Bacteria;p__Bacteroidota;c__Bacteroidia;o__Bacteroidales;f__Bacteroidaceae;g__Paraprevotella;s__
TP018_bin.101	d__Bacteria;p__Firmicutes_A;c__Clostridia;o__Lachnospirales;f__Lachnospiraceae;g__Acetatifactor;s__
TP018_bin.105	d__Bacteria;p__Verrucomicrobiota;c__Verrucomicrobiae;o__Verrucomicrobiales;f__Akkermansiaceae;g__Akkermansia;s__
TP018_bin.180	d__Bacteria;p__Firmicutes_A;c__Clostridia;o__Lachnospirales;f__Lachnospiraceae;g__;s__
TP018_bin.26	d__Bacteria;p__Firmicutes_C;c__Negativicutes;o__Selenomonadales;f__Selenomonadaceae;g__Anaerovibrio;s__
TP018_bin.55	d__Bacteria;p__Firmicutes_A;c__Clostridia;o__Lachnospirales;f__Lachnospiraceae;g__Coprococcus;s__
TP018_bin.78	d__Bacteria;p__Cyanobacteria;c__Vampirovibrionia;o__Gastranaerophilales;f__Gastranaerophilaceae;g__Zag111;s__
TP018_bin.85	d__Bacteria;p__Firmicutes_A;c__Clostridia;o__Lachnospirales;f__Lachnospiraceae;g__;s__
TP018MC_bin.102	d__Bacteria;p__Bacteroidota;c__Bacteroidia;o__Bacteroidales;f__Bacteroidaceae;g__Prevotellamassilia;s__
TP018MC_bin.11	d__Bacteria;p__Proteobacteria;c__Gammaproteobacteria;o__Burkholderiales;f__Burkholderiaceae;g__Sutterella;s__
TP018MC_bin.115	d__Bacteria;p__Actinobacteriota;c__Actinomycetia;o__Actinomycetales;f__Bifidobacteriaceae;g__Bifidobacterium;s__Bifidobacterium thermophilum

SGBs ID	Classification
TP018MC_bin.143	d__Bacteria;p__Firmicutes_A;c__Clostridia;o__Oscillospirales;f__CAG-272;g__CAG-448;s__
TP018MC_bin.156	d__Bacteria;p__Proteobacteria;c__Alphaproteobacteria;o__Rs-D84;f__Rs-D84;g__Rs-D84;s__
TP018MC_bin.157	d__Bacteria;p__Firmicutes_A;c__Clostridia_A;o__Christensenellales;f__Borkfalkiaceae;g__;s__
TP018MC_bin.177	d__Bacteria;p__Elusimicrobiota;c__Elusimicrobia;o__Elusimicrobiales;f__Elusimicrobiaceae;g__UBA1436;s__
TP018MC_bin.185	d__Bacteria;p__Proteobacteria;c__Alphaproteobacteria;o__RF32;f__CAG-239;g__CAG-495;s__
TP018MC_bin.51	d__Bacteria;p__Bacteroidota;c__Bacteroidia;o__Bacteroidales;f__Bacteroidaceae;g__Prevotella;s__
TP018MC_bin.69	d__Bacteria;p__Firmicutes_A;c__Clostridia;o__Lachnospirales;f__Lachnospiraceae;g__Acetatifactor;s__
TP018MC_bin.74	d__Bacteria;p__Firmicutes_A;c__Clostridia;o__Lachnospirales;f__Lachnospiraceae;g__Acetatifactor;s__
TP018MC_bin.85	d__Bacteria;p__Firmicutes_A;c__Clostridia_A;o__Christensenellales;f__CAG-74;g__UBA11524;s__
TP019MC_bin.103	d__Bacteria;p__Firmicutes_A;c__Clostridia;o__Oscillospirales;f__CAG-272;g__UMGS1002;s__
TP019MC_bin.30	d__Bacteria;p__Verrucomicrobiota;c__Kiritimatiellae;o__RFP12;f__UBA1067;g__RUG572;s__
TP019MC_bin.8	d__Bacteria;p__Firmicutes_A;c__Clostridia;o__Oscillospirales;f__Oscillospiraceae;g__Oscillibacter;s__
TP019MC_bin.9	d__Bacteria;p__Firmicutes_A;c__Clostridia;o__Oscillospirales;f__Oscillospiraceae;g__CAG-110;s__
TP019MC_bin.95	d__Bacteria;p__Bacteroidota;c__Bacteroidia;o__Bacteroidales;f__Barnesiellaceae;g__Barnesiella;s__Barnesiella sp900538555
TP020_bin.103	d__Bacteria;p__Bacteroidota;c__Bacteroidia;o__Bacteroidales;f__Bacteroidaceae;g__Prevotellamassilia;s__
TP020_bin.111	d__Bacteria;p__Bacteroidota;c__Bacteroidia;o__Bacteroidales;f__Bacteroidaceae;g__Prevotella;s__Prevotella copri_A
TP020_bin.122	d__Bacteria;p__Bacteroidota;c__Bacteroidia;o__Bacteroidales;f__Muribaculaceae;g__CAG-873;s__CAG-873 sp004554255
TP020_bin.23	d__Bacteria;p__Bacteroidota;c__Bacteroidia;o__Bacteroidales;f__UBA932;g__RC9;s__
TP020_bin.26	d__Bacteria;p__Proteobacteria;c__Gammaproteobacteria;o__Enterobacterales;f__Succinivibrionaceae;g__Succinivibrio;s__Succinivibrio sp900316375
TP020_bin.5	d__Bacteria;p__Bacteroidota;c__Bacteroidia;o__Bacteroidales;f__Rikenellaceae;g__Alistipes;s__Alistipes sp900548155
TP020MC_bin.105	d__Bacteria;p__Firmicutes_A;c__Clostridia_A;o__Christensenellales;f__Borkfalkiaceae;g__;s__
TP020MC_bin.144	d__Bacteria;p__Firmicutes;c__Bacilli;o__RF39;f__CAG-1000;g__UBA11963;s__
TP020MC_bin.157	d__Bacteria;p__Bacteroidota;c__Bacteroidia;o__Bacteroidales;f__UBA932;g__RC9;s__
TP020MC_bin.23	d__Bacteria;p__Firmicutes_A;c__Clostridia_A;o__Christensenellales;f__Borkfalkiaceae;g__UBA11940;s__
TP020MC_bin.75	d__Bacteria;p__Firmicutes_A;c__Clostridia;o__Lachnospirales;f__Lachnospiraceae;g__Acetatifactor;s__
TP021_bin.103	d__Bacteria;p__Proteobacteria;c__Alphaproteobacteria;o__RF32;f__CAG-239;g__RUG410;s__
TP021_bin.106	d__Bacteria;p__Bacteroidota;c__Bacteroidia;o__Bacteroidales;f__Bacteroidaceae;g__Phocaeicola;s__
TP021_bin.115	d__Bacteria;p__Proteobacteria;c__Alphaproteobacteria;o__Rs-D84;f__;g__;s__
TP021_bin.119	d__Bacteria;p__Proteobacteria;c__Alphaproteobacteria;o__RF32;f__CAG-239;g__CAG-495;s__
TP021_bin.140	d__Bacteria;p__Bacteroidota;c__Bacteroidia;o__Bacteroidales;f__UBA932;g__RC9;s__
TP021_bin.147	d__Bacteria;p__Firmicutes_A;c__Clostridia;o__Oscillospirales;f__Ruminococcaceae;g__Faecalibacterium;s__Faecalibacterium prausnitzii_G
TP021_bin.156	d__Bacteria;p__Bacteroidota;c__Bacteroidia;o__Bacteroidales;f__UBA932;g__RC9;s__
TP021_bin.28	d__Bacteria;p__Spirochaetota;c__Spirochaetia;o__Treponematales;f__Treponemataceae;g__Treponema_D;s__
TP021_bin.45	d__Bacteria;p__Firmicutes_A;c__Clostridia;o__Oscillospirales;f__Acutalibacteraceae;g__CAG-180;s__
TP021_bin.51	d__Bacteria;p__Proteobacteria;c__Alphaproteobacteria;o__RF32;f__CAG-239;g__CAG-495;s__
TP021_bin.55	d__Bacteria;p__Bacteroidota;c__Bacteroidia;o__Bacteroidales;f__UBA932;g__RC9;s__
TP021_bin.56	d__Bacteria;p__Firmicutes_A;c__Clostridia;o__Lachnospirales;f__Lachnospiraceae;g__CAG-127;s__
TP021_bin.7	d__Bacteria;p__Firmicutes_A;c__Clostridia;o__Lachnospirales;f__Lachnospiraceae;g__CAG-95;s__
TP021_bin.93	d__Bacteria;p__Firmicutes;c__Bacilli;o__RF39;f__CAG-1000;g__CAG-582;s__
TP021MC_bin.139	d__Bacteria;p__Proteobacteria;c__Alphaproteobacteria;o__RF32;f__CAG-239;g__CAG-495;s__
TP021MC_bin.45	d__Bacteria;p__Bacteroidota;c__Bacteroidia;o__Bacteroidales;f__Bacteroidaceae;g__F0040;s__
TP021MC_bin.53	d__Bacteria;p__Bacteroidota;c__Bacteroidia;o__Bacteroidales;f__W3P20-009;g__W3P20-009;s__
TP021MC_bin.71	d__Bacteria;p__Bacteroidota;c__Bacteroidia;o__Bacteroidales;f__UBA932;g__RC9;s__
TP021MC_bin.72	d__Bacteria;p__Firmicutes_A;c__Clostridia;o__Oscillospirales;f__Acutalibacteraceae;g__CAG-180;s__
TP022_bin.117	d__Bacteria;p__Firmicutes_A;c__Clostridia;o__Lachnospirales;f__Lachnospiraceae;g__CAG-194;s__
TP022_bin.99	d__Bacteria;p__Firmicutes_A;c__Clostridia;o__Oscillospirales;f__Ruminococcaceae;g__CAG-115;s__
TP022MC_bin.127	d__Bacteria;p__Proteobacteria;c__Alphaproteobacteria;o__RF32;f__CAG-239;g__CAG-495;s__
TP022MC_bin.16	d__Bacteria;p__Bacteroidota;c__Bacteroidia;o__Bacteroidales;f__Paludibacteraceae;g__RF16;s__
TP022MC_bin.63	d__Bacteria;p__Firmicutes_A;c__Clostridia;o__Lachnospirales;f__Lachnospiraceae;g__CAG-882;s__
TP022MC_bin.70	d__Bacteria;p__Spirochaetota;c__Spirochaetia;o__Treponematales;f__Treponemataceae;g__Treponema_D;s__
TP023_bin.58	d__Bacteria;p__Firmicutes_A;c__Clostridia;o__Oscillospirales;f__Ruminococcaceae;g__Faecalibacterium;s__Faecalibacterium sp003449675
TP023_bin.70	d__Bacteria;p__Firmicutes_A;c__Clostridia;o__Lachnospirales;f__Lachnospiraceae;g__UBA7050;s__
TP023_bin.85	d__Bacteria;p__Bacteroidota;c__Bacteroidia;o__Bacteroidales;f__Paludibacteraceae;g__RF16;s__
TP024_bin.106	d__Bacteria;p__Bacteroidota;c__Bacteroidia;o__Bacteroidales;f__Bacteroidaceae;g__Prevotella;s__
TP024_bin.115	d__Bacteria;p__Firmicutes_A;c__Clostridia;o__Lachnospirales;f__Lachnospiraceae;g__CAG-590;s__
TP024_bin.125	d__Bacteria;p__Spirochaetota;c__Spirochaetia;o__Treponematales;f__Treponemataceae;g__Treponema_D;s__
TP024_bin.130	d__Bacteria;p__Firmicutes_A;c__Clostridia;o__Oscillospirales;f__Ruminococcaceae;g__;s__
TP024_bin.19	d__Bacteria;p__Campylobacterota;c__Campylobacteria;o__Campylobacterales;f__Helicobacteraceae;g__Helicobacter_D;s__
TP024_bin.56	d__Bacteria;p__Firmicutes_A;c__Clostridia_A;o__Christensenellales;f__Borkfalkiaceae;g__;s__
TP024_bin.91	d__Bacteria;p__Firmicutes_A;c__Clostridia;o__Lachnospirales;f__Lachnospiraceae;g__UBA7050;s__
TP024MC_bin.118	d__Bacteria;p__Firmicutes_A;c__Clostridia;o__Lachnospirales;f__Lachnospiraceae;g__Acetatifactor;s__
TP024MC_bin.130	d__Bacteria;p__Firmicutes_A;c__Clostridia;o__Lachnospirales;f__Lachnospiraceae;g__Agathobacter;s__
TP024MC_bin.131	d__Bacteria;p__Bacteroidota;c__Bacteroidia;o__Bacteroidales;f__Bacteroidaceae;g__Prevotella;s__
TP024MC_bin.53	d__Bacteria;p__Spirochaetota;c__Spirochaetia;o__Sphaerochaetales;f__Sphaerochaetaceae;g__UBA5920;s__
TP024MC_bin.61	d__Bacteria;p__Riflebacteria;c__Ozemobacteria;o__Ozemobacterales;f__Ozemobacteraceae;g__RUG334;s__
TP025_bin.106	d__Bacteria;p__Firmicutes_A;c__Clostridia_A;o__Christensenellales;f__Borkfalkiaceae;g__;s__
TP025_bin.115	d__Bacteria;p__Firmicutes_A;c__Clostridia;o__Lachnospirales;f__Lachnospiraceae;g__CAG-510;s__
TP025_bin.117	d__Bacteria;p__Firmicutes_A;c__Clostridia;o__Lachnospirales;f__Lachnospiraceae;g__TWA4;s__
TP025_bin.120	d__Bacteria;p__Firmicutes;c__Bacilli;o__RF39;f__CAG-822;g__UMGS1045;s__
TP025_bin.136	d__Bacteria;p__Firmicutes_A;c__Clostridia;o__Oscillospirales;f__Oscillospiraceae;g__UBA1777;s__
TP025_bin.138	d__Bacteria;p__Proteobacteria;c__Gammaproteobacteria;o__Burkholderiales;f__SFHR01;g__SFHR01;s__SFHR01 sp004555545
TP025_bin.139	d__Bacteria;p__Firmicutes_A;c__Clostridia;o__Oscillospirales;f__Oscillospiraceae;g__CAG-110;s__
TP025_bin.142	d__Bacteria;p__Bacteroidota;c__Bacteroidia;o__Bacteroidales;f__Paludibacteraceae;g__RF16;s__
TP025_bin.18	d__Bacteria;p__Proteobacteria;c__Gammaproteobacteria;o__Burkholderiales;f__Burkholderiaceae;g__Sutterella;s__
TP025_bin.31	d__Bacteria;p__Firmicutes_A;c__Clostridia;o__Oscillospirales;f__Ruminococcaceae;g__Ruminiclostridium_E;s__
TP025_bin.34	d__Bacteria;p__Firmicutes_A;c__Clostridia_A;o__Christensenellales;f__CAG-74;g__SFFH01;s__
TP025_bin.45	d__Bacteria;p__Bacteroidota;c__Bacteroidia;o__Bacteroidales;f__Paludibacteraceae;g__RF16;s__
TP025_bin.59	d__Bacteria;p__Firmicutes_A;c__Clostridia;o__Oscillospirales;f__Ruminococcaceae;g__CAG-115;s__
TP025_bin.6	d__Bacteria;p__Elusimicrobiota;c__Elusimicrobia;o__Elusimicrobiales;f__Elusimicrobiaceae;g__;s__
TP025_bin.93	d__Bacteria;p__Firmicutes_A;c__Clostridia;o__Oscillospirales;f__Acutalibacteraceae;g__CAG-180;s__
TP025_bin.98	d__Bacteria;p__Verrucomicrobiota;c__Kiritimatiellae;o__RFP12;f__UBA1067;g__UBA1067;s__
TP025MC_bin.148	d__Bacteria;p__Verrucomicrobiota;c__Kiritimatiellae;o__RFP12;f__UBA1067;g__UBA1067;s__
TP025MC_bin.63	d__Bacteria;p__Firmicutes_A;c__Clostridia;o__Lachnospirales;f__Lachnospiraceae;g__Agathobacter;s__
TP026_bin.108	d__Bacteria;p__Proteobacteria;c__Alphaproteobacteria;o__RF32;f__CAG-239;g__RUG410;s__
TP026_bin.115	d__Bacteria;p__Verrucomicrobiota;c__Verrucomicrobiae;o__Opitutales;f__UBA9783;g__UBA1183;s__
TP026_bin.154	d__Bacteria;p__Proteobacteria;c__Alphaproteobacteria;o__Rs-D84;f__Rs-D84;g__Rs-D84;s__
TP026_bin.65	d__Bacteria;p__Bacteroidota;c__Bacteroidia;o__Bacteroidales;f__Bacteroidaceae;g__Prevotella;s__
TP026_bin.87	d__Bacteria;p__Firmicutes_A;c__Clostridia_A;o__Christensenellales;f__Borkfalkiaceae;g__;s__
TP027_bin.100	d__Bacteria;p__Bacteroidota;c__Bacteroidia;o__Bacteroidales;f__Bacteroidaceae;g__Prevotella;s__

SGBs ID	Classification
TP027_bin.147	d__Bacteria;p__Bacteroidota;c__Bacteroidia;o__Bacteroidales;f__Muribaculaceae;g__;s__
TP027_bin.153	d__Bacteria;p__Bacteroidota;c__Bacteroidia;o__Bacteroidales;f__Bacteroidaceae;g__Bacteroides;s__
TP027_bin.157	d__Bacteria;p__Firmicutes_A;c__Clostridia;o__Oscillospirales;f__Ruminococcaceae;g__Ruminococcus_D;s__
TP027_bin.5	d__Bacteria;p__Firmicutes_A;c__Clostridia;o__Lachnospirales;f__Lachnospiraceae;g__Bacteroides_F;s__
TP027_bin.78	d__Bacteria;p__Firmicutes_A;c__Clostridia;o__Lachnospirales;f__Lachnospiraceae;g__Eubacterium_F;s__
TP027_bin.9	d__Bacteria;p__Firmicutes_A;c__Clostridia;o__Oscillospirales;f__Acutalibacteraceae;g__CAG-180;s__
TP027_bin.91	d__Bacteria;p__Firmicutes_C;c__Negativicutes;o__Acidaminococcales;f__Acidaminococcaceae;g__Phascolarctobacterium_A;s__Phascolarctobacterium_A sp004558595
TP027MC_bin.112	d__Bacteria;p__Firmicutes_A;c__Clostridia;o__Lachnospirales;f__Lachnospiraceae;g__Agathobacter;s__
TP027MC_bin.116	d__Bacteria;p__Spirochaetota;c__Spirochaetia;o__Treponematales;f__Treponemataceae;g__Treponema_D;s__Treponema_D berlinense
TP027MC_bin.12	d__Bacteria;p__Bacteroidota;c__Bacteroidia;o__Bacteroidales;f__Bacteroidaceae;g__UBA4372;s__
TP027MC_bin.147	d__Bacteria;p__Firmicutes_A;c__Clostridia;o__Lachnospirales;f__Lachnospiraceae;g__Eubacterium_J;s__
TP027MC_bin.153	d__Bacteria;p__Firmicutes_A;c__Clostridia_A;o__Christensenellales;f__Borkfalkiaceae;g__UBA7597;s__
TP027MC_bin.2	d__Bacteria;p__Firmicutes_A;c__Clostridia;o__Lachnospirales;f__Lachnospiraceae;g__;s__
TP027MC_bin.27	d__Bacteria;p__Proteobacteria;c__Alphaproteobacteria;o__RF32;f__CAG-239;g__CAG-495;s__
TP027MC_bin.38	d__Bacteria;p__Bacteroidota;c__Bacteroidia;o__Bacteroidales;f__Muribaculaceae;g__CAG-1031;s__CAG-1031 sp000431215
TP027MC_bin.89	d__Bacteria;p__Bacteroidota;c__Bacteroidia;o__Bacteroidales;f__Bacteroidaceae;g__Prevotella;s__
TP027MC_bin.96	d__Bacteria;p__Firmicutes_A;c__Clostridia;o__Oscillospirales;f__Acutalibacteraceae;g__CAG-180;s__
TP028_bin.145	d__Bacteria;p__Bacteroidota;c__Bacteroidia;o__Bacteroidales;f__Bacteroidaceae;g__Prevotellamassilia;s__
TP028_bin.30	d__Bacteria;p__Bacteroidota;c__Bacteroidia;o__Bacteroidales;f__Muribaculaceae;g__SFRY01;s__SFRY01 sp004562065
TP028_bin.51	d__Bacteria;p__Bacteroidota;c__Bacteroidia;o__Bacteroidales;f__UBA932;g__RC9;s__
TP028_bin.79	d__Bacteria;p__Bacteroidota;c__Bacteroidia;o__Bacteroidales;f__Bacteroidaceae;g__Prevotella;s__
TP029_bin.122	d__Bacteria;p__Firmicutes_A;c__Clostridia;o__Lachnospirales;f__Lachnospiraceae;g__Acetatifactor;s__
TP029_bin.128	d__Bacteria;p__Elusimicrobiota;c__Elusimicrobia;o__Elusimicrobiales;f__Elusimicrobiaceae;g__UBA1436;s__
TP029_bin.15	d__Bacteria;p__Fibrobacterota;c__Fibrobacteria;o__Fibrobacterales;f__Fibrobacteraceae;g__Fibrobacter;s__
TP029_bin.154	d__Bacteria;p__Bacteroidota;c__Bacteroidia;o__Bacteroidales;f__Bacteroidaceae;g__UBA4372;s__
TP029_bin.158	d__Bacteria;p__Spirochaetota;c__Spirochaetia;o__Treponematales;f__Treponemataceae;g__Treponema_F;s__
TP029_bin.169	d__Bacteria;p__Bacteroidota;c__Bacteroidia;o__Bacteroidales;f__UBA932;g__RC9;s__
TP029_bin.43	d__Bacteria;p__Bacteroidota;c__Bacteroidia;o__Bacteroidales;f__Paludibacteraceae;g__RF16;s__
TP029_bin.61	d__Bacteria;p__Firmicutes_A;c__Clostridia_A;o__Christensenellales;f__Borkfalkiaceae;g__QALS01;s__
TP029_bin.9	d__Bacteria;p__Proteobacteria;c__Alphaproteobacteria;o__Rs-D84;f__Rs-D84;g__Rs-D84;s__
TP029MC_bin.10	d__Bacteria;p__Firmicutes_A;c__Clostridia;o__Lachnospirales;f__Lachnospiraceae;g__Lachnospira;s__
TP029MC_bin.101	d__Bacteria;p__Firmicutes_A;c__Clostridia;o__Oscillospirales;f__UBA644;g__;s__
TP029MC_bin.102	d__Bacteria;p__Bacteroidota;c__Bacteroidia;o__Bacteroidales;f__Bacteroidaceae;g__Bacteroides;s__
TP029MC_bin.20	d__Bacteria;p__Firmicutes_A;c__Clostridia;o__Oscillospirales;f__Ruminococcaceae;g__Ruminiclostridium_E;s__
TP029MC_bin.42	d__Bacteria;p__Bacteroidota;c__Bacteroidia;o__Bacteroidales;f__Bacteroidaceae;g__Prevotella;s__
TP029MC_bin.50	d__Bacteria;p__Bacteroidota;c__Bacteroidia;o__Bacteroidales;f__UBA932;g__UBA1232;s__
TP029MC_bin.53	d__Bacteria;p__Firmicutes_A;c__Clostridia;o__Oscillospirales;f__Ruminococcaceae;g__CAG-115;s__
TP029MC_bin.85	d__Bacteria;p__Bacteroidota;c__Bacteroidia;o__Bacteroidales;f__Muribaculaceae;g__CAG-873;s__
TP029MC_bin.89	d__Bacteria;p__Bacteroidota;c__Bacteroidia;o__Bacteroidales;f__Rikenellaceae;g__Alistipes;s__
TP029MC_bin.9	d__Bacteria;p__Firmicutes_A;c__Clostridia;o__Lachnospirales;f__Lachnospiraceae;g__;s__
TP029MC_bin.90	d__Bacteria;p__Bacteroidota;c__Bacteroidia;o__Bacteroidales;f__Muribaculaceae;g__C941;s__
TP030MC_bin.109	d__Bacteria;p__Bacteroidota;c__Bacteroidia;o__Bacteroidales;f__Bacteroidaceae;g__Prevotellamassilia;s__
TP030MC_bin.27	d__Bacteria;p__Bacteroidota;c__Bacteroidia;o__Bacteroidales;f__Muribaculaceae;g__CAG-485;s__CAG-485 sp900321585
TP030MC_bin.3	d__Bacteria;p__Firmicutes;c__Bacilli;o__RF39;f__CAG-822;g__UBA5578;s__
TP030MC_bin.48	d__Bacteria;p__Firmicutes_A;c__Clostridia;o__Oscillospirales;f__Ruminococcaceae;g__HUN007;s__
TP030MC_bin.59	d__Bacteria;p__Firmicutes_A;c__Clostridia;o__Lachnospirales;f__Lachnospiraceae;g__;s__
TP030MC_bin.63	d__Bacteria;p__Bacteroidota;c__Bacteroidia;o__Bacteroidales;f__Bacteroidaceae;g__UBA4372;s__UBA4372 sp004559785
TP031MC_bin.105	d__Bacteria;p__Bacteroidota;c__Bacteroidia;o__Bacteroidales;f__Bacteroidaceae;g__F0040;s__
TP031MC_bin.2	d__Bacteria;p__Firmicutes_A;c__Clostridia;o__Lachnospirales;f__Lachnospiraceae;g__Agathobacter;s__
TP031MC_bin.26	d__Bacteria;p__Firmicutes_A;c__Clostridia;o__Lachnospirales;f__Lachnospiraceae;g__Lachnospira;s__
TP031MC_bin.46	d__Bacteria;p__Firmicutes_A;c__Clostridia;o__Lachnospirales;f__Lachnospiraceae;g__CAG-194;s__
TP031MC_bin.51	d__Bacteria;p__Firmicutes_A;c__Clostridia;o__Lachnospirales;f__Lachnospiraceae;g__Acetatifactor;s__
TP031MC_bin.68	d__Bacteria;p__Firmicutes_A;c__Clostridia;o__Lachnospirales;f__Lachnospiraceae;g__;s__
TP033_bin.130	d__Bacteria;p__Firmicutes_A;c__Clostridia;o__Oscillospirales;f__Ruminococcaceae;g__CAG-115;s__
TP033_bin.32	d__Bacteria;p__Firmicutes_A;c__Clostridia;o__Oscillospirales;f__Ruminococcaceae;g__CAG-353;s__
TP033_bin.74	d__Bacteria;p__Bacteroidota;c__Bacteroidia;o__Bacteroidales;f__W3P20-009;g__W3P20-009;s__
TP033_bin.8	d__Bacteria;p__Bacteroidota;c__Bacteroidia;o__Bacteroidales;f__UBA932;g__RC9;s__
TP033_bin.92	d__Bacteria;p__Spirochaetota;c__Spirochaetia;o__Treponematales;f__Treponemataceae;g__Treponema_D;s__
TP034MC_bin.112	d__Bacteria;p__Firmicutes_A;c__Clostridia;o__Lachnospirales;f__Lachnospiraceae;g__Agathobacter;s__
TP034MC_bin.136	d__Bacteria;p__Bacteroidota;c__Bacteroidia;o__Bacteroidales;f__Bacteroidaceae;g__Prevotellamassilia;s__Prevotellamassilia sp004552195
TP034MC_bin.147	d__Bacteria;p__Bacteroidota;c__Bacteroidia;o__Bacteroidales;f__Bacteroidaceae;g__Prevotella;s__
TP034MC_bin.156	d__Bacteria;p__Bacteroidota;c__Bacteroidia;o__Bacteroidales;f__Bacteroidaceae;g__UBA4372;s__
TP034MC_bin.157	d__Bacteria;p__Proteobacteria;c__Alphaproteobacteria;o__RF32;f__CAG-239;g__RUG410;s__
TP034MC_bin.30	d__Bacteria;p__Bacteroidota;c__Bacteroidia;o__Bacteroidales;f__Bacteroidaceae;g__Prevotella;s__
TP034MC_bin.40	d__Bacteria;p__Bacteroidota;c__Bacteroidia;o__Bacteroidales;f__Bacteroidaceae;g__Prevotellamassilia;s__
TP034MC_bin.53	d__Bacteria;p__Bacteroidota;c__Bacteroidia;o__Bacteroidales;f__Muribaculaceae;g__;s__
TP034MC_bin.55	d__Bacteria;p__Cyanobacteria;c__Vampirovibrionia;o__Gastranaerophilales;f__Gastranaerophilaceae;g__Gastranaerophilus;s__
TP034MC_bin.57	d__Bacteria;p__Firmicutes_A;c__Clostridia;o__Oscillospirales;f__Ruminococcaceae;g__CAG-115;s__
TP034MC_bin.67	d__Bacteria;p__Firmicutes_A;c__Clostridia;o__Lachnospirales;f__Lachnospiraceae;g__Agathobacter;s__
TP034MC_bin.76	d__Bacteria;p__Firmicutes_A;c__Clostridia;o__Lachnospirales;f__Lachnospiraceae;g__;s__
TP034MC_bin.88	d__Bacteria;p__Firmicutes_A;c__Clostridia;o__Lachnospirales;f__Lachnospiraceae;g__Acetatifactor;s__
TP034MC_bin.90	d__Bacteria;p__Verrucomicrobiota;c__Kiritimatiellae;o__RFP12;f__UBA1067;g__UBA1067;s__
TP034MC_bin.98	d__Bacteria;p__Firmicutes_A;c__Clostridia;o__Lachnospirales;f__CAG-274;g__;s__
TP036_bin.108	d__Bacteria;p__Firmicutes_A;c__Clostridia;o__Lachnospirales;f__Lachnospiraceae;g__CAG-127;s__
TP036_bin.120	d__Bacteria;p__Firmicutes_A;c__Clostridia;o__Oscillospirales;f__Ruminococcaceae;g__UMGS363;s__
TP036_bin.122	d__Bacteria;p__Firmicutes_A;c__Clostridia;o__Lachnospirales;f__Lachnospiraceae;g__Acetatifactor;s__
TP036_bin.44	d__Bacteria;p__Firmicutes_A;c__Clostridia;o__Lachnospirales;f__Lachnospiraceae;g__CAG-882;s__
TP036_bin.66	d__Bacteria;p__Firmicutes_A;c__Clostridia;o__Lachnospirales;f__Lachnospiraceae;g__;s__
TP036_bin.7	d__Bacteria;p__Bacteroidota;c__Bacteroidia;o__Bacteroidales;f__Bacteroidaceae;g__Prevotellamassilia;s__
TP036_bin.79	d__Bacteria;p__Firmicutes_A;c__Clostridia;o__Lachnospirales;f__Lachnospiraceae;g__UBA7050;s__
TP036_bin.80	d__Bacteria;p__Bacteroidota;c__Bacteroidia;o__Bacteroidales;f__Paludibacteraceae;g__W0P28-013;s__
TP036MC_bin.106	d__Bacteria;p__Bacteroidota;c__Bacteroidia;o__Bacteroidales;f__Paludibacteraceae;g__RF16;s__
TP036MC_bin.117	d__Bacteria;p__Firmicutes_A;c__Clostridia;o__Lachnospirales;f__Lachnospiraceae;g__CAG-127;s__
TP036MC_bin.15	d__Bacteria;p__Firmicutes_A;c__Clostridia;o__Oscillospirales;f__Oscillospiraceae;g__CAG-110;s__
TP036MC_bin.37	d__Bacteria;p__Cyanobacteria;c__Vampirovibrionia;o__Gastranaerophilales;f__Gastranaerophilaceae;g__Zag111;s__
TP036MC_bin.50	d__Bacteria;p__Bacteroidota;c__Bacteroidia;o__Bacteroidales;f__Bacteroidaceae;g__Prevotella;s__
TP036MC_bin.76	d__Bacteria;p__Firmicutes_A;c__Clostridia;o__Lachnospirales;f__Lachnospiraceae;g__;s__
TP037MC_bin.162	d__Bacteria;p__Proteobacteria;c__Alphaproteobacteria;o__RF32;f__CAG-239;g__;s__
TP037MC_bin.21	d__Bacteria;p__Firmicutes_A;c__Clostridia_A;o__Christensenellales;f__Borkfalkiaceae;g__UBA11940;s__

SGBs ID	Classification
TP037MC_bin.37	d__Bacteria;p__Proteobacteria;c__Alphaproteobacteria;o__Rs-D84;f__Rs-D84;g__Rs-D84;s__
TP037MC_bin.44	d__Bacteria;p__Firmicutes_A;c__Clostridia;o__Oscillospirales;f__Ruminococcaceae;g__HUN007;s__
TP037MC_bin.57	d__Bacteria;p__Firmicutes_A;c__Clostridia;o__Oscillospirales;f__Oscillospiraceae;g__CAG-110;s__
TP040MC_bin.111	d__Bacteria;p__Firmicutes_A;c__Clostridia_A;o__Christensenellales;f__Borkfalkiaceae;g__;s__
TP040MC_bin.126	d__Bacteria;p__Actinobacteriota;c__Actinomycetia;o__Actinomycetales;f__Brevibacteriaceae;g__Brevibacterium;s__Brevibacterium aurantiacum
TP040MC_bin.137	d__Bacteria;p__Bacteroidota;c__Bacteroidia;o__Bacteroidales;f__UBA932;g__RC9;s__
TP040MC_bin.156	d__Bacteria;p__Firmicutes_A;c__Clostridia_A;o__Christensenellales;f__Borkfalkiaceae;g__UBA11940;s__
TP040MC_bin.19	d__Bacteria;p__Bacteroidota;c__Bacteroidia;o__Bacteroidales;f__Bacteroidaceae;g__Prevotella;s__
TP040MC_bin.35	d__Bacteria;p__Actinobacteriota;c__Actinomycetia;o__Actinomycetales;f__Dermatophilaceae;g__Janibacter;s__
TP040MC_bin.40	d__Bacteria;p__Firmicutes_A;c__Clostridia;o__Lachnospirales;f__Lachnospiraceae;g__;s__
TP040MC_bin.6	d__Bacteria;p__Proteobacteria;c__Alphaproteobacteria;o__Rhodobacterales;f__Rhodobacteraceae;g__Paracoccus;s__Paracoccus alcaliphilus
TP040MC_bin.79	d__Bacteria;p__Bacteroidota;c__Bacteroidia;o__Bacteroidales;f__UBA932;g__RC9;s__
TP040MC_bin.9	d__Bacteria;p__Verrucomicrobiota;c__Verrucomicrobiae;o__Opitutales;f__UBA953;g__W0P29-029;s__W0P29-029 sp004558035
TP040MC_bin.98	d__Bacteria;p__Firmicutes_A;c__Clostridia_A;o__Christensenellales;f__Borkfalkiaceae;g__;s__
TP041_bin.136	d__Bacteria;p__Firmicutes_A;c__Clostridia;o__Oscillospirales;f__Oscillospiraceae;g__CAG-110;s__
TP041_bin.152	d__Bacteria;p__Spirochaetota;c__Spirochaetia;o__Treponematales;f__Treponemataceae;g__Treponema_D;s__
TP041_bin.163	d__Bacteria;p__Verrucomicrobiota;c__Kiritimatiellae;o__RFP12;f__W1P29-020;g__W1P29-020;s__
TP041_bin.31	d__Bacteria;p__Firmicutes_A;c__Clostridia;o__Lachnospirales;f__Lachnospiraceae;g__Ruminococcus_A;s__
TP041_bin.33	d__Bacteria;p__Bacteroidota;c__Bacteroidia;o__Bacteroidales;f__Bacteroidaceae;g__Prevotella;s__
TP041_bin.34	d__Bacteria;p__Verrucomicrobiota;c__Kiritimatiellae;o__RFP12;f__UBA1067;g__UBA1067;s__
TP041MC_bin.110	d__Bacteria;p__Firmicutes_A;c__Clostridia;o__Oscillospirales;f__Oscillospiraceae;g__CAG-110;s__
TP041MC_bin.115	d__Bacteria;p__Bacteroidota;c__Bacteroidia;o__Bacteroidales;f__UBA932;g__RC9;s__
TP041MC_bin.117	d__Bacteria;p__Actinobacteriota;c__Actinomycetia;o__Actinomycetales;f__Bifidobacteriaceae;g__Pseudoscardovia;s__
TP041MC_bin.118	d__Bacteria;p__Firmicutes_A;c__Clostridia;o__Oscillospirales;f__Oscillospiraceae;g__CAG-110;s__
TP041MC_bin.125	d__Bacteria;p__Proteobacteria;c__Alphaproteobacteria;o__Rs-D84;f__Rs-D84;g__Rs-D84;s__
TP041MC_bin.31	d__Bacteria;p__Bacteroidota;c__Bacteroidia;o__Bacteroidales;f__Muribaculaceae;g__CAG-873;s__
TP042_bin.125	d__Bacteria;p__Firmicutes_A;c__Clostridia;o__Oscillospirales;f__Ruminococcaceae;g__;s__
TP042_bin.126	d__Bacteria;p__Eremiobacterota;c__UBP9;o__;f__;g__;s__
TP042_bin.127	d__Bacteria;p__Fibrobacterota;c__Fibrobacteria;o__Fibrobacterales;f__Fibrobacteraceae;g__Fibrobacter;s__Fibrobacter sp900142475
TP042_bin.29	d__Bacteria;p__Bacteroidota;c__Bacteroidia;o__Bacteroidales;f__P3;g__UBA3388;s__
TP042_bin.53	d__Bacteria;p__Firmicutes_A;c__Clostridia;o__Lachnospirales;f__Lachnospiraceae;g__UBA2882;s__
TP043_bin.104	d__Bacteria;p__Bacteroidota;c__Bacteroidia;o__Bacteroidales;f__UBA932;g__RC9;s__
TP043_bin.118	d__Bacteria;p__Firmicutes;c__Bacilli;o__RFN20;f__CAG-826;g__UBA4855;s__
TP043_bin.119	d__Bacteria;p__Bacteroidota;c__Bacteroidia;o__Bacteroidales;f__UBA932;g__RC9;s__
TP043_bin.127	d__Bacteria;p__Firmicutes;c__Bacilli;o__RFN20;f__CAG-826;g__UBA733;s__
TP043_bin.129	d__Bacteria;p__Bacteroidota;c__Bacteroidia;o__Bacteroidales;f__UBA932;g__RC9;s__RC9 sp004552965
TP043_bin.130	d__Bacteria;p__Proteobacteria;c__Alphaproteobacteria;o__Rickettsiales;f__UBA1997;g__;s__
TP043_bin.149	d__Bacteria;p__Myxococcota;c__Bradimonadia;o__Bradymonadales;f__UBA4248;g__UBA4248;s__
TP043_bin.15	d__Bacteria;p__Verrucomicrobiota;c__Kiritimatiellae;o__RFP12;f__UBA1067;g__RUG572;s__
TP043_bin.162	d__Bacteria;p__Firmicutes_A;c__Clostridia;o__Oscillospirales;f__Oscillospiraceae;g__CAG-110;s__
TP043_bin.169	d__Bacteria;p__Bacteroidota;c__Bacteroidia;o__Bacteroidales;f__UBA3663;g__UBA3663;s__
TP043_bin.174	d__Bacteria;p__Firmicutes_A;c__Clostridia;o__Oscillospirales;f__Oscillospiraceae;g__F23-B02;s__F23-B02 sp000431075
TP043_bin.177	d__Bacteria;p__Firmicutes_A;c__Clostridia_A;o__Christensenellales;f__UBA3700;g__UBA3700;s__
TP043_bin.179	d__Bacteria;p__Bacteroidota;c__Bacteroidia;o__Bacteroidales;f__Bacteroidaceae;g__Prevotellamassilia;s__Prevotellamassilia sp004557185
TP043_bin.18	d__Bacteria;p__Bacteroidota;c__Bacteroidia;o__Bacteroidales;f__UBA932;g__RC9;s__
TP043_bin.40	d__Bacteria;p__Bacteroidota;c__Bacteroidia;o__Bacteroidales;f__UBA932;g__RC9;s__
TP043_bin.49	d__Bacteria;p__Firmicutes;c__Bacilli;o__RFN20;f__CAG-449;g__UBA3210;s__
TP043_bin.51	d__Bacteria;p__Bacteroidota;c__Bacteroidia;o__Bacteroidales;f__UBA932;g__RC9;s__
TP043_bin.52	d__Bacteria;p__Bacteroidota;c__Bacteroidia;o__Bacteroidales;f__Bacteroidaceae;g__UBA6382;s__
TP043_bin.81	d__Bacteria;p__Spirochaetota;c__Spirochaetia;o__Treponematales;f__Treponemataceae;g__Treponema_D;s__
TP043_bin.93	d__Bacteria;p__Firmicutes_A;c__Clostridia;o__Oscillospirales;f__CAG-272;g__;s__
TP043_bin.97	d__Bacteria;p__Bacteroidota;c__Bacteroidia;o__Bacteroidales;f__Paludibacteraceae;g__RF16;s__
TP043_bin.98	d__Bacteria;p__Firmicutes_A;c__Clostridia;o__Oscillospirales;f__CAG-272;g__UBA1740;s__
TP043MC_bin.109	d__Bacteria;p__Bacteroidota;c__Bacteroidia;o__Bacteroidales;f__Bacteroidaceae;g__Prevotellamassilia;s__Prevotellamassilia sp004557035
TP043MC_bin.127	d__Bacteria;p__Firmicutes;c__Bacilli;o__RFN20;f__CAG-826;g__UBA733;s__
TP043MC_bin.14	d__Bacteria;p__Proteobacteria;c__Gammaproteobacteria;o__Enterobacterales;f__Succinivibrionaceae;g__Succinivibrio;s__
TP043MC_bin.142	d__Bacteria;p__Firmicutes;c__Bacilli;o__Acholeplasmatales;f__Anaeroplasmataceae;g__;s__
TP043MC_bin.147	d__Bacteria;p__Firmicutes_A;c__Clostridia;o__Oscillospirales;f__Ruminococcaceae;g__Gemmiger;s__Gemmiger variabilis_A
TP043MC_bin.22	d__Bacteria;p__Proteobacteria;c__Alphaproteobacteria;o__RF32;f__CAG-239;g__RUG410;s__
TP043MC_bin.32	d__Bacteria;p__Proteobacteria;c__Gammaproteobacteria;o__Enterobacterales;f__Succinivibrionaceae;g__Succinivibrio;s__
TP043MC_bin.59	d__Bacteria;p__Bacteroidota;c__Bacteroidia;o__Bacteroidales;f__Bacteroidaceae;g__Prevotellamassilia;s__
TP044MC_bin.16	d__Bacteria;p__Firmicutes_A;c__Clostridia;o__Oscillospirales;f__CAG-272;g__UBA1740;s__
TP044MC_bin.5	d__Bacteria;p__Bacteroidota;c__Bacteroidia;o__Bacteroidales;f__Bacteroidaceae;g__Prevotellamassilia;s__
TP044MC_bin.58	d__Bacteria;p__Bacteroidota;c__Bacteroidia;o__Bacteroidales;f__Bacteroidaceae;g__F0040;s__F0040 sp004555055
TP044MC_bin.62	d__Bacteria;p__Firmicutes_A;c__Clostridia;o__Lachnospirales;f__Lachnospiraceae;g__NK4A136;s__
TP044MC_bin.94	d__Bacteria;p__Bacteroidota;c__Bacteroidia;o__Bacteroidales;f__Bacteroidaceae;g__Prevotella;s__
TP044MC_bin.96	d__Bacteria;p__Bacteroidota;c__Bacteroidia;o__Bacteroidales;f__Muribaculaceae;g__CAG-279;s__
TP045_bin.1	d__Bacteria;p__Firmicutes_A;c__Clostridia_A;o__Christensenellales;f__UBA3700;g__UBA3700;s__
TP045_bin.106	d__Bacteria;p__Proteobacteria;c__Alphaproteobacteria;o__Rs-D84;f__Rs-D84;g__Rs-D84;s__
TP045_bin.113	d__Bacteria;p__Spirochaetota;c__Spirochaetia;o__Treponematales;f__Treponemataceae;g__Treponema_D;s__
TP045_bin.120	d__Bacteria;p__Verrucomicrobiota;c__Kiritimatiellae;o__RFP12;f__UBA1067;g__UBA1067;s__
TP045_bin.123	d__Bacteria;p__Firmicutes_A;c__Clostridia;o__Oscillospirales;f__Ruminococcaceae;g__Ruminococcus_D;s__
TP045_bin.129	d__Bacteria;p__Firmicutes_A;c__Clostridia_A;o__Christensenellales;f__Borkfalkiaceae;g__QALS01;s__
TP045_bin.14	d__Bacteria;p__Firmicutes_A;c__Clostridia;o__Oscillospirales;f__Ruminococcaceae;g__UBA1375;s__
TP045_bin.16	d__Bacteria;p__Firmicutes_A;c__Clostridia_A;o__Christensenellales;f__CAG-917;g__CAG-475;s__
TP045_bin.23	d__Bacteria;p__Firmicutes_A;c__Clostridia;o__Oscillospirales;f__CAG-272;g__UMGS1002;s__
TP045_bin.32	d__Bacteria;p__Firmicutes_A;c__Clostridia;o__Oscillospirales;f__CAG-272;g__UBA1740;s__
TP045_bin.44	d__Bacteria;p__Firmicutes_A;c__Clostridia_A;o__Christensenellales;f__CAG-74;g__SFMI01;s__
TP045_bin.48	d__Bacteria;p__Firmicutes_A;c__Clostridia_A;o__Christensenellales;f__Borkfalkiaceae;g__;s__
TP045_bin.86	d__Bacteria;p__Verrucomicrobiota;c__Kiritimatiellae;o__RFP12;f__UBA1067;g__UBA1067;s__
TP045_bin.91	d__Bacteria;p__Bacteroidota;c__Bacteroidia;o__Bacteroidales;f__Bacteroidaceae;g__Prevotellamassilia;s__
TP045_bin.93	d__Bacteria;p__Proteobacteria;p__Proteobacteria;c__Alphaproteobacteria;o__RF32;f__CAG-977;g__;s__
TP047MC_bin.127	d__Bacteria;p__Bacteroidota;c__Bacteroidia;o__Bacteroidales;f__Muribaculaceae;g__W0P31-042;s__W0P31-042 sp004557195
TP047MC_bin.136	d__Bacteria;p__Proteobacteria;c__Alphaproteobacteria;o__RF32;f__CAG-239;g__CAG-267;s__
TP047MC_bin.22	d__Bacteria;p__Firmicutes_A;c__Clostridia;o__Oscillospirales;f__Ruminococcaceae;g__CAG-115;s__
TP047MC_bin.79	d__Bacteria;p__Bacteroidota;c__Bacteroidia;o__Bacteroidales;f__UBA932;g__RC9;s__
TP047MC_bin.9	d__Bacteria;p__Firmicutes_A;c__Clostridia;o__Oscillospirales;f__Oscillospiraceae;g__CAG-110;s__
TP049MC_bin.106	d__Bacteria;p__Verrucomicrobiota;c__Verrucomicrobiae;o__Opitutales;f__UBA953;g__W0P29-029;s__
TP049MC_bin.114	d__Bacteria;p__Proteobacteria;c__Alphaproteobacteria;o__RF32;f__CAG-239;g__UBA1254;s__

SGBs ID	Classification
TP049MC_bin.125	d__Bacteria;p__Cyanobacteria;c__Vampirovibrionia;o__Gastranaerophilales;f__Gastranaerophilaceae;g__;s__
TP049MC_bin.131	d__Bacteria;p__Firmicutes_A;c__Clostridia;o__Lachnospirales;f__Lachnospiraceae;g__Acetatifactor;s__
TP049MC_bin.155	d__Bacteria;p__Verrucomicrobiota;c__Kiritimatiellae;o__RFP12;f__W1P29-020;g__W1P29-020;s__
TP049MC_bin.34	d__Bacteria;p__Bacteroidota;c__Bacteroidia;o__Bacteroidales;f__Bacteroidaceae;g__Phocaeicola;s__
TP049MC_bin.39	d__Bacteria;p__Firmicutes_A;c__Clostridia;o__Lachnospirales;f__Lachnospiraceae;g__;s__
TP049MC_bin.45	d__Bacteria;p__Proteobacteria;c__Alphaproteobacteria;o__RF32;f__CAG-239;g__CAG-495;s__
TP049MC_bin.7	d__Bacteria;p__Firmicutes_A;c__Clostridia;o__Oscillospirales;f__Ruminococcaceae;g__CAG-115;s__
TP049MC_bin.72	d__Bacteria;p__Desulfobacterota;c__Desulfovibrionia;o__Desulfovibrionales;f__Desulfovibrionaceae;g__Desulfovibrio;s__Desulfovibrio piger
TP049MC_bin.85	d__Bacteria;p__Firmicutes_A;c__Clostridia;o__Lachnospirales;f__Lachnospiraceae;g__SFDP01;s__
TP049MC_bin.88	d__Bacteria;p__Verrucomicrobiota;c__Kiritimatiellae;o__RFP12;f__UBA1067;g__UBA1067;s__
TP051_bin.108	d__Bacteria;p__Firmicutes_A;c__Clostridia;o__Lachnospirales;f__Lachnospiraceae;g__Acetatifactor;s__
TP051_bin.119	d__Bacteria;p__Firmicutes_A;c__Clostridia;o__Lachnospirales;f__Lachnospiraceae;g__Eubacterium_J;s__
TP051_bin.130	d__Bacteria;p__Firmicutes;c__Bacilli;o__RF39;f__CAG-611;g__UBA3789;s__
TP051_bin.134	d__Bacteria;p__Firmicutes_A;c__Clostridia;o__Lachnospirales;f__Lachnospiraceae;g__CAG-127;s__
TP051_bin.142	d__Bacteria;p__Firmicutes_A;c__Clostridia;o__Lachnospirales;f__Lachnospiraceae;g__Lachnospira;s__
TP051_bin.24	d__Bacteria;p__Firmicutes_A;c__Clostridia;o__Lachnospirales;f__Lachnospiraceae;g__Lachnospira;s__
TP051_bin.6	d__Bacteria;p__Firmicutes_A;c__Clostridia;o__Lachnospirales;f__Lachnospiraceae;g__;s__
TP051_bin.64	d__Bacteria;p__Bacteroidota;c__Bacteroidia;o__Bacteroidales;f__UBA932;g__RC9;s__
TP051_bin.70	d__Bacteria;p__Cyanobacteria;c__Vampirovibrionia;o__Gastranaerophilales;f__Gastranaerophilaceae;g__;s__
TP051_bin.95	d__Bacteria;p__Cyanobacteria;c__Vampirovibrionia;o__Gastranaerophilales;f__Gastranaerophilaceae;g__Zag111;s__
TP052MC_bin.118	d__Bacteria;p__Firmicutes_A;c__Clostridia;o__Lachnospirales;f__Lachnospiraceae;g__1XD8-76;s__
TP052MC_bin.128	d__Bacteria;p__Firmicutes_A;c__Clostridia;o__Lachnospirales;f__Lachnospiraceae;g__UBA1729;s__
TP052MC_bin.26	d__Bacteria;p__Spirochaetota;c__Spirochaetia;o__Treponematales;f__Treponemataceae;g__Treponema_D;s__
TP052MC_bin.32	d__Bacteria;p__Bacteroidota;c__Bacteroidia;o__Bacteroidales;f__Muribaculaceae;g__SFTJ01;s__
TP052MC_bin.34	d__Bacteria;p__Firmicutes_A;c__Clostridia;o__Lachnospirales;f__Lachnospiraceae;g__Butyrivibrio_A;s__
TP052MC_bin.39	d__Bacteria;p__Firmicutes_A;c__Clostridia;o__Lachnospirales;f__Lachnospiraceae;g__Eubacterium_J;s__
TP052MC_bin.45	d__Bacteria;p__Firmicutes_A;c__Clostridia;o__Lachnospirales;f__Lachnospiraceae;g__Bacteroides_F;s__
TP052MC_bin.56	d__Bacteria;p__Firmicutes_A;c__Clostridia;o__Lachnospirales;f__Lachnospiraceae;g__V9D3004;s__
TP052MC_bin.57	d__Bacteria;p__Bacteroidota;c__Bacteroidia;o__Bacteroidales;f__Muribaculaceae;g__CAG-873;s__
TP052MC_bin.61	d__Bacteria;p__Firmicutes_A;c__Clostridia;o__Oscillospirales;f__Ruminococcaceae;g__CAG-353;s__
TP052MC_bin.64	d__Bacteria;p__Firmicutes_A;c__Clostridia;o__Lachnospirales;f__Lachnospiraceae;g__Butyrivibrio_A;s__
TP052MC_bin.82	d__Bacteria;p__Firmicutes_A;c__Clostridia;o__Oscillospirales;f__Ruminococcaceae;g__CAG-115;s__
TP052MC_bin.93	d__Bacteria;p__Firmicutes_A;c__Clostridia;o__Oscillospirales;f__Ruminococcaceae;g__CAG-353;s__
TP053MC_bin.10	d__Bacteria;p__Proteobacteria;c__Alphaproteobacteria;o__RF32;f__CAG-239;g__CAG-267;s__
TP053MC_bin.119	d__Bacteria;p__Firmicutes_A;c__Clostridia;o__Oscillospirales;f__Acutalibacteraceae;g__UBA737;s__
TP053MC_bin.136	d__Bacteria;p__Bacteroidota;c__Bacteroidia;o__Bacteroidales;f__Paludibacteraceae;g__RF16;s__
TP053MC_bin.17	d__Bacteria;p__Firmicutes_A;c__Clostridia;o__Oscillospirales;f__Oscillospiraceae;g__CAG-110;s__
TP053MC_bin.2	d__Bacteria;p__Firmicutes_C;c__Negativicutes;o__Selenomonadales;f__Selenomonadaceae;g__Anaerovibrio;s__
TP053MC_bin.38	d__Bacteria;p__Firmicutes_A;c__Clostridia;o__Lachnospirales;f__Lachnospiraceae;g__CAG-882;s__
TP053MC_bin.7	d__Bacteria;p__Firmicutes_A;c__Clostridia;o__Oscillospirales;f__Oscillospiraceae;g__CAG-110;s__
TP053MC_bin.93	d__Bacteria;p__Cyanobacteria;c__Vampirovibrionia;o__Gastranaerophilales;f__Gastranaerophilaceae;g__Zag1;s__
TP053MC_bin.97	d__Bacteria;p__Bacteroidota;c__Bacteroidia;o__Bacteroidales;f__Bacteroidaceae;g__Prevotellamassilia;s__
TP056_bin.100	d__Bacteria;p__Firmicutes_C;c__Negativicutes;o__Acidaminococcales;f__Acidaminococcaceae;g__Phascolarctobacterium_A;s__
TP056_bin.102	d__Bacteria;p__Bacteroidota;c__Bacteroidia;o__Bacteroidales;f__UBA932;g__RC9;s__RC9 sp004555145
TP056_bin.109	d__Bacteria;p__Bacteroidota;c__Bacteroidia;o__Bacteroidales;f__UBA932;g__RC9;s__
TP056_bin.37	d__Bacteria;p__Firmicutes_A;c__Clostridia;o__Oscillospirales;f__Ruminococcaceae;g__Ruminococcus;s__
TP056_bin.5	d__Bacteria;p__Bacteroidota;c__Bacteroidia;o__Bacteroidales;f__Bacteroidaceae;g__Prevotella;s__
TP056_bin.56	d__Bacteria;p__Bacteroidota;c__Bacteroidia;o__Bacteroidales;f__Bacteroidaceae;g__Phocaeicola;s__
TP056_bin.70	d__Bacteria;p__Firmicutes_C;c__Negativicutes;o__Acidaminococcales;f__Acidaminococcaceae;g__Succiniclasticum;s__Succiniclasticum sp900314855
TP058_bin.103	d__Bacteria;p__Verrucomicrobiota;c__Kiritimatiellae;o__RFP12;f__W1P29-020;g__W1P29-020;s__
TP058_bin.105	d__Bacteria;p__Proteobacteria;c__Alphaproteobacteria;o__RF32;f__CAG-239;g__RUG410;s__
TP058_bin.17	d__Bacteria;p__Firmicutes_A;c__Clostridia;o__Oscillospirales;f__Oscillospiraceae;g__Oscillibacter;s__
TP058_bin.18	d__Bacteria;p__Verrucomicrobiota;c__Kiritimatiellae;o__RFP12;f__UBA3636;g__;s__
TP058_bin.27	d__Bacteria;p__Bacteroidota;c__Bacteroidia;o__Bacteroidales;f__UBA932;g__RC9;s__
TP058_bin.47	d__Bacteria;p__Firmicutes_A;c__Clostridia;o__Oscillospirales;f__Ruminococcaceae;g__Faecalibacterium;s__
TP058_bin.50	d__Bacteria;p__Bacteroidota;c__Bacteroidia;o__Bacteroidales;f__Bacteroidaceae;g__Prevotella;s__
TP058_bin.7	d__Bacteria;p__Firmicutes_A;c__Clostridia;o__Lachnospirales;f__Lachnospiraceae;g__Agathobacter;s__
TP058_bin.70	d__Bacteria;p__Firmicutes_A;c__Clostridia;o__Oscillospirales;f__Oscillospiraceae;g__CAG-110;s__
TP058_bin.84	d__Bacteria;p__Firmicutes_A;c__Clostridia;o__Oscillospirales;f__Ruminococcaceae;g__CAG-115;s__
TP058_bin.85	d__Bacteria;p__Firmicutes;c__Bacilli;o__RF39;f__CAG-822;g__UBA5026;s__
TP058_bin.96	d__Bacteria;p__Spirochaetota;c__Spirochaetia;o__Sphaerochaetales;f__Sphaerochaetaceae;g__UBA5920;s__
TP059MC_bin.112	d__Bacteria;p__Firmicutes_A;c__Clostridia;o__Lachnospirales;f__Lachnospiraceae;g__;s__
TP059MC_bin.114	d__Bacteria;p__Bacteroidota;c__Bacteroidia;o__Bacteroidales;f__Bacteroidaceae;g__Prevotellamassilia;s__
TP059MC_bin.116	d__Bacteria;p__Firmicutes;c__Bacilli;o__Erysipelotrichales;f__Erysipelotrichaceae;g__UBA636;s__
TP059MC_bin.121	d__Bacteria;p__Firmicutes_A;c__Clostridia_A;o__Christensenellales;f__Borkfalkiaceae;g__;s__
TP059MC_bin.127	d__Bacteria;p__Firmicutes_C;c__Negativicutes;o__Selenomonadales;f__Selenomonadaceae;g__Anaerovibrio;s__
TP059MC_bin.151	d__Bacteria;p__Firmicutes_A;c__Clostridia;o__Lachnospirales;f__Lachnospiraceae;g__Lachnospira;s__
TP059MC_bin.181	d__Bacteria;p__Verrucomicrobiota;c__Kiritimatiellae;o__RFP12;f__UBA1067;g__UBA1067;s__
TP059MC_bin.25	d__Bacteria;p__Bacteroidota;c__Bacteroidia;o__Bacteroidales;f__Paludibacteraceae;g__W0P28-013;s__
TP059MC_bin.84	d__Bacteria;p__Bacteroidota;c__Bacteroidia;o__Bacteroidales;f__Bacteroidaceae;g__Prevotella;s__
TP059MC_bin.87	d__Bacteria;p__Firmicutes_A;c__Clostridia;o__Lachnospirales;f__Lachnospiraceae;g__Acetatifactor;s__

Supplementary Table 4. The enrichment analysis of 1048 SGBs in three groups

SGBs	Compared groups	p values	Fdr	Enriched Group
TP052MC_bin.118	TPs-VS-EPs	7.37E-37	1.89E-35	TPs
TP059MC_bin.112	TPs-VS-EPs	1.18E-10	2.90E-10	TPs
ERR1135277_bin.11	TPs-VS-EPs	0.015469025	0.019894291	EPs
TP017MC_bin.126	TPs-VS-EPs	2.38E-44	1.28E-42	TPs
ERR1135399_bin.16	TPs-VS-EPs	1.33E-54	2.73E-52	TPs
ERR1135218_bin.14	TPs-VS-EPs	1.08E-19	4.93E-19	TPs
TP052MC_bin.32	TPs-VS-EPs	7.66E-34	1.29E-32	TPs
TP015MC_bin.116	TPs-VS-EPs	7.50E-31	9.85E-30	TPs
TP020_bin.26	TPs-VS-EPs	0.000127405	0.000202466	EPs
ERR1135195_bin.61	TPs-VS-EPs	0.006156143	0.008357678	EPs
ERR1135216_bin.4	TPs-VS-EPs	0.006680502	0.009009888	EPs
ERR1135371_bin.2	TPs-VS-EPs	2.65E-21	1.39E-20	TPs
TP007MC_bin.110	TPs-VS-EPs	4.59E-36	1.04E-34	TPs
ERR1135315_bin.55	TPs-VS-EPs	0.001203415	0.001764665	EPs
TP021MC_bin.71	TPs-VS-EPs	9.92E-11	2.47E-10	TPs
TP028_bin.79	TPs-VS-EPs	1.58E-28	1.63E-27	TPs
TP003MC_bin.114	TPs-VS-EPs	2.39E-10	5.76E-10	TPs
ERR1135325_bin.13	TPs-VS-EPs	0.005780209	0.007868146	EPs
ERR1135293_bin.50	TPs-VS-EPs	1.52E-21	8.28E-21	EPs
ERR1135398_bin.29	TPs-VS-EPs	0.038693354	0.048190386	EPs
ERR1135205_bin.17	TPs-VS-EPs	1.08E-16	3.93E-16	EPs
TP007MC_bin.132	TPs-VS-EPs	8.31E-38	2.18E-36	TPs
TP030MC_bin.63	TPs-VS-EPs	1.10E-17	4.37E-17	TPs
TP049MC_bin.34	TPs-VS-EPs	2.15E-49	2.45E-47	TPs
ERR1135409_bin.26	TPs-VS-EPs	1.43E-14	4.58E-14	EPs
TP021_bin.55	TPs-VS-EPs	0.005829168	0.007924267	TPs
TP008MC_bin.81	TPs-VS-EPs	4.39E-25	3.29E-24	TPs
TP014_bin.70	TPs-VS-EPs	5.28E-13	1.51E-12	TPs
ERR1135214_bin.8	TPs-VS-EPs	9.39E-07	1.72E-06	EPs
ERR1135308_bin.44	TPs-VS-EPs	1.45E-11	3.81E-11	EPs
ERR1135318_bin.39	TPs-VS-EPs	0.003536051	0.004920154	EPs
ERR1135274_bin.6	TPs-VS-EPs	1.84E-09	4.15E-09	EPs
ERR1135192_bin.7	TPs-VS-EPs	0.001858124	0.00269008	EPs
TP045_bin.93	TPs-VS-EPs	0.000186189	0.000292256	TPs
ERR1135455_bin.2	TPs-VS-EPs	9.14E-17	3.36E-16	TPs
TP012MC_bin.71	TPs-VS-EPs	1.96E-29	2.28E-28	TPs
ERR1135380_bin.23	TPs-VS-EPs	3.09E-09	6.89E-09	TPs
TP036_bin.66	TPs-VS-EPs	4.81E-24	3.35E-23	TPs
TP043_bin.179	TPs-VS-EPs	1.02E-06	1.86E-06	TPs
TP018MC_bin.143	TPs-VS-EPs	1.94E-21	1.04E-20	TPs
TP015MC_bin.37	TPs-VS-EPs	9.43E-20	4.31E-19	TPs
ERR1135426_bin.50	TPs-VS-EPs	0.004141764	0.00572144	EPs
TP008_bin.114	TPs-VS-EPs	0.000425407	0.000650809	TPs
TP029_bin.169	TPs-VS-EPs	8.36E-35	1.48E-33	TPs
TP009_bin.4	TPs-VS-EPs	3.05E-20	1.45E-19	TPs
ERR1135197_bin.71	TPs-VS-EPs	0.002855762	0.004009803	EPs
TP020MC_bin.23	TPs-VS-EPs	2.07E-41	8.14E-40	TPs
TP031MC_bin.2	TPs-VS-EPs	4.39E-25	3.29E-24	TPs
ERR1135424_bin.1	TPs-VS-EPs	0.000717569	0.001072169	TPs
ERR1135199_bin.61	TPs-VS-EPs	2.07E-19	9.14E-19	EPs
ERR1135187_bin.54	TPs-VS-EPs	1.39E-16	5.06E-16	TPs
ERR1135206_bin.63	TPs-VS-EPs	1.58E-06	2.86E-06	EPs
TP005_bin.59	TPs-VS-EPs	6.96E-28	6.93E-27	TPs
TP022_bin.99	TPs-VS-EPs	3.20E-16	1.14E-15	TPs
TP024MC_bin.131	TPs-VS-EPs	1.15E-36	2.88E-35	TPs
TP021_bin.140	TPs-VS-EPs	1.94E-05	3.26E-05	TPs
TP033_bin.92	TPs-VS-EPs	9.76E-22	5.47E-21	TPs
TP002_bin.87	TPs-VS-EPs	1.22E-27	1.17E-26	TPs
TP043_bin.49	TPs-VS-EPs	0.004625277	0.006346599	TPs
TP022_bin.117	TPs-VS-EPs	5.84E-35	1.11E-33	TPs
ERR1135357_bin.28	TPs-VS-EPs	1.08E-21	6.00E-21	TPs
TP021_bin.28	TPs-VS-EPs	1.58E-28	1.63E-27	TPs
ERR1135215_bin.23	TPs-VS-EPs	9.39E-07	1.72E-06	EPs
TP024MC_bin.61	TPs-VS-EPs	5.05E-07	9.40E-07	TPs
TP028_bin.145	TPs-VS-EPs	2.94E-21	1.54E-20	TPs
TP020_bin.103	TPs-VS-EPs	3.47E-07	6.61E-07	TPs
TP043_bin.97	TPs-VS-EPs	1.74E-13	5.23E-13	TPs
ERR1135447_bin.34	TPs-VS-EPs	0.015469025	0.019894291	EPs
TP001_bin.35	TPs-VS-EPs	2.28E-56	1.17E-53	TPs
TP025_bin.93	TPs-VS-EPs	3.06E-12	8.25E-12	TPs
TP034MC_bin.57	TPs-VS-EPs	2.65E-05	4.39E-05	TPs
TP043_bin.104	TPs-VS-EPs	0.013168665	0.017129292	TPs
TP045_bin.113	TPs-VS-EPs	2.13E-23	1.38E-22	TPs
ERR1135346_bin.81	TPs-VS-EPs	1.26E-12	3.53E-12	EPs
TP003_bin.127	TPs-VS-EPs	1.99E-17	7.83E-17	TPs
TP041_bin.33	TPs-VS-EPs	6.83E-17	2.53E-16	TPs
ERR1135190_bin.91	TPs-VS-EPs	0.007904527	0.010618794	EPs
TP031MC_bin.26	TPs-VS-EPs	0.002395041	0.003400162	TPs
ERR1135208_bin.18	TPs-VS-EPs	2.73E-09	6.11E-09	TPs
ERR1135341_bin.9	TPs-VS-EPs	1.03E-26	9.15E-26	EPs
ERR1135200_bin.85	TPs-VS-EPs	0.006533645	0.008858447	EPs
TP020_bin.23	TPs-VS-EPs	8.36E-35	1.48E-33	TPs
ERR1135245_bin.26	TPs-VS-EPs	9.66E-22	5.47E-21	EPs
ERR1135201_bin.86	TPs-VS-EPs	3.25E-08	6.75E-08	EPs
TP041MC_bin.125	TPs-VS-EPs	6.83E-17	2.53E-16	TPs
TP029MC_bin.9	TPs-VS-EPs	0.003649548	0.005068817	TPs
ERR1135187_bin.84	TPs-VS-EPs	0.012769055	0.016801388	EPs
ERR1135254_bin.35	TPs-VS-EPs	1.03E-05	1.79E-05	EPs
TP043MC_bin.14	TPs-VS-EPs	3.06E-12	8.25E-12	TPs
TP008MC_bin.8	TPs-VS-EPs	1.72E-11	4.51E-11	TPs
ERR1135216_bin.19	TPs-VS-EPs	2.96E-13	8.74E-13	EPs
ERR1135221_bin.24	TPs-VS-EPs	0.006680502	0.009009888	EPs
TP056_bin.109	TPs-VS-EPs	3.08E-24	2.19E-23	TPs

SGBs	Compared groups	p values	Fdr	Enriched Group
TP027MC_bin.96	TPs-VS-EPs	0.013168665	0.017129292	TPs
TP051_bin.130	TPs-VS-EPs	9.30E-08	1.84E-07	TPs
ERR1135219_bin.32	TPs-VS-EPs	2.05E-10	4.96E-10	EPs
TP004MC_bin.38	TPs-VS-EPs	0.000249387	0.000389074	EPs
ERR1135225_bin.24	TPs-VS-EPs	0.015469025	0.019894291	EPs
TP058_bin.27	TPs-VS-EPs	1.86E-25	1.53E-24	TPs
ERR1135202_bin.56	TPs-VS-EPs	8.70E-06	1.51E-05	TPs
TP033_bin.74	TPs-VS-EPs	2.05E-18	8.59E-18	TPs
TP037MC_bin.57	TPs-VS-EPs	2.10E-25	1.71E-24	TPs
TP044MC_bin.5	TPs-VS-EPs	2.31E-43	1.13E-41	TPs
ERR1135280_bin.46	TPs-VS-EPs	0.000393209	0.00060882	TPs
TP043_bin.127	TPs-VS-EPs	4.11E-25	3.19E-24	TPs
ERR1135328_bin.24	TPs-VS-EPs	3.21E-25	2.53E-24	EPs
ERR1135197_bin.53	TPs-VS-EPs	8.50E-21	4.23E-20	EPs
ERR1135252_bin.62	TPs-VS-EPs	0.000498457	0.000753566	EPs
TP021_bin.119	TPs-VS-EPs	0.013168665	0.017129292	TPs
ERR1135316_bin.81	TPs-VS-EPs	8.73E-24	5.85E-23	EPs
TP034MC_bin.136	TPs-VS-EPs	4.65E-30	5.67E-29	TPs
ERR1135200_bin.44	TPs-VS-EPs	9.62E-30	1.13E-28	EPs
ERR1135276_bin.96	TPs-VS-EPs	0.00191426	0.002763545	EPs
TP002_bin.105	TPs-VS-EPs	1.75E-11	4.55E-11	TPs
TP025_bin.142	TPs-VS-EPs	1.59E-17	6.27E-17	TPs
TP045_bin.106	TPs-VS-EPs	9.03E-14	2.73E-13	TPs
TP009_bin.25	TPs-VS-EPs	6.47E-21	3.27E-20	TPs
ERR1135362_bin.43	TPs-VS-EPs	1.43E-20	6.98E-20	EPs
TP044MC_bin.94	TPs-VS-EPs	9.67E-36	2.01E-34	TPs
ERR1135230_bin.14	TPs-VS-EPs	0.000248401	0.000388126	EPs
ERR1135217_bin.57	TPs-VS-EPs	6.84E-17	2.53E-16	EPs
ERR1135264_bin.32	TPs-VS-EPs	6.14E-13	1.75E-12	EPs
TP052MC_bin.56	TPs-VS-EPs	0.000425407	0.000650809	TPs
TP008MC_bin.72	TPs-VS-EPs	4.32E-39	1.30E-37	TPs
TP027MC_bin.112	TPs-VS-EPs	2.65E-12	7.27E-12	TPs
ERR1135241_bin.24	TPs-VS-EPs	0.010175304	0.01352748	EPs
TP043_bin.15	TPs-VS-EPs	4.61E-27	4.25E-26	TPs
TP043_bin.177	TPs-VS-EPs	3.35E-16	1.19E-15	TPs
ERR1135266_bin.18	TPs-VS-EPs	0.009148869	0.012226324	EPs
ERR1135189_bin.44	TPs-VS-EPs	1.19E-05	2.04E-05	EPs
ERR1135465_bin.7	TPs-VS-EPs	0.018627634	0.023926472	EPs
ERR1135435_bin.8	TPs-VS-EPs	1.40E-09	3.18E-09	TPs
ERR1135242_bin.36	TPs-VS-EPs	2.12E-25	1.71E-24	EPs
ERR1135322_bin.22	TPs-VS-EPs	1.55E-28	1.63E-27	EPs
ERR1135317_bin.24	TPs-VS-EPs	0.004293875	0.005923583	EPs
TP053MC_bin.17	TPs-VS-EPs	2.13E-23	1.38E-22	TPs
TP058_bin.17	TPs-VS-EPs	5.83E-31	7.75E-30	TPs
TP043MC_bin.147	TPs-VS-EPs	2.09E-12	5.76E-12	EPs
ERR1135194_bin.5	TPs-VS-EPs	0.015469025	0.019894291	EPs
TP045_bin.91	TPs-VS-EPs	5.60E-33	8.32E-32	TPs
TP012_bin.56	TPs-VS-EPs	9.92E-11	2.47E-10	TPs
ERR1135381_bin.24	TPs-VS-EPs	8.36E-27	7.65E-26	TPs
TP041MC_bin.115	TPs-VS-EPs	9.36E-25	6.95E-24	TPs
TP012_bin.66	TPs-VS-EPs	0.000836889	0.001245009	TPs
ERR1135322_bin.29	TPs-VS-EPs	2.04E-13	6.09E-13	EPs
ERR1135197_bin.36	TPs-VS-EPs	0.000776287	0.001156532	EPs
TP012_bin.84	TPs-VS-EPs	1.73E-39	5.54E-38	TPs
ERR1135208_bin.20	TPs-VS-EPs	1.24E-19	5.59E-19	TPs
ERR1135245_bin.30	TPs-VS-EPs	0.011526174	0.015244294	EPs
TP024_bin.106	TPs-VS-EPs	8.72E-36	1.90E-34	TPs
ERR1135275_bin.23	TPs-VS-EPs	3.97E-05	6.48E-05	EPs
TP024_bin.130	TPs-VS-EPs	1.53E-20	7.43E-20	TPs
TP042_bin.53	TPs-VS-EPs	3.04E-25	2.41E-24	TPs
TP010_bin.119	TPs-VS-EPs	3.55E-08	7.33E-08	EPs
ERR1135188_bin.33	TPs-VS-EPs	7.09E-06	1.24E-05	TPs
ERR1135198_bin.54	TPs-VS-EPs	6.50E-15	2.14E-14	EPs
ERR1135292_bin.87	TPs-VS-EPs	7.15E-05	0.000114458	EPs
TP030MC_bin.109	TPs-VS-EPs	1.58E-28	1.63E-27	TPs
ERR1135360_bin.30	TPs-VS-EPs	4.73E-12	1.26E-11	TPs
TP052MC_bin.26	TPs-VS-EPs	1.16E-10	2.86E-10	TPs
TP012_bin.49	TPs-VS-EPs	0.002329506	0.003311711	TPs
TP052MC_bin.39	TPs-VS-EPs	3.25E-23	2.04E-22	TPs
TP009_bin.28	TPs-VS-EPs	2.35E-26	2.03E-25	TPs
ERR1135421_bin.27	TPs-VS-EPs	2.72E-06	4.84E-06	TPs
TP036MC_bin.117	TPs-VS-EPs	1.28E-14	4.11E-14	TPs
ERR1135213_bin.84	TPs-VS-EPs	0.002855762	0.004009803	EPs
ERR1135304_bin.16	TPs-VS-EPs	1.24E-22	7.59E-22	EPs
TP008_bin.11	TPs-VS-EPs	4.39E-25	3.29E-24	TPs
TP021_bin.103	TPs-VS-EPs	1.72E-09	3.90E-09	TPs
ERR1135182_bin.35	TPs-VS-EPs	0.001186678	0.001752658	TPs
TP003MC_bin.120	TPs-VS-EPs	2.72E-06	4.84E-06	TPs
TP058_bin.47	TPs-VS-EPs	1.17E-46	9.99E-45	TPs
TP018_bin.101	TPs-VS-EPs	1.18E-44	7.55E-43	TPs
TP037MC_bin.44	TPs-VS-EPs	4.20E-16	1.47E-15	TPs
TP030MC_bin.3	TPs-VS-EPs	9.30E-08	1.84E-07	TPs
TP028_bin.51	TPs-VS-EPs	5.60E-33	8.32E-32	TPs
ERR1135284_bin.14	TPs-VS-EPs	0.000622418	0.000932718	EPs
ERR1135408_bin.15	TPs-VS-EPs	4.72E-08	9.66E-08	TPs
TP007MC_bin.123	TPs-VS-EPs	3.91E-22	2.24E-21	TPs
TP058_bin.18	TPs-VS-EPs	0.003537711	0.004920154	TPs
TP043_bin.52	TPs-VS-EPs	1.73E-39	5.54E-38	TPs
ERR1135193_bin.58	TPs-VS-EPs	0.000202483	0.000317348	EPs
ERR1135220_bin.70	TPs-VS-EPs	3.38E-09	7.51E-09	EPs
TP018_bin.180	TPs-VS-EPs	9.28E-24	6.18E-23	TPs
ERR1135221_bin.29	TPs-VS-EPs	1.53E-05	2.59E-05	EPs
ERR1135178_bin.56	TPs-VS-EPs	1.07E-10	2.65E-10	EPs
TP025_bin.120	TPs-VS-EPs	2.65E-05	4.39E-05	TPs
TP014MC_bin.61	TPs-VS-EPs	3.53E-31	4.83E-30	TPs

SGBs	Compared groups	p values	Fdr	Enriched Group
ERR1135276_bin.43	TPs-VS-EPs	8.58E-11	2.18E-10	EPs
TP014_bin.24	TPs-VS-EPs	6.13E-23	3.83E-22	TPs
TP034MC_bin.88	TPs-VS-EPs	4.59E-13	1.34E-12	TPs
TP053MC_bin.10	TPs-VS-EPs	6.83E-17	2.53E-16	TPs
ERR1135396_bin.46	TPs-VS-EPs	4.06E-32	5.83E-31	TPs
ERR1135195_bin.54	TPs-VS-EPs	8.31E-05	0.000132478	EPs
TP008_bin.78	TPs-VS-EPs	5.56E-10	1.29E-09	TPs
TP041MC_bin.117	TPs-VS-EPs	2.54E-15	8.49E-15	TPs
TP021_bin.106	TPs-VS-EPs	1.28E-39	4.68E-38	TPs
ERR1135257_bin.52	TPs-VS-EPs	0.000928695	0.001377586	EPs
TP014_bin.83	TPs-VS-EPs	2.41E-45	1.65E-43	TPs
TP059MC_bin.114	TPs-VS-EPs	1.10E-17	4.37E-17	TPs
TP049MC_bin.39	TPs-VS-EPs	4.66E-11	1.20E-10	TPs
TP005MC_bin.38	TPs-VS-EPs	1.85E-45	1.35E-43	TPs
ERR1135327_bin.92	TPs-VS-EPs	7.41E-11	1.89E-10	TPs
TP058_bin.7	TPs-VS-EPs	9.92E-11	2.47E-10	TPs
ERR1135205_bin.60	TPs-VS-EPs	1.10E-20	5.44E-20	EPs
ERR1135278_bin.32	TPs-VS-EPs	1.60E-13	4.80E-13	EPs
ERR1135267_bin.37	TPs-VS-EPs	2.38E-08	4.98E-08	EPs
TP011_bin.135	TPs-VS-EPs	1.45E-22	8.65E-22	TPs
TP001_bin.109	TPs-VS-EPs	9.98E-36	2.01E-34	TPs
ERR1135378_bin.20	TPs-VS-EPs	2.46E-05	4.08E-05	EPs
ERR1135449_bin.10	TPs-VS-EPs	0.001039552	0.001537577	EPs
TP020_bin.122	TPs-VS-EPs	8.40E-24	5.67E-23	TPs
ERR1135192_bin.10	TPs-VS-EPs	0.01113967	0.014771232	TPs
ERR1135191_bin.22	TPs-VS-EPs	0.000890841	0.001323351	TPs
TP031MC_bin.68	TPs-VS-EPs	1.26E-21	6.97E-21	TPs
ERR1135194_bin.34	TPs-VS-EPs	9.03E-11	2.29E-10	TPs
ERR1135179_bin.22	TPs-VS-EPs	0.005407979	0.007381063	EPs
ERR1135192_bin.2	TPs-VS-EPs	0.006680502	0.009009888	EPs
ERR1135247_bin.31	TPs-VS-EPs	0.001857522	0.00269008	EPs
TP004MC_bin.13	TPs-VS-EPs	5.61E-46	4.42E-44	TPs
TP041_bin.31	TPs-VS-EPs	4.45E-13	1.30E-12	TPs
TP027MC_bin.89	TPs-VS-EPs	5.60E-33	8.32E-32	TPs
TP002_bin.75	TPs-VS-EPs	2.43E-17	9.44E-17	TPs
ERR1135250_bin.5	TPs-VS-EPs	7.94E-26	6.62E-25	EPs
TP034MC_bin.147	TPs-VS-EPs	1.70E-27	1.61E-26	TPs
ERR1135362_bin.58	TPs-VS-EPs	0.001995121	0.002864144	EPs
ERR1135400_bin.30	TPs-VS-EPs	3.32E-25	2.60E-24	TPs
TP025_bin.115	TPs-VS-EPs	1.25E-12	3.51E-12	TPs
ERR1135285_bin.14	TPs-VS-EPs	1.30E-06	2.36E-06	EPs
TP049MC_bin.155	TPs-VS-EPs	1.02E-09	2.35E-09	TPs
ERR1135200_bin.9	TPs-VS-EPs	7.33E-18	2.99E-17	TPs
TP047MC_bin.127	TPs-VS-EPs	3.08E-24	2.19E-23	TPs
ERR1135203_bin.44	TPs-VS-EPs	0.000498457	0.000753566	EPs
ERR1135199_bin.45	TPs-VS-EPs	6.61E-15	2.16E-14	EPs
ERR1135207_bin.59	TPs-VS-EPs	0.001628677	0.002378053	TPs
TP052MC_bin.128	TPs-VS-EPs	1.73E-20	8.34E-20	TPs
ERR1135272_bin.51	TPs-VS-EPs	6.66E-10	1.54E-09	EPs
ERR1135278_bin.75	TPs-VS-EPs	2.10E-14	6.59E-14	EPs
TP005_bin.147	TPs-VS-EPs	4.88E-31	6.58E-30	TPs
TP004MC_bin.86	TPs-VS-EPs	4.55E-32	6.39E-31	TPs
TP049MC_bin.88	TPs-VS-EPs	1.07E-10	2.65E-10	TPs
TP024_bin.91	TPs-VS-EPs	1.87E-12	5.17E-12	TPs
TP021_bin.45	TPs-VS-EPs	2.19E-08	4.61E-08	TPs
ERR1135223_bin.22	TPs-VS-EPs	0.001495893	0.002190414	EPs
ERR1135209_bin.28	TPs-VS-EPs	1.35E-09	3.09E-09	EPs
ERR1135385_bin.9	TPs-VS-EPs	0.000254119	0.000394654	EPs
ERR1135277_bin.29	TPs-VS-EPs	1.44E-14	4.60E-14	EPs
ERR1135348_bin.33	TPs-VS-EPs	0.004374693	0.006010805	EPs
TP008MC_bin.33	TPs-VS-EPs	1.54E-19	6.91E-19	TPs
ERR1135256_bin.60	TPs-VS-EPs	2.45E-12	6.75E-12	EPs
ERR1135289_bin.33	TPs-VS-EPs	0.019064642	0.024305047	EPs
TP049MC_bin.45	TPs-VS-EPs	2.27E-21	1.21E-20	TPs
TP053MC_bin.7	TPs-VS-EPs	6.18E-26	5.19E-25	TPs
ERR1135301_bin.23	TPs-VS-EPs	0.000398697	0.000614534	EPs
ERR1135364_bin.79	TPs-VS-EPs	0.003536051	0.004920154	EPs
ERR1135348_bin.59	TPs-VS-EPs	3.24E-07	6.18E-07	EPs
TP043MC_bin.32	TPs-VS-EPs	2.54E-15	8.49E-15	TPs
ERR1135256_bin.12	TPs-VS-EPs	1.82E-16	6.60E-16	EPs
ERR1135328_bin.36	TPs-VS-EPs	3.48E-22	2.04E-21	EPs
ERR1135264_bin.17	TPs-VS-EPs	4.67E-10	1.11E-09	TPs
ERR1135266_bin.21	TPs-VS-EPs	7.58E-10	1.75E-09	TPs
TP008MC_bin.88	TPs-VS-EPs	1.52E-14	4.82E-14	TPs
ERR1135191_bin.2	TPs-VS-EPs	0.015469025	0.019894291	EPs
TP022MC_bin.70	TPs-VS-EPs	5.56E-10	1.29E-09	TPs
TP043_bin.162	TPs-VS-EPs	3.91E-36	9.12E-35	TPs
TP022MC_bin.127	TPs-VS-EPs	1.70E-08	3.59E-08	TPs
TP053MC_bin.97	TPs-VS-EPs	6.47E-21	3.27E-20	TPs
ERR1135256_bin.53	TPs-VS-EPs	8.08E-24	5.48E-23	EPs
ERR1135286_bin.84	TPs-VS-EPs	8.01E-34	1.32E-32	TPs
TP029MC_bin.53	TPs-VS-EPs	0.013168665	0.017129292	TPs
ERR1135189_bin.63	TPs-VS-EPs	0.02107131	0.026796641	EPs
ERR1135429_bin.48	TPs-VS-EPs	9.30E-08	1.84E-07	TPs
TP022MC_bin.16	TPs-VS-EPs	2.74E-19	1.18E-18	TPs
TP045_bin.16	TPs-VS-EPs	2.37E-10	5.71E-10	TPs
ERR1135426_bin.24	TPs-VS-EPs	2.73E-22	1.61E-21	TPs
TP024_bin.115	TPs-VS-EPs	3.31E-33	5.30E-32	TPs
ERR1135391_bin.5	TPs-VS-EPs	0.003809103	0.005276123	TPs
TP042_bin.29	TPs-VS-EPs	2.74E-19	1.18E-18	TPs
TP015_bin.102	TPs-VS-EPs	7.21E-39	2.11E-37	TPs
ERR1135385_bin.68	TPs-VS-EPs	0.004694885	0.006433499	EPs
ERR1135456_bin.36	TPs-VS-EPs	0.010175304	0.01352748	EPs
ERR1135278_bin.69	TPs-VS-EPs	7.96E-18	3.24E-17	EPs
ERR1135288_bin.11	TPs-VS-EPs	0.007730213	0.010398253	EPs

SGBs	Compared groups	p values	Fdr	Enriched Group
ERR1135335_bin.32	TPs-VS-EPs	1.43E-14	4.59E-14	EPs
TP014_bin.7	TPs-VS-EPs	7.82E-16	2.74E-15	TPs
TP025_bin.34	TPs-VS-EPs	1.67E-55	5.71E-53	TPs
TP029MC_bin.90	TPs-VS-EPs	2.25E-07	4.36E-07	TPs
TP040MC_bin.19	TPs-VS-EPs	1.75E-18	7.37E-18	TPs
TP002_bin.70	TPs-VS-EPs	9.30E-08	1.84E-07	TPs
ERR1135279_bin.11	TPs-VS-EPs	5.71E-08	1.16E-07	EPs
ERR1135373_bin.49	TPs-VS-EPs	1.34E-06	2.42E-06	EPs
TP047MC_bin.79	TPs-VS-EPs	1.72E-20	8.31E-20	TPs
TP036MC_bin.37	TPs-VS-EPs	1.10E-14	3.57E-14	TPs
ERR1135288_bin.89	TPs-VS-EPs	0.024710133	0.031230439	EPs
ERR1135402_bin.1	TPs-VS-EPs	0.035686545	0.044662648	EPs
TP026_bin.115	TPs-VS-EPs	1.22E-19	5.54E-19	TPs
ERR1135377_bin.41	TPs-VS-EPs	7.47E-10	1.73E-09	TPs
ERR1135328_bin.73	TPs-VS-EPs	7.03E-17	2.59E-16	EPs
TP005MC_bin.82	TPs-VS-EPs	5.36E-29	6.04E-28	TPs
ERR1135187_bin.47	TPs-VS-EPs	0.009228069	0.012316108	TPs
TP025_bin.117	TPs-VS-EPs	4.70E-05	7.65E-05	TPs
TP034MC_bin.76	TPs-VS-EPs	3.77E-22	2.19E-21	TPs
ERR1135208_bin.40	TPs-VS-EPs	4.05E-08	8.31E-08	TPs
TP029_bin.122	TPs-VS-EPs	3.06E-12	8.25E-12	TPs
TP011_bin.84	TPs-VS-EPs	1.88E-21	1.01E-20	TPs
TP045_bin.44	TPs-VS-EPs	1.51E-08	3.23E-08	TPs
ERR1135196_bin.23	TPs-VS-EPs	0.005626487	0.007669081	EPs
ERR1135318_bin.98	TPs-VS-EPs	3.55E-30	4.49E-29	TPs
ERR1135196_bin.51	TPs-VS-EPs	6.15E-09	1.34E-08	EPs
ERR1135196_bin.68	TPs-VS-EPs	1.31E-15	4.45E-15	EPs
ERR1135273_bin.20	TPs-VS-EPs	3.40E-28	3.41E-27	EPs
TP058_bin.50	TPs-VS-EPs	3.08E-24	2.19E-23	TPs
TP036MC_bin.15	TPs-VS-EPs	1.81E-41	7.71E-40	TPs
TP025MC_bin.63	TPs-VS-EPs	2.36E-47	2.20E-45	TPs
TP027_bin.9	TPs-VS-EPs	4.92E-21	2.56E-20	TPs
TP029_bin.128	TPs-VS-EPs	8.83E-12	2.32E-11	TPs
TP008MC_bin.31	TPs-VS-EPs	1.52E-14	4.82E-14	TPs
TP018MC_bin.157	TPs-VS-EPs	3.80E-24	2.67E-23	TPs
TP051_bin.95	TPs-VS-EPs	8.58E-13	2.44E-12	TPs
ERR1135422_bin.61	TPs-VS-EPs	0.001203415	0.001764665	EPs
TP059MC_bin.87	TPs-VS-EPs	4.24E-20	1.97E-19	TPs
TP024MC_bin.118	TPs-VS-EPs	3.51E-22	2.04E-21	TPs
TP029_bin.15	TPs-VS-EPs	0.002329506	0.003311711	TPs
TP012_bin.9	TPs-VS-EPs	5.05E-07	9.40E-07	TPs
TP045_bin.14	TPs-VS-EPs	5.28E-13	1.51E-12	TPs
ERR1135200_bin.73	TPs-VS-EPs	8.54E-08	1.71E-07	TPs
TP015MC_bin.36	TPs-VS-EPs	9.03E-14	2.73E-13	TPs
TP020MC_bin.105	TPs-VS-EPs	2.74E-19	1.18E-18	TPs
TP002_bin.106	TPs-VS-EPs	5.05E-07	9.40E-07	TPs
ERR1135222_bin.9	TPs-VS-EPs	0.002855762	0.004009803	EPs
ERR1135199_bin.55	TPs-VS-EPs	1.84E-09	4.15E-09	EPs
ERR1135297_bin.27	TPs-VS-EPs	9.28E-06	1.61E-05	EPs
TP017MC_bin.2	TPs-VS-EPs	9.13E-13	2.58E-12	TPs
ERR1135289_bin.22	TPs-VS-EPs	0.000318506	0.000493901	EPs
ERR1135307_bin.30	TPs-VS-EPs	1.19E-05	2.04E-05	EPs
ERR1135459_bin.47	TPs-VS-EPs	1.22E-06	2.22E-06	EPs
ERR1135294_bin.61	TPs-VS-EPs	2.50E-16	9.00E-16	EPs
ERR1135351_bin.65	TPs-VS-EPs	4.82E-05	7.84E-05	TPs
ERR1135197_bin.2	TPs-VS-EPs	8.05E-05	0.000128558	EPs
ERR1135267_bin.21	TPs-VS-EPs	1.01E-20	4.99E-20	TPs
TP042_bin.126	TPs-VS-EPs	2.72E-06	4.84E-06	TPs
TP009_bin.49	TPs-VS-EPs	4.46E-30	5.50E-29	TPs
ERR1135229_bin.55	TPs-VS-EPs	3.53E-08	7.30E-08	EPs
TP014_bin.35	TPs-VS-EPs	0.000713628	0.001067837	TPs
TP053MC_bin.2	TPs-VS-EPs	5.55E-26	4.70E-25	TPs
TP023_bin.58	TPs-VS-EPs	1.16E-05	1.99E-05	EPs
TP023_bin.70	TPs-VS-EPs	3.70E-18	1.53E-17	TPs
TP059MC_bin.116	TPs-VS-EPs	5.13E-30	6.19E-29	TPs
TP037MC_bin.21	TPs-VS-EPs	1.57E-41	6.99E-40	TPs
TP034MC_bin.53	TPs-VS-EPs	2.07E-11	5.35E-11	TPs
TP024_bin.125	TPs-VS-EPs	1.45E-22	8.65E-22	TPs
ERR1135178_bin.34	TPs-VS-EPs	3.53E-08	7.30E-08	EPs
ERR1135227_bin.69	TPs-VS-EPs	0.000498457	0.000753566	EPs
TP025_bin.98	TPs-VS-EPs	4.20E-16	1.47E-15	TPs
ERR1135312_bin.41	TPs-VS-EPs	0.001203415	0.001764665	EPs
TP059MC_bin.151	TPs-VS-EPs	5.12E-24	3.52E-23	TPs
TP051_bin.134	TPs-VS-EPs	6.83E-17	2.53E-16	TPs
TP058_bin.96	TPs-VS-EPs	1.22E-05	2.09E-05	TPs
ERR1135198_bin.40	TPs-VS-EPs	0.00531124	0.007268386	TPs
TP029_bin.61	TPs-VS-EPs	0.003537711	0.004920154	TPs
TP058_bin.103	TPs-VS-EPs	2.06E-20	9.87E-20	TPs
TP029MC_bin.10	TPs-VS-EPs	9.98E-36	2.01E-34	TPs
TP044MC_bin.96	TPs-VS-EPs	1.45E-22	8.65E-22	TPs
TP043_bin.93	TPs-VS-EPs	4.87E-05	7.90E-05	TPs
TP001_bin.27	TPs-VS-EPs	5.75E-33	8.43E-32	TPs
TP043_bin.51	TPs-VS-EPs	5.54E-08	1.13E-07	TPs
ERR1135462_bin.24	TPs-VS-EPs	1.30E-22	7.93E-22	TPs
TP002_bin.136	TPs-VS-EPs	3.93E-08	8.10E-08	TPs
TP033_bin.8	TPs-VS-EPs	5.56E-10	1.29E-09	TPs
ERR1135219_bin.28	TPs-VS-EPs	4.12E-09	9.13E-09	EPs
TP034MC_bin.156	TPs-VS-EPs	3.06E-12	8.25E-12	TPs
TP027_bin.78	TPs-VS-EPs	7.44E-23	4.59E-22	TPs
ERR1135252_bin.29	TPs-VS-EPs	0.001857522	0.00269008	EPs
ERR1135192_bin.82	TPs-VS-EPs	3.39E-10	8.11E-10	TPs
TP015MC_bin.54	TPs-VS-EPs	4.12E-54	7.04E-52	TPs
ERR1135195_bin.36	TPs-VS-EPs	0.013520248	0.017564328	TPs
ERR1135200_bin.22	TPs-VS-EPs	1.37E-07	2.69E-07	EPs
ERR1135199_bin.64	TPs-VS-EPs	0.005407979	0.007381063	EPs

SGBs	Compared groups	p values	Fdr	Enriched Group
ERR1135226_bin.23	TPs-VS-EPs	2.64E-21	1.39E-20	EPs
ERR1135197_bin.34	TPs-VS-EPs	0.000141135	0.000223246	EPs
ERR1135356_bin.12	TPs-VS-EPs	3.75E-13	1.10E-12	TPs
ERR1135401_bin.33	TPs-VS-EPs	6.78E-08	1.36E-07	EPs
ERR1135388_bin.55	TPs-VS-EPs	9.98E-36	2.01E-34	TPs
TP041_bin.152	TPs-VS-EPs	4.83E-07	9.16E-07	TPs
ERR1135200_bin.65	TPs-VS-EPs	0.011845223	0.015646074	EPs
ERR1135450_bin.9	TPs-VS-EPs	0.015469025	0.019894291	EPs
TP011_bin.6	TPs-VS-EPs	6.47E-21	3.27E-20	TPs
ERR1135178_bin.57	TPs-VS-EPs	0.004374693	0.006010805	EPs
TP027_bin.91	TPs-VS-EPs	6.29E-24	4.30E-23	TPs
TP028_bin.30	TPs-VS-EPs	1.17E-27	1.13E-26	TPs
TP012_bin.137	TPs-VS-EPs	2.39E-17	9.30E-17	TPs
ERR1135273_bin.81	TPs-VS-EPs	0.001857522	0.00269008	EPs
TP004MC_bin.63	TPs-VS-EPs	0.002582297	0.003655876	EPs
TP034MC_bin.90	TPs-VS-EPs	1.78E-10	4.34E-10	TPs
TP036MC_bin.50	TPs-VS-EPs	1.18E-33	1.91E-32	TPs
ERR1135218_bin.58	TPs-VS-EPs	2.65E-08	5.53E-08	EPs
TP015MC_bin.77	TPs-VS-EPs	1.97E-10	4.78E-10	TPs
ERR1135185_bin.73	TPs-VS-EPs	8.68E-16	2.98E-15	EPs
TP043MC_bin.59	TPs-VS-EPs	6.46E-18	2.65E-17	TPs
ERR1135181_bin.2	TPs-VS-EPs	0.028951696	0.036322507	EPs
ERR1135418_bin.17	TPs-VS-EPs	1.14E-05	1.97E-05	EPs
TP015_bin.111	TPs-VS-EPs	2.77E-23	1.76E-22	TPs
ERR1135263_bin.42	TPs-VS-EPs	2.50E-09	5.59E-09	EPs
TP019MC_bin.9	TPs-VS-EPs	1.15E-38	3.17E-37	TPs
ERR1135183_bin.75	TPs-VS-EPs	0.001909174	0.002760089	TPs
TP018MC_bin.185	TPs-VS-EPs	6.83E-17	2.53E-16	TPs
TP021_bin.93	TPs-VS-EPs	0.036027292	0.045034115	TPs
ERR1135290_bin.47	TPs-VS-EPs	0.002855762	0.004009803	EPs
TP027MC_bin.116	TPs-VS-EPs	6.45E-05	0.000103493	EPs
TP007MC_bin.50	TPs-VS-EPs	3.84E-26	3.28E-25	TPs
TP056_bin.56	TPs-VS-EPs	2.91E-34	5.06E-33	TPs
TP027_bin.5	TPs-VS-EPs	1.36E-09	3.10E-09	TPs
TP021_bin.51	TPs-VS-EPs	2.74E-19	1.18E-18	TPs
TP027MC_bin.147	TPs-VS-EPs	1.70E-08	3.59E-08	TPs
TP042_bin.125	TPs-VS-EPs	1.75E-26	1.52E-25	TPs
ERR1135425_bin.34	TPs-VS-EPs	1.80E-06	3.25E-06	TPs
TP058_bin.70	TPs-VS-EPs	6.30E-32	8.72E-31	TPs
ERR1135449_bin.13	TPs-VS-EPs	0.028951696	0.036322507	EPs
ERR1135196_bin.4	TPs-VS-EPs	9.39E-07	1.72E-06	EPs
TP056_bin.100	TPs-VS-EPs	4.09E-32	5.83E-31	TPs
ERR1135219_bin.61	TPs-VS-EPs	0.00012804	0.000202847	EPs
ERR1135217_bin.65	TPs-VS-EPs	3.88E-05	6.36E-05	EPs
ERR1135289_bin.44	TPs-VS-EPs	1.12E-09	2.56E-09	TPs
TP052MC_bin.64	TPs-VS-EPs	0.002633126	0.003722695	TPs
TP018MC_bin.69	TPs-VS-EPs	2.72E-13	8.07E-13	TPs
ERR1135210_bin.54	TPs-VS-EPs	1.99E-05	3.34E-05	TPs
TP036_bin.44	TPs-VS-EPs	2.29E-40	8.71E-39	TPs
TP043MC_bin.127	TPs-VS-EPs	0.028080057	0.035358794	TPs
ERR1135389_bin.43	TPs-VS-EPs	7.24E-11	1.84E-10	TPs
TP045_bin.86	TPs-VS-EPs	9.59E-15	3.11E-14	TPs
TP049MC_bin.106	TPs-VS-EPs	5.05E-07	9.40E-07	TPs
TP059MC_bin.121	TPs-VS-EPs	2.74E-19	1.18E-18	TPs
TP053MC_bin.119	TPs-VS-EPs	1.70E-08	3.59E-08	TPs
TP031MC_bin.46	TPs-VS-EPs	0.008523328	0.011405236	TPs
ERR1135233_bin.32	TPs-VS-EPs	0.037855825	0.047204648	EPs
ERR1135270_bin.77	TPs-VS-EPs	2.60E-18	1.08E-17	EPs
ERR1135308_bin.99	TPs-VS-EPs	2.42E-14	7.54E-14	EPs
ERR1135370_bin.25	TPs-VS-EPs	4.05E-05	6.60E-05	TPs
TP015_bin.103	TPs-VS-EPs	3.06E-12	8.25E-12	TPs
TP040MC_bin.126	TPs-VS-EPs	0.013168665	0.017129292	TPs
TP017MC_bin.98	TPs-VS-EPs	8.57E-27	7.70E-26	TPs
ERR1135342_bin.65	TPs-VS-EPs	0.01254834	0.016532196	EPs
TP011_bin.90	TPs-VS-EPs	1.75E-11	4.55E-11	TPs
ERR1135209_bin.24	TPs-VS-EPs	1.27E-10	3.12E-10	TPs
TP027MC_bin.2	TPs-VS-EPs	0.001619065	0.002367392	TPs
ERR1135232_bin.36	TPs-VS-EPs	0.003767365	0.00522537	TPs
TP006MC_bin.130	TPs-VS-EPs	1.75E-18	7.37E-18	TPs
TP036_bin.108	TPs-VS-EPs	5.28E-13	1.51E-12	TPs
ERR1135245_bin.31	TPs-VS-EPs	1.11E-08	2.38E-08	EPs
TP014MC_bin.66	TPs-VS-EPs	1.16E-21	6.40E-21	TPs
TP021_bin.56	TPs-VS-EPs	2.59E-10	6.22E-10	TPs
ERR1135328_bin.18	TPs-VS-EPs	3.30E-19	1.42E-18	EPs
TP015MC_bin.62	TPs-VS-EPs	1.75E-18	7.37E-18	TPs
TP021_bin.7	TPs-VS-EPs	8.45E-36	1.88E-34	TPs
TP002_bin.148	TPs-VS-EPs	6.83E-17	2.53E-16	TPs
ERR1135227_bin.18	TPs-VS-EPs	3.13E-05	5.15E-05	EPs
TP012_bin.62	TPs-VS-EPs	9.55E-07	1.75E-06	TPs
TP029_bin.43	TPs-VS-EPs	4.28E-09	9.44E-09	TPs
TP034MC_bin.30	TPs-VS-EPs	1.58E-28	1.63E-27	TPs
ERR1135385_bin.11	TPs-VS-EPs	2.75E-05	4.53E-05	TPs
ERR1135225_bin.13	TPs-VS-EPs	3.94E-08	8.10E-08	EPs
ERR1135269_bin.35	TPs-VS-EPs	1.07E-10	2.65E-10	EPs
TP045_bin.123	TPs-VS-EPs	1.70E-10	4.15E-10	TPs
ERR1135220_bin.65	TPs-VS-EPs	2.47E-07	4.78E-07	EPs
ERR1135270_bin.3	TPs-VS-EPs	6.15E-09	1.34E-08	EPs
TP036_bin.120	TPs-VS-EPs	2.76E-10	6.62E-10	TPs
TP008_bin.61	TPs-VS-EPs	6.83E-17	2.53E-16	TPs
TP021MC_bin.72	TPs-VS-EPs	9.30E-08	1.84E-07	TPs
TP006MC_bin.52	TPs-VS-EPs	5.56E-10	1.29E-09	TPs
TP044MC_bin.16	TPs-VS-EPs	2.15E-23	1.39E-22	TPs
TP052MC_bin.82	TPs-VS-EPs	1.10E-35	2.18E-34	TPs
ERR1135275_bin.2	TPs-VS-EPs	0.000498457	0.000753566	EPs
ERR1135219_bin.51	TPs-VS-EPs	4.56E-09	9.99E-09	EPs

SGBs	Compared groups	p values	Fdr	Enriched Group
TP018_bin.78	TPs-VS-EPs	2.46E-23	1.57E-22	TPs
TP043_bin.40	TPs-VS-EPs	6.47E-08	1.30E-07	TPs
TP036MC_bin.106	TPs-VS-EPs	9.76E-22	5.47E-21	TPs
ERR1135275_bin.62	TPs-VS-EPs	2.21E-05	3.69E-05	TPs
TP015_bin.34	TPs-VS-EPs	4.47E-51	5.73E-49	TPs
ERR1135277_bin.54	TPs-VS-EPs	9.94E-10	2.28E-09	EPs
ERR1135224_bin.9	TPs-VS-EPs	1.74E-27	1.64E-26	EPs
TP015_bin.30	TPs-VS-EPs	1.81E-07	3.53E-07	TPs
TP041_bin.34	TPs-VS-EPs	7.16E-15	2.34E-14	TPs
ERR1135265_bin.18	TPs-VS-EPs	0.001787395	0.002606088	EPs
ERR1135217_bin.5	TPs-VS-EPs	0.002254317	0.0032272	EPs
ERR1135201_bin.83	TPs-VS-EPs	0.019064642	0.024305047	EPs
TP051_bin.142	TPs-VS-EPs	5.77E-21	2.99E-20	TPs
TP040MC_bin.137	TPs-VS-EPs	6.82E-17	2.53E-16	TPs
TP053MC_bin.93	TPs-VS-EPs	2.23E-07	4.34E-07	TPs
TP015MC_bin.135	TPs-VS-EPs	0.000425407	0.000650809	TPs
TP024MC_bin.53	TPs-VS-EPs	8.36E-35	1.48E-33	TPs
TP014MC_bin.156	TPs-VS-EPs	1.37E-17	5.41E-17	TPs
TP018MC_bin.177	TPs-VS-EPs	1.51E-15	5.13E-15	TPs
TP037MC_bin.37	TPs-VS-EPs	0.000425407	0.000650809	TPs
ERR1135193_bin.27	TPs-VS-EPs	0.006591234	0.008924722	EPs
ERR1135246_bin.54	TPs-VS-EPs	1.07E-08	2.31E-08	EPs
TP001_bin.90	TPs-VS-EPs	6.12E-29	6.82E-28	TPs
TP005_bin.95	TPs-VS-EPs	1.93E-14	6.10E-14	EPs
ERR1135464_bin.16	TPs-VS-EPs	7.78E-12	2.05E-11	TPs
ERR1135291_bin.88	TPs-VS-EPs	0.023493224	0.029765828	EPs
TP043_bin.118	TPs-VS-EPs	1.17E-27	1.13E-26	TPs
ERR1135221_bin.12	TPs-VS-EPs	5.10E-18	2.10E-17	EPs
TP043MC_bin.22	TPs-VS-EPs	6.83E-17	2.53E-16	TPs
ERR1135205_bin.43	TPs-VS-EPs	0.007703113	0.010375415	EPs
ERR1135196_bin.11	TPs-VS-EPs	2.42E-29	2.75E-28	EPs
ERR1135341_bin.54	TPs-VS-EPs	1.11E-08	2.38E-08	EPs
TP026_bin.154	TPs-VS-EPs	9.92E-11	2.47E-10	TPs
ERR1135350_bin.18	TPs-VS-EPs	1.19E-05	2.04E-05	EPs
ERR1135200_bin.12	TPs-VS-EPs	4.62E-12	1.24E-11	TPs
ERR1135196_bin.55	TPs-VS-EPs	4.39E-06	7.76E-06	EPs
ERR1135205_bin.8	TPs-VS-EPs	5.99E-30	7.14E-29	TPs
TP043MC_bin.109	TPs-VS-EPs	9.92E-11	2.47E-10	TPs
ERR1135211_bin.33	TPs-VS-EPs	5.19E-08	1.06E-07	TPs
TP002_bin.164	TPs-VS-EPs	1.96E-42	9.14E-41	TPs
TP051_bin.70	TPs-VS-EPs	5.80E-48	5.94E-46	TPs
TP043MC_bin.142	TPs-VS-EPs	1.06E-30	1.38E-29	TPs
ERR1135275_bin.38	TPs-VS-EPs	0.000622418	0.000932718	EPs
TP040MC_bin.98	TPs-VS-EPs	1.96E-44	1.18E-42	TPs
ERR1135197_bin.41	TPs-VS-EPs	7.13E-06	1.25E-05	TPs
TP020_bin.111	TPs-VS-EPs	1.40E-26	1.22E-25	EPs
ERR1135456_bin.15	TPs-VS-EPs	0.000622418	0.000932718	EPs
ERR1135265_bin.33	TPs-VS-EPs	6.02E-06	1.06E-05	TPs
TP026_bin.65	TPs-VS-EPs	5.21E-08	1.06E-07	TPs
ERR1135299_bin.83	TPs-VS-EPs	0.001203415	0.001764665	EPs
TP045_bin.48	TPs-VS-EPs	4.83E-17	1.86E-16	TPs
ERR1135190_bin.64	TPs-VS-EPs	1.31E-15	4.45E-15	EPs
TP025_bin.6	TPs-VS-EPs	1.46E-05	2.49E-05	TPs
TP036_bin.122	TPs-VS-EPs	4.46E-14	1.37E-13	TPs
TP021MC_bin.139	TPs-VS-EPs	5.05E-07	9.40E-07	TPs
TP027MC_bin.38	TPs-VS-EPs	2.72E-06	4.84E-06	TPs
TP042_bin.127	TPs-VS-EPs	9.34E-20	4.29E-19	TPs
TP058_bin.84	TPs-VS-EPs	1.91E-21	1.02E-20	TPs
ERR1135252_bin.17	TPs-VS-EPs	1.91E-13	5.71E-13	TPs
ERR1135330_bin.69	TPs-VS-EPs	1.65E-15	5.60E-15	EPs
ERR1135202_bin.1	TPs-VS-EPs	0.019064642	0.024305047	EPs
TP030MC_bin.59	TPs-VS-EPs	4.41E-15	1.45E-14	TPs
ERR1135260_bin.22	TPs-VS-EPs	0.000498457	0.000753566	EPs
TP025_bin.138	TPs-VS-EPs	5.93E-09	1.29E-08	TPs
TP043_bin.130	TPs-VS-EPs	2.18E-05	3.64E-05	TPs
TP040MC_bin.9	TPs-VS-EPs	3.06E-12	8.25E-12	TPs
TP012_bin.29	TPs-VS-EPs	9.69E-08	1.91E-07	TPs
TP045_bin.32	TPs-VS-EPs	1.49E-21	8.18E-21	TPs
TP018_bin.55	TPs-VS-EPs	3.98E-11	1.03E-10	TPs
TP041MC_bin.110	TPs-VS-EPs	8.34E-16	2.90E-15	TPs
ERR1135419_bin.22	TPs-VS-EPs	3.24E-07	6.18E-07	EPs
TP015_bin.88	TPs-VS-EPs	1.01E-05	1.74E-05	TPs
TP025_bin.45	TPs-VS-EPs	2.38E-44	1.28E-42	TPs
TP034MC_bin.55	TPs-VS-EPs	4.48E-18	1.85E-17	TPs
TP043_bin.119	TPs-VS-EPs	9.03E-14	2.73E-13	TPs
ERR1135202_bin.14	TPs-VS-EPs	0.036563758	0.045649027	TPs
ERR1135341_bin.51	TPs-VS-EPs	2.07E-19	9.14E-19	EPs
ERR1135342_bin.51	TPs-VS-EPs	8.68E-16	2.98E-15	EPs
ERR1135266_bin.58	TPs-VS-EPs	9.56E-18	3.87E-17	EPs
TP051_bin.6	TPs-VS-EPs	6.80E-12	1.80E-11	TPs
ERR1135277_bin.10	TPs-VS-EPs	0.019064642	0.024305047	EPs
ERR1135181_bin.32	TPs-VS-EPs	0.002304275	0.00328953	EPs
ERR1135219_bin.8	TPs-VS-EPs	2.17E-05	3.63E-05	EPs
TP025_bin.136	TPs-VS-EPs	3.96E-12	1.07E-11	TPs
ERR1135191_bin.71	TPs-VS-EPs	0.004374693	0.006010805	EPs
ERR1135232_bin.13	TPs-VS-EPs	0.000498457	0.000753566	EPs
ERR1135389_bin.61	TPs-VS-EPs	0.019064642	0.024305047	EPs
TP001_bin.117	TPs-VS-EPs	2.54E-15	8.49E-15	TPs
ERR1135199_bin.23	TPs-VS-EPs	1.43E-07	2.80E-07	EPs
ERR1135453_bin.41	TPs-VS-EPs	1.82E-25	1.51E-24	EPs
TP020MC_bin.75	TPs-VS-EPs	2.66E-28	2.70E-27	TPs
TP034MC_bin.157	TPs-VS-EPs	5.28E-13	1.51E-12	TPs
TP027_bin.157	TPs-VS-EPs	0.001992328	0.002864144	TPs
TP012_bin.39	TPs-VS-EPs	2.36E-25	1.89E-24	TPs
ERR1135304_bin.69	TPs-VS-EPs	4.39E-06	7.76E-06	EPs

SGBs	Compared groups	p values	Fdr	Enriched Group
ERR1135302_bin.36	TPs-VS-EPs	0.000728564	0.001087013	TPs
ERR1135223_bin.20	TPs-VS-EPs	0.02107131	0.026796641	EPs
TP002_bin.8	TPs-VS-EPs	3.05E-24	2.19E-23	TPs
TP049MC_bin.131	TPs-VS-EPs	0.003816133	0.005278726	TPs
ERR1135371_bin.19	TPs-VS-EPs	1.36E-09	3.10E-09	TPs
ERR1135383_bin.11	TPs-VS-EPs	6.97E-06	1.22E-05	TPs
ERR1135260_bin.46	TPs-VS-EPs	0.023493224	0.029765828	EPs
TP021MC_bin.45	TPs-VS-EPs	1.71E-05	2.88E-05	TPs
ERR1135306_bin.47	TPs-VS-EPs	2.50E-09	5.59E-09	EPs
TP026_bin.108	TPs-VS-EPs	6.88E-12	1.82E-11	TPs
ERR1135206_bin.69	TPs-VS-EPs	6.19E-05	9.97E-05	EPs
TP051_bin.64	TPs-VS-EPs	2.22E-18	9.28E-18	TPs
ERR1135371_bin.25	TPs-VS-EPs	1.64E-05	2.76E-05	TPs
ERR1135205_bin.52	TPs-VS-EPs	2.24E-14	6.98E-14	TPs
TP049MC_bin.7	TPs-VS-EPs	2.83E-07	5.46E-07	TPs
TP018MC_bin.74	TPs-VS-EPs	3.21E-24	2.27E-23	TPs
TP047MC_bin.9	TPs-VS-EPs	4.47E-33	7.05E-32	TPs
TP059MC_bin.84	TPs-VS-EPs	5.28E-13	1.51E-12	TPs
TP041MC_bin.31	TPs-VS-EPs	5.56E-10	1.29E-09	TPs
TP029_bin.154	TPs-VS-EPs	2.74E-19	1.18E-18	TPs
TP049MC_bin.125	TPs-VS-EPs	5.43E-09	1.19E-08	TPs
TP051_bin.108	TPs-VS-EPs	2.13E-23	1.38E-22	TPs
TP049MC_bin.114	TPs-VS-EPs	0.013168665	0.017129292	TPs
TP022MC_bin.63	TPs-VS-EPs	3.20E-36	7.63E-35	TPs
ERR1135178_bin.9	TPs-VS-EPs	0.000154474	0.000243593	EPs
TP027_bin.153	TPs-VS-EPs	1.92E-36	4.68E-35	TPs
TP004MC_bin.66	TPs-VS-EPs	1.84E-07	3.59E-07	TPs
TP025_bin.106	TPs-VS-EPs	6.47E-21	3.27E-20	TPs
ERR1135350_bin.27	TPs-VS-EPs	3.93E-05	6.43E-05	TPs
TP043_bin.98	TPs-VS-EPs	4.06E-30	5.07E-29	TPs
TP020MC_bin.144	TPs-VS-EPs	5.56E-10	1.29E-09	TPs
TP052MC_bin.61	TPs-VS-EPs	2.69E-16	9.66E-16	TPs
ERR1135233_bin.45	TPs-VS-EPs	0.000398697	0.000614534	EPs
TP004MC_bin.67	TPs-VS-EPs	4.04E-10	9.60E-10	TPs
ERR1135397_bin.48	TPs-VS-EPs	0.002329506	0.003311711	TPs
ERR1135407_bin.19	TPs-VS-EPs	1.54E-38	4.16E-37	TPs
ERR1135216_bin.67	TPs-VS-EPs	9.68E-14	2.92E-13	EPs
ERR1135348_bin.2	TPs-VS-EPs	1.60E-21	8.69E-21	EPs
TP041_bin.136	TPs-VS-EPs	3.20E-39	9.93E-38	TPs
ERR1135311_bin.91	TPs-VS-EPs	4.56E-09	9.99E-09	EPs
TP006MC_bin.14	TPs-VS-EPs	6.47E-12	1.72E-11	TPs
TP059MC_bin.127	TPs-VS-EPs	2.07E-41	8.14E-40	TPs
ERR1135214_bin.3	TPs-VS-EPs	8.82E-13	2.50E-12	EPs
TP019MC_bin.30	TPs-VS-EPs	3.44E-27	3.21E-26	TPs
TP026_bin.87	TPs-VS-EPs	2.13E-23	1.38E-22	TPs
TP033_bin.130	TPs-VS-EPs	8.05E-13	2.29E-12	TPs
ERR1135410_bin.13	TPs-VS-EPs	3.71E-07	7.04E-07	TPs
TP005_bin.79	TPs-VS-EPs	3.58E-15	1.19E-14	EPs
TP018_bin.26	TPs-VS-EPs	5.67E-22	3.23E-21	TPs
ERR1135224_bin.55	TPs-VS-EPs	0.002304275	0.00328953	EPs
TP043_bin.18	TPs-VS-EPs	1.70E-08	3.59E-08	TPs
TP020MC_bin.157	TPs-VS-EPs	8.57E-27	7.70E-26	TPs
TP053MC_bin.38	TPs-VS-EPs	1.70E-08	3.59E-08	TPs
ERR1135202_bin.31	TPs-VS-EPs	0.040122765	0.049909993	EPs
ERR1135183_bin.4	TPs-VS-EPs	2.14E-08	4.49E-08	TPs
ERR1135259_bin.56	TPs-VS-EPs	4.77E-33	7.40E-32	TPs
TP029MC_bin.20	TPs-VS-EPs	9.92E-11	2.47E-10	TPs
ERR1135348_bin.60	TPs-VS-EPs	3.25E-05	5.34E-05	EPs
ERR1135253_bin.21	TPs-VS-EPs	2.38E-06	4.28E-06	TPs
ERR1135262_bin.62	TPs-VS-EPs	6.63E-08	1.33E-07	TPs
ERR1135458_bin.47	TPs-VS-EPs	6.47E-21	3.27E-20	TPs
TP012_bin.3	TPs-VS-EPs	1.54E-28	1.63E-27	TPs
ERR1135179_bin.29	TPs-VS-EPs	0.01254834	0.016532196	EPs
ERR1135196_bin.50	TPs-VS-EPs	4.76E-08	9.71E-08	EPs
ERR1135319_bin.63	TPs-VS-EPs	0.02503346	0.031600118	EPs
ERR1135360_bin.47	TPs-VS-EPs	1.16E-26	1.03E-25	TPs
TP011_bin.85	TPs-VS-EPs	9.54E-25	7.04E-24	TPs
ERR1135262_bin.43	TPs-VS-EPs	8.60E-06	1.50E-05	TPs
ERR1135188_bin.29	TPs-VS-EPs	0.008379213	0.011227051	EPs
TP025_bin.18	TPs-VS-EPs	1.09E-38	3.12E-37	TPs
TP025MC_bin.148	TPs-VS-EPs	5.05E-07	9.40E-07	TPs
TP027_bin.147	TPs-VS-EPs	2.22E-05	3.70E-05	TPs
ERR1135293_bin.44	TPs-VS-EPs	4.27E-13	1.25E-12	EPs
ERR1135373_bin.22	TPs-VS-EPs	5.08E-24	3.52E-23	EPs
ERR1135212_bin.26	TPs-VS-EPs	2.69E-14	8.36E-14	TPs
ERR1135190_bin.63	TPs-VS-EPs	6.37E-05	0.000102369	EPs
ERR1135288_bin.96	TPs-VS-EPs	0.000148225	0.0002341	EPs
TP001_bin.18	TPs-VS-EPs	1.70E-08	3.59E-08	TPs
TP001_bin.56	TPs-VS-EPs	1.78E-43	9.10E-42	TPs
ERR1135300_bin.25	TPs-VS-EPs	2.50E-16	9.00E-16	EPs
ERR1135216_bin.5	TPs-VS-EPs	8.01E-15	2.61E-14	EPs
ERR1135276_bin.56	TPs-VS-EPs	3.97E-05	6.48E-05	EPs
TP033_bin.32	TPs-VS-EPs	3.74E-20	1.77E-19	TPs
TP041_bin.163	TPs-VS-EPs	4.20E-16	1.47E-15	TPs
ERR1135347_bin.12	TPs-VS-EPs	0.019064642	0.024305047	EPs
TP008MC_bin.139	TPs-VS-EPs	1.70E-08	3.59E-08	TPs
ERR1135401_bin.43	TPs-VS-EPs	0.002022473	0.00289935	EPs
TP030MC_bin.27	TPs-VS-EPs	1.16E-07	2.28E-07	TPs
ERR1135204_bin.18	TPs-VS-EPs	7.29E-20	3.36E-19	TPs
ERR1135189_bin.8	TPs-VS-EPs	3.24E-07	6.18E-07	EPs
TP034MC_bin.40	TPs-VS-EPs	3.61E-20	1.71E-19	TPs
TP012MC_bin.85	TPs-VS-EPs	5.56E-10	1.29E-09	TPs
TP012MC_bin.25	TPs-VS-EPs	1.70E-08	3.59E-08	TPs
TP024_bin.56	TPs-VS-EPs	6.90E-34	1.18E-32	TPs
ERR1135209_bin.29	TPs-VS-EPs	1.59E-07	3.12E-07	TPs

SGBs	Compared groups	p values	Fdr	Enriched Group
ERR1135223_bin.52	TPs-VS-EPs	1.58E-06	2.86E-06	EPs
ERR1135262_bin.42	TPs-VS-EPs	1.09E-06	1.99E-06	TPs
ERR1135324_bin.51	TPs-VS-EPs	0.000254119	0.000394654	EPs
ERR1135468_bin.57	TPs-VS-EPs	0.024309642	0.030762201	EPs
TP002_bin.84	TPs-VS-EPs	0.000425407	0.000650809	TPs
TP018MC_bin.85	TPs-VS-EPs	2.20E-23	1.41E-22	TPs
TP019MC_bin.95	TPs-VS-EPs	9.03E-14	2.73E-13	TPs
ERR1135191_bin.69	TPs-VS-EPs	0.001203415	0.001764665	EPs
ERR1135466_bin.58	TPs-VS-EPs	0.003297096	0.004623151	TPs
TP031MC_bin.51	TPs-VS-EPs	6.45E-23	4.00E-22	TPs
TP043_bin.129	TPs-VS-EPs	5.56E-10	1.29E-09	TPs
TP056_bin.102	TPs-VS-EPs	0.00183245	0.002667984	TPs
ERR1135201_bin.79	TPs-VS-EPs	0.014622012	0.018971598	TPs
TP014MC_bin.90	TPs-VS-EPs	1.45E-22	8.65E-22	TPs
TP011_bin.101	TPs-VS-EPs	0.000450868	0.000688733	TPs
TP053MC_bin.136	TPs-VS-EPs	7.39E-55	1.89E-52	TPs
ERR1135191_bin.33	TPs-VS-EPs	2.60E-12	7.15E-12	TPs
TP036MC_bin.76	TPs-VS-EPs	1.80E-10	4.38E-10	TPs
ERR1135350_bin.23	TPs-VS-EPs	3.24E-07	6.18E-07	EPs
ERR1135219_bin.41	TPs-VS-EPs	0.000161125	0.000253303	EPs
ERR1135406_bin.22	TPs-VS-EPs	2.10E-14	6.59E-14	EPs
TP045_bin.120	TPs-VS-EPs	2.86E-08	5.96E-08	TPs
TP005_bin.3	TPs-VS-EPs	9.96E-17	3.65E-16	TPs
ERR1135213_bin.46	TPs-VS-EPs	5.96E-08	1.21E-07	TPs
ERR1135291_bin.83	TPs-VS-EPs	9.79E-25	7.17E-24	EPs
TP010_bin.76	TPs-VS-EPs	9.03E-14	2.73E-13	TPs
ERR1135453_bin.49	TPs-VS-EPs	0.010820913	0.014367144	TPs
TP021MC_bin.53	TPs-VS-EPs	4.41E-15	1.45E-14	TPs
TP040MC_bin.156	TPs-VS-EPs	6.83E-17	2.53E-16	TPs
ERR1135231_bin.4	TPs-VS-EPs	2.88E-05	4.74E-05	EPs
ERR1135196_bin.29	TPs-VS-EPs	1.41E-12	3.92E-12	TPs
TP014_bin.67	TPs-VS-EPs	1.75E-10	4.27E-10	TPs
ERR1135208_bin.43	TPs-VS-EPs	0.0019931	0.002864144	TPs
ERR1135189_bin.31	TPs-VS-EPs	1.46E-05	2.49E-05	TPs
ERR1135379_bin.27	TPs-VS-EPs	8.82E-05	0.000140414	EPs
ERR1135252_bin.35	TPs-VS-EPs	0.000498457	0.000753566	EPs
TP045_bin.129	TPs-VS-EPs	1.52E-14	4.82E-14	TPs
TP043_bin.81	TPs-VS-EPs	5.05E-07	9.40E-07	TPs
ERR1135378_bin.53	TPs-VS-EPs	1.46E-05	2.49E-05	TPs
ERR1135447_bin.18	TPs-VS-EPs	8.27E-09	1.79E-08	EPs
TP002_bin.4	TPs-VS-EPs	5.09E-05	8.21E-05	TPs
TP051_bin.119	TPs-VS-EPs	4.24E-20	1.97E-19	TPs
ERR1135417_bin.29	TPs-VS-EPs	2.37E-09	5.32E-09	TPs
ERR1135273_bin.2	TPs-VS-EPs	5.64E-06	9.94E-06	EPs
ERR1135426_bin.54	TPs-VS-EPs	4.32E-09	9.53E-09	EPs
ERR1135187_bin.93	TPs-VS-EPs	0.000622418	0.000932718	EPs
TP027MC_bin.153	TPs-VS-EPs	0.02665132	0.033600987	TPs
TP006MC_bin.74	TPs-VS-EPs	4.39E-25	3.29E-24	TPs
TP047MC_bin.136	TPs-VS-EPs	1.10E-17	4.37E-17	TPs
ERR1135472_bin.8	TPs-VS-EPs	5.29E-20	2.45E-19	EPs
TP051_bin.24	TPs-VS-EPs	6.41E-12	1.71E-11	TPs
TP052MC_bin.93	TPs-VS-EPs	9.30E-08	1.84E-07	TPs
ERR1135212_bin.6	TPs-VS-EPs	4.54E-09	9.99E-09	TPs
ERR1135220_bin.68	TPs-VS-EPs	0.022029042	0.027979886	TPs
ERR1135247_bin.55	TPs-VS-EPs	0.003536051	0.004920154	EPs
ERR1135422_bin.56	TPs-VS-EPs	4.12E-08	8.45E-08	TPs
TP018_bin.105	TPs-VS-EPs	2.21E-13	6.55E-13	TPs
TP020_bin.5	TPs-VS-EPs	3.11E-14	9.63E-14	TPs
ERR1135190_bin.88	TPs-VS-EPs	1.09E-17	4.37E-17	TPs
ERR1135215_bin.52	TPs-VS-EPs	2.65E-08	5.53E-08	EPs
ERR1135283_bin.47	TPs-VS-EPs	2.46E-05	4.08E-05	EPs
TP034MC_bin.112	TPs-VS-EPs	1.67E-05	2.82E-05	TPs
TP040MC_bin.35	TPs-VS-EPs	0.013168665	0.017129292	TPs
ERR1135220_bin.1	TPs-VS-EPs	0.01119584	0.014826533	EPs
TP009_bin.66	TPs-VS-EPs	2.00E-28	2.05E-27	TPs
ERR1135268_bin.39	TPs-VS-EPs	0.015469025	0.019894291	EPs
ERR1135208_bin.72	TPs-VS-EPs	0.00793382	0.010644195	TPs
ERR1135336_bin.80	TPs-VS-EPs	0.002855762	0.004009803	EPs
ERR1135194_bin.68	TPs-VS-EPs	1.80E-12	5.01E-12	EPs
TP058_bin.85	TPs-VS-EPs	3.70E-07	7.03E-07	TPs
TP025_bin.139	TPs-VS-EPs	9.03E-14	2.73E-13	TPs
TP002_bin.67	TPs-VS-EPs	4.34E-11	1.12E-10	TPs
ERR1135276_bin.61	TPs-VS-EPs	9.39E-07	1.72E-06	EPs
TP018MC_bin.115	TPs-VS-EPs	1.21E-12	3.41E-12	TPs
ERR1135273_bin.8	TPs-VS-EPs	7.21E-07	1.33E-06	EPs
TP059MC_bin.25	TPs-VS-EPs	2.75E-07	5.30E-07	TPs
TP044MC_bin.62	TPs-VS-EPs	1.42E-20	6.97E-20	TPs
TP015_bin.39	TPs-VS-EPs	4.39E-25	3.29E-24	TPs
TP025_bin.31	TPs-VS-EPs	4.88E-10	1.16E-09	TPs
TP052MC_bin.34	TPs-VS-EPs	3.89E-22	2.24E-21	TPs
TP040MC_bin.111	TPs-VS-EPs	1.10E-17	4.37E-17	TPs
ERR1135378_bin.9	TPs-VS-EPs	1.31E-15	4.45E-15	EPs
ERR1135273_bin.39	TPs-VS-EPs	1.09E-07	2.14E-07	EPs
ERR1135217_bin.14	TPs-VS-EPs	2.46E-05	4.08E-05	EPs
ERR1135291_bin.45	TPs-VS-EPs	2.05E-06	3.68E-06	EPs
TP056_bin.37	TPs-VS-EPs	0.000981974	0.001454513	TPs
TP043_bin.169	TPs-VS-EPs	8.86E-57	9.08E-54	TPs
ERR1135295_bin.34	TPs-VS-EPs	6.21E-08	1.25E-07	EPs
ERR1135205_bin.45	TPs-VS-EPs	5.23E-06	9.22E-06	TPs
TP007MC_bin.20	TPs-VS-EPs	1.17E-27	1.13E-26	TPs
TP015MC_bin.18	TPs-VS-EPs	3.49E-09	7.75E-09	TPs
ERR1135293_bin.33	TPs-VS-EPs	8.68E-16	2.98E-15	EPs
TP052MC_bin.45	TPs-VS-EPs	0.013168665	0.017129292	TPs
ERR1135275_bin.52	TPs-VS-EPs	5.33E-11	1.36E-10	TPs
TP010_bin.100	TPs-VS-EPs	1.73E-39	5.54E-38	TPs

SGBs	Compared groups	p values	Fdr	Enriched Group
ERR1135309_bin.22	TPs-VS-EPs	3.24E-07	6.18E-07	EPs
ERR1135272_bin.36	TPs-VS-EPs	6.36E-05	0.000102369	EPs
TP014_bin.39	TPs-VS-EPs	5.28E-13	1.51E-12	TPs
TP045_bin.1	TPs-VS-EPs	4.24E-20	1.97E-19	TPs
TP024MC_bin.130	TPs-VS-EPs	7.21E-29	7.95E-28	TPs
ERR1135286_bin.7	TPs-VS-EPs	9.39E-06	1.63E-05	EPs
ERR1135295_bin.8	TPs-VS-EPs	2.05E-06	3.68E-06	EPs
TP011_bin.97	TPs-VS-EPs	7.18E-09	1.56E-08	TPs
TP019MC_bin.103	TPs-VS-EPs	1.46E-05	2.49E-05	TPs
TP027MC_bin.27	TPs-VS-EPs	2.28E-22	1.35E-21	TPs
TP021_bin.115	TPs-VS-EPs	1.26E-06	2.29E-06	TPs
ERR1135203_bin.24	TPs-VS-EPs	0.003536051	0.004920154	EPs
ERR1135213_bin.14	TPs-VS-EPs	0.028951696	0.036322507	EPs
ERR1135460_bin.61	TPs-VS-EPs	7.24E-06	1.26E-05	EPs
TP021_bin.156	TPs-VS-EPs	2.26E-07	4.38E-07	TPs
TP009_bin.103	TPs-VS-EPs	3.45E-15	1.15E-14	TPs
TP023_bin.85	TPs-VS-EPs	1.66E-53	2.43E-51	TPs
TP015MC_bin.75	TPs-VS-EPs	8.33E-21	4.19E-20	TPs
TP036_bin.7	TPs-VS-EPs	1.54E-19	6.91E-19	TPs
ERR1135419_bin.8	TPs-VS-EPs	7.21E-07	1.33E-06	EPs
TP058_bin.105	TPs-VS-EPs	4.20E-16	1.47E-15	TPs
TP036_bin.80	TPs-VS-EPs	2.72E-06	4.84E-06	TPs
ERR1135359_bin.27	TPs-VS-EPs	0.000398697	0.000614534	EPs
ERR1135215_bin.21	TPs-VS-EPs	0.000622418	0.000932718	EPs
ERR1135274_bin.69	TPs-VS-EPs	1.26E-12	3.53E-12	EPs
ERR1135351_bin.45	TPs-VS-EPs	1.52E-05	2.57E-05	EPs
ERR1135191_bin.44	TPs-VS-EPs	0.000501354	0.00075683	TPs
ERR1135352_bin.30	TPs-VS-EPs	1.68E-19	7.50E-19	EPs
TP059MC_bin.181	TPs-VS-EPs	0.013168665	0.017129292	TPs
TP037MC_bin.162	TPs-VS-EPs	9.92E-11	2.47E-10	TPs
TP004MC_bin.41	TPs-VS-EPs	4.46E-14	1.37E-13	TPs
TP007MC_bin.55	TPs-VS-EPs	5.05E-07	9.40E-07	TPs
TP018_bin.85	TPs-VS-EPs	0.001979131	0.002853177	TPs
TP045_bin.23	TPs-VS-EPs	5.31E-11	1.36E-10	TPs
ERR1135241_bin.71	TPs-VS-EPs	0.035686545	0.044662648	EPs
TP044MC_bin.58	TPs-VS-EPs	2.00E-35	3.87E-34	TPs
TP003MC_bin.13	TPs-VS-EPs	4.60E-12	1.23E-11	TPs
ERR1135188_bin.12	TPs-VS-EPs	0.002428751	0.00344325	TPs
ERR1135261_bin.34	TPs-VS-EPs	1.27E-05	2.17E-05	TPs
ERR1135266_bin.67	TPs-VS-EPs	0.000128041	0.000202847	EPs
ERR1135311_bin.79	TPs-VS-EPs	3.88E-10	9.25E-10	EPs
ERR1135334_bin.33	TPs-VS-EPs	2.04E-11	5.30E-11	EPs
TP031MC_bin.105	TPs-VS-EPs	8.36E-35	1.48E-33	TPs
TP027_bin.100	TPs-VS-EPs	2.54E-15	8.49E-15	TPs
TP040MC_bin.40	TPs-VS-EPs	2.74E-30	3.51E-29	TPs
ERR1135465_bin.9	TPs-VS-EPs	8.00E-05	0.000127853	TPs
ERR1135260_bin.40	TPs-VS-EPs	5.68E-07	1.06E-06	TPs
ERR1135458_bin.70	TPs-VS-EPs	0.000214274	0.000335314	TPs
TP018MC_bin.102	TPs-VS-EPs	1.73E-39	5.54E-38	TPs
TP027MC_bin.12	TPs-VS-EPs	3.21E-13	9.46E-13	TPs
TP025_bin.59	TPs-VS-EPs	4.31E-06	7.63E-06	TPs
TP036_bin.79	TPs-VS-EPs	5.05E-07	9.40E-07	TPs
ERR1135294_bin.5	TPs-VS-EPs	5.03E-05	8.14E-05	EPs
ERR1135181_bin.67	TPs-VS-EPs	0.000161125	0.000253303	EPs
ERR1135254_bin.65	TPs-VS-EPs	9.94E-10	2.28E-09	EPs
ERR1135199_bin.3	TPs-VS-EPs	0.000254119	0.000394654	EPs
ERR1135351_bin.34	TPs-VS-EPs	0.010175304	0.01352748	EPs
ERR1135192_bin.11	TPs-VS-EPs	3.27E-06	5.81E-06	EPs
TP011_bin.67	TPs-VS-EPs	8.44E-21	4.22E-20	TPs
TP040MC_bin.79	TPs-VS-EPs	1.75E-18	7.37E-18	TPs
TP056_bin.5	TPs-VS-EPs	2.09E-29	2.41E-28	TPs
ERR1135196_bin.24	TPs-VS-EPs	7.98E-16	2.78E-15	EPs
TP018MC_bin.156	TPs-VS-EPs	2.09E-17	8.18E-17	TPs
ERR1135281_bin.21	TPs-VS-EPs	4.57E-17	1.77E-16	EPs
ERR1135198_bin.11	TPs-VS-EPs	6.86E-07	1.27E-06	EPs
ERR1135257_bin.51	TPs-VS-EPs	5.03E-05	8.14E-05	EPs
TP052MC_bin.118	TPs-VS-CPs	1.81E-15	1.09E-14	TPs
TP059MC_bin.112	TPs-VS-CPs	8.59E-08	2.13E-07	TPs
TP017MC_bin.126	TPs-VS-CPs	2.32E-23	5.08E-22	TPs
ERR1135399_bin.16	TPs-VS-CPs	2.88E-05	5.29E-05	TPs
ERR1135218_bin.14	TPs-VS-CPs	2.06E-21	2.86E-20	TPs
TP015MC_bin.116	TPs-VS-CPs	9.78E-29	1.21E-26	TPs
TP020_bin.26	TPs-VS-CPs	0.010195216	0.013933461	TPs
ERR1135195_bin.61	TPs-VS-CPs	1.44E-05	2.68E-05	TPs
ERR1135371_bin.2	TPs-VS-CPs	1.49E-21	2.09E-20	TPs
TP007MC_bin.110	TPs-VS-CPs	2.31E-19	2.30E-18	TPs
TP021MC_bin.71	TPs-VS-CPs	1.44E-05	2.68E-05	TPs
TP028_bin.79	TPs-VS-CPs	0.000480894	0.000760877	TPs
TP003MC_bin.114	TPs-VS-CPs	1.22E-08	3.40E-08	TPs
ERR1135209_bin.8	TPs-VS-CPs	0.000371163	0.000594238	TPs
ERR1135398_bin.29	TPs-VS-CPs	1.66E-07	4.02E-07	CPs
TP007MC_bin.132	TPs-VS-CPs	7.59E-31	2.41E-28	TPs
TP030MC_bin.63	TPs-VS-CPs	5.83E-09	1.70E-08	TPs
TP049MC_bin.34	TPs-VS-CPs	2.43E-26	1.09E-24	TPs
ERR1135409_bin.26	TPs-VS-CPs	4.76E-14	2.44E-13	CPs
TP021_bin.55	TPs-VS-CPs	2.70E-06	5.46E-06	TPs
TP008MC_bin.81	TPs-VS-CPs	9.94E-13	4.26E-12	TPs
TP014_bin.70	TPs-VS-CPs	5.63E-06	1.11E-05	TPs
ERR1135214_bin.8	TPs-VS-CPs	0.007849957	0.010902075	CPs
ERR1135318_bin.39	TPs-VS-CPs	4.71E-15	2.73E-14	CPs
TP045_bin.93	TPs-VS-CPs	0.001824866	0.002727178	TPs
ERR1135455_bin.2	TPs-VS-CPs	8.85E-21	1.15E-19	CPs
TP012MC_bin.71	TPs-VS-CPs	5.62E-15	3.19E-14	TPs
ERR1135380_bin.23	TPs-VS-CPs	7.16E-16	4.53E-15	CPs
TP036_bin.66	TPs-VS-CPs	3.53E-12	1.42E-11	TPs

SGBs	Compared groups	p values	Fdr	Enriched Group
TP043_bin.179	TPs-VS-CPs	1.02E-08	2.92E-08	TPs
TP018MC_bin.143	TPs-VS-CPs	1.99E-11	7.26E-11	TPs
TP015MC_bin.37	TPs-VS-CPs	5.08E-09	1.54E-08	TPs
ERR1135426_bin.50	TPs-VS-CPs	1.41E-26	7.38E-25	CPs
TP029_bin.169	TPs-VS-CPs	6.04E-18	4.87E-17	TPs
TP009_bin.4	TPs-VS-CPs	4.34E-05	7.67E-05	TPs
ERR1135197_bin.71	TPs-VS-CPs	0.00492091	0.006957149	CPs
TP020MC_bin.23	TPs-VS-CPs	1.16E-21	1.70E-20	TPs
TP031MC_bin.2	TPs-VS-CPs	9.94E-13	4.26E-12	TPs
ERR1135424_bin.1	TPs-VS-CPs	1.25E-18	1.15E-17	CPs
ERR1135199_bin.61	TPs-VS-CPs	0.007849957	0.010902075	CPs
ERR1135187_bin.54	TPs-VS-CPs	7.11E-22	1.14E-20	TPs
ERR1135206_bin.63	TPs-VS-CPs	0.007849957	0.010902075	CPs
TP005_bin.59	TPs-VS-CPs	5.22E-12	2.09E-11	TPs
TP022_bin.99	TPs-VS-CPs	1.35E-08	3.75E-08	TPs
ERR1135235_bin.13	TPs-VS-CPs	0.008911928	0.012250653	TPs
TP024MC_bin.131	TPs-VS-CPs	1.44E-20	1.66E-19	TPs
TP021_bin.140	TPs-VS-CPs	3.57E-13	1.68E-12	TPs
TP033_bin.92	TPs-VS-CPs	5.29E-11	1.88E-10	TPs
TP002_bin.87	TPs-VS-CPs	5.20E-15	2.97E-14	TPs
TP043_bin.49	TPs-VS-CPs	2.72E-05	5.01E-05	TPs
TP022_bin.117	TPs-VS-CPs	3.99E-25	1.57E-23	TPs
ERR1135357_bin.28	TPs-VS-CPs	8.76E-25	2.95E-23	TPs
TP021_bin.28	TPs-VS-CPs	0.010963267	0.014918269	TPs
ERR1135462_bin.12	TPs-VS-CPs	2.35E-09	7.21E-09	CPs
TP024MC_bin.61	TPs-VS-CPs	0.001802561	0.002727178	CPs
TP028_bin.145	TPs-VS-CPs	9.94E-13	4.26E-12	TPs
TP030MC_bin.48	TPs-VS-CPs	5.16E-17	3.74E-16	CPs
ERR1135319_bin.20	TPs-VS-CPs	0.000457162	0.000724543	CPs
TP020_bin.103	TPs-VS-CPs	1.99E-11	7.26E-11	TPs
TP043_bin.97	TPs-VS-CPs	4.42E-14	2.28E-13	TPs
ERR1135447_bin.34	TPs-VS-CPs	1.51E-11	5.73E-11	CPs
TP001_bin.35	TPs-VS-CPs	1.25E-24	4.06E-23	TPs
TP025_bin.93	TPs-VS-CPs	2.70E-06	5.46E-06	TPs
TP034MC_bin.57	TPs-VS-CPs	0.001824866	0.002727178	TPs
TP045_bin.113	TPs-VS-CPs	7.43E-12	2.91E-11	TPs
TP003_bin.127	TPs-VS-CPs	1.26E-08	3.53E-08	TPs
TP041_bin.33	TPs-VS-CPs	1.44E-08	3.87E-08	TPs
ERR1135190_bin.91	TPs-VS-CPs	1.41E-10	4.76E-10	CPs
TP031MC_bin.26	TPs-VS-CPs	0.002783466	0.004120579	TPs
TP006MC_bin.121	TPs-VS-CPs	0.012737455	0.017134694	TPs
ERR1135208_bin.18	TPs-VS-CPs	1.59E-05	2.95E-05	TPs
ERR1135341_bin.9	TPs-VS-CPs	3.11E-07	7.25E-07	CPs
ERR1135200_bin.85	TPs-VS-CPs	0.010774986	0.014683255	CPs
TP020_bin.23	TPs-VS-CPs	6.04E-18	4.87E-17	TPs
ERR1135198_bin.77	TPs-VS-CPs	1.17E-05	2.22E-05	CPs
ERR1135201_bin.86	TPs-VS-CPs	0.019795772	0.025988028	TPs
TP041MC_bin.125	TPs-VS-CPs	1.44E-08	3.87E-08	TPs
TP043MC_bin.14	TPs-VS-CPs	2.70E-06	5.46E-06	TPs
TP008MC_bin.8	TPs-VS-CPs	1.32E-06	2.76E-06	TPs
TP056_bin.109	TPs-VS-CPs	3.08E-10	1.02E-09	TPs
TP051_bin.130	TPs-VS-CPs	0.000371163	0.000594238	TPs
ERR1135274_bin.61	TPs-VS-CPs	5.58E-06	1.10E-05	TPs
ERR1135219_bin.32	TPs-VS-CPs	0.00492091	0.006957149	CPs
ERR1135410_bin.80	TPs-VS-CPs	3.44E-10	1.14E-09	CPs
TP058_bin.27	TPs-VS-CPs	6.41E-11	2.27E-10	TPs
ERR1135202_bin.56	TPs-VS-CPs	2.70E-06	5.46E-06	TPs
TP033_bin.74	TPs-VS-CPs	8.67E-23	1.60E-21	TPs
TP037MC_bin.57	TPs-VS-CPs	1.26E-14	7.08E-14	TPs
TP044MC_bin.5	TPs-VS-CPs	8.67E-23	1.60E-21	TPs
ERR1135433_bin.33	TPs-VS-CPs	9.10E-13	4.13E-12	CPs
TP043_bin.127	TPs-VS-CPs	3.07E-13	1.47E-12	TPs
ERR1135328_bin.24	TPs-VS-CPs	9.40E-09	2.68E-08	CPs
ERR1135197_bin.53	TPs-VS-CPs	0.019795772	0.025988028	TPs
ERR1135252_bin.62	TPs-VS-CPs	8.22E-23	1.60E-21	CPs
TP049MC_bin.72	TPs-VS-CPs	2.09E-10	7.01E-10	CPs
TP034MC_bin.136	TPs-VS-CPs	5.78E-16	3.73E-15	TPs
TP002_bin.105	TPs-VS-CPs	6.26E-06	1.20E-05	TPs
TP025_bin.142	TPs-VS-CPs	5.29E-11	1.88E-10	TPs
TP045_bin.106	TPs-VS-CPs	4.91E-07	1.08E-06	TPs
TP009_bin.25	TPs-VS-CPs	1.39E-10	4.70E-10	TPs
ERR1135362_bin.43	TPs-VS-CPs	1.35E-17	1.04E-16	CPs
TP044MC_bin.94	TPs-VS-CPs	3.53E-19	3.47E-18	TPs
ERR1135230_bin.14	TPs-VS-CPs	6.26E-06	1.20E-05	TPs
ERR1135459_bin.65	TPs-VS-CPs	0.009949773	0.013637553	CPs
ERR1135408_bin.67	TPs-VS-CPs	2.64E-18	2.28E-17	CPs
TP008MC_bin.72	TPs-VS-CPs	2.32E-23	5.08E-22	TPs
TP027MC_bin.112	TPs-VS-CPs	1.14E-11	4.37E-11	TPs
TP043_bin.15	TPs-VS-CPs	2.08E-10	6.99E-10	TPs
ERR1135328_bin.48	TPs-VS-CPs	0.000171991	0.000287567	CPs
TP043_bin.177	TPs-VS-CPs	5.29E-11	1.88E-10	TPs
ERR1135189_bin.44	TPs-VS-CPs	0.007849957	0.010902075	CPs
ERR1135465_bin.7	TPs-VS-CPs	3.43E-18	2.94E-17	CPs
ERR1135211_bin.21	TPs-VS-CPs	8.12E-06	1.55E-05	CPs
ERR1135322_bin.22	TPs-VS-CPs	3.10E-08	8.08E-08	CPs
TP043_bin.149	TPs-VS-CPs	3.57E-05	6.38E-05	TPs
ERR1135415_bin.65	TPs-VS-CPs	0.000281038	0.000464946	CPs
TP053MC_bin.17	TPs-VS-CPs	7.43E-12	2.91E-11	TPs
TP058_bin.17	TPs-VS-CPs	2.62E-24	7.49E-23	TPs
TP045_bin.91	TPs-VS-CPs	6.08E-17	4.25E-16	TPs
TP012_bin.56	TPs-VS-CPs	1.44E-05	2.68E-05	TPs
ERR1135381_bin.24	TPs-VS-CPs	3.82E-07	8.76E-07	TPs
TP041MC_bin.115	TPs-VS-CPs	1.95E-12	8.13E-12	TPs
ERR1135188_bin.61	TPs-VS-CPs	1.06E-08	3.01E-08	TPs
TP012_bin.66	TPs-VS-CPs	0.004031315	0.005742493	TPs

SGBs	Compared groups	p values	Fdr	Enriched Group
ERR1135389_bin.51	TPs-VS-CPs	0.034733572	0.044261835	CPs
ERR1135337_bin.78	TPs-VS-CPs	0.010535185	0.014377249	TPs
TP012_bin.84	TPs-VS-CPs	9.83E-20	1.07E-18	TPs
ERR1135208_bin.20	TPs-VS-CPs	0.005160376	0.007284782	TPs
ERR1135245_bin.30	TPs-VS-CPs	3.24E-05	5.87E-05	CPs
TP024_bin.106	TPs-VS-CPs	1.41E-19	1.51E-18	TPs
ERR1135204_bin.76	TPs-VS-CPs	0.029474676	0.038074821	CPs
TP024_bin.130	TPs-VS-CPs	9.24E-11	3.24E-10	TPs
TP042_bin.53	TPs-VS-CPs	4.36E-06	8.68E-06	TPs
TP010_bin.119	TPs-VS-CPs	2.03E-23	4.79E-22	CPs
ERR1135188_bin.33	TPs-VS-CPs	7.40E-05	0.000127155	TPs
ERR1135198_bin.54	TPs-VS-CPs	0.003078578	0.004487015	CPs
TP030MC_bin.109	TPs-VS-CPs	1.53E-14	8.36E-14	TPs
ERR1135360_bin.30	TPs-VS-CPs	3.95E-07	9.03E-07	CPs
TP052MC_bin.26	TPs-VS-CPs	0.000296087	0.000485582	CPs
TP052MC_bin.39	TPs-VS-CPs	5.38E-13	2.46E-12	TPs
TP009_bin.28	TPs-VS-CPs	4.42E-14	2.28E-13	TPs
ERR1135421_bin.27	TPs-VS-CPs	0.015597915	0.020863594	CPs
TP036MC_bin.117	TPs-VS-CPs	1.44E-08	3.87E-08	TPs
ERR1135380_bin.51	TPs-VS-CPs	0.012507337	0.01684917	CPs
ERR1135213_bin.84	TPs-VS-CPs	0.007849957	0.010902075	CPs
ERR1135178_bin.36	TPs-VS-CPs	2.22E-12	9.17E-12	TPs
TP008_bin.11	TPs-VS-CPs	9.94E-13	4.26E-12	TPs
TP021_bin.103	TPs-VS-CPs	6.26E-06	1.20E-05	TPs
ERR1135182_bin.35	TPs-VS-CPs	2.30E-06	4.73E-06	TPs
TP058_bin.47	TPs-VS-CPs	5.85E-27	3.25E-25	TPs
TP018_bin.101	TPs-VS-CPs	5.85E-27	3.25E-25	TPs
ERR1135191_bin.13	TPs-VS-CPs	2.70E-17	2.00E-16	CPs
TP037MC_bin.44	TPs-VS-CPs	3.35E-05	6.00E-05	TPs
ERR1135192_bin.68	TPs-VS-CPs	4.33E-07	9.88E-07	TPs
TP030MC_bin.3	TPs-VS-CPs	0.000371163	0.000594238	TPs
TP028_bin.51	TPs-VS-CPs	6.08E-17	4.25E-16	TPs
ERR1135408_bin.15	TPs-VS-CPs	0.003708512	0.005380195	CPs
TP007MC_bin.123	TPs-VS-CPs	2.43E-26	1.09E-24	TPs
TP043_bin.52	TPs-VS-CPs	1.44E-20	1.66E-19	TPs
ERR1135193_bin.58	TPs-VS-CPs	9.10E-13	4.13E-12	CPs
ERR1135220_bin.70	TPs-VS-CPs	0.003078578	0.004487015	CPs
TP018_bin.180	TPs-VS-CPs	2.18E-12	9.04E-12	TPs
ERR1135221_bin.29	TPs-VS-CPs	0.008911928	0.012250653	TPs
TP014MC_bin.61	TPs-VS-CPs	5.78E-16	3.73E-15	TPs
ERR1135276_bin.43	TPs-VS-CPs	0.004031315	0.005742493	TPs
TP014_bin.24	TPs-VS-CPs	4.42E-14	2.28E-13	TPs
ERR1135371_bin.47	TPs-VS-CPs	0.001824866	0.002727178	TPs
TP034MC_bin.88	TPs-VS-CPs	3.54E-08	9.03E-08	TPs
TP053MC_bin.10	TPs-VS-CPs	1.44E-08	3.87E-08	TPs
ERR1135396_bin.46	TPs-VS-CPs	1.27E-23	3.07E-22	TPs
ERR1135464_bin.7	TPs-VS-CPs	0.000108425	0.000185563	CPs
TP008_bin.78	TPs-VS-CPs	0.001621283	0.002481932	TPs
ERR1135471_bin.59	TPs-VS-CPs	0.000457162	0.000724543	CPs
ERR1135391_bin.3	TPs-VS-CPs	3.52E-14	1.88E-13	CPs
TP041MC_bin.117	TPs-VS-CPs	8.59E-08	2.13E-07	TPs
ERR1135407_bin.53	TPs-VS-CPs	3.10E-08	8.08E-08	CPs
TP021_bin.106	TPs-VS-CPs	6.76E-30	1.06E-27	TPs
TP014_bin.83	TPs-VS-CPs	5.84E-22	9.66E-21	TPs
TP059MC_bin.114	TPs-VS-CPs	5.83E-09	1.70E-08	TPs
ERR1135195_bin.18	TPs-VS-CPs	4.58E-19	4.40E-18	TPs
TP049MC_bin.39	TPs-VS-CPs	2.06E-07	4.88E-07	TPs
TP005MC_bin.38	TPs-VS-CPs	1.57E-24	4.63E-23	TPs
ERR1135327_bin.92	TPs-VS-CPs	4.08E-14	2.16E-13	TPs
TP058_bin.7	TPs-VS-CPs	0.000286715	0.000472678	TPs
TP011_bin.135	TPs-VS-CPs	1.99E-11	7.26E-11	TPs
TP001_bin.109	TPs-VS-CPs	1.86E-18	1.63E-17	TPs
ERR1135468_bin.29	TPs-VS-CPs	1.64E-06	3.39E-06	CPs
ERR1135378_bin.20	TPs-VS-CPs	1.50E-17	1.15E-16	CPs
ERR1135449_bin.10	TPs-VS-CPs	5.42E-16	3.60E-15	CPs
TP020_bin.122	TPs-VS-CPs	9.60E-12	3.74E-11	TPs
ERR1135192_bin.10	TPs-VS-CPs	4.31E-05	7.63E-05	TPs
ERR1135191_bin.22	TPs-VS-CPs	4.73E-06	9.37E-06	TPs
TP031MC_bin.68	TPs-VS-CPs	1.99E-11	7.26E-11	TPs
ERR1135202_bin.10	TPs-VS-CPs	0.004031315	0.005742493	TPs
ERR1135209_bin.42	TPs-VS-CPs	0.022339975	0.029017351	CPs
ERR1135192_bin.2	TPs-VS-CPs	0.003078578	0.004487015	CPs
ERR1135419_bin.55	TPs-VS-CPs	1.37E-05	2.58E-05	CPs
TP004MC_bin.13	TPs-VS-CPs	3.21E-28	3.36E-26	TPs
TP041_bin.31	TPs-VS-CPs	1.14E-08	3.20E-08	TPs
TP027MC_bin.89	TPs-VS-CPs	6.08E-17	4.25E-16	TPs
TP002_bin.75	TPs-VS-CPs	5.29E-11	1.88E-10	TPs
TP034MC_bin.147	TPs-VS-CPs	1.75E-15	1.07E-14	TPs
ERR1135400_bin.30	TPs-VS-CPs	7.43E-17	5.15E-16	TPs
ERR1135420_bin.28	TPs-VS-CPs	2.18E-10	7.28E-10	CPs
TP025_bin.115	TPs-VS-CPs	0.011727735	0.015935524	TPs
ERR1135415_bin.35	TPs-VS-CPs	0.012507337	0.01684917	CPs
TP049MC_bin.155	TPs-VS-CPs	1.44E-05	2.68E-05	TPs
ERR1135200_bin.9	TPs-VS-CPs	2.73E-12	1.11E-11	TPs
TP047MC_bin.127	TPs-VS-CPs	2.73E-12	1.11E-11	TPs
ERR1135199_bin.45	TPs-VS-CPs	0.020265433	0.026395447	CPs
ERR1135207_bin.59	TPs-VS-CPs	0.003789557	0.005480908	TPs
TP052MC_bin.128	TPs-VS-CPs	1.44E-20	1.66E-19	TPs
ERR1135272_bin.51	TPs-VS-CPs	5.78E-16	3.73E-15	TPs
TP008MC_bin.100	TPs-VS-CPs	3.90E-08	9.89E-08	CPs
ERR1135184_bin.93	TPs-VS-CPs	0.004031315	0.005742493	TPs
ERR1135472_bin.83	TPs-VS-CPs	1.37E-05	2.58E-05	CPs
ERR1135181_bin.62	TPs-VS-CPs	0.031784016	0.040612909	CPs
TP005_bin.147	TPs-VS-CPs	1.93E-17	1.46E-16	TPs
ERR1135407_bin.25	TPs-VS-CPs	0.019925074	0.025988028	CPs

SGBs	Compared groups	p values	Fdr	Enriched Group
ERR1135201_bin.67	TPs-VS-CPs	1.50E-12	6.32E-12	TPs
TP004MC_bin.86	TPs-VS-CPs	6.08E-17	4.25E-16	TPs
TP049MC_bin.88	TPs-VS-CPs	2.06E-07	4.88E-07	TPs
TP024_bin.91	TPs-VS-CPs	6.30E-07	1.38E-06	TPs
TP021_bin.45	TPs-VS-CPs	0.022252789	0.028943973	TPs
ERR1135183_bin.20	TPs-VS-CPs	0.001705887	0.002602995	CPs
ERR1135209_bin.28	TPs-VS-CPs	1.09E-18	1.03E-17	CPs
TP008MC_bin.33	TPs-VS-CPs	3.57E-13	1.68E-12	TPs
ERR1135256_bin.60	TPs-VS-CPs	0.028030585	0.036259042	CPs
ERR1135289_bin.33	TPs-VS-CPs	0.000457162	0.000724543	CPs
ERR1135383_bin.33	TPs-VS-CPs	0.001920912	0.00285263	CPs
TP049MC_bin.45	TPs-VS-CPs	1.26E-13	6.18E-13	TPs
TP053MC_bin.7	TPs-VS-CPs	3.57E-13	1.68E-12	TPs
TP043MC_bin.32	TPs-VS-CPs	8.59E-08	2.13E-07	TPs
ERR1135256_bin.12	TPs-VS-CPs	0.013752003	0.018420652	CPs
ERR1135264_bin.17	TPs-VS-CPs	1.32E-21	1.89E-20	TPs
ERR1135266_bin.21	TPs-VS-CPs	8.94E-21	1.15E-19	TPs
ERR1135464_bin.8	TPs-VS-CPs	2.21E-08	5.87E-08	CPs
TP008MC_bin.88	TPs-VS-CPs	1.11E-05	2.11E-05	TPs
ERR1135191_bin.2	TPs-VS-CPs	0.031784016	0.040612909	CPs
TP022MC_bin.70	TPs-VS-CPs	3.27E-05	5.87E-05	TPs
TP043_bin.162	TPs-VS-CPs	3.62E-23	7.60E-22	TPs
TP022MC_bin.127	TPs-VS-CPs	0.000166254	0.000280461	TPs
TP053MC_bin.97	TPs-VS-CPs	1.39E-10	4.70E-10	TPs
ERR1135256_bin.53	TPs-VS-CPs	8.92E-09	2.57E-08	CPs
ERR1135286_bin.84	TPs-VS-CPs	6.58E-28	5.64E-26	TPs
TP022MC_bin.16	TPs-VS-CPs	9.19E-10	2.90E-09	TPs
TP045_bin.16	TPs-VS-CPs	1.27E-11	4.86E-11	TPs
ERR1135178_bin.73	TPs-VS-CPs	1.19E-08	3.35E-08	TPs
ERR1135426_bin.24	TPs-VS-CPs	1.07E-07	2.60E-07	CPs
TP024_bin.115	TPs-VS-CPs	2.05E-19	2.06E-18	TPs
ERR1135391_bin.5	TPs-VS-CPs	4.15E-06	8.28E-06	CPs
ERR1135386_bin.37	TPs-VS-CPs	6.33E-18	5.02E-17	CPs
TP042_bin.29	TPs-VS-CPs	9.19E-10	2.90E-09	TPs
ERR1135181_bin.58	TPs-VS-CPs	5.45E-07	1.19E-06	CPs
TP015_bin.102	TPs-VS-CPs	9.29E-22	1.44E-20	TPs
ERR1135385_bin.68	TPs-VS-CPs	2.13E-17	1.59E-16	CPs
ERR1135190_bin.27	TPs-VS-CPs	0.006541023	0.009206246	TPs
ERR1135456_bin.36	TPs-VS-CPs	6.35E-05	0.00011067	CPs
TP018MC_bin.11	TPs-VS-CPs	1.17E-05	2.21E-05	TPs
ERR1135278_bin.69	TPs-VS-CPs	0.019925074	0.025988028	CPs
ERR1135288_bin.11	TPs-VS-CPs	6.89E-09	2.00E-08	TPs
ERR1135335_bin.32	TPs-VS-CPs	9.93E-12	3.85E-11	CPs
ERR1135472_bin.52	TPs-VS-CPs	0.000171991	0.000287567	CPs
TP014_bin.7	TPs-VS-CPs	1.02E-05	1.94E-05	TPs
TP025_bin.34	TPs-VS-CPs	4.01E-15	2.36E-14	TPs
TP029MC_bin.90	TPs-VS-CPs	4.91E-07	1.08E-06	TPs
TP040MC_bin.19	TPs-VS-CPs	1.17E-08	3.31E-08	TPs
TP002_bin.70	TPs-VS-CPs	0.000371163	0.000594238	TPs
ERR1135279_bin.11	TPs-VS-CPs	0.001113674	0.001724457	CPs
TP047MC_bin.79	TPs-VS-CPs	3.48E-06	6.97E-06	TPs
TP036MC_bin.37	TPs-VS-CPs	1.59E-13	7.72E-13	TPs
TP041MC_bin.118	TPs-VS-CPs	0.000719075	0.001135825	CPs
ERR1135402_bin.1	TPs-VS-CPs	2.22E-14	1.21E-13	CPs
TP026_bin.115	TPs-VS-CPs	3.57E-13	1.68E-12	TPs
ERR1135328_bin.73	TPs-VS-CPs	0.000457162	0.000724543	CPs
ERR1135187_bin.47	TPs-VS-CPs	0.01348145	0.018083937	TPs
TP025_bin.117	TPs-VS-CPs	1.16E-06	2.43E-06	TPs
TP034MC_bin.76	TPs-VS-CPs	9.94E-13	4.26E-12	TPs
ERR1135208_bin.40	TPs-VS-CPs	7.40E-05	0.000127155	TPs
TP029_bin.122	TPs-VS-CPs	2.70E-06	5.46E-06	TPs
TP011_bin.84	TPs-VS-CPs	1.99E-11	7.26E-11	TPs
ERR1135196_bin.23	TPs-VS-CPs	1.53E-11	5.78E-11	TPs
ERR1135318_bin.98	TPs-VS-CPs	4.77E-05	8.37E-05	TPs
ERR1135273_bin.20	TPs-VS-CPs	2.14E-07	5.06E-07	CPs
TP058_bin.50	TPs-VS-CPs	1.02E-11	3.93E-11	TPs
TP036MC_bin.15	TPs-VS-CPs	1.57E-24	4.63E-23	TPs
TP025MC_bin.63	TPs-VS-CPs	1.01E-23	2.65E-22	TPs
TP027_bin.9	TPs-VS-CPs	3.90E-05	6.95E-05	TPs
TP029_bin.128	TPs-VS-CPs	4.91E-07	1.08E-06	TPs
ERR1135407_bin.2	TPs-VS-CPs	1.79E-19	1.82E-18	CPs
TP008MC_bin.31	TPs-VS-CPs	2.06E-07	4.88E-07	TPs
TP018MC_bin.157	TPs-VS-CPs	9.94E-13	4.26E-12	TPs
ERR1135178_bin.26	TPs-VS-CPs	1.16E-06	2.43E-06	TPs
TP051_bin.95	TPs-VS-CPs	3.60E-09	1.09E-08	TPs
ERR1135422_bin.61	TPs-VS-CPs	2.14E-15	1.27E-14	CPs
ERR1135192_bin.18	TPs-VS-CPs	5.83E-09	1.70E-08	TPs
TP059MC_bin.87	TPs-VS-CPs	3.59E-10	1.18E-09	TPs
TP024MC_bin.118	TPs-VS-CPs	1.26E-13	6.18E-13	TPs
ERR1135419_bin.50	TPs-VS-CPs	3.27E-09	1.00E-08	CPs
TP012_bin.9	TPs-VS-CPs	0.00082451	0.001283024	TPs
TP045_bin.14	TPs-VS-CPs	1.16E-06	2.43E-06	TPs
ERR1135200_bin.73	TPs-VS-CPs	3.80E-07	8.76E-07	TPs
TP015MC_bin.36	TPs-VS-CPs	4.91E-07	1.08E-06	TPs
TP020MC_bin.105	TPs-VS-CPs	9.19E-10	2.90E-09	TPs
TP002_bin.106	TPs-VS-CPs	0.00082451	0.001283024	TPs
ERR1135199_bin.55	TPs-VS-CPs	5.58E-08	1.40E-07	CPs
TP017MC_bin.2	TPs-VS-CPs	4.91E-07	1.08E-06	TPs
ERR1135307_bin.30	TPs-VS-CPs	0.000281038	0.000464946	CPs
ERR1135318_bin.25	TPs-VS-CPs	1.11E-16	7.55E-16	CPs
ERR1135459_bin.47	TPs-VS-CPs	9.47E-07	2.05E-06	CPs
ERR1135351_bin.65	TPs-VS-CPs	6.04E-18	4.87E-17	TPs
ERR1135267_bin.21	TPs-VS-CPs	3.15E-27	2.28E-25	TPs
TP042_bin.126	TPs-VS-CPs	0.001824866	0.002727178	TPs
TP009_bin.49	TPs-VS-CPs	1.25E-17	9.74E-17	TPs

SGBs	Compared groups	p values	Fdr	Enriched Group
TP014_bin.35	TPs-VS-CPs	0.004031315	0.005742493	TPs
TP053MC_bin.2	TPs-VS-CPs	3.72E-23	7.62E-22	TPs
TP023_bin.58	TPs-VS-CPs	2.57E-08	6.78E-08	TPs
TP023_bin.70	TPs-VS-CPs	9.19E-10	2.90E-09	TPs
TP059MC_bin.116	TPs-VS-CPs	4.60E-05	8.13E-05	TPs
TP037MC_bin.21	TPs-VS-CPs	3.19E-22	5.57E-21	TPs
TP034MC_bin.53	TPs-VS-CPs	3.48E-06	6.97E-06	TPs
TP024_bin.125	TPs-VS-CPs	1.56E-06	3.24E-06	TPs
ERR1135178_bin.34	TPs-VS-CPs	0.001194805	0.001835018	CPs
ERR1135181_bin.68	TPs-VS-CPs	1.08E-07	2.62E-07	CPs
ERR1135322_bin.55	TPs-VS-CPs	3.03E-05	5.56E-05	TPs
TP025_bin.98	TPs-VS-CPs	3.54E-08	9.03E-08	TPs
TP059MC_bin.151	TPs-VS-CPs	1.89E-16	1.26E-15	TPs
TP051_bin.134	TPs-VS-CPs	1.44E-08	3.87E-08	TPs
TP058_bin.96	TPs-VS-CPs	8.86E-20	9.83E-19	TPs
ERR1135198_bin.40	TPs-VS-CPs	3.69E-13	1.72E-12	TPs
ERR1135213_bin.9	TPs-VS-CPs	0.019795772	0.025988028	TPs
TP058_bin.103	TPs-VS-CPs	1.14E-12	4.86E-12	TPs
TP029MC_bin.10	TPs-VS-CPs	1.86E-18	1.63E-17	TPs
TP044MC_bin.96	TPs-VS-CPs	1.99E-11	7.26E-11	TPs
TP043_bin.93	TPs-VS-CPs	6.26E-06	1.20E-05	TPs
ERR1135193_bin.102	TPs-VS-CPs	0.003078578	0.004487015	CPs
ERR1135201_bin.99	TPs-VS-CPs	0.00165626	0.002531366	TPs
TP001_bin.27	TPs-VS-CPs	8.35E-21	1.11E-19	TPs
TP043_bin.51	TPs-VS-CPs	1.16E-06	2.43E-06	TPs
TP002_bin.136	TPs-VS-CPs	0.000338306	0.000550989	TPs
TP033_bin.8	TPs-VS-CPs	3.27E-05	5.87E-05	TPs
ERR1135219_bin.28	TPs-VS-CPs	0.008603379	0.011895875	TPs
TP034MC_bin.156	TPs-VS-CPs	2.70E-06	5.46E-06	TPs
TP027_bin.78	TPs-VS-CPs	1.72E-15	1.07E-14	TPs
ERR1135416_bin.41	TPs-VS-CPs	1.80E-11	6.78E-11	CPs
ERR1135252_bin.29	TPs-VS-CPs	9.96E-08	2.43E-07	CPs
TP015MC_bin.54	TPs-VS-CPs	1.35E-30	2.54E-28	TPs
ERR1135267_bin.18	TPs-VS-CPs	1.11E-05	2.11E-05	TPs
ERR1135195_bin.36	TPs-VS-CPs	0.008449611	0.011700416	TPs
ERR1135200_bin.22	TPs-VS-CPs	2.06E-07	4.88E-07	CPs
ERR1135226_bin.23	TPs-VS-CPs	0.003078578	0.004487015	CPs
ERR1135356_bin.12	TPs-VS-CPs	1.29E-08	3.60E-08	CPs
ERR1135401_bin.33	TPs-VS-CPs	7.81E-25	2.83E-23	CPs
TP041_bin.152	TPs-VS-CPs	3.37E-09	1.03E-08	TPs
ERR1135200_bin.65	TPs-VS-CPs	2.87E-22	5.11E-21	CPs
ERR1135450_bin.9	TPs-VS-CPs	1.06E-23	2.71E-22	CPs
TP011_bin.6	TPs-VS-CPs	1.39E-10	4.70E-10	TPs
TP027_bin.91	TPs-VS-CPs	1.15E-18	1.07E-17	TPs
TP028_bin.30	TPs-VS-CPs	4.42E-14	2.28E-13	TPs
TP012_bin.137	TPs-VS-CPs	1.53E-14	8.36E-14	TPs
TP004MC_bin.63	TPs-VS-CPs	0.004031315	0.005742493	TPs
TP036MC_bin.50	TPs-VS-CPs	1.96E-26	9.74E-25	TPs
TP015MC_bin.77	TPs-VS-CPs	6.26E-06	1.20E-05	TPs
ERR1135185_bin.73	TPs-VS-CPs	0.019925074	0.025988028	CPs
TP043MC_bin.59	TPs-VS-CPs	4.00E-20	4.49E-19	TPs
ERR1135356_bin.72	TPs-VS-CPs	0.008911928	0.012250653	TPs
ERR1135418_bin.17	TPs-VS-CPs	3.26E-17	2.40E-16	CPs
TP015_bin.111	TPs-VS-CPs	1.26E-13	6.18E-13	TPs
TP019MC_bin.9	TPs-VS-CPs	2.43E-21	3.33E-20	TPs
ERR1135183_bin.75	TPs-VS-CPs	3.22E-05	5.87E-05	CPs
TP018MC_bin.185	TPs-VS-CPs	1.44E-08	3.87E-08	TPs
TP021_bin.93	TPs-VS-CPs	7.40E-05	0.000127155	TPs
TP027MC_bin.116	TPs-VS-CPs	3.71E-07	8.58E-07	TPs
TP007MC_bin.50	TPs-VS-CPs	1.23E-06	2.56E-06	TPs
TP056_bin.56	TPs-VS-CPs	5.85E-27	3.25E-25	TPs
TP027_bin.5	TPs-VS-CPs	4.91E-07	1.08E-06	TPs
ERR1135471_bin.11	TPs-VS-CPs	3.10E-08	8.08E-08	CPs
TP021_bin.51	TPs-VS-CPs	9.19E-10	2.90E-09	TPs
TP027MC_bin.147	TPs-VS-CPs	0.000166254	0.000280461	TPs
TP058_bin.70	TPs-VS-CPs	8.38E-22	1.32E-20	TPs
ERR1135449_bin.13	TPs-VS-CPs	3.52E-17	2.57E-16	CPs
TP056_bin.100	TPs-VS-CPs	1.18E-12	5.02E-12	TPs
ERR1135289_bin.44	TPs-VS-CPs	5.83E-09	1.70E-08	TPs
TP052MC_bin.64	TPs-VS-CPs	0.007408597	0.010411784	TPs
ERR1135192_bin.94	TPs-VS-CPs	0.019795772	0.025988028	TPs
TP018MC_bin.69	TPs-VS-CPs	1.10E-08	3.11E-08	TPs
TP036_bin.44	TPs-VS-CPs	8.67E-23	1.60E-21	TPs
TP043MC_bin.127	TPs-VS-CPs	0.000370633	0.000594238	TPs
ERR1135389_bin.43	TPs-VS-CPs	0.000288783	0.000475257	TPs
TP045_bin.86	TPs-VS-CPs	0.029724892	0.038345518	TPs
TP049MC_bin.106	TPs-VS-CPs	0.013354012	0.017938509	TPs
TP059MC_bin.121	TPs-VS-CPs	9.19E-10	2.90E-09	TPs
ERR1135374_bin.10	TPs-VS-CPs	0.00082451	0.001283024	TPs
ERR1135446_bin.34	TPs-VS-CPs	2.24E-22	4.07E-21	CPs
TP031MC_bin.46	TPs-VS-CPs	4.91E-07	1.08E-06	TPs
ERR1135419_bin.72	TPs-VS-CPs	0.003078578	0.004487015	CPs
ERR1135270_bin.77	TPs-VS-CPs	0.009308817	0.012777605	TPs
ERR1135370_bin.25	TPs-VS-CPs	9.19E-10	2.90E-09	TPs
TP015_bin.103	TPs-VS-CPs	2.70E-06	5.46E-06	TPs
TP017MC_bin.98	TPs-VS-CPs	1.26E-13	6.18E-13	TPs
ERR1135342_bin.65	TPs-VS-CPs	0.007849957	0.010902075	CPs
TP011_bin.90	TPs-VS-CPs	6.26E-06	1.20E-05	TPs
TP027MC_bin.2	TPs-VS-CPs	9.08E-07	1.97E-06	TPs
ERR1135232_bin.36	TPs-VS-CPs	0.000171646	0.000287567	TPs
TP006MC_bin.130	TPs-VS-CPs	2.33E-09	7.17E-09	TPs
TP036_bin.108	TPs-VS-CPs	1.16E-06	2.43E-06	TPs
TP014MC_bin.66	TPs-VS-CPs	4.91E-05	8.61E-05	TPs
TP021_bin.56	TPs-VS-CPs	4.24E-06	8.45E-06	TPs
ERR1135439_bin.54	TPs-VS-CPs	5.45E-07	1.19E-06	CPs

SGBs	Compared groups	p values	Fdr	Enriched Group
TP015MC_bin.62	TPs-VS-CPs	2.33E-09	7.17E-09	TPs
TP021_bin.7	TPs-VS-CPs	3.58E-24	9.92E-23	TPs
TP002_bin.148	TPs-VS-CPs	1.73E-06	3.59E-06	TPs
TP012_bin.62	TPs-VS-CPs	0.000371163	0.000594238	TPs
TP034MC_bin.30	TPs-VS-CPs	1.53E-14	8.36E-14	TPs
ERR1135385_bin.11	TPs-VS-CPs	4.70E-05	8.28E-05	CPs
ERR1135225_bin.13	TPs-VS-CPs	7.40E-05	0.000127155	TPs
ERR1135269_bin.35	TPs-VS-CPs	9.96E-08	2.43E-07	CPs
TP045_bin.123	TPs-VS-CPs	6.26E-06	1.20E-05	TPs
ERR1135220_bin.65	TPs-VS-CPs	0.000281038	0.000464946	CPs
TP001_bin.41	TPs-VS-CPs	0.018813536	0.025058142	TPs
TP036_bin.120	TPs-VS-CPs	2.33E-09	7.17E-09	TPs
TP008_bin.61	TPs-VS-CPs	1.44E-08	3.87E-08	TPs
TP021MC_bin.72	TPs-VS-CPs	0.000371163	0.000594238	TPs
TP006MC_bin.52	TPs-VS-CPs	3.27E-05	5.87E-05	TPs
TP044MC_bin.16	TPs-VS-CPs	1.03E-17	8.08E-17	TPs
TP052MC_bin.82	TPs-VS-CPs	1.69E-19	1.75E-18	TPs
ERR1135191_bin.26	TPs-VS-CPs	0.000170851	0.000287567	CPs
TP018_bin.78	TPs-VS-CPs	1.53E-14	8.36E-14	TPs
TP043_bin.40	TPs-VS-CPs	3.81E-07	8.76E-07	TPs
TP036MC_bin.106	TPs-VS-CPs	7.82E-10	2.53E-09	TPs
ERR1135190_bin.104	TPs-VS-CPs	0.001824866	0.002727178	TPs
ERR1135201_bin.17	TPs-VS-CPs	2.42E-12	9.96E-12	CPs
ERR1135187_bin.70	TPs-VS-CPs	2.81E-06	5.66E-06	CPs
ERR1135275_bin.62	TPs-VS-CPs	9.22E-09	2.65E-08	TPs
TP015_bin.34	TPs-VS-CPs	5.36E-23	1.07E-21	TPs
ERR1135224_bin.9	TPs-VS-CPs	9.40E-09	2.68E-08	CPs
ERR1135353_bin.10	TPs-VS-CPs	0.019925074	0.025988028	CPs
TP015_bin.30	TPs-VS-CPs	0.000166254	0.000280461	TPs
TP041_bin.34	TPs-VS-CPs	9.94E-13	4.26E-12	TPs
ERR1135265_bin.18	TPs-VS-CPs	2.52E-07	5.92E-07	TPs
ERR1135217_bin.5	TPs-VS-CPs	4.83E-06	9.53E-06	CPs
TP047MC_bin.22	TPs-VS-CPs	3.27E-05	5.87E-05	TPs
ERR1135201_bin.84	TPs-VS-CPs	0.022857181	0.029607585	TPs
TP051_bin.142	TPs-VS-CPs	1.87E-12	7.82E-12	TPs
TP040MC_bin.137	TPs-VS-CPs	1.24E-13	6.18E-13	TPs
TP053MC_bin.93	TPs-VS-CPs	7.40E-05	0.000127155	TPs
TP015MC_bin.135	TPs-VS-CPs	0.019795772	0.025988028	TPs
TP024MC_bin.53	TPs-VS-CPs	6.04E-18	4.87E-17	TPs
TP014MC_bin.156	TPs-VS-CPs	5.78E-16	3.73E-15	TPs
TP018MC_bin.177	TPs-VS-CPs	5.83E-09	1.70E-08	TPs
TP037MC_bin.37	TPs-VS-CPs	0.019795772	0.025988028	TPs
ERR1135193_bin.27	TPs-VS-CPs	0.030978317	0.039907859	TPs
TP001_bin.90	TPs-VS-CPs	6.31E-15	3.57E-14	TPs
ERR1135464_bin.16	TPs-VS-CPs	0.008142506	0.01129174	TPs
TP043_bin.118	TPs-VS-CPs	4.42E-14	2.28E-13	TPs
TP043MC_bin.22	TPs-VS-CPs	1.44E-08	3.87E-08	TPs
ERR1135196_bin.11	TPs-VS-CPs	0.019925074	0.025988028	CPs
TP026_bin.154	TPs-VS-CPs	6.92E-05	0.000120326	TPs
ERR1135200_bin.12	TPs-VS-CPs	1.44E-08	3.87E-08	TPs
ERR1135196_bin.55	TPs-VS-CPs	0.007849957	0.010902075	CPs
ERR1135205_bin.8	TPs-VS-CPs	2.54E-14	1.37E-13	TPs
TP043MC_bin.109	TPs-VS-CPs	0.000320389	0.000523617	TPs
ERR1135211_bin.33	TPs-VS-CPs	4.04E-05	7.18E-05	TPs
TP002_bin.164	TPs-VS-CPs	5.54E-17	3.99E-16	TPs
TP051_bin.70	TPs-VS-CPs	4.27E-25	1.61E-23	TPs
TP043MC_bin.142	TPs-VS-CPs	1.69E-19	1.75E-18	TPs
TP040MC_bin.98	TPs-VS-CPs	6.08E-24	1.64E-22	TPs
ERR1135197_bin.41	TPs-VS-CPs	4.51E-13	2.10E-12	TPs
ERR1135265_bin.33	TPs-VS-CPs	9.75E-20	1.07E-18	TPs
TP026_bin.65	TPs-VS-CPs	5.83E-09	1.70E-08	TPs
TP045_bin.48	TPs-VS-CPs	1.86E-18	1.63E-17	TPs
ERR1135190_bin.64	TPs-VS-CPs	4.79E-06	9.47E-06	CPs
TP025_bin.6	TPs-VS-CPs	0.004031315	0.005742493	TPs
TP036_bin.122	TPs-VS-CPs	0.003497352	0.005089511	TPs
TP021MC_bin.139	TPs-VS-CPs	0.00082451	0.001283024	TPs
TP027MC_bin.38	TPs-VS-CPs	0.001824866	0.002727178	TPs
TP042_bin.127	TPs-VS-CPs	0.000190744	0.000318356	TPs
TP058_bin.84	TPs-VS-CPs	1.87E-18	1.63E-17	TPs
ERR1135252_bin.17	TPs-VS-CPs	1.35E-18	1.24E-17	CPs
ERR1135330_bin.69	TPs-VS-CPs	0.016159352	0.021583951	TPs
ERR1135202_bin.1	TPs-VS-CPs	0.003078578	0.004487015	CPs
TP030MC_bin.59	TPs-VS-CPs	3.54E-08	9.03E-08	TPs
ERR1135445_bin.40	TPs-VS-CPs	4.16E-10	1.35E-09	CPs
TP025_bin.138	TPs-VS-CPs	3.27E-05	5.87E-05	TPs
TP043_bin.130	TPs-VS-CPs	0.001893114	0.002824694	CPs
TP040MC_bin.9	TPs-VS-CPs	1.55E-05	2.89E-05	TPs
TP012_bin.29	TPs-VS-CPs	0.000903327	0.001403356	TPs
ERR1135202_bin.70	TPs-VS-CPs	0.003078578	0.004487015	CPs
TP045_bin.32	TPs-VS-CPs	1.99E-11	7.26E-11	TPs
TP018_bin.55	TPs-VS-CPs	5.83E-09	1.70E-08	TPs
TP041MC_bin.110	TPs-VS-CPs	1.44E-08	3.87E-08	TPs
ERR1135319_bin.2	TPs-VS-CPs	0.010083388	0.013800631	TPs
ERR1135267_bin.30	TPs-VS-CPs	9.56E-07	2.06E-06	TPs
ERR1135419_bin.22	TPs-VS-CPs	1.48E-09	4.65E-09	CPs
TP015_bin.88	TPs-VS-CPs	4.00E-08	1.01E-07	TPs
TP025_bin.45	TPs-VS-CPs	2.32E-23	5.08E-22	TPs
TP034MC_bin.55	TPs-VS-CPs	1.39E-10	4.70E-10	TPs
TP043_bin.119	TPs-VS-CPs	4.91E-07	1.08E-06	TPs
ERR1135341_bin.51	TPs-VS-CPs	0.031784016	0.040612909	CPs
ERR1135371_bin.30	TPs-VS-CPs	0.000405282	0.000647765	CPs
TP051_bin.6	TPs-VS-CPs	4.91E-07	1.08E-06	TPs
ERR1135277_bin.10	TPs-VS-CPs	0.000281038	0.000464946	CPs
ERR1135333_bin.56	TPs-VS-CPs	1.12E-06	2.41E-06	CPs
ERR1135453_bin.4	TPs-VS-CPs	3.13E-23	6.71E-22	CPs

SGBs	Compared groups	p values	Fdr	Enriched Group
ERR1135181_bin.32	TPs-VS-CPs	5.45E-07	1.19E-06	CPs
ERR1135263_bin.3	TPs-VS-CPs	0.019925074	0.025988028	TPs
TP025_bin.136	TPs-VS-CPs	3.77E-08	9.59E-08	CPs
ERR1135389_bin.61	TPs-VS-CPs	1.13E-10	3.92E-10	CPs
TP001_bin.117	TPs-VS-CPs	8.59E-08	2.13E-07	TPs
ERR1135453_bin.41	TPs-VS-CPs	0.000104758	0.000179613	CPs
TP020MC_bin.75	TPs-VS-CPs	1.75E-15	1.07E-14	TPs
TP034MC_bin.157	TPs-VS-CPs	1.16E-06	2.43E-06	TPs
TP027_bin.157	TPs-VS-CPs	0.001824866	0.002727178	TPs
TP012_bin.39	TPs-VS-CPs	7.96E-14	4.01E-13	TPs
ERR1135294_bin.23	TPs-VS-CPs	2.34E-08	6.19E-08	TPs
ERR1135304_bin.69	TPs-VS-CPs	0.031784016	0.040612909	CPs
ERR1135302_bin.36	TPs-VS-CPs	0.002012131	0.002983396	TPs
ERR1135458_bin.26	TPs-VS-CPs	1.84E-20	2.09E-19	CPs
ERR1135403_bin.7	TPs-VS-CPs	0.012430291	0.016841616	TPs
TP002_bin.8	TPs-VS-CPs	4.69E-13	2.17E-12	TPs
TP049MC_bin.131	TPs-VS-CPs	0.003622439	0.005263421	CPs
ERR1135371_bin.19	TPs-VS-CPs	2.06E-07	4.88E-07	TPs
ERR1135383_bin.11	TPs-VS-CPs	4.74E-05	8.34E-05	TPs
ERR1135260_bin.46	TPs-VS-CPs	0.000457162	0.000724543	CPs
TP021MC_bin.45	TPs-VS-CPs	1.39E-15	8.75E-15	TPs
TP026_bin.108	TPs-VS-CPs	1.16E-06	2.43E-06	TPs
TP051_bin.64	TPs-VS-CPs	2.73E-12	1.11E-11	TPs
ERR1135371_bin.25	TPs-VS-CPs	0.003738875	0.005415912	TPs
ERR1135205_bin.52	TPs-VS-CPs	3.37E-22	5.78E-21	TPs
TP049MC_bin.7	TPs-VS-CPs	0.000338306	0.000550989	TPs
TP018MC_bin.74	TPs-VS-CPs	0.001732926	0.002639983	TPs
TP047MC_bin.9	TPs-VS-CPs	6.44E-22	1.05E-20	TPs
TP059MC_bin.84	TPs-VS-CPs	1.16E-06	2.43E-06	TPs
TP041MC_bin.31	TPs-VS-CPs	3.27E-05	5.87E-05	TPs
TP029_bin.154	TPs-VS-CPs	9.19E-10	2.90E-09	TPs
ERR1135325_bin.58	TPs-VS-CPs	4.47E-07	1.02E-06	TPs
TP049MC_bin.125	TPs-VS-CPs	3.27E-05	5.87E-05	TPs
TP051_bin.108	TPs-VS-CPs	7.43E-12	2.91E-11	TPs
TP022MC_bin.63	TPs-VS-CPs	1.16E-21	1.70E-20	TPs
TP027_bin.153	TPs-VS-CPs	4.12E-21	5.54E-20	TPs
TP004MC_bin.66	TPs-VS-CPs	6.26E-06	1.20E-05	TPs
TP025_bin.106	TPs-VS-CPs	1.39E-10	4.70E-10	TPs
TP043_bin.98	TPs-VS-CPs	4.69E-19	4.46E-18	TPs
TP020MC_bin.144	TPs-VS-CPs	0.005333237	0.007517552	TPs
TP052MC_bin.61	TPs-VS-CPs	2.84E-07	6.64E-07	TPs
TP004MC_bin.67	TPs-VS-CPs	2.70E-06	5.46E-06	TPs
ERR1135397_bin.48	TPs-VS-CPs	1.55E-05	2.88E-05	CPs
TP041_bin.136	TPs-VS-CPs	8.22E-25	2.87E-23	TPs
TP006MC_bin.14	TPs-VS-CPs	1.94E-08	5.19E-08	TPs
TP059MC_bin.127	TPs-VS-CPs	1.16E-21	1.70E-20	TPs
TP019MC_bin.30	TPs-VS-CPs	6.04E-18	4.87E-17	TPs
TP026_bin.87	TPs-VS-CPs	7.43E-12	2.91E-11	TPs
TP033_bin.130	TPs-VS-CPs	2.58E-12	1.06E-11	TPs
ERR1135410_bin.13	TPs-VS-CPs	0.000285114	0.000470863	TPs
TP018_bin.26	TPs-VS-CPs	9.94E-13	4.26E-12	TPs
TP043_bin.18	TPs-VS-CPs	0.000166254	0.000280461	TPs
ERR1135446_bin.30	TPs-VS-CPs	7.14E-16	4.53E-15	CPs
TP020MC_bin.157	TPs-VS-CPs	1.26E-13	6.18E-13	TPs
TP053MC_bin.38	TPs-VS-CPs	0.000166254	0.000280461	TPs
ERR1135202_bin.31	TPs-VS-CPs	1.86E-15	1.11E-14	CPs
ERR1135183_bin.4	TPs-VS-CPs	3.30E-07	7.66E-07	TPs
ERR1135385_bin.10	TPs-VS-CPs	0.001194805	0.001835018	CPs
ERR1135259_bin.56	TPs-VS-CPs	1.30E-06	2.71E-06	CPs
ERR1135441_bin.78	TPs-VS-CPs	1.03E-10	3.60E-10	CPs
TP029MC_bin.20	TPs-VS-CPs	0.000294808	0.000484327	TPs
ERR1135389_bin.14	TPs-VS-CPs	0.008655317	0.011950166	CPs
ERR1135262_bin.62	TPs-VS-CPs	2.06E-07	4.88E-07	TPs
ERR1135458_bin.47	TPs-VS-CPs	6.45E-07	1.41E-06	TPs
TP012_bin.3	TPs-VS-CPs	6.35E-09	1.85E-08	TPs
ERR1135319_bin.63	TPs-VS-CPs	1.53E-18	1.39E-17	CPs
ERR1135360_bin.47	TPs-VS-CPs	1.66E-16	1.12E-15	TPs
TP011_bin.85	TPs-VS-CPs	6.64E-14	3.37E-13	TPs
ERR1135262_bin.43	TPs-VS-CPs	0.000371163	0.000594238	TPs
ERR1135188_bin.29	TPs-VS-CPs	1.81E-15	1.09E-14	CPs
ERR1135462_bin.8	TPs-VS-CPs	0.004735078	0.006714555	CPs
TP025_bin.18	TPs-VS-CPs	1.22E-30	2.54E-28	TPs
TP025MC_bin.148	TPs-VS-CPs	0.00082451	0.001283024	TPs
TP027_bin.147	TPs-VS-CPs	5.10E-09	1.54E-08	TPs
TP001_bin.18	TPs-VS-CPs	0.000166254	0.000280461	TPs
TP001_bin.56	TPs-VS-CPs	2.91E-27	2.28E-25	TPs
ERR1135216_bin.5	TPs-VS-CPs	2.24E-06	4.61E-06	CPs
ERR1135276_bin.56	TPs-VS-CPs	2.30E-05	4.24E-05	CPs
TP033_bin.32	TPs-VS-CPs	2.22E-14	1.21E-13	TPs
TP041_bin.163	TPs-VS-CPs	3.54E-08	9.03E-08	TPs
ERR1135347_bin.12	TPs-VS-CPs	0.003078578	0.004487015	CPs
TP008MC_bin.139	TPs-VS-CPs	0.000166254	0.000280461	TPs
ERR1135401_bin.43	TPs-VS-CPs	1.05E-06	2.26E-06	CPs
TP030MC_bin.27	TPs-VS-CPs	1.39E-10	4.70E-10	TPs
ERR1135204_bin.18	TPs-VS-CPs	3.61E-13	1.69E-12	TPs
ERR1135189_bin.8	TPs-VS-CPs	0.000171991	0.000287567	CPs
TP034MC_bin.40	TPs-VS-CPs	1.68E-13	8.11E-13	TPs
TP012MC_bin.85	TPs-VS-CPs	3.27E-05	5.87E-05	TPs
TP012MC_bin.25	TPs-VS-CPs	0.000166254	0.000280461	TPs
TP024_bin.56	TPs-VS-CPs	1.93E-17	1.46E-16	TPs
ERR1135209_bin.29	TPs-VS-CPs	1.99E-11	7.26E-11	TPs
ERR1135468_bin.57	TPs-VS-CPs	9.90E-11	3.46E-10	CPs
TP002_bin.84	TPs-VS-CPs	0.019795772	0.025988028	TPs
TP019MC_bin.95	TPs-VS-CPs	4.91E-07	1.08E-06	TPs
ERR1135191_bin.69	TPs-VS-CPs	0.007849957	0.010902075	CPs

SGBs	Compared groups	p values	Fdr	Enriched Group
ERR1135383_bin.49	TPs-VS-CPs	3.10E-08	8.08E-08	CPs
ERR1135387_bin.51	TPs-VS-CPs	4.71E-15	2.73E-14	CPs
ERR1135466_bin.58	TPs-VS-CPs	3.39E-08	8.82E-08	CPs
ERR1135426_bin.43	TPs-VS-CPs	5.53E-05	9.68E-05	CPs
TP031MC_bin.51	TPs-VS-CPs	2.77E-14	1.48E-13	TPs
TP043_bin.129	TPs-VS-CPs	3.27E-05	5.87E-05	TPs
TP056_bin.102	TPs-VS-CPs	0.001427385	0.002188657	TPs
ERR1135201_bin.79	TPs-VS-CPs	0.004065763	0.005782827	TPs
TP014MC_bin.90	TPs-VS-CPs	1.99E-11	7.26E-11	TPs
TP011_bin.101	TPs-VS-CPs	0.011818532	0.016035792	TPs
TP053MC_bin.136	TPs-VS-CPs	2.90E-31	2.41E-28	TPs
ERR1135191_bin.33	TPs-VS-CPs	7.49E-09	2.17E-08	TPs
TP036MC_bin.76	TPs-VS-CPs	6.26E-06	1.20E-05	TPs
ERR1135466_bin.30	TPs-VS-CPs	0.003078578	0.004487015	CPs
ERR1135406_bin.22	TPs-VS-CPs	5.49E-27	3.25E-25	CPs
TP045_bin.120	TPs-VS-CPs	8.59E-08	2.13E-07	TPs
ERR1135470_bin.34	TPs-VS-CPs	1.01E-20	1.27E-19	CPs
TP005_bin.3	TPs-VS-CPs	5.83E-09	1.70E-08	TPs
ERR1135188_bin.70	TPs-VS-CPs	3.87E-05	6.89E-05	TPs
ERR1135213_bin.46	TPs-VS-CPs	2.14E-08	5.71E-08	TPs
ERR1135291_bin.83	TPs-VS-CPs	5.82E-11	2.06E-10	CPs
ERR1135464_bin.77	TPs-VS-CPs	0.001920912	0.00285263	CPs
TP010_bin.76	TPs-VS-CPs	4.91E-07	1.08E-06	TPs
ERR1135453_bin.49	TPs-VS-CPs	3.68E-12	1.48E-11	CPs
TP021MC_bin.53	TPs-VS-CPs	3.54E-08	9.03E-08	TPs
TP040MC_bin.156	TPs-VS-CPs	1.44E-08	3.87E-08	TPs
TP024_bin.19	TPs-VS-CPs	0.004031315	0.005742493	TPs
ERR1135200_bin.105	TPs-VS-CPs	1.44E-07	3.49E-07	TPs
ERR1135196_bin.29	TPs-VS-CPs	0.00031442	0.000514754	TPs
TP014_bin.67	TPs-VS-CPs	6.26E-06	1.20E-05	TPs
ERR1135208_bin.43	TPs-VS-CPs	1.81E-09	5.66E-09	TPs
ERR1135379_bin.27	TPs-VS-CPs	1.27E-21	1.84E-20	CPs
ERR1135252_bin.35	TPs-VS-CPs	1.79E-19	1.82E-18	CPs
TP045_bin.129	TPs-VS-CPs	2.06E-07	4.88E-07	TPs
TP043_bin.81	TPs-VS-CPs	0.00082451	0.001283024	TPs
ERR1135378_bin.53	TPs-VS-CPs	2.20E-10	7.32E-10	CPs
ERR1135447_bin.18	TPs-VS-CPs	6.33E-18	5.02E-17	CPs
TP002_bin.4	TPs-VS-CPs	9.78E-08	2.41E-07	TPs
TP051_bin.119	TPs-VS-CPs	3.59E-10	1.18E-09	TPs
ERR1135458_bin.60	TPs-VS-CPs	0.004456028	0.006328365	CPs
ERR1135426_bin.54	TPs-VS-CPs	7.40E-26	3.17E-24	CPs
TP006MC_bin.74	TPs-VS-CPs	9.94E-13	4.26E-12	TPs
TP047MC_bin.136	TPs-VS-CPs	5.83E-09	1.70E-08	TPs
TP018MC_bin.51	TPs-VS-CPs	7.40E-05	0.000127155	TPs
ERR1135472_bin.8	TPs-VS-CPs	1.15E-25	4.72E-24	CPs
ERR1135194_bin.3	TPs-VS-CPs	8.65E-08	2.13E-07	CPs
TP051_bin.24	TPs-VS-CPs	4.91E-07	1.08E-06	TPs
TP052MC_bin.93	TPs-VS-CPs	0.000371163	0.000594238	TPs
ERR1135212_bin.6	TPs-VS-CPs	5.29E-11	1.88E-10	TPs
ERR1135220_bin.68	TPs-VS-CPs	0.001824866	0.002727178	TPs
ERR1135439_bin.5	TPs-VS-CPs	1.10E-20	1.34E-19	CPs
TP018_bin.105	TPs-VS-CPs	8.59E-08	2.13E-07	TPs
TP020_bin.5	TPs-VS-CPs	1.90E-07	4.59E-07	TPs
ERR1135190_bin.88	TPs-VS-CPs	2.75E-07	6.46E-07	TPs
ERR1135452_bin.26	TPs-VS-CPs	3.72E-22	6.27E-21	CPs
ERR1135467_bin.21	TPs-VS-CPs	5.35E-14	2.73E-13	CPs
ERR1135258_bin.21	TPs-VS-CPs	9.96E-08	2.43E-07	CPs
ERR1135283_bin.47	TPs-VS-CPs	1.10E-20	1.34E-19	CPs
TP034MC_bin.112	TPs-VS-CPs	8.51E-17	5.86E-16	TPs
TP009_bin.66	TPs-VS-CPs	1.02E-16	6.95E-16	TPs
ERR1135208_bin.72	TPs-VS-CPs	0.004031315	0.005742493	TPs
ERR1135191_bin.23	TPs-VS-CPs	5.83E-09	1.70E-08	TPs
ERR1135194_bin.68	TPs-VS-CPs	0.000740518	0.00116774	CPs
TP058_bin.85	TPs-VS-CPs	0.022701313	0.029446132	TPs
TP009_bin.41	TPs-VS-CPs	5.29E-11	1.88E-10	TPs
TP025_bin.139	TPs-VS-CPs	4.91E-07	1.08E-06	TPs
TP002_bin.67	TPs-VS-CPs	1.16E-06	2.43E-06	TPs
TP018MC_bin.115	TPs-VS-CPs	4.17E-08	1.05E-07	TPs
TP059MC_bin.25	TPs-VS-CPs	3.27E-05	5.87E-05	TPs
TP044MC_bin.62	TPs-VS-CPs	1.02E-11	3.93E-11	TPs
TP015_bin.39	TPs-VS-CPs	9.94E-13	4.26E-12	TPs
TP052MC_bin.34	TPs-VS-CPs	5.70E-12	2.27E-11	TPs
TP040MC_bin.111	TPs-VS-CPs	5.83E-09	1.70E-08	TPs
ERR1135378_bin.9	TPs-VS-CPs	4.71E-15	2.73E-14	CPs
ERR1135201_bin.12	TPs-VS-CPs	0.000260132	0.000433399	CPs
TP056_bin.37	TPs-VS-CPs	0.018007025	0.024017857	TPs
TP043_bin.169	TPs-VS-CPs	7.66E-31	2.41E-28	TPs
ERR1135205_bin.45	TPs-VS-CPs	1.96E-15	1.17E-14	TPs
TP007MC_bin.20	TPs-VS-CPs	3.01E-08	7.92E-08	TPs
ERR1135275_bin.52	TPs-VS-CPs	1.02E-28	1.21E-26	TPs
TP010_bin.100	TPs-VS-CPs	1.44E-20	1.66E-19	TPs
TP014_bin.39	TPs-VS-CPs	5.82E-06	1.14E-05	TPs
TP045_bin.1	TPs-VS-CPs	3.59E-10	1.18E-09	TPs
TP024MC_bin.130	TPs-VS-CPs	5.83E-18	4.87E-17	TPs
ERR1135210_bin.33	TPs-VS-CPs	0.019925074	0.025988028	CPs
ERR1135286_bin.7	TPs-VS-CPs	3.96E-19	3.85E-18	CPs
TP011_bin.97	TPs-VS-CPs	1.16E-06	2.43E-06	TPs
TP019MC_bin.103	TPs-VS-CPs	0.004031315	0.005742493	TPs
TP027MC_bin.27	TPs-VS-CPs	9.94E-13	4.26E-12	TPs
TP021_bin.115	TPs-VS-CPs	7.40E-05	0.000127155	TPs
ERR1135203_bin.24	TPs-VS-CPs	0.001920912	0.00285263	CPs
ERR1135213_bin.14	TPs-VS-CPs	9.47E-07	2.05E-06	CPs
ERR1135460_bin.61	TPs-VS-CPs	1.31E-24	4.11E-23	CPs
TP021_bin.156	TPs-VS-CPs	1.80E-05	3.32E-05	TPs
TP009_bin.103	TPs-VS-CPs	1.45E-12	6.12E-12	TPs

SGBs	Compared groups	p values	Fdr	Enriched Group
ERR1135441_bin.14	TPs-VS-CPs	0.012507337	0.01684917	CPs
TP023_bin.85	TPs-VS-CPs	5.49E-28	5.18E-26	TPs
TP015MC_bin.75	TPs-VS-CPs	0.019403999	0.025808139	TPs
TP036_bin.7	TPs-VS-CPs	5.25E-13	2.41E-12	TPs
ERR1135419_bin.8	TPs-VS-CPs	0.001194805	0.001835018	CPs
TP058_bin.105	TPs-VS-CPs	3.54E-08	9.03E-08	TPs
TP036_bin.80	TPs-VS-CPs	0.001824866	0.002727178	TPs
ERR1135359_bin.27	TPs-VS-CPs	0.001194805	0.001835018	CPs
TP021_bin.147	TPs-VS-CPs	2.70E-06	5.46E-06	TPs
ERR1135215_bin.21	TPs-VS-CPs	0.031784016	0.040612909	CPs
ERR1135352_bin.30	TPs-VS-CPs	1.23E-23	3.06E-22	CPs
TP037MC_bin.162	TPs-VS-CPs	1.44E-05	2.68E-05	TPs
TP004MC_bin.41	TPs-VS-CPs	3.54E-08	9.03E-08	TPs
TP007MC_bin.55	TPs-VS-CPs	0.00082451	0.001283024	TPs
TP018_bin.85	TPs-VS-CPs	0.000371163	0.000594238	TPs
TP045_bin.23	TPs-VS-CPs	2.06E-07	4.88E-07	TPs
TP044MC_bin.58	TPs-VS-CPs	1.69E-19	1.75E-18	TPs
TP003MC_bin.13	TPs-VS-CPs	6.11E-16	3.92E-15	TPs
ERR1135188_bin.12	TPs-VS-CPs	0.001824866	0.002727178	TPs
ERR1135261_bin.34	TPs-VS-CPs	1.44E-08	3.87E-08	TPs
ERR1135464_bin.47	TPs-VS-CPs	0.001194805	0.001835018	CPs
ERR1135311_bin.79	TPs-VS-CPs	4.16E-10	1.35E-09	CPs
TP031MC_bin.105	TPs-VS-CPs	6.04E-18	4.87E-17	TPs
TP034MC_bin.67	TPs-VS-CPs	1.16E-06	2.43E-06	TPs
TP027_bin.100	TPs-VS-CPs	3.69E-07	8.54E-07	TPs
TP040MC_bin.40	TPs-VS-CPs	1.75E-15	1.07E-14	TPs
ERR1135465_bin.9	TPs-VS-CPs	9.97E-21	1.27E-19	CPs
ERR1135260_bin.40	TPs-VS-CPs	1.17E-05	2.22E-05	TPs
ERR1135199_bin.71	TPs-VS-CPs	0.00102415	0.001588443	CPs
ERR1135458_bin.70	TPs-VS-CPs	0.033594187	0.042867819	CPs
TP018MC_bin.102	TPs-VS-CPs	1.44E-20	1.66E-19	TPs
TP027MC_bin.12	TPs-VS-CPs	4.80E-07	1.08E-06	TPs
TP025_bin.59	TPs-VS-CPs	4.50E-06	8.94E-06	TPs
TP036_bin.79	TPs-VS-CPs	0.00082451	0.001283024	TPs
ERR1135181_bin.67	TPs-VS-CPs	0.012507337	0.01684917	CPs
ERR1135254_bin.65	TPs-VS-CPs	0.031784016	0.040612909	CPs
ERR1135199_bin.3	TPs-VS-CPs	8.12E-06	1.55E-05	CPs
ERR1135417_bin.79	TPs-VS-CPs	6.35E-05	0.00011067	CPs
ERR1135192_bin.11	TPs-VS-CPs	3.90E-06	7.81E-06	CPs
TP011_bin.67	TPs-VS-CPs	9.94E-13	4.26E-12	TPs
TP040MC_bin.79	TPs-VS-CPs	2.33E-09	7.17E-09	TPs
TP056_bin.5	TPs-VS-CPs	5.20E-15	2.97E-14	TPs
ERR1135196_bin.24	TPs-VS-CPs	0.001824866	0.002727178	TPs
TP018MC_bin.156	TPs-VS-CPs	2.33E-09	7.17E-09	TPs
ERR1135281_bin.21	TPs-VS-CPs	3.11E-07	7.25E-07	CPs
ERR1135198_bin.11	TPs-VS-CPs	7.96E-11	2.80E-10	TPs
TP052MC_bin.118	EPs-VS-CPs	4.22E-07	1.17E-06	CPs
ERR1135277_bin.11	EPs-VS-CPs	0.005187304	0.009264525	EPs
ERR1135399_bin.16	EPs-VS-CPs	2.00E-39	9.38E-38	CPs
ERR1135218_bin.14	EPs-VS-CPs	0.001671929	0.003259897	EPs
TP052MC_bin.32	EPs-VS-CPs	3.16E-40	1.66E-38	CPs
TP015MC_bin.116	EPs-VS-CPs	1.71E-07	4.95E-07	EPs
TP020_bin.26	EPs-VS-CPs	2.00E-12	9.23E-12	EPs
ERR1135195_bin.61	EPs-VS-CPs	4.42E-11	1.80E-10	EPs
ERR1135216_bin.4	EPs-VS-CPs	0.00175246	0.003380015	EPs
ERR1135371_bin.2	EPs-VS-CPs	0.008382131	0.014506284	EPs
TP007MC_bin.110	EPs-VS-CPs	3.72E-10	1.41E-09	CPs
ERR1135315_bin.55	EPs-VS-CPs	0.000190723	0.000416419	EPs
TP028_bin.79	EPs-VS-CPs	2.30E-18	1.59E-17	CPs
ERR1135209_bin.8	EPs-VS-CPs	0.019962915	0.03206274	EPs
ERR1135325_bin.13	EPs-VS-CPs	0.007967385	0.013869152	EPs
ERR1135293_bin.50	EPs-VS-CPs	3.61E-26	4.81E-25	EPs
ERR1135398_bin.29	EPs-VS-CPs	9.33E-08	2.80E-07	CPs
ERR1135205_bin.17	EPs-VS-CPs	5.36E-19	3.96E-18	EPs
TP007MC_bin.132	EPs-VS-CPs	2.02E-05	4.87E-05	EPs
TP021_bin.55	EPs-VS-CPs	0.00175246	0.003380015	EPs
ERR1135214_bin.8	EPs-VS-CPs	0.000160016	0.000352825	EPs
ERR1135308_bin.44	EPs-VS-CPs	1.43E-14	7.58E-14	EPs
ERR1135318_bin.39	EPs-VS-CPs	1.14E-19	8.99E-19	CPs
ERR1135274_bin.6	EPs-VS-CPs	6.54E-12	2.86E-11	EPs
ERR1135192_bin.7	EPs-VS-CPs	0.000262825	0.000562837	EPs
ERR1135455_bin.2	EPs-VS-CPs	4.74E-52	1.41E-49	CPs
ERR1135380_bin.23	EPs-VS-CPs	4.81E-46	5.37E-44	CPs
TP043_bin.179	EPs-VS-CPs	0.005914652	0.010521483	EPs
ERR1135426_bin.50	EPs-VS-CPs	1.56E-44	1.27E-42	CPs
TP008_bin.114	EPs-VS-CPs	0.03214519	0.049492507	CPs
TP009_bin.4	EPs-VS-CPs	2.22E-06	5.87E-06	CPs
ERR1135311_bin.94	EPs-VS-CPs	0.001333339	0.00261686	EPs
ERR1135424_bin.1	EPs-VS-CPs	1.11E-47	1.65E-45	CPs
ERR1135199_bin.61	EPs-VS-CPs	1.46E-19	1.14E-18	EPs
ERR1135187_bin.54	EPs-VS-CPs	0.002132686	0.004069419	EPs
ERR1135206_bin.63	EPs-VS-CPs	0.000207825	0.000452654	EPs
TP005_bin.59	EPs-VS-CPs	0.006500678	0.01147254	CPs
ERR1135235_bin.13	EPs-VS-CPs	0.000769496	0.001544179	EPs
TP021_bin.140	EPs-VS-CPs	2.32E-06	6.08E-06	EPs
TP043_bin.49	EPs-VS-CPs	0.010023018	0.016983976	EPs
TP022_bin.117	EPs-VS-CPs	0.011644241	0.019436089	EPs
ERR1135357_bin.28	EPs-VS-CPs	0.00060447	0.001238055	EPs
TP021_bin.28	EPs-VS-CPs	7.01E-21	6.26E-20	CPs
ERR1135215_bin.23	EPs-VS-CPs	5.03E-07	1.39E-06	EPs
ERR1135462_bin.12	EPs-VS-CPs	2.85E-16	1.65E-15	CPs
TP024MC_bin.61	EPs-VS-CPs	9.58E-18	6.43E-17	CPs
TP030MC_bin.48	EPs-VS-CPs	1.08E-44	9.64E-43	CPs
ERR1135284_bin.38	EPs-VS-CPs	0.019962915	0.03206274	EPs
ERR1135319_bin.20	EPs-VS-CPs	5.34E-07	1.46E-06	CPs

SGBs	Compared groups	p values	Fdr	Enriched Group
TP020_bin.103	EPs-VS-CPs	0.00010815	0.000241445	EPs
TP043_bin.97	EPs-VS-CPs	0.001013453	0.002011141	EPs
ERR1135447_bin.34	EPs-VS-CPs	1.71E-16	1.02E-15	CPs
TP001_bin.35	EPs-VS-CPs	1.97E-29	3.92E-28	CPs
ERR1135346_bin.81	EPs-VS-CPs	6.57E-16	3.67E-15	EPs
ERR1135190_bin.91	EPs-VS-CPs	5.74E-11	2.32E-10	CPs
TP006MC_bin.121	EPs-VS-CPs	0.000158353	0.000350024	EPs
ERR1135341_bin.9	EPs-VS-CPs	1.60E-27	2.65E-26	EPs
ERR1135198_bin.77	EPs-VS-CPs	7.31E-06	1.85E-05	CPs
ERR1135245_bin.26	EPs-VS-CPs	3.12E-27	4.81E-26	EPs
ERR1135201_bin.86	EPs-VS-CPs	1.78E-13	8.64E-13	EPs
ERR1135187_bin.18	EPs-VS-CPs	0.00351553	0.006459605	EPs
TP029MC_bin.9	EPs-VS-CPs	0.005984777	0.010625061	CPs
ERR1135187_bin.84	EPs-VS-CPs	4.57E-05	0.00010594	EPs
ERR1135254_bin.35	EPs-VS-CPs	8.65E-09	2.91E-08	EPs
ERR1135216_bin.19	EPs-VS-CPs	1.06E-16	6.44E-16	EPs
ERR1135221_bin.24	EPs-VS-CPs	0.00175246	0.003380015	EPs
TP056_bin.109	EPs-VS-CPs	0.008515306	0.014595333	CPs
ERR1135274_bin.61	EPs-VS-CPs	1.43E-13	7.04E-13	EPs
ERR1135219_bin.32	EPs-VS-CPs	1.06E-07	3.18E-07	EPs
TP004MC_bin.38	EPs-VS-CPs	0.000696846	0.001411075	EPs
ERR1135410_bin.80	EPs-VS-CPs	1.43E-13	7.04E-13	CPs
ERR1135225_bin.24	EPs-VS-CPs	0.005187304	0.009264525	EPs
TP058_bin.27	EPs-VS-CPs	0.015640888	0.02558116	CPs
TP033_bin.74	EPs-VS-CPs	5.79E-10	2.15E-09	EPs
ERR1135433_bin.33	EPs-VS-CPs	6.14E-31	1.37E-29	CPs
ERR1135328_bin.24	EPs-VS-CPs	3.55E-17	2.23E-16	EPs
ERR1135197_bin.53	EPs-VS-CPs	1.67E-27	2.71E-26	EPs
ERR1135252_bin.62	EPs-VS-CPs	6.33E-30	1.28E-28	CPs
TP049MC_bin.72	EPs-VS-CPs	1.75E-27	2.78E-26	CPs
ERR1135316_bin.81	EPs-VS-CPs	6.36E-29	1.23E-27	EPs
ERR1135200_bin.44	EPs-VS-CPs	4.28E-38	1.74E-36	EPs
ERR1135276_bin.96	EPs-VS-CPs	8.13E-05	0.000182792	EPs
ERR1135230_bin.14	EPs-VS-CPs	5.10E-14	2.57E-13	EPs
ERR1135217_bin.57	EPs-VS-CPs	1.39E-22	1.45E-21	EPs
ERR1135264_bin.32	EPs-VS-CPs	2.66E-16	1.55E-15	EPs
ERR1135408_bin.67	EPs-VS-CPs	1.59E-34	4.60E-33	CPs
TP052MC_bin.56	EPs-VS-CPs	0.008515306	0.014595333	CPs
TP027MC_bin.112	EPs-VS-CPs	0.01412334	0.023399151	EPs
ERR1135241_bin.24	EPs-VS-CPs	0.003019379	0.005582413	EPs
TP043_bin.15	EPs-VS-CPs	2.48E-10	9.45E-10	CPs
ERR1135328_bin.48	EPs-VS-CPs	2.93E-06	7.64E-06	CPs
ERR1135266_bin.18	EPs-VS-CPs	0.000442099	0.000922417	EPs
ERR1135189_bin.44	EPs-VS-CPs	0.00411652	0.007532894	EPs
ERR1135465_bin.7	EPs-VS-CPs	1.53E-21	1.41E-20	CPs
ERR1135211_bin.21	EPs-VS-CPs	1.35E-10	5.33E-10	CPs
ERR1135435_bin.8	EPs-VS-CPs	1.28E-08	4.22E-08	CPs
ERR1135242_bin.36	EPs-VS-CPs	4.62E-30	9.83E-29	EPs
ERR1135322_bin.22	EPs-VS-CPs	1.23E-24	1.54E-23	EPs
TP043_bin.149	EPs-VS-CPs	1.08E-09	3.93E-09	EPs
ERR1135317_bin.24	EPs-VS-CPs	0.008294746	0.014382929	EPs
ERR1135415_bin.65	EPs-VS-CPs	4.81E-10	1.80E-09	CPs
TP043MC_bin.147	EPs-VS-CPs	1.13E-17	7.41E-17	EPs
ERR1135381_bin.24	EPs-VS-CPs	2.81E-11	1.16E-10	CPs
ERR1135188_bin.61	EPs-VS-CPs	5.10E-15	2.78E-14	EPs
ERR1135322_bin.29	EPs-VS-CPs	6.45E-16	3.64E-15	EPs
ERR1135197_bin.36	EPs-VS-CPs	0.000411115	0.00086791	EPs
ERR1135337_bin.78	EPs-VS-CPs	5.91E-06	1.51E-05	EPs
TP012_bin.84	EPs-VS-CPs	0.03214519	0.049492507	CPs
ERR1135208_bin.20	EPs-VS-CPs	4.70E-17	2.89E-16	CPs
ERR1135245_bin.30	EPs-VS-CPs	0.002595699	0.004879914	CPs
ERR1135204_bin.76	EPs-VS-CPs	0.010204693	0.017259074	CPs
ERR1135275_bin.23	EPs-VS-CPs	8.82E-06	2.22E-05	EPs
TP042_bin.53	EPs-VS-CPs	7.23E-09	2.44E-08	CPs
TP010_bin.119	EPs-VS-CPs	2.66E-23	2.90E-22	CPs
ERR1135198_bin.54	EPs-VS-CPs	1.21E-13	5.99E-13	EPs
ERR1135292_bin.87	EPs-VS-CPs	2.23E-06	5.88E-06	EPs
ERR1135360_bin.30	EPs-VS-CPs	6.32E-39	2.82E-37	CPs
TP052MC_bin.26	EPs-VS-CPs	8.63E-29	1.60E-27	CPs
TP052MC_bin.39	EPs-VS-CPs	2.32E-07	6.69E-07	CPs
ERR1135421_bin.27	EPs-VS-CPs	6.20E-13	2.90E-12	CPs
ERR1135380_bin.51	EPs-VS-CPs	1.43E-05	3.53E-05	CPs
ERR1135178_bin.36	EPs-VS-CPs	3.50E-16	2.00E-15	EPs
ERR1135304_bin.16	EPs-VS-CPs	6.67E-27	9.45E-26	EPs
ERR1135182_bin.35	EPs-VS-CPs	0.010331961	0.017441288	EPs
TP003MC_bin.120	EPs-VS-CPs	6.55E-09	2.22E-08	CPs
ERR1135191_bin.13	EPs-VS-CPs	2.15E-26	2.96E-25	CPs
TP037MC_bin.44	EPs-VS-CPs	0.000647976	0.001315096	CPs
ERR1135192_bin.68	EPs-VS-CPs	1.10E-06	2.96E-06	EPs
ERR1135284_bin.14	EPs-VS-CPs	0.000905156	0.001812342	EPs
ERR1135408_bin.15	EPs-VS-CPs	1.17E-19	9.17E-19	CPs
TP007MC_bin.123	EPs-VS-CPs	2.59E-07	7.39E-07	EPs
ERR1135193_bin.58	EPs-VS-CPs	7.80E-12	3.40E-11	CPs
ERR1135220_bin.70	EPs-VS-CPs	4.78E-06	1.23E-05	EPs
ERR1135221_bin.29	EPs-VS-CPs	1.18E-09	4.22E-09	EPs
ERR1135178_bin.56	EPs-VS-CPs	1.78E-13	8.64E-13	EPs
ERR1135276_bin.43	EPs-VS-CPs	1.63E-17	1.04E-16	EPs
ERR1135371_bin.47	EPs-VS-CPs	0.02615844	0.041271178	EPs
ERR1135396_bin.46	EPs-VS-CPs	1.17E-09	4.22E-09	CPs
ERR1135464_bin.7	EPs-VS-CPs	5.37E-12	2.37E-11	CPs
ERR1135195_bin.54	EPs-VS-CPs	1.80E-05	4.37E-05	EPs
TP008_bin.78	EPs-VS-CPs	0.008515306	0.014595333	CPs
ERR1135471_bin.59	EPs-VS-CPs	1.78E-09	6.31E-09	CPs
ERR1135391_bin.3	EPs-VS-CPs	2.57E-35	7.90E-34	CPs
ERR1135407_bin.53	EPs-VS-CPs	3.03E-20	2.53E-19	CPs

SGBs	Compared groups	p values	Fdr	Enriched Group
TP021_bin.106	EPs-VS-CPs	0.001431189	0.002802746	EPs
ERR1135257_bin.52	EPs-VS-CPs	0.006899522	0.012080927	EPs
TP014_bin.83	EPs-VS-CPs	0.000647976	0.001315096	CPs
ERR1135195_bin.18	EPs-VS-CPs	4.57E-18	3.12E-17	EPs
ERR1135327_bin.92	EPs-VS-CPs	0.00154993	0.003028638	EPs
TP058_bin.7	EPs-VS-CPs	0.03214519	0.049492507	CPs
ERR1135205_bin.60	EPs-VS-CPs	3.40E-27	5.15E-26	EPs
ERR1135278_bin.32	EPs-VS-CPs	4.67E-17	2.89E-16	EPs
ERR1135267_bin.37	EPs-VS-CPs	1.34E-07	3.96E-07	EPs
ERR1135468_bin.29	EPs-VS-CPs	3.10E-09	1.08E-08	CPs
ERR1135378_bin.20	EPs-VS-CPs	1.27E-17	8.25E-17	CPs
ERR1135449_bin.10	EPs-VS-CPs	4.97E-19	3.73E-18	CPs
ERR1135192_bin.10	EPs-VS-CPs	0.016277853	0.026525772	EPs
ERR1135194_bin.34	EPs-VS-CPs	1.50E-18	1.06E-17	CPs
ERR1135202_bin.10	EPs-VS-CPs	0.000769496	0.001544179	EPs
ERR1135209_bin.42	EPs-VS-CPs	3.81E-07	1.06E-06	CPs
ERR1135179_bin.22	EPs-VS-CPs	0.001333339	0.00261686	EPs
ERR1135247_bin.31	EPs-VS-CPs	0.0092168	0.015647533	EPs
ERR1135419_bin.55	EPs-VS-CPs	7.00E-11	2.80E-10	CPs
ERR1135250_bin.5	EPs-VS-CPs	9.28E-37	3.31E-35	EPs
ERR1135420_bin.28	EPs-VS-CPs	1.57E-20	1.34E-19	CPs
TP025_bin.115	EPs-VS-CPs	1.22E-05	3.04E-05	CPs
ERR1135415_bin.35	EPs-VS-CPs	1.43E-05	3.53E-05	CPs
ERR1135285_bin.14	EPs-VS-CPs	1.69E-12	7.86E-12	EPs
ERR1135203_bin.44	EPs-VS-CPs	6.10E-05	0.000139248	EPs
ERR1135199_bin.45	EPs-VS-CPs	3.32E-13	1.57E-12	EPs
TP052MC_bin.128	EPs-VS-CPs	0.000442099	0.000922417	EPs
ERR1135272_bin.51	EPs-VS-CPs	6.65E-31	1.45E-29	EPs
TP008MC_bin.100	EPs-VS-CPs	1.79E-11	7.63E-11	CPs
ERR1135184_bin.93	EPs-VS-CPs	0.011644241	0.019436089	EPs
ERR1135278_bin.75	EPs-VS-CPs	3.87E-18	2.66E-17	EPs
ERR1135472_bin.83	EPs-VS-CPs	3.47E-09	1.20E-08	CPs
ERR1135407_bin.25	EPs-VS-CPs	5.10E-05	0.00011802	CPs
ERR1135201_bin.67	EPs-VS-CPs	4.18E-14	2.12E-13	EPs
TP021_bin.45	EPs-VS-CPs	0.00426457	0.007771961	CPs
ERR1135183_bin.20	EPs-VS-CPs	1.13E-09	4.11E-09	CPs
ERR1135223_bin.22	EPs-VS-CPs	0.000252744	0.00054255	EPs
ERR1135209_bin.28	EPs-VS-CPs	4.45E-13	2.09E-12	CPs
ERR1135385_bin.9	EPs-VS-CPs	0.00772413	0.013471968	EPs
ERR1135277_bin.29	EPs-VS-CPs	4.33E-20	3.52E-19	EPs
ERR1135348_bin.33	EPs-VS-CPs	0.001013453	0.002011141	EPs
ERR1135256_bin.60	EPs-VS-CPs	7.48E-07	2.02E-06	EPs
ERR1135289_bin.33	EPs-VS-CPs	0.025170721	0.039924429	CPs
ERR1135383_bin.33	EPs-VS-CPs	8.68E-08	2.62E-07	CPs
ERR1135301_bin.23	EPs-VS-CPs	0.000503816	0.001046297	EPs
ERR1135348_bin.59	EPs-VS-CPs	2.18E-08	6.92E-08	EPs
ERR1135256_bin.12	EPs-VS-CPs	1.81E-17	1.15E-16	EPs
ERR1135328_bin.36	EPs-VS-CPs	8.91E-28	1.53E-26	EPs
ERR1135264_bin.17	EPs-VS-CPs	6.48E-09	2.20E-08	EPs
ERR1135266_bin.21	EPs-VS-CPs	7.34E-10	2.70E-09	EPs
ERR1135464_bin.8	EPs-VS-CPs	1.73E-14	9.15E-14	CPs
TP008MC_bin.88	EPs-VS-CPs	0.008515306	0.014595333	CPs
TP043_bin.162	EPs-VS-CPs	2.93E-09	1.02E-08	CPs
ERR1135256_bin.53	EPs-VS-CPs	5.83E-17	3.56E-16	EPs
ERR1135286_bin.84	EPs-VS-CPs	0.000619756	0.001263566	EPs
ERR1135429_bin.48	EPs-VS-CPs	3.44E-11	1.40E-10	CPs
TP045_bin.16	EPs-VS-CPs	0.007702439	0.013460427	EPs
ERR1135178_bin.73	EPs-VS-CPs	2.12E-13	1.01E-12	EPs
ERR1135426_bin.24	EPs-VS-CPs	7.03E-43	4.18E-41	CPs
ERR1135391_bin.5	EPs-VS-CPs	7.50E-22	7.05E-21	CPs
ERR1135386_bin.37	EPs-VS-CPs	1.84E-36	6.09E-35	CPs
ERR1135181_bin.58	EPs-VS-CPs	1.21E-08	3.99E-08	CPs
TP015_bin.102	EPs-VS-CPs	1.43E-10	5.60E-10	CPs
ERR1135385_bin.68	EPs-VS-CPs	4.65E-21	4.20E-20	CPs
ERR1135190_bin.27	EPs-VS-CPs	3.85E-05	9.01E-05	EPs
ERR1135456_bin.36	EPs-VS-CPs	0.002528195	0.004763034	CPs
TP018MC_bin.11	EPs-VS-CPs	2.08E-08	6.63E-08	EPs
ERR1135278_bin.69	EPs-VS-CPs	8.81E-19	6.35E-18	EPs
ERR1135288_bin.11	EPs-VS-CPs	1.16E-15	6.42E-15	EPs
ERR1135335_bin.32	EPs-VS-CPs	0.011068784	0.018579744	CPs
ERR1135472_bin.52	EPs-VS-CPs	1.29E-10	5.13E-10	CPs
TP014_bin.7	EPs-VS-CPs	0.014505228	0.02394301	CPs
TP025_bin.34	EPs-VS-CPs	5.18E-34	1.40E-32	CPs
ERR1135279_bin.11	EPs-VS-CPs	0.00046397	0.000965794	EPs
ERR1135373_bin.49	EPs-VS-CPs	1.07E-08	3.58E-08	EPs
TP047MC_bin.79	EPs-VS-CPs	0.000116084	0.000258512	CPs
TP036MC_bin.37	EPs-VS-CPs	0.016132143	0.026336388	EPs
ERR1135288_bin.89	EPs-VS-CPs	0.000518002	0.001070777	EPs
TP041MC_bin.118	EPs-VS-CPs	2.75E-05	6.52E-05	CPs
ERR1135402_bin.1	EPs-VS-CPs	1.27E-24	1.58E-23	CPs
ERR1135377_bin.41	EPs-VS-CPs	1.40E-21	1.30E-20	CPs
ERR1135328_bin.73	EPs-VS-CPs	8.50E-16	4.72E-15	EPs
TP005MC_bin.82	EPs-VS-CPs	4.79E-40	2.38E-38	CPs
TP025_bin.117	EPs-VS-CPs	0.015244076	0.024977908	EPs
TP045_bin.44	EPs-VS-CPs	2.08E-09	7.35E-09	CPs
ERR1135196_bin.23	EPs-VS-CPs	3.20E-22	3.18E-21	EPs
ERR1135318_bin.98	EPs-VS-CPs	7.07E-29	1.34E-27	CPs
ERR1135196_bin.51	EPs-VS-CPs	5.31E-10	1.98E-09	EPs
ERR1135196_bin.68	EPs-VS-CPs	7.27E-19	5.28E-18	EPs
ERR1135273_bin.20	EPs-VS-CPs	3.12E-26	4.23E-25	EPs
TP025MC_bin.63	EPs-VS-CPs	0.03214519	0.049492507	CPs
TP027_bin.9	EPs-VS-CPs	4.58E-06	1.18E-05	CPs
ERR1135407_bin.2	EPs-VS-CPs	9.94E-44	6.85E-42	CPs
ERR1135178_bin.26	EPs-VS-CPs	6.54E-12	2.86E-11	EPs
ERR1135422_bin.61	EPs-VS-CPs	2.29E-19	1.76E-18	CPs

SGBs	Compared groups	p values	Fdr	Enriched Group
ERR1135192_bin.18	EPs-VS-CPs	1.18E-09	4.22E-09	EPs
ERR1135419_bin.50	EPs-VS-CPs	3.28E-22	3.18E-21	CPs
ERR1135222_bin.9	EPs-VS-CPs	0.000583607	0.001200785	EPs
ERR1135297_bin.27	EPs-VS-CPs	3.56E-07	9.98E-07	EPs
ERR1135289_bin.22	EPs-VS-CPs	3.42E-05	8.03E-05	EPs
ERR1135318_bin.25	EPs-VS-CPs	1.28E-28	2.29E-27	CPs
ERR1135294_bin.61	EPs-VS-CPs	1.54E-20	1.33E-19	EPs
ERR1135351_bin.65	EPs-VS-CPs	1.95E-10	7.48E-10	EPs
ERR1135197_bin.2	EPs-VS-CPs	5.78E-06	1.48E-05	EPs
ERR1135267_bin.21	EPs-VS-CPs	1.54E-08	5.03E-08	EPs
ERR1135229_bin.55	EPs-VS-CPs	2.81E-10	1.07E-09	EPs
TP053MC_bin.2	EPs-VS-CPs	0.001279101	0.002527074	EPs
TP023_bin.58	EPs-VS-CPs	3.65E-21	3.33E-20	EPs
TP059MC_bin.116	EPs-VS-CPs	5.71E-15	3.07E-14	CPs
TP024_bin.125	EPs-VS-CPs	3.13E-07	8.85E-07	CPs
ERR1135178_bin.34	EPs-VS-CPs	7.96E-05	0.000180922	EPs
ERR1135181_bin.68	EPs-VS-CPs	1.01E-18	7.23E-18	CPs
ERR1135227_bin.69	EPs-VS-CPs	6.10E-05	0.000139248	EPs
ERR1135312_bin.41	EPs-VS-CPs	0.000190723	0.000416419	EPs
TP058_bin.96	EPs-VS-CPs	1.48E-09	5.27E-09	EPs
ERR1135198_bin.40	EPs-VS-CPs	2.05E-23	2.26E-22	EPs
ERR1135213_bin.9	EPs-VS-CPs	0.019962915	0.03206274	EPs
ERR1135193_bin.102	EPs-VS-CPs	0.004196294	0.007663171	CPs
ERR1135201_bin.99	EPs-VS-CPs	3.85E-06	9.94E-06	EPs
ERR1135462_bin.24	EPs-VS-CPs	2.82E-25	3.59E-24	CPs
ERR1135219_bin.28	EPs-VS-CPs	2.31E-16	1.37E-15	EPs
ERR1135416_bin.41	EPs-VS-CPs	9.87E-29	1.80E-27	CPs
ERR1135252_bin.29	EPs-VS-CPs	1.38E-05	3.43E-05	CPs
ERR1135192_bin.82	EPs-VS-CPs	1.88E-11	7.95E-11	CPs
ERR1135267_bin.18	EPs-VS-CPs	1.24E-07	3.67E-07	EPs
ERR1135226_bin.23	EPs-VS-CPs	6.62E-22	6.28E-21	EPs
ERR1135197_bin.34	EPs-VS-CPs	1.71E-06	4.55E-06	EPs
ERR1135356_bin.12	EPs-VS-CPs	2.27E-31	5.33E-30	CPs
ERR1135401_bin.33	EPs-VS-CPs	1.73E-32	4.40E-31	CPs
ERR1135388_bin.55	EPs-VS-CPs	9.82E-37	3.37E-35	CPs
TP041_bin.152	EPs-VS-CPs	0.003440494	0.006334765	EPs
ERR1135212_bin.78	EPs-VS-CPs	0.014264986	0.023590061	EPs
ERR1135200_bin.65	EPs-VS-CPs	4.81E-27	7.17E-26	CPs
ERR1135450_bin.9	EPs-VS-CPs	1.99E-43	1.27E-41	CPs
ERR1135178_bin.57	EPs-VS-CPs	0.001013453	0.002011141	EPs
TP012_bin.137	EPs-VS-CPs	0.006793832	0.011919237	EPs
ERR1135273_bin.81	EPs-VS-CPs	0.000334488	0.000709496	EPs
TP004MC_bin.63	EPs-VS-CPs	2.62E-08	8.27E-08	EPs
TP034MC_bin.90	EPs-VS-CPs	2.29E-11	9.56E-11	CPs
TP036MC_bin.50	EPs-VS-CPs	0.000296073	0.000632519	EPs
ERR1135218_bin.58	EPs-VS-CPs	1.95E-10	7.48E-10	EPs
ERR1135185_bin.73	EPs-VS-CPs	5.84E-16	3.32E-15	EPs
TP043MC_bin.59	EPs-VS-CPs	8.06E-05	0.00018271	EPs
ERR1135356_bin.72	EPs-VS-CPs	0.005187304	0.009264525	EPs
ERR1135418_bin.17	EPs-VS-CPs	4.18E-12	1.86E-11	CPs
ERR1135263_bin.42	EPs-VS-CPs	9.63E-12	4.16E-11	EPs
TP019MC_bin.9	EPs-VS-CPs	1.63E-18	1.14E-17	CPs
ERR1135183_bin.75	EPs-VS-CPs	7.58E-21	6.70E-20	CPs
TP021_bin.93	EPs-VS-CPs	0.005187304	0.009264525	EPs
ERR1135290_bin.47	EPs-VS-CPs	0.029875964	0.046805677	EPs
TP027MC_bin.116	EPs-VS-CPs	1.81E-24	2.21E-23	EPs
TP007MC_bin.50	EPs-VS-CPs	1.47E-20	1.28E-19	CPs
TP056_bin.56	EPs-VS-CPs	0.001013453	0.002011141	EPs
ERR1135471_bin.11	EPs-VS-CPs	1.32E-16	7.96E-16	CPs
TP042_bin.125	EPs-VS-CPs	2.43E-36	7.75E-35	CPs
ERR1135425_bin.34	EPs-VS-CPs	5.34E-07	1.46E-06	CPs
TP058_bin.70	EPs-VS-CPs	8.95E-06	2.25E-05	CPs
ERR1135449_bin.13	EPs-VS-CPs	1.91E-28	3.34E-27	CPs
ERR1135196_bin.4	EPs-VS-CPs	1.87E-08	6.00E-08	EPs
TP056_bin.100	EPs-VS-CPs	2.89E-05	6.84E-05	CPs
ERR1135219_bin.61	EPs-VS-CPs	3.30E-05	7.77E-05	EPs
ERR1135217_bin.65	EPs-VS-CPs	2.59E-07	7.39E-07	EPs
ERR1135192_bin.94	EPs-VS-CPs	0.000252744	0.00054255	EPs
ERR1135210_bin.54	EPs-VS-CPs	1.67E-10	6.50E-10	CPs
TP043MC_bin.127	EPs-VS-CPs	0.030325182	0.047426248	EPs
TP045_bin.86	EPs-VS-CPs	2.55E-09	8.94E-09	CPs
TP049MC_bin.106	EPs-VS-CPs	0.03214519	0.049492507	CPs
ERR1135374_bin.10	EPs-VS-CPs	0.011644241	0.019436089	EPs
TP053MC_bin.119	EPs-VS-CPs	1.43E-05	3.53E-05	CPs
ERR1135446_bin.34	EPs-VS-CPs	5.39E-51	1.20E-48	CPs
TP031MC_bin.46	EPs-VS-CPs	0.000442099	0.000922417	EPs
ERR1135419_bin.72	EPs-VS-CPs	3.13E-07	8.85E-07	CPs
ERR1135233_bin.32	EPs-VS-CPs	0.003019379	0.005582413	EPs
ERR1135270_bin.77	EPs-VS-CPs	5.18E-26	6.80E-25	EPs
ERR1135308_bin.99	EPs-VS-CPs	5.43E-19	3.98E-18	EPs
ERR1135370_bin.25	EPs-VS-CPs	0.000334488	0.000709496	EPs
ERR1135209_bin.24	EPs-VS-CPs	2.46E-05	5.88E-05	CPs
TP027MC_bin.2	EPs-VS-CPs	0.005689373	0.010140938	EPs
ERR1135245_bin.31	EPs-VS-CPs	1.87E-10	7.25E-10	EPs
TP014MC_bin.66	EPs-VS-CPs	1.07E-07	3.20E-07	CPs
ERR1135328_bin.18	EPs-VS-CPs	9.41E-24	1.09E-22	EPs
ERR1135439_bin.54	EPs-VS-CPs	2.78E-11	1.15E-10	CPs
TP021_bin.7	EPs-VS-CPs	0.01370582	0.022791988	EPs
TP002_bin.148	EPs-VS-CPs	0.008515306	0.014595333	CPs
ERR1135227_bin.18	EPs-VS-CPs	1.81E-05	4.40E-05	EPs
TP029_bin.43	EPs-VS-CPs	6.08E-06	1.55E-05	CPs
ERR1135385_bin.11	EPs-VS-CPs	1.93E-13	9.25E-13	CPs
ERR1135225_bin.13	EPs-VS-CPs	1.63E-17	1.04E-16	EPs
ERR1135220_bin.65	EPs-VS-CPs	0.009038159	0.015373479	EPs
ERR1135270_bin.3	EPs-VS-CPs	3.03E-11	1.24E-10	EPs

SGBs	Compared groups	p values	Fdr	Enriched Group
TP036_bin.120	EPs-VS-CPs	0.019962915	0.03206274	EPs
TP044MC_bin.16	EPs-VS-CPs	0.019277322	0.031356372	EPs
ERR1135275_bin.2	EPs-VS-CPs	6.10E-05	0.000139248	EPs
ERR1135219_bin.51	EPs-VS-CPs	2.07E-11	8.70E-11	EPs
ERR1135191_bin.26	EPs-VS-CPs	6.73E-10	2.48E-09	CPs
TP036MC_bin.106	EPs-VS-CPs	0.03214519	0.049492507	CPs
ERR1135190_bin.104	EPs-VS-CPs	0.002301235	0.004381669	EPs
ERR1135201_bin.17	EPs-VS-CPs	1.01E-17	6.74E-17	CPs
ERR1135187_bin.70	EPs-VS-CPs	4.13E-08	1.29E-07	CPs
ERR1135275_bin.62	EPs-VS-CPs	0.001080354	0.002139149	EPs
TP015_bin.34	EPs-VS-CPs	3.45E-14	1.77E-13	CPs
ERR1135277_bin.54	EPs-VS-CPs	2.99E-12	1.36E-11	EPs
ERR1135224_bin.9	EPs-VS-CPs	4.17E-19	3.15E-18	EPs
ERR1135353_bin.10	EPs-VS-CPs	0.014869742	0.024499409	CPs
TP041_bin.34	EPs-VS-CPs	0.019962915	0.03206274	EPs
ERR1135265_bin.18	EPs-VS-CPs	5.66E-14	2.82E-13	EPs
ERR1135217_bin.5	EPs-VS-CPs	0.00803621	0.013961741	CPs
TP047MC_bin.22	EPs-VS-CPs	2.32E-06	6.08E-06	EPs
ERR1135201_bin.83	EPs-VS-CPs	0.006793832	0.011919237	EPs
TP014MC_bin.156	EPs-VS-CPs	0.005187304	0.009264525	EPs
ERR1135193_bin.27	EPs-VS-CPs	1.44E-07	4.22E-07	EPs
ERR1135246_bin.54	EPs-VS-CPs	2.02E-12	9.23E-12	EPs
TP005_bin.95	EPs-VS-CPs	4.28E-15	2.35E-14	EPs
ERR1135464_bin.16	EPs-VS-CPs	0.000382659	0.00080975	CPs
ERR1135291_bin.88	EPs-VS-CPs	0.008894998	0.015158842	EPs
ERR1135202_bin.68	EPs-VS-CPs	0.010798244	0.018159759	EPs
ERR1135221_bin.12	EPs-VS-CPs	1.22E-22	1.30E-21	EPs
ERR1135205_bin.43	EPs-VS-CPs	2.55E-05	6.07E-05	EPs
ERR1135196_bin.11	EPs-VS-CPs	1.53E-33	4.02E-32	EPs
ERR1135341_bin.54	EPs-VS-CPs	6.43E-11	2.59E-10	EPs
ERR1135350_bin.18	EPs-VS-CPs	1.87E-05	4.52E-05	EPs
ERR1135196_bin.55	EPs-VS-CPs	0.000584929	0.001200785	EPs
ERR1135205_bin.8	EPs-VS-CPs	1.40E-22	1.45E-21	CPs
TP043MC_bin.109	EPs-VS-CPs	0.03214519	0.049492507	CPs
TP002_bin.164	EPs-VS-CPs	1.30E-10	5.14E-10	CPs
ERR1135275_bin.38	EPs-VS-CPs	8.13E-05	0.000182792	EPs
ERR1135197_bin.41	EPs-VS-CPs	8.18E-05	0.000183495	EPs
TP020_bin.111	EPs-VS-CPs	2.49E-37	9.25E-36	EPs
ERR1135456_bin.15	EPs-VS-CPs	0.02204834	0.035159228	EPs
ERR1135260_bin.45	EPs-VS-CPs	0.001975761	0.003778061	CPs
ERR1135265_bin.33	EPs-VS-CPs	1.77E-08	5.75E-08	EPs
TP026_bin.65	EPs-VS-CPs	0.008894998	0.015158842	EPs
ERR1135299_bin.83	EPs-VS-CPs	0.000619756	0.001263566	EPs
TP045_bin.48	EPs-VS-CPs	0.000334488	0.000709496	EPs
ERR1135190_bin.64	EPs-VS-CPs	5.41E-08	1.67E-07	EPs
TP036_bin.122	EPs-VS-CPs	1.45E-05	3.56E-05	CPs
TP042_bin.127	EPs-VS-CPs	5.53E-09	1.90E-08	CPs
TP058_bin.84	EPs-VS-CPs	0.006131346	0.010863675	EPs
ERR1135252_bin.17	EPs-VS-CPs	5.45E-45	5.41E-43	CPs
ERR1135330_bin.69	EPs-VS-CPs	2.13E-24	2.54E-23	EPs
ERR1135260_bin.22	EPs-VS-CPs	6.10E-05	0.000139248	EPs
ERR1135445_bin.40	EPs-VS-CPs	3.68E-17	2.30E-16	CPs
TP043_bin.130	EPs-VS-CPs	1.12E-17	7.41E-17	CPs
ERR1135202_bin.70	EPs-VS-CPs	2.76E-06	7.22E-06	CPs
ERR1135267_bin.30	EPs-VS-CPs	7.77E-08	2.37E-07	EPs
ERR1135419_bin.22	EPs-VS-CPs	0.001923682	0.003694296	CPs
TP015_bin.88	EPs-VS-CPs	0.023657139	0.037590437	EPs
ERR1135202_bin.14	EPs-VS-CPs	0.021555796	0.034435288	CPs
ERR1135341_bin.51	EPs-VS-CPs	1.59E-22	1.63E-21	EPs
ERR1135342_bin.51	EPs-VS-CPs	5.05E-19	3.76E-18	EPs
ERR1135371_bin.30	EPs-VS-CPs	0.002893434	0.005394231	CPs
ERR1135266_bin.58	EPs-VS-CPs	2.54E-22	2.58E-21	EPs
ERR1135277_bin.10	EPs-VS-CPs	0.0140217	0.023273937	CPs
ERR1135333_bin.56	EPs-VS-CPs	3.38E-07	9.48E-07	CPs
ERR1135453_bin.4	EPs-VS-CPs	3.54E-42	1.98E-40	CPs
ERR1135181_bin.32	EPs-VS-CPs	0.000533664	0.001100604	CPs
ERR1135200_bin.11	EPs-VS-CPs	0.019962915	0.03206274	EPs
ERR1135219_bin.8	EPs-VS-CPs	2.66E-11	1.10E-10	EPs
ERR1135319_bin.78	EPs-VS-CPs	0.020697768	0.033183315	CPs
ERR1135263_bin.3	EPs-VS-CPs	0.002636199	0.004945643	EPs
TP025_bin.136	EPs-VS-CPs	2.11E-37	8.21E-36	CPs
ERR1135191_bin.71	EPs-VS-CPs	0.025338558	0.040119383	EPs
ERR1135232_bin.13	EPs-VS-CPs	6.10E-05	0.000139248	EPs
ERR1135389_bin.61	EPs-VS-CPs	3.14E-14	1.62E-13	CPs
ERR1135199_bin.23	EPs-VS-CPs	5.57E-09	1.91E-08	EPs
ERR1135453_bin.41	EPs-VS-CPs	1.13E-23	1.29E-22	EPs
ERR1135294_bin.23	EPs-VS-CPs	4.10E-07	1.14E-06	EPs
ERR1135304_bin.69	EPs-VS-CPs	7.09E-05	0.000161628	EPs
ERR1135458_bin.26	EPs-VS-CPs	3.70E-31	8.46E-30	CPs
ERR1135223_bin.20	EPs-VS-CPs	0.00175246	0.003380015	EPs
TP049MC_bin.131	EPs-VS-CPs	1.75E-12	8.08E-12	CPs
ERR1135383_bin.11	EPs-VS-CPs	4.16E-19	3.15E-18	EPs
ERR1135260_bin.46	EPs-VS-CPs	0.021074345	0.033726506	CPs
TP021MC_bin.45	EPs-VS-CPs	1.70E-08	5.53E-08	EPs
ERR1135306_bin.47	EPs-VS-CPs	9.63E-12	4.16E-11	EPs
ERR1135206_bin.69	EPs-VS-CPs	1.36E-07	3.99E-07	EPs
ERR1135205_bin.52	EPs-VS-CPs	1.55E-05	3.79E-05	EPs
TP018MC_bin.74	EPs-VS-CPs	3.28E-12	1.48E-11	CPs
TP047MC_bin.9	EPs-VS-CPs	5.22E-07	1.44E-06	CPs
ERR1135325_bin.58	EPs-VS-CPs	1.69E-07	4.93E-07	EPs
ERR1135178_bin.9	EPs-VS-CPs	6.71E-07	1.82E-06	EPs
ERR1135350_bin.27	EPs-VS-CPs	4.72E-08	1.46E-07	CPs
TP020MC_bin.144	EPs-VS-CPs	0.002330532	0.00441861	CPs
ERR1135233_bin.45	EPs-VS-CPs	4.57E-05	0.00010594	EPs
ERR1135397_bin.48	EPs-VS-CPs	9.58E-18	6.43E-17	CPs

SGBs	Compared groups	p values	Fdr	Enriched Group
ERR1135407_bin.19	EPs-VS-CPs	1.09E-52	4.85E-50	CPs
ERR1135216_bin.67	EPs-VS-CPs	2.89E-16	1.66E-15	EPs
ERR1135348_bin.2	EPs-VS-CPs	5.81E-27	8.37E-26	EPs
TP041_bin.136	EPs-VS-CPs	2.33E-09	8.18E-09	CPs
ERR1135311_bin.91	EPs-VS-CPs	2.07E-11	8.70E-11	EPs
ERR1135214_bin.3	EPs-VS-CPs	4.13E-14	2.11E-13	EPs
TP033_bin.130	EPs-VS-CPs	0.029523061	0.046334083	EPs
ERR1135410_bin.13	EPs-VS-CPs	0.004802749	0.008699503	CPs
TP005_bin.79	EPs-VS-CPs	4.05E-20	3.32E-19	EPs
ERR1135224_bin.55	EPs-VS-CPs	0.000442099	0.000922417	EPs
ERR1135446_bin.30	EPs-VS-CPs	4.29E-32	1.06E-30	CPs
ERR1135202_bin.31	EPs-VS-CPs	7.68E-15	4.10E-14	CPs
ERR1135385_bin.10	EPs-VS-CPs	1.66E-07	4.84E-07	CPs
ERR1135259_bin.56	EPs-VS-CPs	1.06E-47	1.65E-45	CPs
ERR1135441_bin.78	EPs-VS-CPs	4.94E-27	7.23E-26	CPs
TP029MC_bin.20	EPs-VS-CPs	0.03214519	0.049492507	CPs
ERR1135348_bin.60	EPs-VS-CPs	2.59E-07	7.39E-07	EPs
ERR1135389_bin.14	EPs-VS-CPs	3.25E-07	9.17E-07	CPs
ERR1135253_bin.21	EPs-VS-CPs	2.70E-08	8.50E-08	CPs
ERR1135458_bin.47	EPs-VS-CPs	0.00018154	0.000399298	CPs
TP012_bin.3	EPs-VS-CPs	2.10E-05	5.04E-05	CPs
ERR1135179_bin.29	EPs-VS-CPs	0.003958781	0.00725912	EPs
ERR1135196_bin.50	EPs-VS-CPs	1.05E-10	4.17E-10	EPs
ERR1135319_bin.63	EPs-VS-CPs	1.93E-24	2.33E-23	CPs
ERR1135360_bin.47	EPs-VS-CPs	4.00E-12	1.79E-11	CPs
ERR1135188_bin.29	EPs-VS-CPs	1.89E-13	9.11E-13	CPs
ERR1135462_bin.8	EPs-VS-CPs	3.11E-07	8.85E-07	CPs
TP025_bin.18	EPs-VS-CPs	3.59E-05	8.42E-05	EPs
TP027_bin.147	EPs-VS-CPs	0.003337218	0.006157305	EPs
ERR1135293_bin.44	EPs-VS-CPs	2.71E-14	1.41E-13	EPs
ERR1135373_bin.22	EPs-VS-CPs	4.99E-30	1.04E-28	EPs
ERR1135212_bin.26	EPs-VS-CPs	2.46E-16	1.45E-15	CPs
ERR1135190_bin.63	EPs-VS-CPs	1.45E-05	3.56E-05	EPs
ERR1135288_bin.96	EPs-VS-CPs	0.00186352	0.003586474	EPs
ERR1135300_bin.25	EPs-VS-CPs	1.01E-19	8.03E-19	EPs
ERR1135216_bin.5	EPs-VS-CPs	2.60E-05	6.17E-05	EPs
ERR1135265_bin.44	EPs-VS-CPs	0.002365686	0.004475758	EPs
ERR1135401_bin.43	EPs-VS-CPs	0.002844439	0.005313983	CPs
ERR1135190_bin.41	EPs-VS-CPs	0.006352799	0.011233761	EPs
TP030MC_bin.27	EPs-VS-CPs	0.000442099	0.000922417	EPs
ERR1135204_bin.18	EPs-VS-CPs	0.00022763	0.000493383	CPs
ERR1135189_bin.8	EPs-VS-CPs	0.029409515	0.046237143	EPs
ERR1135209_bin.29	EPs-VS-CPs	0.000190723	0.000416419	EPs
ERR1135223_bin.52	EPs-VS-CPs	3.66E-08	1.14E-07	EPs
ERR1135262_bin.42	EPs-VS-CPs	0.010442903	0.017595307	CPs
ERR1135324_bin.51	EPs-VS-CPs	2.55E-05	6.07E-05	EPs
ERR1135468_bin.57	EPs-VS-CPs	5.37E-14	2.70E-13	CPs
TP018MC_bin.85	EPs-VS-CPs	4.66E-22	4.47E-21	CPs
ERR1135383_bin.49	EPs-VS-CPs	3.03E-20	2.53E-19	CPs
ERR1135387_bin.51	EPs-VS-CPs	2.05E-27	3.20E-26	CPs
ERR1135466_bin.58	EPs-VS-CPs	1.11E-25	1.44E-24	CPs
ERR1135426_bin.43	EPs-VS-CPs	1.03E-11	4.40E-11	CPs
ERR1135350_bin.23	EPs-VS-CPs	5.56E-08	1.71E-07	EPs
ERR1135466_bin.30	EPs-VS-CPs	0.000155788	0.000345208	CPs
ERR1135219_bin.41	EPs-VS-CPs	0.002390108	0.004512403	EPs
ERR1135406_bin.22	EPs-VS-CPs	2.70E-22	2.71E-21	CPs
ERR1135470_bin.34	EPs-VS-CPs	9.97E-44	6.85E-42	CPs
ERR1135188_bin.70	EPs-VS-CPs	0.002325984	0.00441861	EPs
TP015MC_bin.87	EPs-VS-CPs	0.022638985	0.036036745	EPs
ERR1135291_bin.83	EPs-VS-CPs	2.33E-14	1.22E-13	EPs
ERR1135464_bin.77	EPs-VS-CPs	8.68E-08	2.62E-07	CPs
ERR1135453_bin.49	EPs-VS-CPs	2.45E-34	6.85E-33	CPs
ERR1135231_bin.4	EPs-VS-CPs	2.10E-05	5.04E-05	EPs
TP024_bin.19	EPs-VS-CPs	0.001333339	0.00261686	EPs
ERR1135200_bin.105	EPs-VS-CPs	7.06E-08	2.16E-07	EPs
ERR1135196_bin.29	EPs-VS-CPs	0.004629349	0.008402456	CPs
ERR1135208_bin.43	EPs-VS-CPs	8.11E-05	0.000182792	EPs
ERR1135189_bin.31	EPs-VS-CPs	3.78E-12	1.70E-11	CPs
ERR1135379_bin.27	EPs-VS-CPs	1.81E-23	2.02E-22	CPs
ERR1135252_bin.35	EPs-VS-CPs	1.12E-22	1.21E-21	CPs
ERR1135378_bin.53	EPs-VS-CPs	1.60E-34	4.60E-33	CPs
ERR1135447_bin.18	EPs-VS-CPs	1.08E-11	4.62E-11	CPs
TP002_bin.4	EPs-VS-CPs	0.004526912	0.008233263	EPs
ERR1135417_bin.29	EPs-VS-CPs	7.82E-06	1.98E-05	CPs
ERR1135273_bin.2	EPs-VS-CPs	1.88E-07	5.43E-07	EPs
ERR1135458_bin.60	EPs-VS-CPs	2.21E-06	5.87E-06	CPs
ERR1135426_bin.54	EPs-VS-CPs	1.24E-26	1.72E-25	CPs
ERR1135187_bin.93	EPs-VS-CPs	0.013602443	0.022662279	EPs
TP018MC_bin.51	EPs-VS-CPs	0.000143722	0.000319263	EPs
ERR1135472_bin.8	EPs-VS-CPs	1.44E-17	9.32E-17	CPs
ERR1135194_bin.3	EPs-VS-CPs	1.49E-18	1.05E-17	CPs
ERR1135212_bin.6	EPs-VS-CPs	0.00175246	0.003380015	EPs
ERR1135247_bin.55	EPs-VS-CPs	0.000769496	0.001544179	EPs
ERR1135422_bin.56	EPs-VS-CPs	0.000510129	0.00105695	CPs
ERR1135439_bin.5	EPs-VS-CPs	1.96E-38	8.35E-37	CPs
ERR1135190_bin.88	EPs-VS-CPs	4.37E-10	1.65E-09	CPs
ERR1135452_bin.26	EPs-VS-CPs	2.23E-46	2.84E-44	CPs
ERR1135467_bin.21	EPs-VS-CPs	1.48E-23	1.67E-22	CPs
ERR1135215_bin.52	EPs-VS-CPs	1.95E-10	7.48E-10	EPs
ERR1135258_bin.21	EPs-VS-CPs	3.07E-12	1.39E-11	CPs
ERR1135283_bin.47	EPs-VS-CPs	3.76E-20	3.11E-19	CPs
TP034MC_bin.112	EPs-VS-CPs	8.55E-08	2.60E-07	EPs
ERR1135220_bin.1	EPs-VS-CPs	0.000769496	0.001544179	EPs
ERR1135268_bin.39	EPs-VS-CPs	0.005187304	0.009264525	EPs
ERR1135191_bin.23	EPs-VS-CPs	9.52E-09	3.18E-08	EPs

SGBs	Compared groups	p values	Fdr	Enriched Group
ERR1135336_bin.80	EPs-VS-CPs	0.001974721	0.003778061	EPs
ERR1135194_bin.68	EPs-VS-CPs	7.54E-10	2.76E-09	EPs
TP058_bin.85	EPs-VS-CPs	0.015048876	0.024748888	CPs
TP009_bin.41	EPs-VS-CPs	1.87E-08	6.00E-08	EPs
ERR1135212_bin.14	EPs-VS-CPs	0.006793832	0.011919237	EPs
ERR1135276_bin.61	EPs-VS-CPs	1.87E-08	6.00E-08	EPs
ERR1135273_bin.8	EPs-VS-CPs	1.34E-08	4.39E-08	EPs
TP025_bin.31	EPs-VS-CPs	1.11E-08	3.68E-08	CPs
ERR1135201_bin.12	EPs-VS-CPs	5.27E-07	1.45E-06	CPs
ERR1135273_bin.39	EPs-VS-CPs	1.18E-09	4.22E-09	EPs
ERR1135217_bin.14	EPs-VS-CPs	0.000211451	0.00045943	EPs
ERR1135291_bin.45	EPs-VS-CPs	1.67E-06	4.47E-06	EPs
ERR1135295_bin.34	EPs-VS-CPs	1.85E-08	5.99E-08	EPs
ERR1135205_bin.45	EPs-VS-CPs	2.23E-06	5.88E-06	EPs
TP007MC_bin.20	EPs-VS-CPs	1.12E-06	3.02E-06	CPs
TP015MC_bin.18	EPs-VS-CPs	3.01E-20	2.53E-19	CPs
ERR1135293_bin.33	EPs-VS-CPs	7.23E-20	5.82E-19	EPs
ERR1135275_bin.52	EPs-VS-CPs	3.25E-22	3.18E-21	EPs
ERR1135309_bin.22	EPs-VS-CPs	4.79E-09	1.65E-08	EPs
ERR1135272_bin.36	EPs-VS-CPs	9.17E-07	2.47E-06	EPs
ERR1135197_bin.22	EPs-VS-CPs	0.02615844	0.041271178	EPs
ERR1135286_bin.7	EPs-VS-CPs	1.69E-16	1.01E-15	CPs
ERR1135295_bin.8	EPs-VS-CPs	5.10E-08	1.57E-07	EPs
ERR1135213_bin.14	EPs-VS-CPs	2.73E-08	8.55E-08	CPs
ERR1135460_bin.61	EPs-VS-CPs	1.37E-27	2.32E-26	CPs
TP009_bin.103	EPs-VS-CPs	0.030623955	0.047809776	EPs
ERR1135441_bin.14	EPs-VS-CPs	1.43E-05	3.53E-05	CPs
TP023_bin.85	EPs-VS-CPs	1.07E-07	3.20E-07	CPs
TP015MC_bin.75	EPs-VS-CPs	5.21E-15	2.82E-14	CPs
ERR1135419_bin.8	EPs-VS-CPs	0.005045083	0.009119957	EPs
TP021_bin.147	EPs-VS-CPs	0.000252744	0.00054255	EPs
ERR1135274_bin.69	EPs-VS-CPs	6.57E-16	3.67E-15	EPs
ERR1135351_bin.45	EPs-VS-CPs	6.71E-07	1.82E-06	EPs
ERR1135191_bin.44	EPs-VS-CPs	0.002649172	0.004959561	CPs
ERR1135352_bin.30	EPs-VS-CPs	0.008573261	0.014666518	CPs
ERR1135347_bin.19	EPs-VS-CPs	0.019962915	0.03206274	EPs
ERR1135241_bin.71	EPs-VS-CPs	0.015244076	0.024977908	EPs
TP003MC_bin.13	EPs-VS-CPs	4.55E-05	0.00010594	EPs
ERR1135261_bin.34	EPs-VS-CPs	0.003019379	0.005582413	EPs
ERR1135464_bin.47	EPs-VS-CPs	2.39E-08	7.57E-08	CPs
ERR1135266_bin.67	EPs-VS-CPs	1.05E-05	2.63E-05	EPs
ERR1135334_bin.33	EPs-VS-CPs	2.19E-14	1.15E-13	EPs
TP034MC_bin.67	EPs-VS-CPs	0.000252744	0.00054255	EPs
ERR1135204_bin.75	EPs-VS-CPs	9.80E-05	0.000219426	CPs
ERR1135465_bin.9	EPs-VS-CPs	2.37E-57	2.12E-54	CPs
ERR1135199_bin.71	EPs-VS-CPs	1.15E-07	3.41E-07	CPs
ERR1135458_bin.70	EPs-VS-CPs	3.30E-13	1.57E-12	CPs
ERR1135294_bin.5	EPs-VS-CPs	3.15E-06	8.16E-06	EPs
ERR1135181_bin.67	EPs-VS-CPs	0.026538943	0.041797665	EPs
ERR1135254_bin.65	EPs-VS-CPs	5.90E-09	2.01E-08	EPs
ERR1135351_bin.34	EPs-VS-CPs	0.003019379	0.005582413	EPs
ERR1135417_bin.79	EPs-VS-CPs	4.79E-10	1.80E-09	CPs
ERR1135196_bin.24	EPs-VS-CPs	2.32E-24	2.73E-23	EPs
ERR1135281_bin.21	EPs-VS-CPs	1.04E-05	2.61E-05	EPs
ERR1135198_bin.11	EPs-VS-CPs	4.96E-32	1.20E-30	EPs
ERR1135257_bin.51	EPs-VS-CPs	3.15E-06	8.16E-06	EPs

Supplementary Table 5. The enrichment analysis of CAZymes in three groups

CAZy Family	Compared groups	p value	Fdr	Enriched Group
GH53	TPs-VS-EPs	0.049358681	0.121674797	TPs
GH140	TPs-VS-EPs	0.04986672	0.121674797	TPs
CE15	TPs-VS-EPs	0.001230832	0.005631055	TPs
GH29	TPs-VS-EPs	1.12E-05	0.000157741	TPs
PL27	TPs-VS-EPs	0.042633123	0.106874816	TPs
PL12	TPs-VS-EPs	0.000969477	0.004928173	TPs
GH30	TPs-VS-EPs	0.000360826	0.002130036	TPs
PL35	TPs-VS-EPs	0.000210787	0.001330139	TPs
GH31	TPs-VS-EPs	0.002467822	0.010436847	TPs
GH112	TPs-VS-EPs	0.038023538	0.102328052	TPs
CE11	TPs-VS-EPs	0.00011192	0.000819253	TPs
CE6	TPs-VS-EPs	0.028153868	0.080502468	TPs
GH102	TPs-VS-EPs	6.39E-05	0.000584611	TPs
GH88	TPs-VS-EPs	7.93E-10	5.02E-08	TPs
GH36	TPs-VS-EPs	9.73E-05	0.000808979	TPs
GH123	TPs-VS-EPs	0.035153987	0.098971994	TPs
PL26	TPs-VS-EPs	0.004564072	0.016704502	TPs
PL1	TPs-VS-EPs	8.93E-06	0.000136159	TPs
GH38	TPs-VS-EPs	1.43E-06	2.62E-05	EPs
GH105	TPs-VS-EPs	4.54E-09	2.08E-07	TPs
GH84	TPs-VS-EPs	0.0001166	0.000820682	EPs
GH74	TPs-VS-EPs	2.86E-05	0.000313719	TPs
PL9	TPs-VS-EPs	0.000404066	0.00231075	TPs
GH95	TPs-VS-EPs	1.21E-05	0.000157741	TPs
GH133	TPs-VS-EPs	0.000280815	0.001712969	TPs
CE19	TPs-VS-EPs	0.002343534	0.010211111	TPs
GH120	TPs-VS-EPs	8.48E-05	0.000739154	TPs
GH10	TPs-VS-EPs	0.000104666	0.000819253	TPs
GH106	TPs-VS-EPs	0.00637766	0.021613182	TPs
GH68	TPs-VS-EPs	0.041526982	0.106874816	EPs
CE2	TPs-VS-EPs	0.013938725	0.045549762	TPs
PL40	TPs-VS-EPs	0.004181773	0.015617643	TPs
GH35	TPs-VS-EPs	2.28E-08	8.33E-07	TPs
GH99	TPs-VS-EPs	0.010719863	0.035667907	TPs
GH59	TPs-VS-EPs	0.024694752	0.072889347	TPs
CE7	TPs-VS-EPs	0.002509406	0.010436847	TPs
PL11	TPs-VS-EPs	0.004011465	0.015293711	TPs
GH89	TPs-VS-EPs	0.04208017	0.106874816	TPs
GH103	TPs-VS-EPs	0.003023827	0.012029572	TPs
GH2	TPs-VS-EPs	0.021967507	0.067000895	TPs
PL33	TPs-VS-EPs	5.74E-06	9.54E-05	TPs
PL17	TPs-VS-EPs	0.017998021	0.05778312	TPs
PL8	TPs-VS-EPs	0.001576351	0.007035907	TPs
GH26	TPs-VS-EPs	0.00042558	0.002360033	TPs
GH146	TPs-VS-EPs	8.65E-07	1.76E-05	TPs
PL29	TPs-VS-EPs	0.025341771	0.07361181	TPs
GH97	TPs-VS-EPs	0.000199239	0.00130217	TPs
GH78	TPs-VS-EPs	0.00081878	0.00428105	TPs
CE12	TPs-VS-EPs	8.23E-10	5.02E-08	TPs
GH43	TPs-VS-EPs	1.31E-17	2.39E-15	TPs
GH33	TPs-VS-EPs	0.006325113	0.021613182	TPs
GH170	TPs-VS-EPs	2.99E-07	7.81E-06	EPs
GH13	TPs-VS-EPs	0.041526982	0.106874816	TPs
GH42	TPs-VS-EPs	0.037194583	0.101591174	TPs
GH51	TPs-VS-EPs	6.98E-08	2.13E-06	TPs
GH93	TPs-VS-EPs	0.001215861	0.005631055	TPs
GH9	TPs-VS-EPs	0.005172875	0.018561492	TPs
GH148	TPs-VS-EPs	0.003320593	0.012929117	TPs
CE1	TPs-VS-EPs	0.000147952	0.001002785	TPs
GH110	TPs-VS-EPs	0.000522219	0.002810767	TPs
GH92	TPs-VS-EPs	4.12E-05	0.000418668	TPs
GH113	TPs-VS-EPs	0.018398177	0.058049421	TPs
CE3	TPs-VS-EPs	0.005481531	0.019290774	TPs
GH109	TPs-VS-EPs	0.018738635	0.058121529	TPs
GH67	TPs-VS-EPs	0.03874687	0.102763437	TPs
GH171	TPs-VS-EPs	0.001126124	0.005569749	TPs
PL0	TPs-VS-EPs	0.036759964	0.101591174	TPs
GH57	TPs-VS-EPs	0.001157184	0.005572755	TPs
GH154	TPs-VS-EPs	0.000107844	0.000819253	TPs
GH20	TPs-VS-EPs	0.022364273	0.067092818	TPs
GH77	TPs-VS-EPs	0.002910362	0.011835474	TPs
GH39	TPs-VS-EPs	1.79E-05	0.000218671	TPs
GH127	TPs-VS-EPs	3.48E-07	7.95E-06	TPs
GH115	TPs-VS-EPs	4.56E-05	0.000439522	TPs
CE17	TPs-VS-EPs	2.91E-05	0.000313719	TPs
GH53	TPs-VS-CPs	2.07E-08	4.73E-07	TPs
GH140	TPs-VS-CPs	0.001821994	0.006946351	TPs
GH3	TPs-VS-CPs	0.015686671	0.03779572	TPs
AA4	TPs-VS-CPs	0.011710445	0.03221518	TPs
GH29	TPs-VS-CPs	0.00294106	0.010350268	TPs
PL27	TPs-VS-CPs	0.008716745	0.02532007	TPs
GH73	TPs-VS-CPs	0.006899034	0.021398698	TPs
GH30	TPs-VS-CPs	0.015329484	0.03779572	TPs
PL35	TPs-VS-CPs	0.012496847	0.032485393	TPs
GH31	TPs-VS-CPs	2.68E-05	0.0002043	TPs
GH112	TPs-VS-CPs	0.019011831	0.045183963	TPs
PL10	TPs-VS-CPs	0.011794629	0.03221518	TPs
GH16	TPs-VS-CPs	0.000428119	0.002117455	TPs
CE11	TPs-VS-CPs	2.06E-10	9.43E-09	TPs
CE6	TPs-VS-CPs	0.011710445	0.03221518	TPs
GH102	TPs-VS-CPs	0.006327688	0.020315207	TPs
GH88	TPs-VS-CPs	8.66E-08	1.76E-06	TPs
GH36	TPs-VS-CPs	4.80E-07	5.85E-06	TPs
PL26	TPs-VS-CPs	0.001499646	0.005965985	TPs

CAZy Family	Compared groups	p value	Fdr	Enriched Group
PL1	TPs-VS-CPs	1.86E-09	6.81E-08	TPs
GH38	TPs-VS-CPs	1.04E-05	9.08E-05	CPs
GH28	TPs-VS-CPs	0.008375934	0.024722515	TPs
GH55	TPs-VS-CPs	0.04659256	0.100311041	TPs
GH105	TPs-VS-CPs	1.40E-12	8.53E-11	TPs
GH84	TPs-VS-CPs	0.006458945	0.020379084	CPs
PL9	TPs-VS-CPs	6.26E-07	7.01E-06	TPs
GH95	TPs-VS-CPs	1.40E-05	0.000116471	TPs
GH133	TPs-VS-CPs	1.95E-06	1.87E-05	TPs
AA7	TPs-VS-CPs	0.012095901	0.032389703	CPs
CE19	TPs-VS-CPs	6.64E-05	0.00041917	TPs
GH120	TPs-VS-CPs	0.013423817	0.034118867	TPs
GH152	TPs-VS-CPs	0.000119531	0.000729139	CPs
GH10	TPs-VS-CPs	0.00496026	0.01620942	TPs
GH106	TPs-VS-CPs	0.007766134	0.023470745	TPs
cohesin	TPs-VS-CPs	0.015531942	0.03779572	CPs
GH68	TPs-VS-CPs	0.022946451	0.052099728	CPs
CE2	TPs-VS-CPs	0.001198884	0.004986269	TPs
PL40	TPs-VS-CPs	0.000361298	0.001943579	TPs
GH35	TPs-VS-CPs	3.36E-09	8.78E-08	TPs
GH59	TPs-VS-CPs	0.001077129	0.004584063	TPs
CE7	TPs-VS-CPs	0.003430456	0.011844781	TPs
PL11	TPs-VS-CPs	0.000382343	0.001943579	TPs
GH156	TPs-VS-CPs	0.045540339	0.099212881	CPs
GH89	TPs-VS-CPs	0.012212511	0.032389703	TPs
GH103	TPs-VS-CPs	0.000767769	0.003602609	TPs
GH2	TPs-VS-CPs	0.000300815	0.001720287	TPs
PL33	TPs-VS-CPs	1.17E-07	1.94E-06	TPs
PL8	TPs-VS-CPs	0.000376618	0.001943579	TPs
GH26	TPs-VS-CPs	1.72E-05	0.000136566	TPs
GH146	TPs-VS-CPs	0.000688822	0.003317223	TPs
PL29	TPs-VS-CPs	0.002933682	0.010350268	TPs
GH97	TPs-VS-CPs	1.67E-06	1.70E-05	TPs
GH143	TPs-VS-CPs	0.019997522	0.046917262	TPs
GH78	TPs-VS-CPs	5.72E-05	0.000393521	TPs
CE12	TPs-VS-CPs	3.21E-17	2.94E-15	TPs
GH43	TPs-VS-CPs	2.91E-18	5.32E-16	TPs
GH33	TPs-VS-CPs	0.012603622	0.032485393	TPs
GH170	TPs-VS-CPs	0.015696583	0.03779572	CPs
GH13	TPs-VS-CPs	0.001821394	0.006946351	TPs
SLH	TPs-VS-CPs	0.035415559	0.078084908	CPs
GH128	TPs-VS-CPs	0.002766978	0.010333817	TPs
GH32	TPs-VS-CPs	4.30E-07	5.62E-06	TPs
GH24	TPs-VS-CPs	0.023060535	0.052099728	CPs
GH42	TPs-VS-CPs	0.003541428	0.012001505	TPs
GH51	TPs-VS-CPs	2.52E-09	7.67E-08	TPs
CE14	TPs-VS-CPs	0.001452191	0.005905577	CPs
CE1	TPs-VS-CPs	1.08E-07	1.94E-06	TPs
GH65	TPs-VS-CPs	0.027347021	0.061030548	CPs
GH110	TPs-VS-CPs	6.02E-05	0.000393521	TPs
AA10	TPs-VS-CPs	0.022946451	0.052099728	CPs
GH92	TPs-VS-CPs	2.79E-05	0.000204486	TPs
GH113	TPs-VS-CPs	0.002875975	0.010350268	TPs
CE3	TPs-VS-CPs	0.003681552	0.012249527	TPs
GH109	TPs-VS-CPs	0.008974599	0.025661743	TPs
PL0	TPs-VS-CPs	0.007823582	0.023470745	TPs
GH57	TPs-VS-CPs	0.000819455	0.003749009	TPs
GH154	TPs-VS-CPs	5.83E-05	0.000393521	TPs
GH101	TPs-VS-CPs	0.000369781	0.001943579	TPs
GH166	TPs-VS-CPs	0.00096019	0.004183686	TPs
GH77	TPs-VS-CPs	2.47E-06	2.26E-05	TPs
GH39	TPs-VS-CPs	2.57E-07	3.74E-06	TPs
GH127	TPs-VS-CPs	6.51E-07	7.01E-06	TPs
GH115	TPs-VS-CPs	2.65E-07	3.74E-06	TPs
AA3	TPs-VS-CPs	0.000862838	0.003851203	CPs
CE17	TPs-VS-CPs	0.000291982	0.001720287	TPs
GH53	EPs-VS-CPs	0.000119151	0.021804665	EPs
GH140	EPs-VS-CPs	0.04251895	0.28053735	EPs
GH124	EPs-VS-CPs	0.014250053	0.165982884	CPs
GH73	EPs-VS-CPs	0.001353332	0.049531954	EPs
PL4	EPs-VS-CPs	0.03992176	0.28053735	EPs
GH31	EPs-VS-CPs	0.034382561	0.251680349	EPs
GH16	EPs-VS-CPs	0.003202579	0.07728063	EPs
CE11	EPs-VS-CPs	0.001273089	0.049531954	EPs
GH116	EPs-VS-CPs	0.042923748	0.28053735	CPs
GH36	EPs-VS-CPs	0.02592959	0.225957852	EPs
GH74	EPs-VS-CPs	0.029623758	0.235702075	CPs
PL9	EPs-VS-CPs	0.013528401	0.165982884	EPs
GH120	EPs-VS-CPs	0.003800687	0.07728063	CPs
GH152	EPs-VS-CPs	0.029506045	0.235702075	CPs
GH138	EPs-VS-CPs	0.049262644	0.300502131	EPs
cohesin	EPs-VS-CPs	0.001081169	0.049531954	CPs
GH99	EPs-VS-CPs	0.023756701	0.217373811	CPs
GH2	EPs-VS-CPs	0.01271761	0.165982884	EPs
GH143	EPs-VS-CPs	0.003020763	0.07728063	EPs
GH48	EPs-VS-CPs	0.046149077	0.291216587	CPs
CE12	EPs-VS-CPs	0.01540738	0.165982884	EPs
GH170	EPs-VS-CPs	0.003655906	0.07728063	EPs
GH13	EPs-VS-CPs	0.033502165	0.251680349	EPs
GH32	EPs-VS-CPs	0.000280286	0.025646191	EPs
GH24	EPs-VS-CPs	0.015419175	0.165982884	CPs
GH148	EPs-VS-CPs	0.022556691	0.217256555	CPs
GH49	EPs-VS-CPs	0.022389597	0.217256555	CPs
GH101	EPs-VS-CPs	0.006852328	0.113997827	EPs
GH77	EPs-VS-CPs	0.011242974	0.165982884	EPs
AA3	EPs-VS-CPs	0.006046979	0.110659716	CPs

Supplementary Table 6. The enrichment analysis of pathways in two phyla

Phylum	Total KOs	Kos ratio	p values	Fdr	Pathway Information	Pathway level 2
Fibrobacterota	1338	0.382352941	4.16855E-12	1.7091E-09	Biosynthesis of amino acids	Global and overview maps
Fibrobacterota	1375	0.377622378	1.45444E-10	2.98161E-08	Ribosome	Translation
Fibrobacterota	1208	0.10508797	1.91535E-09	2.61764E-07	Biosynthesis of secondary metabolites	Global and overview maps
Fibrobacterota	1322	0.285333333	1.17182E-08	1.20111E-06	Biosynthesis of cofactors	Global and overview maps
Fibrobacterota	1393	0.072144289	3.8604E-06	0.000316553	Two-component system	Signal transduction
Fibrobacterota	1404	0.378787879	5.93953E-06	0.000405868	Aminoacyl-tRNA biosynthesis	Translation
Fibrobacterota	1339	0.071713147	2.89768E-05	0.001633334	Microbial metabolism in diverse environments	Global and overview maps
Fibrobacterota	1408	0.291666667	3.18699E-05	0.001633334	Homologous recombination	Replication and repair
Fibrobacterota	1389	0.077669903	0.000163525	0.007449455	ABC transporters	Membrane transport
Fibrobacterota	1407	0.268292683	0.000204468	0.008383188	2-Oxocarboxylic acid metabolism	Global and overview maps
Fibrobacterota	1412	0.386363636	0.000242784	0.009049232	Mismatch repair	Replication and repair
Fibrobacterota	1416	0.066666667	0.000359419	0.012280142	Methane metabolism	Energy metabolism
Fibrobacterota	1417	0.5	0.000569572	0.017963414	Biotin metabolism	Metabolism of cofactors and vitamins
Fibrobacterota	1414	0.483870968	0.000786864	0.023043876	Cell cycle - Caulobacter	Cell growth and death
Fibrobacterota	1418	0.578947368	0.001583035	0.043269631	Valine, leucine and isoleucine biosynthesis	Amino acid metabolism
Fibrobacterota	1408	0.396226415	0.002695956	0.069083885	Peptidoglycan biosynthesis	Glycan biosynthesis and metabolism
Fibrobacterota	1416	0.333333333	0.003353245	0.080872376	Protein export	Folding, sorting and degradation
Fibrobacterota	1416	0.361111111	0.004406148	0.100362252	Thiamine metabolism	Metabolism of cofactors and vitamins
Fibrobacterota	1429	0	0.005443692	0.117469147	Coronavirus disease - COVID-19	Infectious disease: viral
Fibrobacterota	952	0.102010265	0.00780901	0.160084704	Metabolic pathways	Global and overview maps
Fibrobacterota	1410	0.271428571	0.009663869	0.172475851	Alanine, aspartate and glutamate metabolism	Amino acid metabolism
Fibrobacterota	1417	0.151898734	0.009790293	0.172475851	RNA degradation	Folding, sorting and degradation
Fibrobacterota	1414	0.340909091	0.009821106	0.172475851	Pantothenate and CoA biosynthesis	Metabolism of cofactors and vitamins
Fibrobacterota	1391	0.175115207	0.010096147	0.172475851	Purine metabolism	Nucleotide metabolism
Fibrobacterota	1418	0.261904762	0.013156865	0.212205085	One carbon pool by folate	Metabolism of cofactors and vitamins
Fibrobacterota	1421	0.170212766	0.014197128	0.212205085	Nucleotide excision repair	Replication and repair
Fibrobacterota	1414	0.25	0.014430666	0.212205085	Arginine biosynthesis	Amino acid metabolism
Fibrobacterota	1424	0.050505051	0.014492055	0.212205085	Fluid shear stress and atherosclerosis	Cardiovascular disease
Fibrobacterota	1424	0.069444444	0.018723134	0.264706382	Phosphotransferase system (PTS)	Membrane transport
Fibrobacterota	1402	0.226890756	0.02255505	0.287635226	Nucleotide metabolism	Global and overview maps
Fibrobacterota	1412	0.236111111	0.022709526	0.287635226	Phenylalanine, tyrosine and tryptophan biosynthesis	Amino acid metabolism
Fibrobacterota	1423	0.0625	0.024141856	0.287635226	Propanoate metabolism	Carbohydrate metabolism
Fibrobacterota	1401	0.125560538	0.024975443	0.287635226	Oxidative phosphorylation	Energy metabolism
Fibrobacterota	1416	0.216666667	0.02538623	0.287635226	DNA replication	Replication and repair
Fibrobacterota	1416	0.270833333	0.02538623	0.287635226	Lysine biosynthesis	Amino acid metabolism
Fibrobacterota	1416	0.276595745	0.02538623	0.287635226	Histidine metabolism	Amino acid metabolism
Fibrobacterota	1422	0.291666667	0.025957325	0.287635226	Vitamin B6 metabolism	Metabolism of cofactors and vitamins
Fibrobacterota	1419	0.23255814	0.027959028	0.300395428	Base excision repair	Replication and repair
Fibrobacterota	1425	0.153846154	0.029118431	0.300395428	Antifolate resistance	Drug resistance: antineoplastic
Fibrobacterota	1424	0.023255814	0.029306871	0.300395428	Degradation of aromatic compounds	Global and overview maps
Fibrobacterota	1406	0.18699187	0.031923128	0.319231281	Cysteine and methionine metabolism	Amino acid metabolism
Fibrobacterota	1422	0.333333333	0.034423881	0.336042648	Streptomycin biosynthesis	Biosynthesis of other secondary metabolites
Fibrobacterota	1425	0.072727273	0.035456002	0.338068855	Flagellar assembly	Cell motility
Fibrobacterota	1429	0	0.0471813	0.429874066	Biosynthesis of enediyne antibiotics	Metabolism of terpenoids and polyketides
Fibrobacterota	1429	0	0.0471813	0.429874066	Nonribosomal peptide structures	Metabolism of terpenoids and polyketides
Fibrobacterota	1424	0.087719298	0.048306597	0.430558796	Longevity regulating pathway - worm	Aging
Elusimicrobiota	1018	0.370629371	1.82639E-14	7.48819E-12	Ribosome	Translation
Elusimicrobiota	1054	0.033009709	3.44247E-08	6.99379E-06	ABC transporters	Membrane transport
Elusimicrobiota	1046	0.378787879	5.11741E-08	6.99379E-06	Aminoacyl-tRNA biosynthesis	Translation
Elusimicrobiota	1044	0.435483871	3.13352E-06	0.000291359	Lipopolysaccharide biosynthesis	Glycan biosynthesis and metabolism
Elusimicrobiota	1037	0.285714286	3.55316E-06	0.000291359	Nucleotide metabolism	Global and overview maps
Elusimicrobiota	1028	0.203791469	2.25092E-05	0.001538128	Biosynthesis of nucleotide sugars	Global and overview maps
Elusimicrobiota	1032	0.25	5.81044E-05	0.003403256	Amino sugar and nucleotide sugar metabolism	Carbohydrate metabolism
Elusimicrobiota	1057	0.451612903	0.000147159	0.006790428	Cell cycle - Caulobacter	Cell growth and death
Elusimicrobiota	1055	0.222222222	0.000149058	0.006790428	Homologous recombination	Replication and repair
Elusimicrobiota	1058	0.295454545	0.000773967	0.031564983	Mismatch repair	Replication and repair
Elusimicrobiota	1000	0.056573705	0.000846865	0.031564983	Microbial metabolism in diverse environments	Global and overview maps
Elusimicrobiota	1063	0.126984127	0.00146889	0.050187083	Photosynthesis	Energy metabolism
Elusimicrobiota	1046	0.235849057	0.001861263	0.058701356	Glycolysis / Gluconeogenesis	Carbohydrate metabolism
Elusimicrobiota	1061	0.051282051	0.002051676	0.060084793	Methane metabolism	Energy metabolism
Elusimicrobiota	1068	0.021582734	0.002685396	0.070761037	Porphyrin metabolism	Metabolism of cofactors and vitamins
Elusimicrobiota	1054	0.320754717	0.002761406	0.070761037	Peptidoglycan biosynthesis	Glycan biosynthesis and metabolism
Elusimicrobiota	1058	0.216666667	0.003332703	0.080376957	DNA replication	Replication and repair
Elusimicrobiota	1062	0.28125	0.003713847	0.084593171	Drug metabolism - other enzymes	Xenobiotics biodegradation and metabolism
Elusimicrobiota	1049	0.2	0.006421713	0.136530418	Pyrimidine metabolism	Nucleotide metabolism
Elusimicrobiota	1061	0.256410256	0.00666002	0.136530418	Protein export	Folding, sorting and degradation
Elusimicrobiota	1029	0.176470588	0.007946037	0.155136904	Biosynthesis of amino acids	Global and overview maps
Elusimicrobiota	1052	0.195876289	0.008951226	0.163874969	O-Antigen nucleotide sugar biosynthesis	Glycan biosynthesis and metabolism
Elusimicrobiota	1064	0.14893617	0.009592681	0.163874969	Nucleotide excision repair	Replication and repair
Elusimicrobiota	1064	0.333333333	0.009592681	0.163874969	Streptomycin biosynthesis	Biosynthesis of other secondary metabolites
Elusimicrobiota	1061	0.277777778	0.01040692	0.17067349	Carbon fixation in photosynthetic organisms	Energy metabolism
Elusimicrobiota	1069	0.02247191	0.012438425	0.19425809	Pentose and glucuronate interconversions	Carbohydrate metabolism
Elusimicrobiota	1013	0.154666667	0.013141074	0.19425809	Biosynthesis of cofactors	Global and overview maps
Elusimicrobiota	1062	0.209302326	0.013266406	0.19425809	Base excision repair	Replication and repair
Elusimicrobiota	941	0.061816453	0.015193903	0.21481036	Biosynthesis of secondary metabolites	Global and overview maps
Elusimicrobiota	1060	0.229166667	0.017353591	0.237165741	Lysine biosynthesis	Amino acid metabolism
Elusimicrobiota	1071	0	0.018452302	0.244046577	Coronavirus disease - COVID-19	Infectious disease: viral
Elusimicrobiota	1069	0.036363636	0.023628834	0.30274443	Flagellar assembly	Cell motility
Elusimicrobiota	1031	0.080160321	0.027333734	0.333740542	Two-component system	Signal transduction
Elusimicrobiota	1051	0.183486239	0.027676045	0.333740542	Glycine, serine and threonine metabolism	Amino acid metabolism
Elusimicrobiota	1059	0.107142857	0.029181904	0.341845159	beta-Lactam resistance	Drug resistance: antimicrobial
Elusimicrobiota	1064	0.318181818	0.033594991	0.382537258	Vancomycin resistance	Drug resistance: antimicrobial
Elusimicrobiota	1052	0.179245283	0.034521655	0.382537258	Starch and sucrose metabolism	Carbohydrate metabolism
Elusimicrobiota	1053	0.146341463	0.035457664	0.382569528	Cysteine and methionine metabolism	Amino acid metabolism
Elusimicrobiota	1044	0.124423963	0.038865199	0.400995248	Purine metabolism	Nucleotide metabolism
Elusimicrobiota	1067	0.036697248	0.040234567	0.400995248	Sulfur metabolism	Energy metabolism
Elusimicrobiota	1071	0	0.042201733	0.400995248	Glutathione metabolism	Metabolism of other amino acids
Elusimicrobiota	1061	0.158730159	0.042245338	0.400995248	Terpenoid backbone biosynthesis	Metabolism of terpenoids and polyketides
Elusimicrobiota	1048	0.174242424	0.04257035	0.400995248	Pyruvate metabolism	Carbohydrate metabolism
Elusimicrobiota	1065	0.25	0.043677176	0.400995248	Biotin metabolism	Metabolism of cofactors and vitamins
Elusimicrobiota	1069	0.028985507	0.044011674	0.400995248	Valine, leucine and isoleucine degradation	Amino acid metabolism
Elusimicrobiota	721	0.074850299	0.047065831	0.419499802	Metabolic pathways	Global and overview maps

Supplementary Table 7. Tibetan pig associated pathway KOs and definition

EC	Symbol	KOs	Definition
2.7.1.202	FruA/B	K02770	fructose PTS system EIIBC or EIIC component
2.7.3.9	PtsI	K08483	phosphoenolpyruvate-protein phosphotransferase (PTS system enzyme I)
2.7.1.202	FruB/PtsH	K11183	multiphosphoryl transfer protein
2.7.1.56	FruK	K00882	1-phosphofructokinase
	ManY	K02795	mannose PTS system EIIC component
	ManZ	K02796	mannose PTS system EIID component
2.7.1.191	ManX	K02793/K02794	mannose PTS system EIIA component
2.7.1.211	ScrA	K02810	sucrose PTS system EIIBCA or EIIBC component
2.7.1.199	PtsG	K20118	glucose PTS system EIICBA or EIICB component
5.3.1.9		K01810	glucose-6-phosphate isomerase
2.7.1.11		K00850/ K21071	6-phosphofructokinase
5.3.1.1		K01803	triosephosphate isomerase
1.2.1.12		K00134	glyceraldehyde 3-phosphate dehydrogenase
2.7.2.3		K00927	phosphoglycerate kinase
5.4.2.11		K01834	2,3-bisphosphoglycerate-dependent phosphoglycerate mutase
4.2.1.11		K01689	enolase
2.7.1.40		K00873	pyruvate kinase
4.1.2.13		K01624	fructose-bisphosphate aldolase, class II
2.7.1.1		K00844	hexokinase
1.1.1.27		K00016	K00016 L-lactate dehydrogenase
6.4.1.1		K01958/ K01960	pyruvate carboxylase
2.3.3.1		K01647	citrate synthase
4.2.1.3		K01681	aconitate hydratase
1.1.1.42		K00031	isocitrate dehydrogenase
1.2.7.11		K00174/ K 00175	2-oxoacid oxidoreductase (ferredoxin)
1.2.7.3		K00174/ K 00175/ K00176/ K00177	2-oxoglutarate synthase
2.8.3.18		K18118	succinyl-CoA:acetate CoA-transferase
1.3.5.1/1.3.5.4		K00239/ K 00240/ K00241	succinate dehydrogenase/fumarate reductase
4.2.1.2		K01676/ K 01677/ K01678/ K01679	fumarate hydratase
2.2.1.1		K00615	transketolase
2.5.1.54		K03856	3-deoxy-7-phosphoheptulonate synthase
4.2.3.4		K01735/ K13829	3-dehydroquinate synthase
4.2.1.10		K03785/ K03786/ K13832	3-dehydroquinate dehydratase I
1.1.1.25		K00014/ K13832/	shikimate dehydrogenase
2.7.1.71		K00891/K13829	shikimate kinase
2.5.1.19		K00800	3-phosphoshikimate 1-carboxyvinyltransferase
4.2.3.5		K01736	chorismate synthase
5.1.3.1		K01783	ribulose-phosphate 3-epimerase
5.3.1.6		K01807/ K01808	ribose 5-phosphate isomerase A/B
2.7.6.1		K00948	ribose-phosphate pyrophosphokinase
2.4.2.14		K00764	amidophosphoribosyltransferase
6.3.4.13		K01945	phosphoribosylamine---glycine ligase
2.1.2.2		K11175	phosphoribosylglycinamide formyltransferase
6.3.1.21		K08289	phosphoribosylglycinamide formyltransferase 2
6.3.5.3		K01952	phosphoribosylformylglycinamidine synthase
6.3.3.1		K01933	phosphoribosylformylglycinamidine cyclo-ligase
6.3.2.6		K01923	phosphoribosylaminoimidazole-succinocarboxamide synthase
4.3.2.2		K01756	adenylosuccinate lyase
2.1.2.3		K00602	phosphoribosylaminoimidazolecarboxamide formyltransferase
3.5.4.10		K00602	IMP cyclohydrolase
1.1.1.205		K00088	IMP dehydrogenase
6.3.5.2		K01951	GMP synthase (glutamine-hydrolysing)
2.7.4.8		K00942	guanylate kinase
2.7.4.6		K00940	nucleoside-diphosphate kinase
2.3.1.54		K00656	formate C-acetyltransferase
1.2.7.1 / 1.2.7-		K03737	pyruvate-ferredoxin/flavodoxin oxidoreductase
1.2.7.11		K00174/ K00175	2-oxoacid oxidoreductase (ferredoxin)
1.2.1.10		K04072	acetaldehyde dehydrogenase / alcohol dehydrogenase
1.1.1.1		K00001	alcohol dehydrogenase
2.3.1.8		K13788/ K 00625	phosphate acetyltransferase
2.7.2.1		K00925	acetate kinase
6.2.1.1		K01895	acetyl-CoA synthetase
2.8.3.1		K01026	propionate CoA-transferase
2.8.3.18		K18118	succinyl-CoA:acetate CoA-transferase
6.2.1.13		K01905	acetate---CoA ligase (ADP-forming) subunit alpha
2.3.1.9		K00626	acetyl-CoA C-acetyltransferase
1.1.1.157		K00074	3-hydroxybutyryl-CoA dehydrogenase
4.2.1.17		K01715	enoyl-CoA hydratase
1.3.1.44		K00626	trans-2-enoyl-CoA reductase (NAD+)
1.3.8.1		K00248	butyryl-CoA dehydrogenase
2.8.3.8		K01034/ K01035	acetate CoA transferase alpha/beta subunit
2.7.2.7		K00929	butyrate kinase
2.3.1.19		K00634	phosphate butyryltransferase
6.4.1.2		K11263	acetyl-CoA carboxylase
6.2.1.-		K18660	malonyl-CoA/methylmalonyl-CoA synthetase
2.3.1.39	FabD	K00645	[acyl-carrier-protein] S-malonyltransferase
2.3.1.41	FabB	K00647	3-oxoacyl-[acyl-carrier-protein] synthase I
2.3.1.179	FabF	K09458	3-oxoacyl-[acyl-carrier-protein] synthase II
1.1.1.100	FabG	K00059	3-oxoacyl-[acyl-carrier protein] reductase
4.2.1.59	FabZ	K02372/K16363	3-hydroxyacyl-[acyl-carrier-protein] dehydratase
1.3.1.9/1.3.1.44	FabV	K00209	enoyl-[acyl-carrier protein] reductase / trans-2-enoyl-CoA reductase (NAD+)
2.3.1.180	FabH	K00648	3-oxoacyl-[acyl-carrier-protein] synthase III
3.1.2.21		K01071	medium-chain acyl-[acyl-carrier-protein] hydrolase
7.1.1.2	NuoA	K00330	NADH-quinone oxidoreductase subunit A
	NuoB	K00331	NADH-quinone oxidoreductase subunit B
	NuoC	K00332	NADH-quinone oxidoreductase subunit C
	NuoD	K00333	NADH-quinone oxidoreductase subunit D
	NuoE	K00334	NADH-quinone oxidoreductase subunit E
	NuoF	K00335	NADH-quinone oxidoreductase subunit F
	NuoG	K00336	NADH-quinone oxidoreductase subunit G
	NuoH	K00337	NADH-quinone oxidoreductase subunit H
	NuoI	K00338	NADH-quinone oxidoreductase subunit I
	NuoJ	K00339	NADH-quinone oxidoreductase subunit J

EC	Symbol	KOs	Definition
	NuoL	K00341	NADH-quinone oxidoreductase subunit L
	NuoM	K00342	NADH-quinone oxidoreductase subunitM
	NuoN	K00343	NADH-quinone oxidoreductase subunit N
1.3.5.1/	SdhA	K00239	succinate dehydrogenase / fumarate reductase, flavoprotein subunit
1.3.5.4	SdhB	K00240	succinate dehydrogenase / fumarate reductase, iron-sulfur subunit
	SdhC	K00242	succinate dehydrogenase / fumarate reductase, cytochrome b subunit
7.1.2.2/	alpha	K02111	F-type H+/Na+-transporting ATPase subunit alpha
7.2.2.1	beta	K02112	F-type H+/Na+-transporting ATPase subunit beta
	gamma	K02115	F-type H+/Na+-transporting ATPase subunit gamma
	delta	K02113	F-type H+/Na+-transporting ATPase subunit delta
	epsilon	K02114	F-type H+/Na+-transporting ATPase subunit epsilon
	a	K02108	F-type H+-transporting ATPase subunit a
	b	K02109	F-type H+-transporting ATPase subunit b
	c	K02110	F-type H+-transporting ATPase subunit c
7.1.2.2/	A	K02117	V/A-type H+/Na+-transporting ATPase subunit A
7.2.2.1	B	K02118	V/A-type H+/Na+-transporting ATPase subunit B
	D	K02120	V/A-type H+/Na+-transporting ATPase subunit D
	E	K02121	V/A-type H+/Na+-transporting ATPase subunit E
	I	K02124	V/A-type H+/Na+-transporting ATPase subunit I
	K	K02124	V/A-type H+/Na+-transporting ATPase subunit K
7.5.2.-	MalK	K10111	multiple sugar transport system ATP-binding protein
	MsmX	K10112	multiple sugar transport system ATP-binding protein
	MsmK	K10112	multiple sugar transport system ATP-binding protein
	MsiK	K10112	multiple sugar transport system ATP-binding protein
2.6.1.2		K14260	alanine transaminase
2.6.1.1		K00812/K00813/ K11358	aspartate aminotransferase, cytoplasmic
6.3.1.1		K01914	aspartate--ammonia ligase
6.3.1.2		K01915	glutamine synthetase
3.5.1.1		K01424	L-asparaginase
2.4.2.17		K00765	ATP phosphoribosyltransferase
3.6.1.31		K11755/K01523	phosphoribosyl-ATP pyrophosphohydrolase
3.5.4.19		K11755/K01496	phosphoribosyl-AMP cyclohydrolase
5.3.1.16		K01814	phosphoribosylformimino-5-aminoimidazole carboxamide ribotide isomerase
4.3.2.10		K02501/K02500	imidazole glycerol-phosphate synthase subunit HisF
4.2.1.19		K01089/K01693	imidazoleglycerol-phosphate dehydratase
2.6.1.9		K00817	histidinol-phosphate aminotransferase
3.1.3.15		K05602/K04486/K01089	histidinol-phosphatase
1.1.1.23		K00013	histidinol dehydrogenase
2.7.2.4		K12524/K00928	aspartate kinase
1.2.1.11		K00133	aspartate-semialdehyde dehydrogenase
4.3.3.7		K01714	4-hydroxy-tetrahydrodipicolinate synthase
1.17.1.8		K00215	4-hydroxy-tetrahydrodipicolinate reductase
2.6.1.83		K10206	LL-diaminopimelate aminotransferase
5.1.1.7		K01778	diaminopimelate epimerase
4.1.1.20		K01586	diaminopimelate decarboxylase
2.6.1.2		K00830	alanine transaminase
2.3.1.1		K00620/K14682	N-acetylglutamate synthase
2.7.2.8		K00930	acetylglutamate kinase
1.2.1.38		K00145	N-acetyl-gamma-glutamyl-phosphate reductase
2.6.1.11		K00821	acetylornithine aminotransferase
2.3.1.35		K00620	glutamate N-acetyltransferase
2.1.3.3		K00611	ornithine carbamoyltransferase
6.3.4.5		K01940	argininosuccinate synthase
4.3.2.1		K01755	argininosuccinate lyase
3.5.3.1		K01476	arginase
4.1.3.27		K13497/K01658/K01657	anthranilate synthase
2.4.2.18		K00766/K13497	anthranilate phosphoribosyltransferase
5.3.1.24		K13498/K01817	phosphoribosylanthranilate isomerase
4.1.1.48		K13498/K01609	indole-3-glycerol phosphate synthase
4.2.1.20		K06001/K01696/K01695	tryptophan synthase alpha chain
1.1.1.95		K00058	D-3-phosphoglycerate dehydrogenase
2.6.1.52		K00831	phosphoserine aminotransferase
3.1.3.3		K02203	phosphoserine phosphatase
1.2.1.11		K00133	aspartate-semialdehyde dehydrogenase
1.1.1.3		K00003	homoserine dehydrogenase
2.7.1.39		K02203	homoserine kinase
4.2.3.1		K01733	threonine synthase
1.1.1.103		K00060	threonine 3-dehydrogenase
4.1.2.48		K01620	threonine aldolase
2.3.1.29		K00639	glycine C-acetyltransferase
2.1.2.1		K00600	glycine hydroxymethyltransferase
1.4.4.2		K00282/K00283	glycine dehydrogenase
2.1.2.10		K00605	aminomethyltransferase
1.8.1.4		K00382	dihydrolipoamide dehydrogenase
	GCSH	K02437	glycine cleavage system H protein
2.2.1.6		K01652/K01653	acetolactate synthase I/II/III large subunit
1.1.1.86		K00053	ketol-acid reductoisomerase
4.2.1.9		K01687	dihydroxy-acid dehydratase
2.6.1.66		K14260/K00835	valine--pyruvate aminotransferase
2.3.1.182		K09011	(R)-citramalate synthase
1.1.1.85		K00052	3-isopropylmalate dehydrogenase
4.3.1.19		K01754	L-threonine ammonia-lyase
4.2.1.35		K01703	(R)-2-methylmalate dehydratase large subunit
4.2.1.9		K01687	dihydroxy-acid dehydratase
2.6.1.42		K00826	branched-chain amino acid aminotransferase
1.1.1.86		K00053	ketol-acid reductoisomerase
2.3.3.13		K01649	2-isopropylmalate synthase
4.2.1.33		K01703/K01704	3-isopropylmalate dehydratase
1.1.1.3		K00003	homoserine dehydrogenase
2.3.1.31		K00641	homoserine O-acetyltransferase/O-succinyltransferase
2.5.1.49		K01740	O-acetylhomoserine (thiol)-lyase
2.1.1.13		K00548	5-methyltetrahydrofolate--homocysteine methyltransferase
3.13.2.1		K01251	adenosylhomocysteinase
2.1.1.37		K00558	DNA (cytosine-5)-methyltransferase 1
2.5.1.6		K00789	S-adenosylmethionine synthetase

EC	Symbol	KOs	Definition
1.1.1.95		K00058	D-3-phosphoglycerate dehydrogenase
2.6.1.52		K00831	phosphoserine aminotransferase
2.5.1.47		K01738	cysteine synthase
2.3.1.30		K00640	serine O-acetyltransferase
5.4.99.5		K14170/K04516/K06208/K04093	chorismate mutase
4.2.1.51		K04518	prephenate dehydratase
2.7.2.11		K00931	glutamate 5-kinase
1.2.1.41		K00147	glutamate-5-semialdehyde dehydrogenase
1.5.1.2		K00286	pyrroline-5-carboxylate reductase
2.6.1.57		K00832	aromatic-amino-acid transaminase
3.5.4.25		K14652	GTP cyclohydrolase II
3.5.4.26		K11752	diaminohydroxyphosphoribosylaminopyrimidine deaminase
1.1.1.193		K11752	5-amino-6-(5-phosphoribosylamino)uracil reductase
3.1.3.104		K21064	5-amino-6-(5-phospho-D-ribitylamino)uracil phosphatase
2.5.1.78		K00794	6,7-dimethyl-8-ribityllumazine synthase
2.5.1.9		K00793	riboflavin synthase
4.1.99.12		K14652	3,4-dihydroxy 2-butanone 4-phosphate synthase
3.5.4.16		K09007/K22391/K01495	GTP cyclohydrolase IA
3.1.3.1		K01077	alkaline phosphatase
4.1.2.25		K01633	dihydroneopterin aldolase
2.7.6.3		K00950	2-amino-4-hydroxy-6-hydroxymethyldihydropteridine diphosphokinase
2.5.1.15		K00796	dihydropteroate synthase
6.3.2.12		K22099/K11754	dihydrofolate synthase
6.3.2.17		K22099/K11754	folylpolyglutamate synthase
1.5.1.3		K00287	dihydrofolate reductase
4.1.99.17		K03147	phosphomethylpyrimidine synthase
2.7.4.7		K00941	phosphomethylpyrimidine kinase
2.8.1.7		K04487	cysteine desulfurase
4.1.99.19		K03150	2-iminoacetate synthase
2.2.1.7		K01662	1-deoxy-D-xylulose-5-phosphate synthase
2.5.1.3		K00788	thiamine-phosphate pyrophosphorylase
3.1.3.100		K06949	thiamine phosphate phosphatase
2.7.7.73		K03148	sulfur carrier protein ThiS adenylyltransferase
2.8.1.10		K03149	thiazole synthase
3.1.3.2		K14379/K09474	acid phosphatase
2.7.1.26		K11753	riboflavin kinase
2.7.7.2		K14656/K11753	FAD synthetase
1.4.3.16		K00278	L-aspartate oxidase
2.4.2.19		K00767	nicotinate-nucleotide pyrophosphorylase(carboxylating)
2.5.1.72		K03517	quinolinate synthase
2.3.1.286		K12410	NAD+-dependent protein deacetylase SIR2
2.7.7.18		K00969	nicotinate-nucleotide adenylyltransferase
3.6.1.22		K03426	NAD+ diphosphatase
2.7.7.1		K06211	nicotinate-nucleotide adenylyltransferase
6.3.5.1		K01950	NAD+ synthase (glutamine-hydrolysing)
2.7.1.23		K00858	NAD+ kinase
2.4.2.1		K03783/K03784	purine-nucleoside phosphorylase
3.1.3.5		K03787/K11751/K01081	5'-nucleotidase
3.5.1.42		K03743/K03742	nicotinamide-nucleotide amidase
2.7.1.22		K06211	nicotinamide
2.2.1.6		K01652/K01653	acetolactate synthase I/II/III large subunit
4.2.1.9		K01687	dihydroxy-acid dehydratase
1.1.1.169		K00077	2-dehydropantoate 2-reductase
6.3.2.1		K01918	pantoate--beta-alanine ligase
2.1.2.11		K00606	3-methyl-2-oxobutanoate hydroxymethyltransferase
1.1.1.86		K00053	ketol-acid reductoisomerase
4.1.1.36		K01598/ K13038	hosphopantothenoylcysteine decarboxylase
2.7.1.24		K00859	dephospho-CoA kinase
6.3.2.5		K13038/K21977	phosphopantothenate---cysteine ligase(CTP)
2.7.7.3		K00954	pantetheine-phosphate adenylyltransferase
2.1.1.197		K02169	malonyl-CoA O-methyltransferase
3.1.1.85		K09789	pimeloyl-[acyl-carrier protein] methyl ester esterase
6.2.1.14		K01906	6-carboxyhexanoate--CoA ligase
2.6.1.62		K00833	adenosylmethionine---8-amino-7-oxononanoate aminotransferase
2.8.1.6		K01012	biotin synthase
2.3.1.47		K00652	8-amino-7-oxononanoate synthase
6.3.3.3		K01935	dethiobiotin synthetase
6.1.1.17		K01885	glutamyl-tRNA synthetase
1.2.1.70		K02492	glutamyl-tRNA reductase
5.4.3.8		K01845	glutamate-1-semialdehyde 2,1-aminomutase
4.2.1.24		K01698	porphobilinogen synthase
2.5.1.61		K01749	hydroxymethylbilane synthase
4.2.1.75		K01719/K13542	uroporphyrinogen-III synthase
2.1.1.107		K13542	uroporphyrin-III C-methyltransferase
1.3.1.76		K02304	precorrin-2 dehydrogenase
4.99.1.4		K02304	sirohydrochlorin ferrochelatase
4.1.1.111		K22225	siroheme decarboxylase
AhbC		K22226	Fe-coproporphyrin III synthase
4.1.1.37		K01599	uroporphyrinogen decarboxylase
1.3.98.3		K02495	oxygen-independent coproporphyrinogen III oxidase
1.3.3.15		K00231	coproporphyrinogen III oxidase
4.99.1.9		K01772	coproporphyrin ferrochelatase
1.3.3.4		K00231	protoporphyrinogen
4.98.1.1		K01772	protoporphyrin
1.3.98.6		K22227	AdoMet-dependent heme synthase

Code

```
# Quality control
java -jar $SOFTWARE/Trimmomatic-0.38/trimmomatic-0.38.jar PE -phred33
$sample_reads1 $sample_reads2 $sample_pair1 $sample_unpair1 $sample_pair2
$sample_unpair2
# Metagenomic assembly
megahit -1 $sample_pair1 -2 $sample_pair2 --min-contig-len 500 -o
$megahit_dir/$sample
# Binning
cd $megahit_dir/$sample
bwa index final.contigs.fa
bwa mem final.contigs.fa $sample_pair1 $sample_pair2 | samtools sort >
$sample.sort.bam
cd $metabat_dir/$sample
$SOFTWARE/metabat/runMetaBat.sh  $megahit_dir/$sample/final.contigs.fa
$megahit_dir/$sample/$sample.sort.bam
#Mapping rate
cd $megahit_dir/$sample
samtools flagstat $sample.sort.bam >$sample.bam.summary
# CheckM
cd $checkm_dir/$sample
checkm lineage_wf -f checkm.txt  -x fa
/$metabat_sample_dir/final.contigs.fa.metabat-bins* ./
#call genes
cd $refinem_dir
$SOFTWARE/refinem/bin/refinem call_genes  -x fa
$checkm_filtered_bins/$sample $refinem_gene_dir/$sample
#taxon_profile
cd $refinem_dir
$SOFTWARE/refinem/bin/refinem taxon_profile $refinem_gene_dir/$sample
$stats_dir/scaffold_stats.tsv
/home/shipeng/yanglili/refinem/refinem/db/gtdb_r80_protein_db.2017-11-
09.faa.dmnd
/home/shipeng/yanglili/refinem/refinem/db/gtdb_r80_taxonomy.2017-12-15.tsv
$taxon_profile_dir/$sample
$SOFTWARE/refinem/bin/refinem taxon_filter $taxon_profile_dir/$sample
$taxon_profile_dir/$sample/taxon_filter.tsv
$SOFTWARE/refinem/bin/refinem filter_bins -x fa
$checkm_filtered_bins/$sample $taxon_profile_dir/$sample/taxon_filter.tsv
$taxon_profile_dir/$sample/$filtered_bin_dir
#16s rRNA
cd $refinem_dir
$SOFTWARE/refinem/bin/refinem ssu_erroneous -x fa
$taxon_profile_dir/$sample/$filtered_bin_dir $taxon_profile_dir/$sample/
/home/shipeng/yanglili/refinem/refinem/db/gtdb_r80_ssu_db.2018-01-18.fna
/home/shipeng/yanglili/refinem/refinem/db/gtdb_r80_taxonomy.2017-12-15.tsv
$ssu_dir/$sample
#Drep
cd $drep-no-gc_dir
$SOFTWARE/Python-3.6.5/bin/dRep dereplicate --P_ani 0.90 --S_ani 0.95
$output_dir  -g input/*.fa --cov_thresh 0.3 --genomeInfo $input-checkm-info
#GTDBtk
gtdbtk classify_wf --genome_dir dereplicated_genomes/ --extension fa --
out_dir dRep95_GTDBtk --keep_intermediates --cpus 60
# Coverage
bwa index all.fa
bwa mem final.contigs.fa $sample_pair1 $sample_pair2 | samtools sort >
$sample.sort.bam
bedtools coverage -a $all.fa.bed -b $sample.sort.bam > $sample_cov_ori

#### R language
# alpha and beta diversity
library(ape)
library(vegan)
library(ggplot2)
library(phyloseq)

bin_tree <- read.tree('cov_filter_2.tree')
rownames(depth_filter_use) <-
gsub('bin_','bin.',rownames(depth_filter_use),fixed=T)
phy_uni <- phyloseq(otu_table(depth_filter_use,taxa_are_rows =
TRUE),phy_tree(bin_tree))
uni_weight_res <- UniFrac(phy_uni,TRUE)

n=adonis(as.dist(uni_weight_res) ~ Region, by = "margin",
           data = sampFile)
get_adjusted_r2 <- function(adonis_object) {
  n_observations <- ncol(adonis_object$coef.sites)
  d_freedom <- adonis_object$aov.tab$Df[1]
  r2 <- adonis_object$aov.tab$R2[1]
  adjusted_r2 <- RsquareAdj(r2, n_observations, d_freedom)
  adjusted_r2
}
R2adj=get_adjusted_r2(n)
pval=n$aov.tab$`Pr(>F)`[1]
```

```
pcoa_unifrac_weight_res <- cmdscale(uni_weight_res,eig=T)
pcoa_unifrac_weight_res_df <- data.frame(sample=meta_use$Sample_ID,
            pc1=pcoa_unifrac_weight_res$points[,1],
            pc2=pcoa_unifrac_weight_res$points[,2],
            Location=meta_use$Region,
            Body_part=meta_use$Body_site)
eig = pcoa_unifrac_weight_res$eig
labx=paste("PCoA 1 (", format(100 * eig[1] / sum(eig),digits = 3), "%)",
sep="")
laby=paste("PCoA 2 (", format(100 * eig[2] / sum(eig),digits = 3), "%)",
sep="")
unifrac_weighted_pic <- ggplot(pcoa_unifrac_weight_res_df,
        aes(x=pc1,y=pc2,color=Location,
         shape=Body_part))+
geom_point()+
labs(x=labx,y=laby,title="Weighted UniFrac PCoA")+
scale_color_brewer(palette='Dark2')+
theme(legend.text=element_text(size=18),legend.title =
element_text(size=20),
   title = element_text(size=22),axis.title.x = element_text(size=18),
   axis.title.y =element_text(size=18) )+
ggtitle(paste( "Weighted UniFrac PCoA P = ",pval," R2adj = ", format(R2adj,
digits = 4),
      sep = ""))+
theme_classic()


NMDS_res <- metaMDS(t(depth_filter_use),trace = F,distance='jaccard')
tmp <- NMDS_res$points
NMDs_res_df <-
data.frame(sample=meta_use$sample,Location=meta_use$Location,Body_part=meta_use$Body_part,NMDS1=tmp[,1],NMDS2=tmp[,2]
)
NMDS_pic <-
ggplot(NMDs_res_df,aes(x=NMDS1,y=NMDS2,color=Location,shape=Body_part))+geom_point()
+
    labs(title="NMDS")+scale_color_brewer(palette='Dark2')+
    theme(legend.text=element_text(size=18),legend.title =
element_text(size=20),
    title = element_text(size=22),axis.title.x = element_text(size=18),
    axis.title.y =element_text(size=18) )

# KEGG enrich
Elu_pth_enrich=read.table("Elusimicrobiota.Fibrobacterota.self.enrich.PTH.final.txt",heade
r = 1,
                            sep="\t",check.names = F)
Elu_pth_enrich=Elu_pth_enrich[Elu_pth_enrich$p_values<0.05,]
Elu_pth_enrich=Elu_pth_enrich[Elu_pth_enrich$enrichGroup=="Group1",]
Elu_pth_enrich=Elu_pth_enrich[!Elu_pth_enrich$Pathway_level2=="Global and
overview maps",]
colr <- colorRampPalette(rev(brewer.pal(12,
"Paired")))(length(unique(Elu_pth_enrich$Pathway_level2)))
axis_colr=colr
names(axis_colr)=unique(Elu_pth_enrich$Pathway_level2)
axis_colr=as.data.frame(axis_colr)
Elu_pth_enrich$axiscol=axis_colr[Elu_pth_enrich$Pathway_level2,"axis_colr"]
axis_colr_df=Elu_pth_enrich$axiscol[order(Elu_pth_enrich$Pathway_Info)]
library(gridExtra)
get_legend<-function(myggplot){
  tmp <- ggplot_gtable(ggplot_build(myggplot))
  leg <- which(sapply(tmp$grobs, function(x) x$name) == "guide-box")
  legend <- tmp$grobs[[leg]]
  return(legend)
}
plot_point = ggplot(Elu_pth_enrich,aes(x=log10(p_values),y=Pathway_Info,
                          color=log10(p_values)))+
  geom_point(aes(size=Group1_ratio))+
  scale_color_gradient(low="darkorchid4", high="plum") +
  theme_minimal()+
  facet_grid(Phylum~.,space = "free",scales = "free_y")
plot_axis_color=ggplot(Elu_pth_enrich,aes(x=log10(p_values),y=Pathway_Info,
                          color=Pathway_level2))+
  geom_label(aes(label=Pathway_Info,x=5,fill=Pathway_level2),
                 colour = "white", fontface = "bold")+
  scale_fill_manual(values = axis_colr$axis_colr )+
  theme_classic()+
  facet_grid(Phylum~.,space = "free",scales = "free_y")+
  theme(axis.text.y=element_blank())
axis_legend=get_legend(plot_axis_color)
grid.arrange(plot_point,plot_axis_color,ncol=2,widths=c(3,3))

# CAZy heatmap
get_cluster_order <- function(input_df){
  axis_orders=list()
  #input_df=sel_CAZy2Bin
  #tmp_dst=as.data.frame(as.matrix(dist(input_df)))
  hc <- hclust(dist(input_df))
```

```
  y_orders=rownames(input_df)[hc$order]
  #tmp_dst=as.data.frame(as.matrix(dist(t(input_df))))
  hc <- hclust(dist(t(input_df)))
  x_orders=colnames(input_df)[hc$order]
  axis_orders[['y_orders']]=y_orders
  axis_orders[['x_orders']]=x_orders
  return(axis_orders)
}
plot_heat=list()
height_heat=c()
for (i in unique(CAZy_Fisher_ck$Compare_groups)){
  ### prepare enriched SGBs and CAZy2SGB data
  #i=unique(CAZy_Fisher_ck$Compare_groups)[1]
  dim(Dep_wilcox_ck_diff)
  ### get enriched SGBs
  sel_SGB_diff=Dep_wilcox_ck_diff[Dep_wilcox_ck_diff$Compare_groups==i,]
  rownames(sel_SGB_diff)=sel_SGB_diff$SGBs
  ### get enriched families in selected groups
  sel_CAZy_diff=CAZy_Fisher_ck[CAZy_Fisher_ck$Compare_groups==i,]
  sel_CAZy_diff=sel_CAZy_diff[sel_CAZy_diff$fdr<0.05,]
  ###### selected group
  sel_EnrichGroup=sub("-VS-.*","",i)
  sel_CAZy_diff=sel_CAZy_diff[sel_CAZy_diff$EnrichGroup==sel_EnrichGroup,]

  ### get profile for heatmap
  ###### basically melted CAZy2SGB profile with additional annotation info
  sel_SGBs=sel_SGB_diff$SGBs
  if(length(sel_SGBs)!=length(unique(sel_SGBs))){print("error!! must check
it")}
  sel_CAZy_Fam=sel_CAZy_diff$CAZy_Family
  if(length(sel_CAZy_Fam)!=length(unique(sel_CAZy_Fam))){print("error!!
must check it")}
  sel_CAZy2Bin=CAZy2Bin[sel_CAZy_Fam,sel_SGBs]
  sel_CAZy2Bin=sel_CAZy2Bin[rowSums(sel_CAZy2Bin)>0,]
  axis_orders=get_cluster_order(sel_CAZy2Bin)
  sel_CAZy2Bin$CAZy=rownames(sel_CAZy2Bin)
  sel_CAZy2Bin4heat=melt(sel_CAZy2Bin,"CAZy")
  colnames(sel_CAZy2Bin4heat)=c("CAZyFam","SGB","Present")

sel_CAZy2Bin4heat$EnrichGroup=sel_SGB_diff[sel_CAZy2Bin4heat$SGB,"EnrichGroup"
]
  sel_CAZy2Bin4heat$CAZyFam=factor(sel_CAZy2Bin4heat$CAZyFam,levels =
axis_orders[['y_orders']])
  sel_CAZy2Bin4heat$SGB=factor(sel_CAZy2Bin4heat$SGB,levels =
axis_orders[['x_orders']])

  plot=
    ggplot(sel_CAZy2Bin4heat, aes(SGB, CAZyFam, fill= Present)) +
    geom_tile(alpha=0.7) +
    facet_grid(~EnrichGroup,scales = "free_x",space = "free_x")+
    #scale_fill_viridis(discrete=TRUE) +
    scale_fill_gradient2(low = "ghostwhite", high = "darkorchid4", mid =
"grey",
                         midpoint = 0.5, limit = c(0,1), space = "Lab",
                         na.value = "grey90",name="Present/Absent")+
    scale_y_discrete(position = "right")+theme_minimal()+
    theme(axis.text.x=element_blank(),legend.position="none")
  plot_heat[[i]]=plot
  height_heat=c(height_heat,length(unique(sel_CAZy2Bin$CAZy)))
    #theme_ipsum()
}
grid.arrange(grobs = plot_heat,heights=(height_heat+6))

# beta distance inter or inner groups
new_meta_use=meta_use
unique(new_meta_use$Body_site) ##"Cecum content" "Stool"
new_meta_use$Body_site=sub(" content","",new_meta_use$Body_site)

new_meta_use$Region[new_meta_use$Body_site == "Cecum"] = "Cecum"}

uni_weight_res_4melt=as.data.frame(as.matrix(uni_weight_res))
uni_weight_res_4melt$Sample_ID=rownames(uni_weight_res_4melt)
melt_WU=melt(uni_weight_res_4melt,id=c("Sample_ID"))
colnames(melt_WU)=c("Sample1","Sample2","Distance")
melt_WU$Group1=new_meta_use[melt_WU$Sample1,"Region"]
melt_WU$Group2=new_meta_use[melt_WU$Sample2,"Region"]
Group_ck1 <- function(X1) {
  if(X1[1] == X1[2]){return(X1[1])}
  else if( "Cecum" %in% X1){
    tmp_group = paste(sort(X1),collapse = "-vs-")
    return(tmp_group)
  }
  else{return("Inter_Region")}
}

Group_ck2 <- function(X1) {
```

```
  if(X1[1] == X1[2]){return(X1[1])}
  else if(( "Cecum" %in% X1)||( "Tibetan" %in% X1)){
    tmp_group = paste(sort(X1),collapse = "-vs-")
    return(tmp_group)
  }
  else{return("Inter_Region")}
}

melt_WU$Group=apply(melt_WU[,c("Group1","Group2")],1,Group_ck2)
melt_WU$Distan_type="WU"

Jac_res_4melt=as.data.frame(as.matrix(dist_df))
Jac_res_4melt$Sample_ID=rownames(Jac_res_4melt)
melt_Jac=melt(Jac_res_4melt,id=c("Sample_ID"))
colnames(melt_Jac)=c("Sample1","Sample2","Distance")
melt_Jac$Group1=new_meta_use[melt_Jac$Sample1,"Region"]
melt_Jac$Group2=new_meta_use[melt_Jac$Sample2,"Region"]
Group_ck <- function(X1) {
  if(X1[1] == X1[2]){return(X1[1])}else{return("Inter_Region")}
}
melt_Jac$Group=apply(melt_Jac[,c("Group1","Group2")],1,Group_ck2)
melt_Jac$Distan_type="JAC"

colnames(melt_WU)
colnames(melt_Jac)
Finsl_melt_dist=rbind(melt_WU,melt_Jac)
unique(Finsl_melt_dist$Group)
Finsl_melt_dist$SuperGroup="Single"
sel_super=grepl("-vs-",Finsl_melt_dist$Group)
Finsl_melt_dist$SuperGroup[sel_super]="Tibetan"
gp_lvls=unique(Finsl_melt_dist$Group)
Finsl_melt_dist$Group=factor(Finsl_melt_dist$Group,
                             levels =gp_lvls)
library(RColorBrewer)
colr <- colorRampPalette(rev(brewer.pal(12, "Paired")))(13)
beta_boxplot <-
ggplot(Finsl_melt_dist,aes(x=Group,y=Distance,fill=Group,col=Group))+
  geom_boxplot(width = .2, fill = "white",
               size = 1.5, outlier.shape = NA)+
  ggdist::stat_halfeye(
    adjust = .33, ## bandwidth
    width = .67,
    color = NA, ## remove slab interval
    position = position_nudge(x = .15)
  )+
  scale_fill_manual(values = colr)+
  #scale_color_brewer(palette='Paired')+
  scale_color_manual(values = colr)+
  facet_wrap(SuperGroup~Distan_type,scales = "free")+
  theme_classic2()+ theme(axis.text.x = element_text(angle = 50, hjust = 1)
```
