# Supplementary material for: Gut microbiome signatures of extreme environment adaption in Tibetan pig
Source: NPJ Biofilms Microbiomes. 2023 May 24;9:27. doi: 10.1038/s41522-023-00395-3 (PMC10209067; doi:10.1038/s41522-023-00395-3)
Supplement: Supplementary file 2 — Reporting Summary [file 41522_2023_395_MOESM2_ESM.pdf]

nature portfolio

Corresponding author(s): Zhigang Zhang

Last updated by author(s): May 4, 2023

Reporting Summary

Nature Portfolio wishes to improve the reproducibility of the work that we publish. This form provides structure for consistency and transparency in reporting. For further information on Nature Portfolio policies, see our Editorial Policies and the Editorial Policy Checklist.

Statistics

For all statistical analyses, confirm that the following items are present in the figure legend, table legend, main text, or Methods section.

n/a	Confirmed	
☐	☒	The exact sample size (*n*) for each experimental group/condition, given as a discrete number and unit of measurement
☐	☒	A statement on whether measurements were taken from distinct samples or whether the same sample was measured repeatedly
☐	☒	The statistical test(s) used AND whether they are one- or two-sided<br>*Only common tests should be described solely by name; describe more complex techniques in the Methods section.*
☒	☐	A description of all covariates tested
☐	☒	A description of any assumptions or corrections, such as tests of normality and adjustment for multiple comparisons
☐	☒	A full description of the statistical parameters including central tendency (e.g. means) or other basic estimates (e.g. regression coefficient) AND variation (e.g. standard deviation) or associated estimates of uncertainty (e.g. confidence intervals)
☐	☒	For null hypothesis testing, the test statistic (e.g. *F*, *t*, *r*) with confidence intervals, effect sizes, degrees of freedom and *P* value noted<br>*Give P values as exact values whenever suitable.*
☒	☐	For Bayesian analysis, information on the choice of priors and Markov chain Monte Carlo settings
☒	☐	For hierarchical and complex designs, identification of the appropriate level for tests and full reporting of outcomes
☒	☐	Estimates of effect sizes (e.g. Cohen's *d*, Pearson's *r*), indicating how they were calculated

Our web collection on statistics for biologists contains articles on many of the points above.

Software and code

Policy information about availability of computer code

Data collection	Sample metagenome sequencing:DNA extractions were performed using the QIAamp DNA Stool Mini Kit (Qiagen, Hilden, Germany) according to the manufacturer's instructions, with additional steps of mechanical disruption and ammonium acetate precipitate protein. The concentration and purity of DNA were measured using the NanoDrop (Thermo Fisher Scientific Inc., Wilmington, DE, USA)) and agarose gel electrophoresis. Metagenome library preparation was conducted in one DNA paired-end library with an insert size of 300 base pairs (bp) for each sample following the manufacturer's instructions (Illumina, San Diego, CA, USA). The sample was sequenced on an Illumina HiSeq X10 platform adopting a 150-bp PE sequencing strategy. All raw reads and publically available reads were filtered to remove low-quality sequences including the ones: (1) more than 40% continuous bases whose quality score was lower than 32-bp; (2) more than 10% N bases. Then the reads were further quality controlled by using Trimmomatic for filtering poor quality reads adapters and lower quality reads55. We obtained a total of 2.3Tb high-quality clean reads for 352 specimens with an average of 6.6 Gb per specimen.
Data analysis	The data analysis was performed by MEGAHIT 1.0, BWA(v.0.7.12), Samtools (v.1.9), Bedtools (v.2.27.1), MetaBAT2, CheckM (v.1.0.7), RefineM (v.0.0.14), dRep (v.2.6.2). All software are open source. The detailed information of code used to perform our data analyses is available in the supplementary materials with our manuscript.

For manuscripts utilizing custom algorithms or software that are central to the research but not yet described in published literature, software must be made available to editors and reviewers. We strongly encourage code deposition in a community repository (e.g. GitHub). See the Nature Portfolio guidelines for submitting code & software for further information.

Data

Policy information about availability of data

All manuscripts must include a data availability statement. This statement should provide the following information, where applicable:

- Accession codes, unique identifiers, or web links for publicly available datasets
- A description of any restrictions on data availability
- For clinical datasets or third party data, please ensure that the statement adheres to our policy

Raw sequence data and MAGs profiles have been deposited into the CNGB Sequence Archive (CNSA) of China National GeneBank DataBase (CNGBdb) with accession number CNP0003325. They are also available at https://db.cngb.org/qtp/. The source data underlying Figures 1-2 and Supplementary Figures 1-2 are provided as a Source Data file.

Research involving human participants, their data, or biological material

Policy information about studies with human participants or human data. See also policy information about sex, gender (identity/presentation), and sexual orientation and race, ethnicity and racism.

Reporting on sex and gender	This study does not address this issue
Reporting on race, ethnicity, or other socially relevant groupings	This study does not address this issue
Population characteristics	This study does not address this issue
Recruitment	This study does not address this issue
Ethics oversight	This study does not address this issue

Note that full information on the approval of the study protocol must also be provided in the manuscript.

Field-specific reporting

Please select the one below that is the best fit for your research. If you are not sure, read the appropriate sections before making your selection.

☒ Life sciences ☐ Behavioural & social sciences ☐ Ecological, evolutionary & environmental sciences

For a reference copy of the document with all sections, see nature.com/documents/nr-reporting-summary-flat.pdf

Life sciences study design

All studies must disclose on these points even when the disclosure is negative.

Sample size	Sample size was chosen based on previous experience and standards in the field. We collected 34 Fresh cecum content samples and 31 fecal samples from 47 freely grazing Tibetan pigs. 287 fecal samples from low-altitude captive pig (87 from China - CPs and 200 from Europe - EPs)
Data exclusions	No data exclusions
Replication	Three sample groups were designed in this study. More than 65 samples were repeated in each sample group.
Randomization	The samples were allocated randomly into groups
Blinding	No blinding

Reporting for specific materials, systems and methods

We require information from authors about some types of materials, experimental systems and methods used in many studies. Here, indicate whether each material, system or method listed is relevant to your study. If you are not sure if a list item applies to your research, read the appropriate section before selecting a response.

Materials & experimental systems

n/a	Involved in the study	
☒	☐	Antibodies
☒	☐	Eukaryotic cell lines
☒	☐	Palaeontology and archaeology
☐	☒	Animals and other organisms
☒	☐	Clinical data
☒	☐	Dual use research of concern
☒	☐	Plants

Methods

n/a	Involved in the study	
☒	☐	ChIP-seq
☒	☐	Flow cytometry
☒	☐	MRI-based neuroimaging

Animals and other research organisms

Policy information about studies involving animals; ARRIVE guidelines recommended for reporting animal research, and Sex and Gender in Research

Laboratory animals	The host animals are freely grazing Tibetan pigs located 3,500m above sea level at the Deqen prefecture in Yunnan province in China
Wild animals	This study does not involve wild animals
Reporting on sex	Sex is not considered in this study
Field-collected samples	Samples are collected into sterile collection tubes and immediately frozen in dry ice and then stored at −80°C until use
Ethics oversight	All animal experiment was approved by the Yunnan University Ethical Committee for animal experiments

Note that full information on the approval of the study protocol must also be provided in the manuscript.
